# Identification of the Molecular Determinants Involved in Antimicrobial Activity of Pseudodesmin A, a Cyclic Lipopeptide From the Viscosin Group

**DOI:** 10.3389/fmicb.2020.00646

**Published:** 2020-04-21

**Authors:** Matthias De Vleeschouwer, Tim Van Kersavond, Yentl Verleysen, Davy Sinnaeve, Tom Coenye, José C. Martins, Annemieke Madder

**Affiliations:** ^1^Organic and Biomimetic Chemistry Research Group, Department of Organic and Macromolecular Chemistry, Ghent University, Ghent, Belgium; ^2^NMR and Structure Analysis Research Group, Department of Organic and Macromolecular Chemistry, Ghent University, Ghent, Belgium; ^3^Laboratory of Pharmaceutical Microbiology, Department of Pharmaceutical Analysis, Ghent University, Ghent, Belgium

**Keywords:** lipopeptides, bioactivity, alanine scan, structure-activity relationship, *Pseudomonas*, antimicrobial activity, viscosin group

## Abstract

Cyclic lipo(depsi)peptides (CLiPs) from *Pseudomonas* constitute a class of natural products involved in a broad range of biological functions for their producers. They also display interesting antimicrobial potential including activity against Gram-positive bacteria. Literature has indicated that these compounds can induce membrane permeabilization, possibly through pore-formation, leading to the general view that the cellular membrane constitutes the primary target in their mode of action. In support of this view, we previously demonstrated that the enantiomer of pseudodesmin A, a member of the viscosin group of CLiPs, shows identical activity against a test panel of six Gram-positive bacterial strains. Here, a previously developed total organic synthesis route is used and partly adapted to generate 20 novel pseudodesmin A analogs in an effort to derive links between molecular constitution, structure and activity. From these, the importance of a macrocycle closed by an ester bond as well as a critical length of β-OH fatty acid chain capping the N-terminus is conclusively demonstrated, providing further evidence for the importance of peptide-membrane interactions in the mode of action. Moreover, an alanine scan is used to unearth the contribution of specific amino acid residues to biological activity. Subsequent interpretation in terms of a structural model describing the location and orientation of pseudodesmin A in a membrane environment, allows first insight in the peptide-membrane interactions involved. The biological screening also identified residue positions that appear less sensitive to conservative modifications, allowing the introduction of a non-perturbing tryptophan residue which will pave the way toward biophysical studies using fluorescence spectroscopy.

## Introduction

**FIGURE 1:**
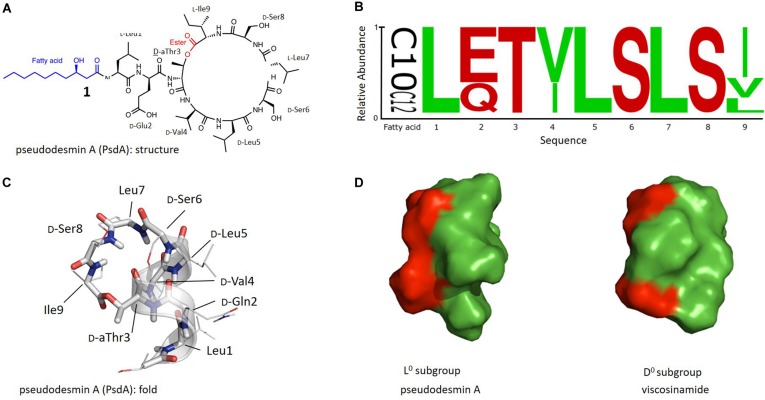
**(A)** Chemical structure of Pseudodesmin A (**1**) also used to illustrate the main aspects of the chemical blueprint of *Pseudomonas* cyclic lipo(depsi)peptides: the ester bond forming the macrocycle (red) and the N-capping fatty acid chain (blue). **(B)** Sequence logo of Ps-CLiPs belonging to the viscosin group including variations in fatty acid length of the 3-hydroxy-fatty acid moiety (C10 vs. C12) (see Supplementary Material for sequence listing). Stereochemistry was not included as these are often made by inference or not available at all in the original literature. **(C)** Overall 3 days fold representing the solution structure of pseudodesmin A in acetonitrile solution, the helix is indicated by a ribbon. **(D)** Solvent-accessible surface representation of Pseudodesmin A and Viscosinamide, with green and red surfaces indicating hydrophobic and hydrophilic residues respectfully. Compared to the orientation in **(C)**, the helix barrel is turned 90° to allow the best presentation of the amphipathic character. In **(C,D)** the 3-OH decanoic acid chain is not shown beyond C3 as its flexibility precludes a definite orientation to be determined.

Antimicrobial resistance is eroding the utility of today’s antibiotics and fuels the renewed drive to discover new antibiotics (Bax et al., 1998; Fischbach and Walsh, 2009; Breidenstein et al., 2011; Laxminarayan et al., 2013). Given the dearth of new broad-spectrum antibiotics in the drug development pipeline, focus has also expanded to the development of narrow-spectrum or even species-specific antibiotics, allowing to create a much more selective arsenal (Lewis, 2013). Antimicrobial peptides or AMP’s have received wide-spread attention as a potential source of such antibiotics (Lewies et al., 2019). The archetypal AMP consists of a linear oligopeptide sequence bearing cationic and hydrophobic residues with a sequence and spatial distribution that generates amphipathic properties once properly folded (Bradshaw, 2003; Jenssen et al., 2006; Huang et al., 2010; Bahar and Ren, 2013). Such folding occurs as part of a variety of modes of action whereby interaction with the bacterial membrane perturbs the latter, ultimately causing lysis and killing the bacteria. Many other classes of AMPs abound in Nature do not fit this ‘archetype’ and appear less prominently in the spotlight of antimicrobial research. Cyclic lipopeptides (CLiPs) typically from *Bacillus* and *Pseudomonas* represent such an alternative class of AMPs (Raaijmakers et al., 2010; Bionda et al., 2013; Geudens and Martins, 2018; Götze and Stallforth, 2019). With few exceptions, the common chemical blueprint of these biosurfactants features an overall negative charge (as opposed to positive), an N-terminal fatty acid chain (as opposed to its absence) typically ten carbons long, while part of the oligopeptide chain forms a macrocycle (as opposed to linear sequences) by ester (depsi-) bond formation involving the C-terminus and an alcohol functionality present in the structure (Figure 1A). In contrast to well-known classes of AMPs, their biosynthesis involves large multi-domain, non-ribosomal peptide synthetases (Raaijmakers et al., 2010; Süssmuth and Mainz, 2017; Götze and Stallforth, 2019), capable of introducing these features as well as the presence of non-proteinogenic amino acids.

In addition to the antimicrobial potency of many of these CLiPs, their broad functional role in a variety of biological processes for their producers is well-established, as clearly lined out in the seminal review of Raaijmakers et al. (2010) and more recently reviewed for *Pseudomonas* CLiPs (Geudens and Martins, 2018; Götze and Stallforth, 2019). Focusing on the latter, *Pseudomonas* CLiPs (Ps-CLiPs) are involved in cell motility, adhesion and biofilm formation (Alsohim et al., 2014; D’aes et al., 2014; Song et al., 2014), but also have functions in bacterial ecology as they promote plant-growth (D’aes et al., 2010; Alsohim et al., 2014) or can trigger defense responses in plants (Nielsen et al., 1999; D’aes et al., 2014). In addition to antibiotic activity (Sinnaeve et al., 2009b; Jang et al., 2013), antagonistic properties including antifungal (Gerard et al., 1997), insecticidal (Groupe et al., 1951), antiviral (Van De Mortel et al., 2009), and anti-oomycete activity have been established (Saini et al., 2008) while more recently antitumor activity has also been reported (Pascual et al., 2014; Cautain et al., 2015; Götze and Stallforth, 2019). Thus, Ps-CLiPs appear as veritable biological ‘swiss-army knives’ (Geudens and Martins, 2018), a trait which is increasingly recognized for AMPs in general (Haney et al., 2019). In view of this, a clear potential exists for biomedical and agricultural applications that invites further exploration using a concerted approach involving structure determination, biophysical investigation and biological evaluation of both natural and synthetic analogs of a representative Ps-CLiP (Geudens et al., 2018). Such an approach requires an efficient (bio-)synthetic route for production of relevant analogs, unavailable from Nature, whose biological evaluation of activity can then be interpreted in terms of a three-dimensional structure in a biologically relevant context. Here, we present such a study for the viscosin group of *Pseudomonas* CliPs, building on our previously reported total synthesis route for pseudodesmin A (**1**, PsdA) (De Vleeschouwer et al., 2014) and our recently obtained understanding of the structure and dynamics of viscosinamide A at the lipid-water interface using its investigation by NMR in a dodecylphosphatidylcholine (DPC):water solution (Geudens et al., 2019).

With 17 characterized members, the viscosin group represents the largest and most extensively characterized Ps-CLiP-group. The ester (depsi) bond cyclizes these nonapeptides between the C-terminus and the alcohol function of the D-*allo*-Thr (D-aThr) sidechain at position 3, thus leading to a 7-residue macrocycle as shown for PsdA (Figure 1A). The N-terminus is generally capped by a 3-hydroxydecanoic acid (3-HDA) with *R* stereochemistry, whenever explicitly reported. The sequence logo (Stephens, 1990) of this group shows a conserved distribution of polar and hydrophobic residues, with conservative substitutions for the latter (Ile/Val) at position 4 and 9 (Figure 1B, original sequences in Supplementary Material). The most notable variations occur independently from each other and consist of a D-Glu/Gln variation at position 2 and a D/L variation of stereochemistry for Leu5. As a result, naturally occurring viscosin group CLiPs can be subdivided in 4 groups (here labeled as L^–^, L^0^, D^–^, and D^0^) depending on overall charge (0 or −1) and stereochemistry (D or L).

We have previously reported the three-dimensional structure of the 2 neutral subgroup representatives, viscosinamide A (D^0^) (Geudens et al., 2014) and pseudodesmin A (L^0^) (Sinnaeve et al., 2009a, b), while that of anionic WLIP (D^–^) was already available from literature (Han et al., 1992). To avoid ambiguity, the three-dimensional structure is referred to hereafter as the ‘fold’ of a Ps-CLiP whereas the chemical (covalent) structure will simply be referred to as ‘structure’ as indicated in Figure 1C. In spite of the D/L switch in stereochemistry for Leu5, all have identical backbone folds, consisting of an N-terminal left-handed α-helix spanning the first six residues, after which the backbone folds back to close the macrocycle through ester bond formation (Figure 1C). This fold organizes all side-chains such that an amphipathic surface results (Figure 1D), with a neater segregation of the polar and apolar surface for the D-subgroup compared to the L-subgroup molecules (Figure 1D) (Geudens et al., 2014). This amphipathic character is fully in line with the current view (obtained from studies of individual Ps-CLiPs) that the most likely mode of action involves direct interaction with the cellular membrane, possibly through pore-formation (Coraiola et al., 2006; Lo Cantore et al., 2006; Reder-Christ et al., 2012). We used fluorescence permeability assays involving model membranes to show that the natural compounds (Geudens et al., 2017), each representing an aforementioned viscosin CLiP subgroup, induce permeabilization at μM concentration, the neutral Ps-CLiPs pseudodesmin A (D^0^) and viscosinamide A (L^0^) being somewhat more potent (C^1/2^∼3.7 μM) than the charged ones WLIP and viscosin (C^1/2^∼4.9 μM). This finding was found to be mostly in line with antibacterial activity against a panel of Gram-positive pathogens (Geudens et al., 2017). Finally, by developing a total chemical synthesis route to viscosin group members, we demonstrated that the biological activity of the enantiomer of PsdA (2) is identical to the natural compound (1), apparently excluding a receptor based interaction as the determining factor for biological activity thus further substantiating the membrane as the primary target (De Vleeschouwer et al., 2014).

To proceed toward full structure-activity studies, total synthesis must be complemented by knowledge of the CLiP fold within the lipid-water interface. To this end, we determined the solution structure and the dynamics of viscosinamide (L^0^) in a DPC-water mixture using NMR spectroscopy and Molecular Dynamics simulations (Geudens et al., 2019). We showed that the solution conformation previously available only from organic solutions or in a crystal environment, is fully maintained in DPC micelles. Paramagnetic relaxation enhancement measurements allowed to locate individual viscosinamide molecules near the water-lipid interface with an insertion depth and overall orientation dictated by the amphipathic molecular properties. Moreover, we established using NMR and ^13^C/^15^N labeled viscosinamide that the hydrogen-bonding network is maintained at the ms to s time scale, indicating a compact and rigid fold in the lipid environment. With the availability of a suitable model for the fold of viscosin group CLiPs in a biologically relevant environment, the solid grounds required to interpret structure-activity related information from a library of pseudodesmin A analogs^1^ is now available, and is reported here, representing the first such studies for *Pseudomonas* CLiPs.

## Materials and Methods

### Synthesis, Purification, and Characterization of PsdA Analogs

Chemicals and general procedures are listed in the Supplementary Materials section. All procedures for the synthesis and purification of specific building blocks as well as solid-phase peptide synthesis, and cyclization release of the PsdA-analogs were carried out as described previously for the total synthesis of pseudodesmin A (**1**) and its enantiomer (**2**) (De Vleeschouwer et al., 2014). Practical details and relevant variations of the procedures for specific cases are collected in the Supplementary Materials section of this paper, together with full characterization of all fatty acid building blocks and analogs. The crude cyclic lipopeptides were purified using either semi-preparative or preparative RP-HPLC. Semi-preparative purification was performed on an Agilent 1100 series instrument using a Kromasil column (C18, 250 mm × 7.8 mm, 5 μm particle size). The analyses were executed with a flow rate of 3 ml/min and with the following solvent systems: H_2_O containing 0.1% HCOOH (A) and CH_3_CN (B). Preparative purification was performed on an Agilent 218 solvent delivery system with a UV-VIS dual wavelength detector using a Phenomenex column [AXIA packed Luna C18(2), 250 ^∗^ 21.2 mm, 5 μm particle size] with a flow rate of 20 ml/min and following solvent systems: H_2_O containing 0.1% TFA (A) and CH_3_CN (B). All LC-MS data were collected with an Agilent 1100 Series HPLC with an ESI detector type VL, equipped with a Kinetex column (C18, 150 mm × 4.60 mm, 5 μm particle size) with a flow rate of 1.5 ml/min. Two different gradients were used: (0–100) and (75–100)%B in 6 min. NMR characterization of intermediates and final building blocks was performed using ^1^H NMR and ^13^C NMR in CDCl_3_ on a Bruker Avance spectrometer equipped with a 5 mm BBO probe and operating at 300 and 75.77 MHz, respectively. High-resolution mass spectra were recorded on an Agilent 6220A time-of-flight mass spectrometer, equipped with an Agilent ESI/APCI multimode source. The ionization mode was set to APCI (atmospheric pressure chemical ionization), while the mass spectra were acquired in 4 GHz high-resolution mode with a mass range set to 3200 Da.

### NMR Characterization of the PsdA-Analogs

Characterization was performed on a Bruker Avance III spectrometer operating at a frequency of 500.13 and 125.76 MHz for ^1^H and ^13^C, respectively, and equipped with a 5 mm ^1^H BBI-Z probe. All NMR measurements on the final compounds were performed on peptide solutions with 600 μl of CD_3_CN, CDCl_3_ or dioxane-*d8* (Eurisotop). High quality HP-7 (New Era Enterprises Inc.) NMR tubes were used. Sample temperature was set to 25°C throughout. 2D spectra measured for structure confirmation include ^1^H-^1^H TOCSY with a 90 ms MLEV-17 spinlock, sensitivity-improved, multiplicity edited, ^1^H-^13^C gHSQC using adiabatic 180° pulses, ^1^H -^1^H NOESY with a 300 ms mixing time and ^1^H-^13^C gHMBC experiments optimized for a ^n^J_CH_ coupling of 8 Hz. Standard pulse sequences as present in the Bruker library were used throughout. Typically, 2048 data points were sampled in the direct dimension for 512 data points in the indirect one, with the spectral width, respectively, set to 12 ppm along the ^1^H dimension and 110 ppm (gHSQC) or 220 ppm (gHMBC) along the ^13^C dimension. For 2D processing, the spectra were zero filled to obtain a 2048 × 2048 real data matrix. Before Fourier transformation, all spectra were multiplied with a squared cosine bell function in both dimensions except for the gHMBC where a squared sine bell was applied together with magnitude calculation to address phase twisted lineshapes. The complete assignments and spectral fingerprints are available as Supplementary Material.

### Biological Activity Assays

Antimicrobial activity of all compounds in Table 1 was tested against *Clostridium perfringens* NCTC 8798, *Enterococcus faecalis* LMG 8222, *Enterococcus faecium* LMG 9431, *Staphylococcus aureus* LMG 10147, *Streptococcus pneumoniae* LMG 21598, and *Streptococcus pyogenes* LMG 15868, with those of **1**, **2**, **11**, **24**, and **25** already reported before in the literature (De Vleeschouwer et al., 2014, 2016, 2017). Strains with LMG designation were obtained from the BCCM/LMG Bacteria Collection (Ghent, Belgium) while strain NCTC 8798 was obtained from the National Collection of Type Cultures (HPA, London, United Kingdom). All strains were grown aerobically at 37°C on Brain Heart Infusion (Oxoid, Erembodegem, Belgium), with the exception of *C. perfringens* NCTC 9789 which was grown aerobically at 37°C on Reinforced Clostridial Medium (Oxoid). Vancomycin (Sigma-Aldrich, St. Louis, MO, United States) and daptomycin (SelleckChem, Boston, MA, United States) were included as reference antibiotics to monitor performance of the assay involving the broth micro-dilution method (Clinical and laboratory standards Institute [CLSI], 2012). The minimal inhibitory concentration that inhibited growth by at least 50% (MIC_1__/__2_) compared to the untreated control was used as a measure of activity. MIC_1/2_s were determined using flat-bottomed 96-well microtiter plates (TPP, Trasadingen, Switzerland). The quantity of CLIP used for biological assays were determined from solutions in CD_3_CN using the digital ERETIC method as available in Topspin 3, based on PULCON (Wider and Dreier, 2006). When insoluble in CD_3_CN, CDCl_3_ was used instead. Following solvent removal, stock solutions were of were obtained by adding appropriate amounts of DMSO. Concentrations of CLIPs and antibiotics tested ranged from 0.016 to 32 μg/ml. The inoculum was standardized at approximately 5 × 10^5^ colony forming units/ml. The plates were incubated at 37°C for 24 h and the optical density was determined at 590 nm using a multi-label microtiter plate reader (Envision Xcite, PerkinElmer LAS, Waltham, MA, United States). In accordance with CLSI guidelines, MIC_1/2_ values were considered identical if they did not differ more than one twofold dilution (Clinical and laboratory standards Institute [CLSI], 2012).

**TABLE 1:** Biological activity of Pseudodesmin A (**1**) and a series of synthetic analogs against a test panel of organisms, used to judge the importance of specific molecular determinants for overall activity.

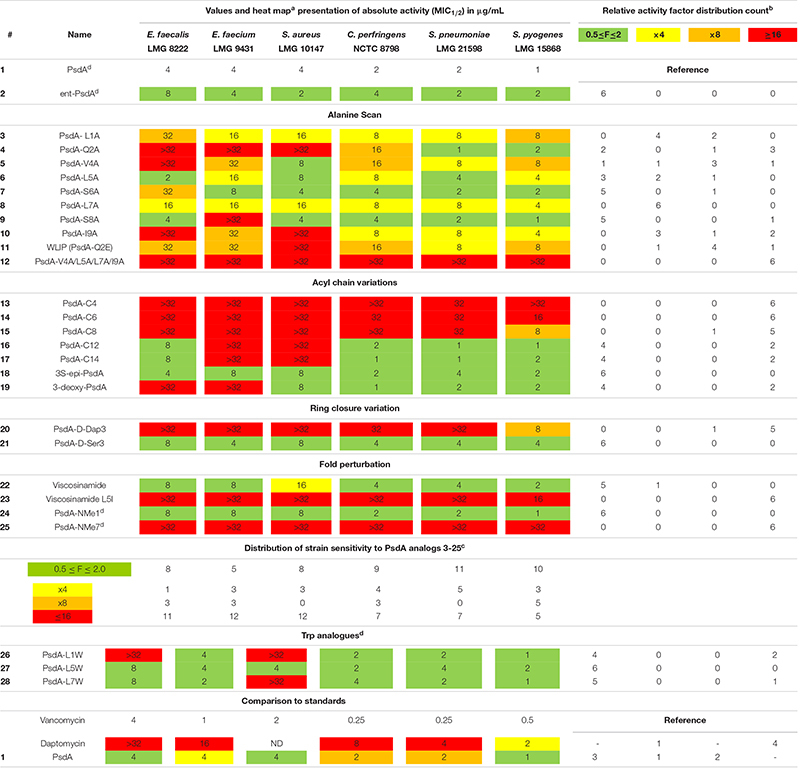

*^*a*^The heat map presentation used here indicates the factor F by which a specific MIC_1/2_ value has changed relative to the chosen reference compound. Within an interval 0.5 ≤ F ≤ 2.0, MIC_1/2_ values are assumed identical to the reference, in accordance with CLSI guidelines (Clinical and laboratory standards Institute [CLSI], 2012) and indicated in green. Factors F of 4, 8, or ≥16 are highlighted using yellow, orange and red heat codes, respectively. ^*b*^Distribution count of relative activity factors corresponding to the number of occurrences of the various heat codes for a particular compound. ^*c*^Distribution of the sensitivity of a strain as indicated by the number of times it showed a change in factor F corresponding to a particular heat code. ^*d*^(De Vleeschouwer et al., 2014, 2017). ¡ltx:attrib¿*

## Results

### Library Set Up and Synthesis of the PsdA Analogs

**FIGURE 2:**
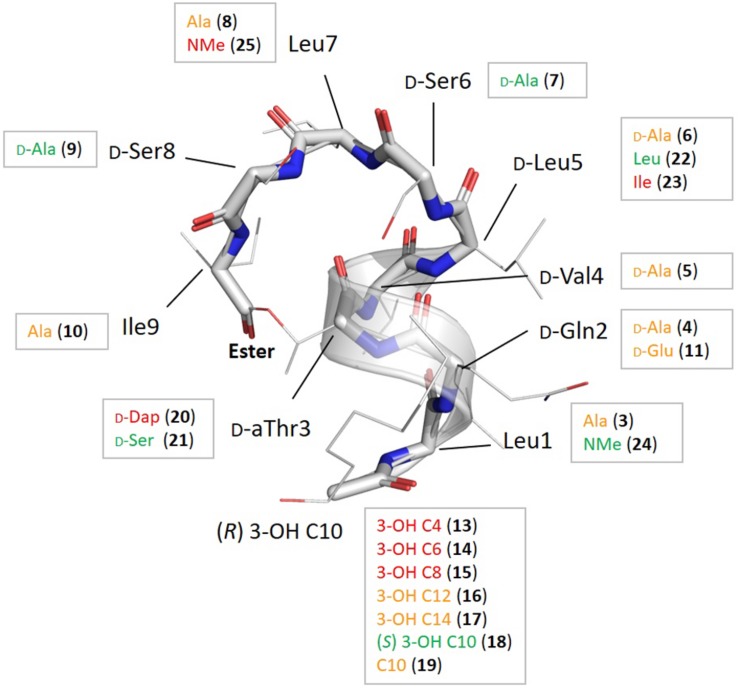
Summary of the chemical modifications with their location shown on the overall fold of pseudodesmin A (1), together with the impact on bioactivity derived from the heat map in Table 1 as follows: if the distribution count of activity factors *F* where 0.5 ≤ *F* ≤ 2.0 for a particular compound is at least 5 (out of 6) the modification is labeled in green as this signifies a particular modification does not result in any significant activity change; if the distribution count of relative activity factors has at least 5 times *F* ≥ 16, the residue is colored red, as this modification causes a major change in activity. All modifications shown in orange indicate points of activity modulation in the fold that are not very significant or do not occur uniformly enough, i.e., they have a low distribution count value.

**FIGURE 3:**
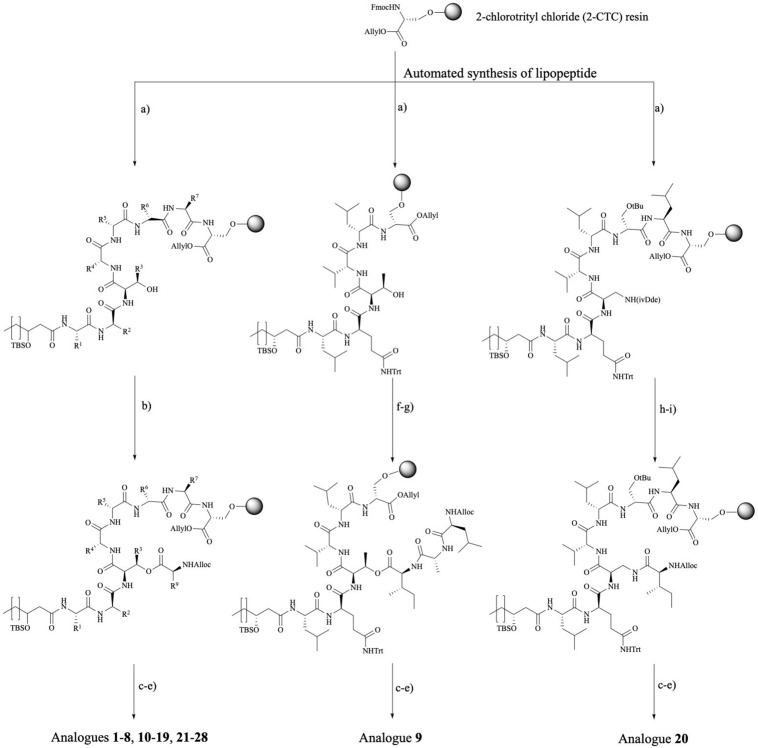
Synthetic route toward pseudodesmin A and analogs. Left branch: Ala-scan (**3**–**10**, **12**), WLIP (11), lipid tail (**13**–**19**), D-Ser3 (**21**), viscosinamide (**22**), viscosinamide L5I (**23**), NMe analogs (**24**–**25**), and Trp analogs (**26**–**28**) – note that the configuration at the 3-hydroxy position is not specified as this branch covers the synthesis of compounds featuring *S* and *R* stereochemistry, respectively. Middle branch: PsdA S8A (**9**) analog. Right branch: amide cyclized D-Dap3-analog (**20**). Reagents and conditions: (a) (i) 20% piperidine, NMP; (ii) Fmoc-AA-OH, HBTU, DIPEA, NMP; (b) Alloc-L-Ile, DIC, pyridine, DMAP, DMF; (c) [Pd(PPh_3_)_4_], PhSiH_3_, DCM; (d) HATU, HOAt, DIPEA, DMF; (e) 0.1 M HCl in HFIP + 1%TIS; (f) Fmoc-L-Ile-OH, DIC, DMAP, pyridine, DMF; (g) (i) 20% piperidine, NMP (ii) Fmoc-D-Ala-OH, HBTU, DIPEA, (iii) 20% piperidine, NMP (iv) Alloc-L-Leu-OH, HBTU, DIPEA; (h) 2% hydrazine, allyl alcohol, NMP (following the standard NovaBiochem protocol, Rohwedder et al., 1998); (i) Alloc-L-Ile-OH, HBTU, DIPEA, and DMF. Full chemical structures for all analogs are collected in an overview table in the Section “Supplementary Material.”

The library of 20 PsdA analogs **3**–**21** and **23** collected in Table 1 is summarized in Figure 2. It was designed to (1) address the possible importance of individual amino acids by using an alanine scan of the sequence, except for the D-aThr3 required for cyclization (**3**–**10**), two additional analogs (**11**–**12**) inspired by the results of the alanine scan; (2) evaluate the role of the fatty acid tail by varying the carbon chain length and the stereochemistry and presence of the 3-hydroxyl functionality (**13**–**19**); (3) establish the importance of the ester bond as ring-closing linkage by introducing an amide (lactam) instead of the ester bond (**20**) and evaluate the influence of a more subtle replacement of D-aThr3 by a D-Ser (**21**) – such modifications have been shown to either increase or abolish activity in other CLiPs (see below) and; (4) assess the importance of maintaining the helical fold by introducing a helix disrupting residue (**22** vs. **23**). As the original synthesis route (Figure 3) was developed to maximize automation and allow facile substitution of the amino acids as well as the lipid moiety, our total synthesis route allowed convenient generation of these 20 novel and non-natural analogs (Table 1), testifying to the versatility of the designed synthesis route. The strategy (left branch, Figure 3) relies on side-chain attachment of the 8th residue to the solid support, invariable a D-serine in the viscosin group CLiPs, with the C-terminus protected via an allyl protecting group, thus allowing to perform an on-resin cyclization in a later stage (Figure 3). For this we successfully introduced a less conventional serine side-chain to resin attachment and subsequently elongated the peptide chain via automated solid phase peptide synthesis protocols, including the enantioselectively synthesized N-terminal (*R*)-3-hydroxydecanoic acid lipid, using the classical Fmoc/*t*Bu protecting strategy. The synthesis of the fatty acid building block, allowing easy variation of both the chain length as well as good control of the stereochemistry, was performed as previously described (De Vleeschouwer et al., 2014). Next, the C-terminal amino acid (Alloc-Ile) was coupled manually through esterification with the D-aThr3 side-chain, thus introducing the depsi bond. After simultaneous removal of the remaining Alloc and Allyl protecting groups, the linear peptide was cyclized through on-resin macrolactamization. After cleavage from the solid support, the desired CLiP was obtained in a very high crude purity, further purified using RP-HPLC and characterized by LC-MS and NMR spectroscopy, relevant details for each compound are available in the supporting information.

The S8A analog **9** and analog **20**, where the ester is replaced by an amide bond, required an adapted synthesis route that will be described in further detail here. As the general synthesis approach relies on the initial attachment of D-serine as future 8th residue, S8A analog **9** cannot be made following the existing route. Therefore, we adapted our strategy without losing the convenient on-resin cyclization step (Figure 3-middle branch) by anchoring the D-Ser6 side-chain instead. In this fashion, many characteristic steps of the general pseudodesmin A synthesis scheme can be maintained. Actually, the same preloaded resin can be used as starting point, followed by automated peptide elongation and on-resin esterification with Fmoc-L-Ile. After esterification with Fmoc-L-Ile, Fmoc-D-Ala8 and then Alloc-L-Leu7 were coupled manually. Subsequent Allyl/Alloc deprotection and on-resin cyclization yielded pseudodesmin S8A **(9)**.

For **20**, where the ester (depsi) bond is substituted by an amide, the amine equivalent of D-*a*Thr would be (2*R*, 3*R*)-diaminobutyric acid. However, this building block equipped with appropriate protecting groups is not commercially available and requires a multistep synthesis (Schmidt et al., 1992). Instead, we chose to use 2,3-diaminopropionic acid (Dap) similar to the previous examples from literature, which, however, implies the loss of the β-methyl group. Since the D-Ser3 PsdA analog **21** where the β-methyl group is absent, is equally active as the natural compound 1 (*vide infra*), our choice for Dap is justified. An additional difficulty arising from the use of Dap is that the two amino groups (α and β) needed to be protected with two different and orthogonal protecting groups (Bionda et al., 2012) used a 4-methyltrityl (Mtt) protecting group which is removed with 1% TFA in DCM (Isidro-llobet et al., 2009). These conditions are, however, not compatible with 2-CTC resin and would cause premature cleavage from the solid the support. In the second example the β-amino was protected with an Alloc group. However, the C-terminal carboxylic acid of Ser8 is Allyl protected, which is not compatible with Alloc-removal. Therefore, we opted for the use of an 1-(4,4-dimethyl-2,6-dioxocyclohexylidene)-3-methylbutyl (ivDde) protecting group which is (quasi-) orthogonal with a Fmoc/tBu/Alloc strategy and is hydrazine labile (Rohwedder et al., 1998). Noteworthy is that this new strategy has an additional degree of orthogonality compared to the original one.

Taking into account the features of the D-Dap(ivDde) building block, a new synthesis route toward PsdA D-Dap3 analog **20** was drafted (Figure 3, right branch). The synthesis started from the same preloaded resin which was elongated in automated fashion to obtain the linear lipopeptide. After removal of the ivDde group, the deprotected β-amino group is coupled with Alloc-L-Ile through the formation of an amide bond. Subsequently, Alloc and Allyl protecting groups are removed followed by on-resin cyclization. After the automated synthesis we detected the formation of an additional cyclic product (the α-amino cyclized isomer of **20**) which resulted from migration of the ivDde protecting group from the side chain to the alpha-amino group during Fmoc deprotection following Dde-Dap introduction (see Supplementary Material) (Wilhelm et al., 2006). However, this side product was easily separated using RP-HPLC from the desired compound 20 that was ultimately obtained in a satisfying yield of 25%, based on the initial loading of the resin (see Supplementary Material).

### Biological Activity and Structure Activity Relationship

#### General Considerations

Prior to biological assays, the chemical identity and integrity of all synthesized PsdA analogs was confirmed by LC-MS and NMR spectroscopy (Supplementary Material). As conservation of the PsdA fold is essential in order to correlate structure to function, significant changes in conformation resulting from the modification were assessed by monitoring changes in ^1^H and ^13^C chemical shifts and ^3^J_H__α HN_ coupling constants in CD_3_CN relative to natural PsdA for each analog (Supplementary Material). Since the PsdA fold comprises a left-handed α-helix extending from Leu1 tot D-Ser6, it is accompanied by a characteristic fingerprint of sequential NH_i_ to NH_i__+__1_ nOe cross-peaks, which was also used to easily check for the preservation of the PsdA fold. As will be detailed further below, all spectroscopic information reveals that the conformation of all novel analogs but one (**23**, *vide infra*) is conserved, placing the discussion of structure-activity relations on a firm basis both in two (structure) and three (fold) dimensions.

Given that PsdA did not show activity against a selection of Gram-negative bacteria (Sinnaeve et al., 2009b), a test panel consisting solely of Gram-positive pathogens was used to create a benchmark for biological activity against which the impact of structural modifications was scored. The selection of six organisms for the panel was not intended to assess the activity spectrum or strength but aimed to provide a means to correlate changes in the activity profile to the structural modifications introduced, while including some representatives of different clinically relevant genera of Gram-positive bacteria. Furthermore, antifungal activity against *Candida albicans* and *Aspergillus fumigatus* was also tested but proved absent (data not shown). All activity tests used a broth microdilution method (Clinical and laboratory standards Institute [CLSI], 2012) with 12 concentrations ranging from 0.016 to 32 μg/mL in twofold dilution steps. From previous screens (De Vleeschouwer et al., 2014), it was already apparent that some of the curves describing the relationship between CLiP activity (i.e., growth inhibition) and concentration were not as clear-cut as those observed for vancomycin and daptomycin (clinically used antibiotics included as controls), but can show a more complicated behavior in which growth is apparently not completely inhibited at higher concentrations, thus leading to difficulties in reproducibility when establishing the MIC value. Therefore, we used the minimal concentration that inhibited growth by at least 50% compared to the untreated control (i.e., MIC1/2) instead as it proved to be a more reliable measure to describe changes in relative potency over the range of compounds (Table 1), enabling sound comparative analysis of the analogs’ performance.

To aid in the interpretation we find it useful to introduce a heat map representation of the values in Table 1, whereby the color coding reports the factor by which a specific structural change has affected the MIC_1/2_ value relative to natural PsdA, considered as the reference. In this heat map, MIC_1/2_ values identical to the reference are indicated in green, as are relative changes by a factor of 0.5 or 2 since CLSI guidelines state that a difference in MIC_1/2_ value corresponding to one twofold dilution should not be considered as a relevant difference (Clinical and laboratory standards Institute [CLSI], 2012). This was the case for the activity of the synthetic versions of WLIP (**11**) (De Vleeschouwer et al., 2016), PsdA (De Vleeschouwer et al., 2014), and (**22**) viscosinamide (data not shown) when compared to their natural counterpart. Significant reductions in activity are color-coded starting from a factor of 4, coded in yellow, followed by a factor of 8 in orange, while any change in relative MIC_1/2_ value by a factor equal to or larger than 16 is coded in red. The table is completed by a frequency count of these factors, giving a more quantitative impression of the performance of a particular compound with respect to the test panel (rows in Table 1) and the sensitivity of a particular organism against changes in PsdA structure (columns in Table 1, *vide infra*). This frequency count was subsequently also used to provide an overview of how specific modifications impact on bioactivity (Figure 2). Finally, as vancomycin and daptomycin were used as controls in all assays, the relative performance of PsdA with respect to these antibiotics against the panel of Gram-positive organisms is also rendered using a heat map, as shown at the bottom of Table 1, now with the color coding based on vancomycin as reference substance. While many other factors of course play a role in determining whether a compound has a potential future as an antibiotic (toxicity, distribution in the body, stability, …) our data indicate that at least in this *in vitro* assay PsdA performs quite adequately when compared to the reference compounds vancomycin and daptomycin.

#### The Role of Individual Amino Acids: Alanine Scan **3**–**10** and Subsequent Analogs **11**–**12**

PsdA analogs **3**–**10** together constitute the alanine scan of the PsdA sequence wherein each amino acid is consecutively substituted by an alanine except for D-alloThr3, for obvious reasons (Figure 2). Inspection of the NMR spectra and assignment data (see Supplementary Material) shows very high similarity in resonance positions for non-substituted amino acid residues, and additional analysis of the NOESY spectra indicates that each substitution has conserved the overall structure, thus allowing assessment of each residue’s contribution to the activity profile. As is evident from the bioactivity screen in Table 1, no single amino acid residue appears to be an absolute determinant for PsdA activity against the test panel. Indeed, no single analog experiences total loss of detectable activity for all six pathogens. Nevertheless, some positions appear more sensitive (MIC_1/2_) or strain-selective (number and extent of strains affected) than others. Thus, substitution at the N- or C-terminal position significantly perturbs activity in all strains. For I9A (**10**) the impact on activity is larger than for L1A (**3**), moving below detectable levels for two strains. Substitution of D-Val4 (**5**) has a similar impact except for *S. aureus* LMG 10147, where activity is retained. As is evident from the activity distribution count in Table 1, the impact of leucine substitutions for alanine at positions 5 and 7 is less pronounced overall, the relative MIC_1/2_ increasing fourfold at most. When substituting D-Ser6 (**7**) for alanine in between these positions, activity remains unchanged except for *E. faecalis*, where it moves close to the detection limit. A similar observation can be made for S8A (**9**) which remains as effective as PsdA in five strains, but displays no detectable activity against *E. faecium*. Finally, substitution of D-Gln2 by D-alanine leads to an interesting analog (**4**) that has reduced activity in four strains, with MIC_1__/__2_ values above 32 μg/ml for three strains. However, for both *Streptococcus* strains, MIC_1/2_ values remain unaffected, again indicating strain-specific differences in susceptibility to the PsdA analogs within the test panel.

It is interesting to note that position 2 represents the spot where the most notable sequence variation occurs within the viscosin group. While most other positions show complete conservation or conservative variations (Figure 1B), position 2 either features a negatively charged D-Glu, like in WLIP(D^–^) or a neutral D-Gln as in PsdA (D^0^). We previously showed (Geudens et al., 2017) that the activity of natural WLIP(D^–^) is attenuated compared to natural PsdA(D^0^), a result which could be reproduced here with synthetic WLIP (**11**) (De Vleeschouwer et al., 2016) which was not assayed before. It would therefore appear that the presence of an uncharged but polar side-chain amide is a trait that confers increased activity against a majority of strains, as compared to a negatively charged or apolar side-chain, respectively.

Simultaneous substitution of the hydrophobic side-chains at positions 1, 4, 7, and 9 by the appropriate D- or L-alanine in compound 12 fully abolishes the activity. The PsdA fold, however, is maintained, as indicated by the characteristic string of sequential nOe’s connecting the amide NH’s from Leu1 to Leu7, and the excellent ^1^H chemical shift dispersion, as also observed in PsdA (Supplementary Material). Even though the amphipathic organization of this PsdA analog therefore appears preserved, a strong and overall reduction in activity is observed. Of all analogs considered it features by far the strongest reduction in overall hydrophobicity as evident from the reduced HPLC retention time of 5.4 min compared to 7.4 min for 1 (see Supplementary Material). This most likely impacts its membrane association and perturbation potential.

#### On the Importance of the Lipid Tail 13–19

The carbon chain length of the fatty acid is an important molecular determinant for activity as immediately apparent from entries for **13** to **17** in Table 1 and Figure 2. Truncation of the lipid tail by replacing the (*R*)-3-HDA with a (*R*)-3-hydroxylated butanoic (C4), hexanoic (C6) or octanoic (C8) chain invariably leads to strong and mostly complete loss of detectable activity (MIC_1/2_ ≥ 32 μg/mL) irrespective of the strain tested. In *S. pyogenes* the effect is also visible but somewhat more gradual, increasing in effect from C8 (**15**) over C6 (**14**) down to a C4 (**13**) fatty acid chain. When longer chains are introduced such as C12 (**16**) and C14 (**17**) the effect is less general, but quite binary: activity is unaffected in four strains, but remains undetectable in *E. faecium* LMG 9431 and *S. aureus* LMG 10197. Not surprisingly, no chemical shift or conformational changes are apparent from the NMR spectra of these analogs as only the N-linked fatty acid is modified. Therefore, these results unambiguously show that a minimal carbon chain length exists for the fatty acid moiety for PsdA to affect Gram-positive bacteria. Also, a decanoic acid chain length appears optimal as the activity is absent for shorter chains, while it is reduced to a smaller number of strains when lengthened. Additional lipid tail analogs **18**–**19** focus on the importance of the 3-hydroxyl group which – whenever reported in literature – always occurs with *R* stereochemistry in natural viscosin group members (De Vleeschouwer et al., 2014). As can be seen from **18**, inversion to *S* does not affect the activity, whereas its complete removal in the deoxy-PsdA analog **19** again gives quite a binary effect, strongly impacting two strains while leaving the other four unaffected.

#### An Ester Rather Than Amide Cyclization Is Required for Antimicrobial Activity

This substitution has already been explored for other CLiPs with mixed results. In the class of fusaricidin analogs from *Bacillus polymyxa*, the substitution of D-aThr by D-Dap [(*R*)-2-diaminopropionic acid] afforded equally potent analogs with improved stability and decreased cytotoxicity (Bionda et al., 2012, 2016), although further analysis indicated that both CLiPs exhibited a significant difference in their mode of action (Hart et al., 2014). Daptomycin analogs in which the L-Thr was replaced by L-Dap have also been made (Hart et al., 2014). This resulted in a dramatic decrease in antimicrobial activity, which the authors attribute to conformational changes or restrictions. As mentioned above, we chose to synthesize the D-Dap3 analog **20**, which should be considered the amide (lactam) analog of a D-Ser3 PsdA rather than natural PsdA which features a D-aThr3, in order to avoid synthesis of the non-commercial building block. To dissect the added impact of the removal of the β-methyl, the D-Ser3 analog **21** was also synthesized and evaluated. Remarkably, whereas **21** has identical biological activity as PsdA, indicating that the loss of the methyl has no impact, complete loss of detectable activity is observed in five out of six strains for the D-Dap3 analog **20**, activity against the sixth strain being strongly reduced as well (Figure 2). Inspection of the NOESY spectrum and comparison to PsdA shows that the overall conformation is not significantly affected compared to PsdA **1** and the D-Ser analog **21**. Considering that ester bonds are generally more sensitive to degradation than amide bonds, this observation is quite remarkable as it indicates that the nature of the ester functionality itself appears to be an important determinant for biological activity.

#### Importance of the Helical Fold for Biological Activity

The main element of the PsdA fold is the N-terminal α_L_ helix – containing D-amino acid residues – which is stabilized through ‘stapling’ of the C-terminus (Ile9) halfway onto the helix (D-aThr3) (Figures 1C, 2). The importance of overall fold integrity for biological activity of PsdA was already disclosed in previous work, where an *N*-methylated PsdA analog with strongly perturbed fold was reported (De Vleeschouwer et al., 2017). Here, we chose a more subtle approach aimed at selectively perturbing the helical fold by exploiting the occurrence of D- and L-Leu5 homologs in Nature giving rise to D^0^ and L^0^ subgroups (*vide supra*). Indeed, despite being present in an otherwise all-D α-helix, an L-Leu5 can populate a small but energetically allowed φφ, ψ region of the Ramachandran map corresponding to a left-handed α_L_ conformation (Richardson and Richardson, 1989). As shown before for viscosinamide (L^0^) (22) the α_L_-helix conformation is fully maintained and despite some reshuffling of the amphipathic interface, identical activity to Psd A (D^0^) (**1**) is observed (Geudens et al., 2014). This is no longer the case, however, when a β-branched L-amino acid, such as Val or Ile is introduced in the all-D α_L_-helix (Figure 2). Now, the side-chain becomes involved in a steric clash with the backbone and is no longer compatible with an α_L_ fold. As a result, a disruption of the helix at this position can be anticipated (Richardson and Richardson, 1989). To minimize spectral overlap for NMR analysis, L-Ile rather than L-Val was introduced at position 5, a D-Val already being present at position 4. The L5I analog **23** was no longer soluble in acetonitrile, requiring the use of DMF as NMR solvent for mutual comparison, thereby already indicating considerable change. Compared to viscosinamide (L^0^) (**22**) in DMF, the amide signals appear broadened and the overall dispersion of the signals is quite different and more convoluted (Supplementary Material). This strongly suggests a disruption of the helical fold, now possibly interconverting between multiple conformations, rather than completely unfolding. The MIC_1/2_ of **23** indicates a marked and uniform reduction in biological activity at the same level as that observed when truncating the fatty acid carbon chain length. This observation is in line with those reported earlier for *N*-methylated PsdA analogs, which were re-assayed here (De Vleeschouwer et al., 2017). While *N*-methylation of the Leu1 amide (**24**) left the activity and conformation unchanged, *N*-methylation of Leu7 (**25**) uniformly abolished activity, as the structural constraints imposed by the inwards facing methyl group and the lack of hydrogen-bond formation potential strongly perturbed and destabilized the overall fold. Together, our results demonstrate that the α-helix plays an important role in the stability of the overall fold and that maintaining its conformation is an absolute prerequisite for biological activity.

## Discussion

**FIGURE 4:**
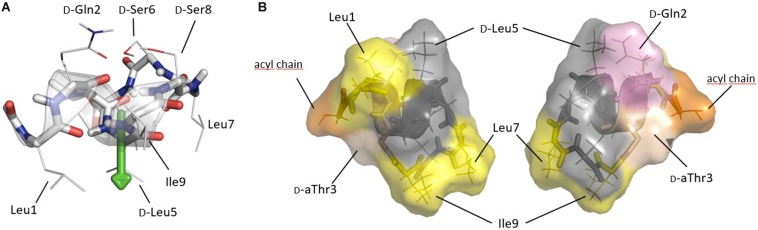
**(A)** Solution conformation of pseudodesmin A (**1**) in acetonitrile (Sinnaeve et al., 2009b; Geudens et al., 2014) displaying the helix as a ribbon and side-chains as indicated. The fatty acid chain is not shown, as it does not adopt a specific conformation. The helix is shown with its main axis oriented horizontally, an orientation parallel to the water(top)/lipid(bottom) interface as experimentally determined for viscosinamide, the L-Leu5 analog of **1**, in DPC micelles. The green arrow indicates the hydrophobic moment which is quasi perpendicular to the helix and points toward the lipid interior. **(B)** Solvent accessible surface representation of pseudodesmin A as seen from a position in the plane of figure **(A)** either from below (left) or above (right) the helix, thus representing opposing views from either inside or outside a DPC micelle. Yellow indicates hydrophobic residues of importance for activity, with pink and orange indicating D-Gln2 and the acyl chain truncated to the C3 atom, respectively. The D-aThr3 featuring the ester bond is shown in pale (salmon) color, while residues of less or no importance for activity are rendered with a gray color. Note that the gray area forms a rim spanning most of the molecule (see text).

Several literature reports have highlighted membrane permeabilization effects for viscosin group CLiPs, possibly through pore formation (Coraiola et al., 2006; Lo Cantore et al., 2006; Reder-Christ et al., 2012). Together with our disclosure that the mirror-image of PsdA (**2**) is equally active as the natural compound (**1**) (De Vleeschouwer et al., 2014), a mechanism of action sensitive to chiral interactions, either with a membrane embedded receptor or with specific lipids (Henriques et al., 2019) appears highly unlikely. Thus, a better understanding of how the peptide constitution and three-dimensional structure combine to interact and perturb the lipid bilayer is an important key to unravel the mode of action. It is important to realize that the impact of peptide-membrane interactions depends both on the local concentration of the active agent in the membrane (i.e., partitioning) and the magnitude of the perturbation the agent subsequently induces in membrane organization and function (Heerklotz and Keller, 2013; Fiedler and Heerklotz, 2015). Since both aspects depend on the peptide as well as on the lipid composition, a straightforward interpretation of the results obtained here in term of structure-activity relations, is a challenging undertaking. This is already apparent from the variation in activity response (MIC_1/2_ values) from the Gram-positive organisms in the test panel. Of the six Gram-positive strains, *E. faecalis* LMG 8222, *E. faecium* LMG 9431, and *S. aureus* LMG 10147 are clearly more sensitive to structural variations in **3**–**21** and **23** compared to the other three strains. Indeed, a count of the number of analogs with modifications that move the activity close to or above the detection threshold (i.e., ≥32 μg/mL) occurs in 53% of the cases for the sensitive strains, a value that halves to 27% for the less sensitive ones. We propose that this division in sensitivity between the groups results, at least in part, from differences in composition of the membrane which is well-known to vary between species. Since our screening aimed to investigate the molecular determinants of activity at the level of the peptide, we used the response of these most sensitive strains to identify loci of importance for bioactivity, whereas the response recorded for the less sensitive strains was used to assess its generality importance. All biological activity results were confronted with the fold of pseudodesmin A (Figure 4B), complemented with information obtained using NMR on the location and orientation of viscosinamide in DPC micelles (Geudens et al., 2017, 2019). While both CLiPs differ by a D-Leu5 to L-Leu5 switch in stereochemistry, they are equally active. Therefore, the impact of this naturally occurring configuration switch on the molecular properties appears limited and therefore of no consequence when matching the activities of pseudodesmin analogs with the behavior of viscosinamide when bound to the membrane.

In DPC/H_2_O solutions of viscosinamide, the amphipathic distribution of residues over opposing faces of the viscosin CLiP fold drives individual molecules to be located near the phosphocholine head groups of DPC molecules. The α-helix orients parallel to the lipid/water interface with the center of mass close to the transition of the polar head-group to the non-polar lipid chain and the hydrophobic moment pointing toward the micelle’s core (Geudens et al., 2019). Applying this orientation to pseudodesmin A (Figure 4A) most hydrophobic side-chains oriented toward the lipid interior (Figure 4B), while the polar residues are exposed to the choline head group and are readily solvent accessible (Figure 4B). In the following, these two opposite viewpoints are used to map the contributions from residues to the biological activity on the pseudodesmin A fold. Not surprisingly, modifications that perturb fold integrity and the associated amphipathicity also abolish biological activity as shown before for N-Me-Leu7 PsdA (**25**) and here now also for L5I PsdA (**23**) because it disrupts the amphipathic profile. In addition, the alanine scan results show that replacing the branched aliphatic side-chain of (iso-)leucine (**3**, **8**, **10**) and valine (**5**) residues by a simple methyl reduces the activity rather uniformly (Figure 4B). The only exception is D-Leu5 (**6**), which appears to be a considerably less sensitive position for alanine substitution, an observation which correlates with the natural occurrence of D/L variations in viscosin group CLiPs. When all these hydrophobic residues are replaced by alanine, a cumulative effect occurs leading to inactivity. This may be caused be a reduced affinity caused by the reduction in hydrophobic surface, while maintaining the polar one, or a decreased membrane perturbation potential resulting from reduced side-chain – lipid chain interactions. Indeed, the compound needs less space to ‘carve into’ the outer lipid bilayer thereby reducing curvature stress over the bilayer, for instance. For the polar residues, only substitution of D-Gln2 causes a marked effect (Figure 4B) although less uniformly so when considering the less sensitive strains. Interestingly, the D-Glu variant (WLIP, **11**), which introduces a negative charge at physiological pH, has an overall reduced activity. Since bacterial membranes carry an overall negative surface charge, it appears most likely that this reduces its partitioning into the lipid bilayer, thus impacting activity. Nevertheless, the presence of the D-Gln2 amide side-chain appears important for activity in its own right as evidenced by the Q2A (**4**) analog, possibly indicating the need for hydrogen-bonding interactions with the phospholipid head group. Finally, the ester bond linkage appears of uniform importance as well, since replacement by an amide causes loss of activity, an observation which is harder to rationalize, given the enhanced chemical stability of the latter. Nevertheless, the positions where biological activity is most sensitive to modification in both sensitive and less-sensitive strains, while maintaining the overall fold, i.e., Leu1, D-Gln2, D-Thr3, Leu7, and Ile9, cluster together (Figure 4B), indicating that residues on this face of the CLiP fold are more important for bioactivity. Interestingly, the other residues form an inert ‘rim’ along the molecular surface. The acyl-chain, is known to penetrate more deeply into the DPC micelles while maintaining flexibility (Geudens et al., 2019) and located in close vicinity to the active face of the molecule. Due to this flexibility, its orientation is not displayed beyond the (*R*)-3-OH, to avoid over-interpretation. Truncation of the acyl chain below ten carbons (**13**–**15**) causes a strong and uniform reduction in activity, with increasing impact as one moves from eight to four carbons (**13**). The impact of acyl chain lengthening (**16**–**18**), on the other hand, is strain specific, as is the configuration (**18**) and presence of the 3-OH group (**19**).

To translate the structure-activity information obtained from our study into a mode of action more targeted biophysical investigations are required. Previously, we used FT-IR and CD spectroscopy to monitor the interaction of viscosin, viscosinamide, WLIP and pseudodesmin in liposomes (Geudens et al., 2017). Fluorescence spectroscopy provides a richer source of information to study peptide-lipid interactions (Ladokhin et al., 2000; Matos et al., 2010; Amaro et al., 2014) but requires the presence of a tryptophan residue. Here, we demonstrate the value of our structure-activity analysis by using these to develop a Trp-analog of pseudodesmin A showing minimal impact on its structural and functional behavior, an absolute prerequisite for meaningful studies. Being an aromatic hydrophobic residue, aliphatic hydrophobic positions were considered for substitutions by tryptophan. Considering the results of the alanine scan, substitution of D-Leu5 appears most suitable, with substitutions of Leu1 and Leu7 representing more sensitive alternatives, Val4 and Ile9 not being considered due to their higher sensitivity to alanine substitution and the presence of β-branched side-chain. The L1W (**26**), L5W (**27**), and L7W (**28**) analogs were synthesized, respecting the L/D/L configuration at these positions. As is evident from Table 1, the biological activity of the L5W analog remains unaffected for all strains, while L1W and LW7 show inactivation for one and two strains, respectively, in good qualitative agreement with expectations. Our results indicate the validity of these Trp analogs for future investigation using fluorescence spectroscopy.

## Conclusion

The specific amphipathic fold of viscosin group CLiPs associated with the depsipeptide cycle, known from X-ray and NMR recently shown to be maintained in DPC/water solutions, together with a minimal acyl chain length which is optimally composed of ten carbons, represent the essential molecular determinants for bioactivity of pseudodesmin A, and by extension, any member of the viscosin group. This is in excellent agreement with the occurrence of peptide-membrane interactions as an important primary step in the mode of action of this group of compounds. Changes at the residue level reduce activity but few cause uniformly strong effects, with residues in the area where the N-terminal exocyclic peptide transitions into the macrocycle, and the lack of a negative charge appearing most important. These insights, together with the possibility to introduce modifications, such as tryptophan residues, for biophysical studies, should pave the way to a deeper understanding of the mode of action of this group of compounds known for antimicrobial, plant biocontrol and more recently antitumor potential (Raaijmakers et al., 2010; Geudens and Martins, 2018; Götze and Stallforth, 2019).

## Data Availability Statement

The datasets generated for this study are available on request to the corresponding author.

## Author Contributions

MD, DS, AM, and JM designed the strategy. MD and TV performed the synthesis under supervision of AM and all chemical characterization under supervision of DS and JM. TC was in charge of antimicrobial screening and interpretation of the outcome. YV performed additional synthesis and biological evaluations, and contributed with MD to the generation of the Supplementary Materials section, as well as to the revision of the manuscript. All authors contributed to the interpretation of (parts) of the data. MD and JM wrote the manuscript. All authors participated in manuscript iteration and final proofreading.

## Conflict of Interest

The authors declare that the research was conducted in the absence of any commercial or financial relationships that could be construed as a potential conflict of interest.

## Footnotes

1 Pseudodesmin A (L^0^) was initially used a lead compound to investigate structure activity relationships, using the solution structure in acetonitrile as guide. Later work demonstrated that in order to establish this solution structure in DPC micelles, isotope labeled compound was an absolute requirement. Preference was given to production of viscosinamide (D^0^), because of the high biosynthetic performance of its bacterial producer (DR54), a trait required when producing isotope labeled compounds from minimal media (Geudens et al., 2019). This was not the case for the original Pseudodesmin A producer which is currently unavailable due to third party patent protection issues.
