# Supplementary material for: Identification of the Molecular Determinants Involved in Antimicrobial Activity of Pseudodesmin A, a Cyclic Lipopeptide From the Viscosin Group
Source: Front Microbiol. 2020 Apr 21;11:646. doi: 10.3389/fmicb.2020.00646 (PMC7187754; doi:10.3389/fmicb.2020.00646)
Supplement: Supplementary file 1 [file Data_Sheet_1.PDF]

Supplementary Material

Table of Contents

Materials and Methods 2
General method for synthesis of pseudodesmin A (and other CLiPs) 5
Side-chain anchoring of Fmoc-D-Ser(OH)-OAllyl (29) 9
Automated synthesis of peptide 30 10
Formation of ester bond (31) 11
Alloc/Allyl deprotection and on-resin cyclization (1) 12
Total deprotection and purification 13
Application of synthesis strategy for the synthesis of CLiP analogues 15
Enantiomer of pseudodesmin A (2) 15
Analogues of the Ala-scan 18
WLIP (11) 34
pseudodesmin V4A L5A L7A I9A (12) 36
Analogues with modified lipid tails 38
pseudodesmin D-Dap3 (20; = substitution of ester bond by amide bond) 52
Single amino acid substitutions 58
Trp-analogues of pseudodesmin 69
NMR characterization of cyclic lipodepsipeptides 1-28 75
Creation of the sequence logo 131
References 132

Materials and Methods

General. 2-chlorotrityl chloride linked polystyrene resin (2-CTC resin) (1.60 mmol/g), all amino acids, HBTU, TFA and Allyloxycarbonyl succinimidyl ester (Alloc-OSu) were purchased from Iris Biotech GmbH. DIC, HOAt, dry DIPEA, piperidine, phenylsilane, triisopropylsilane, (*R*)-3-hydroxybutyric acid, decanoic acid, tetrakistriphenylphosphine palladium (0), (cod)Ru(2-methylallyl)$_2$, (*R*) and (*S*)-BINAP were obtained from Sigma Aldrich (Saint Louis, MO, U.S.A.) and HATU from Merck (Kenilworth, NJ, U.S.A.). Octanoyl chloride, decanoyl chloride, lauroyl chloride, DMAP and 1-methylimidazole were purchased from Acros Organics (Geel, Belgium) and caproic acid and allylbromide from Fluka (Buchs, Switzerland. Butyric acid was provided by VWR (Radnor, PA, U.S.A.) and hydrobromic acid was obtained from Janssen Chimica. (Beerse, Belgium). Peptide grade NMP and DMF obtained from Biosolve (Valkenswaard, The Nederlands) were used throughout the on-resin synthesis and washing of the resin. Dry THF, DMF, MeOH and pyridine were purchased from Acros Organics. All other solvents were supplied by Sigma Aldrich and used without further purification or drying, except for dichloromethane, which was distilled from CaH_2.

LC-MS analysis was performed on an Agilent (Santa Clara, CA, U.S.A) 1100 Series HPLC with an ESI detector type VL, equipped with a Kinetex column (C18, 150 × 4.60 mm, 5 µm particle size) with a flow rate of 1.5 ml/min and a two solvent system was used: 5 mM NH_4OAc in H_2O (A) and CH_3CN (B). Two different gradients were used: (0-100) and (75-100) %B in 6 minutes (0.5 – 6.5 minutes in the chromatogram). The gradient is preceded by 0% A for 0.5 minutes and followed by 100% B for 2 minutes.

Semi-preparative purification Semi-preparative purification was performed on an Agilent 1100 series instrument using a Kromasil column (C18, 250 × 7.8 mm, 5 µm particle size). The analyses were executed with a flow rate of 3 ml/min and with the following solvent systems: H_2O containing 0.1% HCOOH (A) and CH_3CN (B).

Preparative purification was performed on an Agilent 218 solvent delivery system with a UV-VIS dual wavelength detector using a Phenomenex column (AXIA packed Luna C18(2), 250 × 21.2 mm, 5 µm particle size) with a flow rate of 20 ml/min and following solvent systems: H_2O containing 0.1% TFA (A) and CH_3CN (B).

^{1}H NMR and ^{13}C NMR of intermediate products were recorded in $CDCl_3$ on a Bruker Avance spectrometer equipped with a 5 mm BBO probe and operating at 300 MHz and 75.77 MHz respectively. High-resolution mass spectra were recorded on an Agilent 6220A time-of-flight mass spectrometer, equipped with an Agilent ESI/APCI multimode source. The ionization mode was set to APCI (atmospheric pressure chemical ionization), while the mass spectra were acquired in 4 GHz high-resolution mode with a mass range set to 3200 Da.

NMR characterization of the final compounds was performed on a Bruker (Billerica, MA, U.S.A) Avance III spectrometer operating at a frequency of 500.13 MHz and 125.76 MHz for ^{1}H and ^{13}C respectively and equipped with a 5 mm ^{1}H BBI-Z probe. All NMR measurements on the final compounds were performed on peptide solutions with 600 µl of CD_3CN, $CDCl_3$ or dioxane-*d8* (Eurisotop). High quality HP-7 (New Era Ent. Inc) NMR tubes were used. Sample temperature was set to 25°C throughout.

2D spectra measured for structure confirmation include ^1H-^1H TOCSY with a 90 ms MLEV-17 spinlock, sensitivity-improved, multiplicity edited, ^1H-^{13}C gHSQC using adiabatic 180° pulses, ^1H -^1H NOESY with a 300 ms mixing time and ^1H-^{13}C gHMBC experiments optimized for a $^nJ_{CH}$ coupling of 8 Hz. Standard pulse sequences as present in the Bruker library were used throughout. Typically, 2048 data points were sampled in the direct dimension for 512 data points in the indirect one, with the spectral width respectively set to 12 ppm along the ^1H dimension and 110 ppm (gHSQC) or 220 ppm (gHMBC) along the ^{13}C dimension. For 2D processing, the spectra were zero filled to obtain a 2048×2048 real data matrix. Before Fourier transformation, all spectra were multiplied with a squared cosine bell function in both dimensions except for the gHMBC where a squared sine bell was applied together with magnitude calculation to address phase twisted lineshapes.

Solid Phase Peptide Synthesis. Automated peptide synthesis was performed in plastic reaction vessels equipped with Teflon frits (MultiSyn Tech GmbH (Witten, Germany)). The automated peptide synthesis was executed at room temperature with a SYRO II Multiple Peptide Synthesizer Robot, equipped with a vortex unit. The resin was swollen in the solvent used in the reaction for 10 minutes prior to reaction. The solid phase peptide synthesis made use of the ubiquitous Fmoc/tBu protecting strategy with HBTU/DIPEA as coupling reagents. The Fmoc deprotection step was performed by treatment with a solution of 40% piperidine in NMP(v/v) for 4 minutes, after which the deprotection step was repeated for another 12 minutes. The coupling step was carried out on the resin using Fmoc protected amino acids (5 equiv.) dissolved in NMP in the presence of HBTU (5 equiv) and DIPEA (10 equiv.) for 40 minutes at room temperature. The coupling step was repeated for another 40 minutes to ensure complete coupling of the expensive D-amino acids.

For a manual removal of the Fmoc group, the resin was typically treated with 20% piperidine in NMP (v/v) (10 ml/g resin) for 20 minutes, after which the reaction mixture was filtered and the resin was again treated for 20 minutes with 20% piperidine in NMP. Subsequently, the resin was washed with NMP, MeOH and DCM (3×30s, 10 ml/g resin)

For manual coupling of a building block, 5 equiv. of Fmoc protected amino acid (or fatty acid) in DMF (0.5 M), 5 equiv. HBTU in DMF (0.5 M) and 10 equiv. DIPEA (2 M in NMP) were added to the resin. After 2 hours of shaking, the reagent was filtered and the resin was washed. The outcome of the reaction can be monitored with a color test, but mostly a small scale cleavage and subsequent LC-MS analysis on the obtained peptide was preferred.

Intermediate compounds were analyzed by subjecting a small fraction of the peptidyl resin to acidic cleavage. The released peptide was then analyzed by LC-MS. The following small-scale cleavage conditions with TFA were typically applied: 1 mg of peptidyl resin was brought in a plastic reactor vessel equipped with a Teflon frit. A mixture of TFA/triisopropylsilane/water (95/2.5/2.5 %v/v/v) was added and the vessel was shaken for 30 minutes. The resin was washed with TFA (1 ml) and the combined filtrates were dried using an Argon stream to obtain the crude peptide. Subsequently, the peptides are precipitated in cold MTBE while the protecting group debris remains dissolved.

Two different procedures were used for the final cleavage and deprotection step. In the first procedure a solution of TFA/TIS/H_2O (95/2.5/2.5 (v/v/v); 10 ml/g resin) was added to the resin and shaken for 15 minutes. The mixture is filtered and the resin is washed 3 times with 1 ml TFA. The filtrate is collected in a Falcon® tube. The cleavage reaction is repeated twice and the

combined filtrates were dried (using Ar or N_2) to obtain the crude peptide. Thereafter cold MTBE was added to precipitate the peptide and this mixture was centrifuged for 5 minutes, after which the solution containing the protecting group debris was decanted, leaving the crude peptide as a white precipitate. This precipitation procedure is repeated 3 times in total, after which the remaining MTBE is evaporated and the peptide was dried on an oil pump.

In the second procedure a solution of 0.1 M HCl in HFIP (equivalent to 1 ml of ca. 37% aq. HCl per 99 ml HFIP) was used to which 1% v/v TIS was added as scavenger. This solution was added to the resin (10 ml/g resin) and reacted for half an hour. Then the reaction mixture was filtered and the resin was washed two times with 0.1 M HCl in HFIP. All filtrates were collected in a Falcon® tube. The cleavage reaction and washing steps were repeated twice. After a total time of 5h, the combined filtrates were dried using Ar to obtain the crude peptide. An identical workup procedure with MTBE was followed to remove protecting groups.

Synthesis of building blocks. The building blocks necessary for the synthesis of pseudodesmin A (**1**) and its analogues, including Fmoc-D-Ser(OH)-OAll, Alloc-L-Ile, the lipid tail (C10-fragment, (*R*)-3-(tert-butyldimethylsilyloxy)decanoic acid) and their stereoisomers were synthesized according to the methods described in literature.(De Vleeschouwer et al., 2014) For the lipid tail analogues of pseudodesmin A (**13**-**17**), fatty acid moieties with different lengths needed to be synthesized. The C4 fragment which was used for the synthesis of PsdA-C4 (**13**) was made through TBS-protection of the alcohol functionality of the commercially available (*R*)-3-hydroxybutanoic acid. The synthesis of the C6, C8, C12 and C14-fragments, which for all derivatives was earlier described in literature by others, was here performed according to the procedure that we earlier described in literature for the C10-fragment (De Vleeschouwer et al., 2014), starting from an alkanoyl chloride with appropriate length. Chiral HPLC analysis was run on a Daicel Chiracel ODH kolom (250x4.6 mm) with an isocratic elution of hexane/EtOH (97/3) over 30 minutes and a flow rate of 1 ml/min. For each compound except **13** (commercial compound used as such), an enantiomeric excess of 99.0% of the (*R*)-enantiomer was established after the asymmetric hydrogenation step. The obtained NMR spectral data were in accordance with earlier reported data. Optical rotation was measured and found in accordance with previously obtained values in literature (where available).

C4-fragment: (*R*)-3(tert-butyldimethylsilyloxy)butanoic acid: $[\boldsymbol{\alpha}]^{25}_{D}$ (c=0.98, CH_2Cl_2): -4.9, ^{1}H NMR (500 MHz, $CDCl_3$): δ=0.06 (s, 3H; CH_3), 0.07 (s, 3H; CH_3), 0.87 (s, 9H; 3×CH_3), 1.22 (d,3H; CH_3), 2.46 (m, 2H, CH_2), 4.27 (h, ^{3}J(H,H)= 6.2 Hz, 1H; CH), 7.24 ppm (broad,1H; COOH); ^{13}C NMR (125 MHz, $CDCl_3$): δ=-3.0 (CH_3), -5.0 (CH_3), 18.0 (C_q), 24.0 (CH_3), 26.0 (3×CH_3), 45.0 (CH_2), 66.0 (CH), 177.0 ppm (CO).

C6-fragment: (*R*)-3(tert-butyldimethylsilyloxy)hexanoic acid: ^{1}H NMR (500 MHz, $CDCl_3$): δ=0.07 (s, 3H; CH_3), 0.08 (s, 3H; CH_3), 0.88 (s, 9H; 3×CH_3), 0.91 (t,3H; CH_3), 1.35-1.51 (m, 4H, 2×CH_2), 2.47-2.50 (dd, 2H, CH_2), 4.11 (p, ^{3}J(H,H)= 5.92 Hz, 1H; CH), 6.64 ppm (broad,1H; COOH); ^{13}C NMR (125 MHz, $CDCl_3$): δ=-4.2 (CH3), -4.5 (CH_3), 14.0 (CH_3), 18.0 (C_q), 19.0 (CH_2), 26.0 (3×CH_3), 40.0 (CH_2), 42.0 (CH_2), 70.0 (CH), 177.0 ppm (CO). Optical rotation after asymmetric hydrogenation, (*R*)-methyl 3-hydroxyhexanoate: $[\boldsymbol{\alpha}]^{25}_{D}$ (c=1.01, CH_2Cl_2): -24.7, previously reported $[\boldsymbol{\alpha}]^{rt}_{D}$ (c=1.16, $CHCl_3$): -23.3.(Jiang et al., 2010)

C8-fragment: (*R*)-3(tert-butyldimethylsilyloxy)octanoic acid: ^{1}H NMR (500 MHz, $CDCl_3$): δ=0.07 (s, 3H; CH_3), 0.08 (s, 3H; CH_3), 0.87 (s, 9H; 3×CH_3), 0.87 (m, 3H; CH_3), 1.26-1.31 (m, 6H, 3×CH_2), 1.51 (m, 2H, CH_2), 2.46-2.50 (dd, 2H, CH_2), 4.10 (p, ^{3}J(H,H)= 5.97 Hz, 1H; CH), 6.42 ppm (broad,1H; COOH); ^{13}C NMR (125 MHz, $CDCl_3$): δ=-4.2 (CH_3), -4.5 (CH_3), 14.0 (CH_3), 18.0 (C_q), 23.0 (CH_2), 25.0 (CH_2), 26.0 (3×CH_3), 32.0 (CH_2), 38.0 (CH_2), 42.0 (CH_2), 70.0 (CH), 177.0 ppm (CO). $[\boldsymbol{\alpha}]^{25}_{D}$ (c=0.99, CH_2Cl_2): 1.4.

C12-fragment: (*R*)-3(tert-butyldimethylsilyloxy)dodecanoic acid: ^{1}H NMR (500 MHz, $CDCl_3$): δ=0.07 (s, 3H; CH_3), 0.08 (s, 3H; CH_3), 0.88 (s, 9H; 3×CH_3), 0.88 (t, 3H; CH_3), 1.26 (m, 10H, 5×CH_2), 1.29 (m, 2H, CH_2), 1.30 (m, 2H, CH_2), 1.51 (m, 2H, CH_2), 2.46-2.50 (dd, 2H, CH_2), 4.11 (p, ^{3}J(H,H)= 6.05 Hz, 1H; CH), 9.40 ppm (broad,1H; COOH); ^{13}C NMR (125 MHz, $CDCl_3$): δ=-4.0 (CH_3), -5.0 (CH_3), 14.0 (CH_3), 18.0 (C_q), 23.0 (CH_2), 25.0 (CH_2), 26.0 (3×CH_3), 30.0 (4×CH_2), 32.0 (CH_2), 38.0 (CH_2), 42.0 (CH_2), 70.0 (CH), 177.0 ppm (CO). Optical rotation before TBS-protection, (*R*)-3-hydroxydodecanoic acid; $[\boldsymbol{\alpha}]^{25}_{D}$ (c=0.99, CH_2Cl_2): -13.5, previously reported $[\boldsymbol{\alpha}]^{25}_{D}$ (c=1.00, $CHCl_3$): -16.9.(Pirrung et al., 2016)

C14-fragment: (*R*)-3(tert-butyldimethylsilyloxy)tetradecanoic acid: ^{1}H NMR (500 MHz, $CDCl_3$): δ=0.06 (s, 3H; CH_3), 0.08 (s, 3H; CH_3), 0.88 (s, 9H; 3×CH_3), 0.88 (t, 3H; CH_3), 1.26-1.30 (m, 18H, 9×CH_2), 1.51 (m, 2H, CH_2), 2.47-2.51 (dd, 2H, CH_2), 4.11 (p, ^{3}J(H,H)= 6.05 Hz, 1H; CH), 10.88 ppm (broad,1H; COOH); ^{13}C NMR (125 MHz, $CDCl_3$): δ=-4.2 (CH_3), -5.5 (CH_3), 14.0 (CH_3), 18.0 (C_q), 23.0 (CH_2), 25.0 (CH_2), 26.0 (3×CH_3), 30.0 (6×CH_2), 32.0 (CH_2), 38.0 (CH_2), 42.0 (CH_2), 70.0 (CH), 177.0 ppm (CO). Optical rotation before TBS-protection, (*R*)-3-hydroxytetradecanoic acid; $[\boldsymbol{\alpha}]^{25}_{D}$ (c=1.01, CH_2Cl_2): -12.8, previously reported $[\boldsymbol{\alpha}]^{20}_{D}$ (c=1.00, $CHCl_3$): -16.0.(Kücük and Yusufoğlu, 2013)

General method for synthesis of pseudodesmin A (and other CLiPs)

The general approach for the synthesis of pseudodesmin A (PsdA) is well documented and identical procedures were used throughout.(De Vleeschouwer et al., 2014) A more detailed discussion about the design and development of the synthesis strategy can be found there. As this strategy is used for the generation of a library of analogues, its key features are discussed below. The general scheme for the synthesis of pseudodesmin A (**1**) is shown in Scheme S1. For all other analogues (**2-8, 10-19, 21-28**), except pseudodesmin S8A (**9**) and pseudodesmin D-Dap3 (**20**), a similar strategy was used.

The synthesis of WLIP (**11**) appeared less straightforward than expected. The critical point during the synthesis of this analogue was the esterification which turned out to be extremely cumbersome and required additional repetitions of this step. The yield was significantly lower than for other CLiPs, however, we were able to isolate more than sufficient material for NMR characterization and biological activity testing. Nevertheless, as described elsewhere, we also developed a customized synthesis strategy with optimized protecting group choice.(De Vleeschouwer et al., 2016)

The lipid tail analogues of pseudodesmin A (**13-19**) were obtained via coupling of various fatty acid fragments which were synthesized as described above. 3-deoxy-PsdA (**19**) was obtained through coupling of the commercially available decanoic acid to the peptide precursor.

Pseudodesmin NMe1 (**24**) and pseudodesmin NMe7 (**25**) were also made following a similar strategy, as previously reported.(De Vleeschouwer et al., 2017) The N-methylated building block Fmoc-NMe-L-Leu was used throughout.

Table S1: Overview of cyclic lipodepsipeptides that were synthesized according to synthesis scheme in figure 2. The table clarifies the nature of the various R^n groups as shown in Figure 2 of the main text and highlights the differences of each synthetic compound with the main compound PsdA.

N°	Acronym (Name)	Amino acid side chains								
		-ξ-R^1	-ξ-R^2	-ξ-R^3	-ξ-R^4	-ξ-R^5	-ξ-R^6	-ξ-R^7	-ξ-R^8	-ξ-R^9
**1**<br>**2**	PsdA<br>*ent*-PsdA	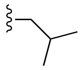	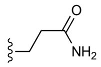	-ξ-CH_3	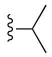	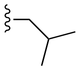	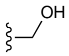	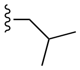	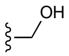	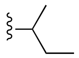
**Alanine Scan**										
		-ξ-R^1	-ξ-R^2	-ξ-R^3	-ξ-R^4	-ξ-R^5	-ξ-R^6	-ξ-R^7	-ξ-R^8	-ξ-R^9
**1**	PsdA	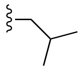	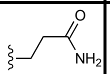	-ξ-CH_3	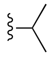	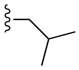	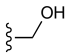	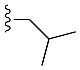	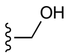	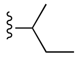
**3**	PsdA- L1A	-ξ-CH_3								
**4**	PsdA-Q2A		-ξ-CH_3							
**5**	PsdA-V4A				-ξ-CH_3					
**6**	PsdA-L5A					-ξ-CH_3				
**7**	PsdA-S6A						-ξ-CH_3			
**8**	PsdA-L7A							-ξ-CH_3		
**9**	PsdA-S8A								-ξ-CH_3	
**10**	PsdA-I9A									-ξ-CH_3
**11**	WLIP (PsdA-Q2E)		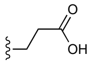							
**12**	PsdA-V4A/L5A/L7A/I9A				-ξ-CH_3	-ξ-CH_3		-ξ-CH_3		-ξ-CH_3

**Acyl chain variation**			
		Chain length (unsaturations are 0)	Additional functionalities
**1**	PsdA	C10	(*R*) 3- OH
**13**	PsdA-C4	C4	
**14**	PsdA-C6	C6	
**15**	PsdA-C8	C8	
**16**	PsdA-C12	C12	
**17**	PsdA-C14	C14	
**18**	3*S*-epi-PsdA		(*S*) 3-OH

**19**	3-deoxy-PsdA		/ (no OH)

Ring closure variation

		Ring closing amino acid	Ring closing functionality
**1**	PsdA	D-aThr	Ester bond
**20**	PsdA-D-Dap3	D-Dap	Amide bond
**21**	PsdA-D-Ser3	D-Ser	

Fold pertubation

		AA^1	AA^2	AA^3	AA^4	AA^5	AA^6	AA^7	AA^8	AA^9
**1**	PsdA	L-Leu	D-Gln	D-aThr	D-Val	D-Leu	D-Ser	L-Leu	D-Ser	L-Ile
**22**	Viscosin-amide					L-Leu				
**23**	Viscosin-amide L5I					L-Ile				
**24**	PsdA-NMe1	NMe-								
**25**	PsdA-NMe7							NMe-		

Trp analogues

		-ξ-R^1	-ξ-R^2	-ξ-R^3	-ξ-R^4	-ξ-R^5	-ξ-R^6	-ξ-R^7	-ξ-R^8	-ξ-R^9
**1**	PsdA	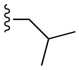	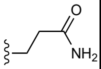	-ξ-CH_3	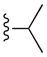	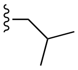	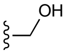	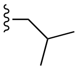	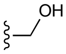	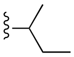
**26**	PsdA-L1W	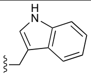								
**27**	PsdA-L5W					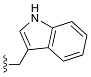				
**28**	PsdA-L7W							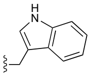		

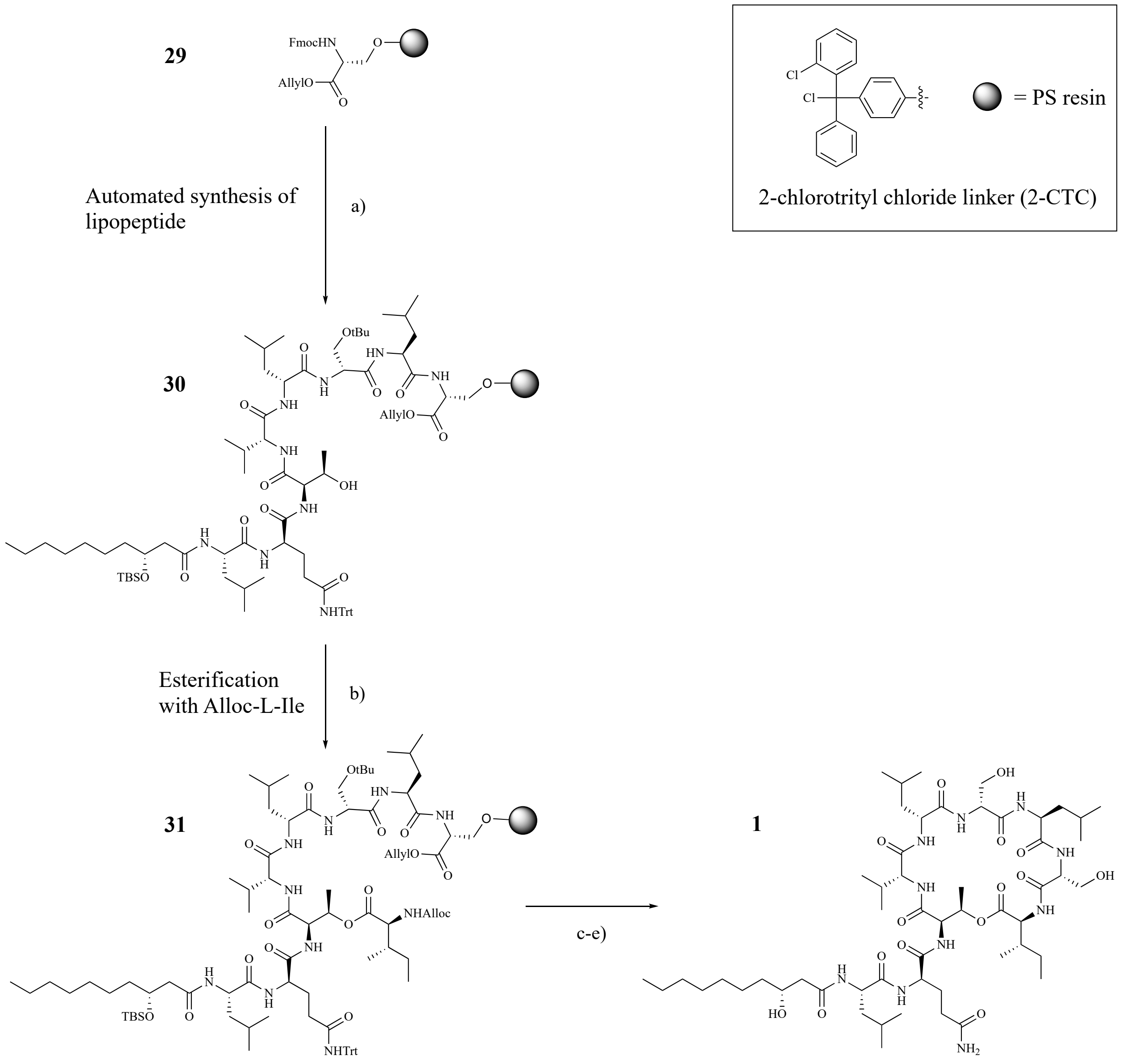

Scheme S1. Synthetic route towards pseudodesmin A (**1**). Reagents and conditions: a) (i) 20% piperidine, NMP; (ii) Fmoc-AA-OH, HBTU, DIPEA, NMP; b) Alloc-L-Ile, DIC, pyridine, DMAP, DMF; c) [$Pd(PPh_3)_4$], $PhSiH_3$, CH_2Cl_2; d) HATU, HOAt, DIPEA, DMF; e) 0.1 M HCl in HFIP + 1% TIS

Side-chain anchoring of Fmoc- D-Ser(OH)-OAllyl (29)

Fmoc- D-Ser(OH)-OAllyl (1.19 g, 3.24 mmol, 1.5 equiv.) was dissolved in dry THF (13.5 ml) in a dry flask under argon atmosphere and pyridine was added (520 µl, 6.48 mmol, 3 equiv.). The 2-CTC resin (1.60 mmol/g; 1.35 g, 2.16 mmol, 1 equiv.) was brought in a dry reaction vessel (100 ml) and then the first reaction mixture was added to the reactor, which was subsequently flushed with argon and sealed. The reactor was placed in a Selecta Vibromatic shaker and connected to a thermostat with a temperature set at 60°C and the reactor was shaken overnight (24h). Next, the excess of reagents was filtered off, the resin was washed with dry DCM and unreacted

functionalities were capped by adding a DCM/MeOH/DIPEA (15 ml; 17/2/1; 2×10 min.) solution. After washings with DCM (3x), DMF (3x) and DCM (3x), the beads were dried on the oil pump overnight prior to loading determination. The loading was determined by monitoring the dibenzofulvene-piperidine adduct formed after Fmoc-deprotection (De Vleeschouwer et al., 2014) and a loading of 0.51 mmol/g was obtained.

Automated synthesis of peptide 30

For the synthesis, we started from 150 mg of preloaded resin **29** (150 mg, 0.0765 mmol). The attachment of the next 7 amino acid residues and the protected lipid tail happened in an automated and iterative fashion in which each cycle consists of two steps: removal of the Fmoc group and coupling of the next building block. The preloaded resin (0.51 mmol/g, 0.150 g, 0.0765 mmol) was swollen in NMP prior to coupling. In this way, following building blocks are coupled respectively: Fmoc-L-Leu-OH, Fmoc-D-Ser(OtBu)-OH, Fmoc-D-Leu-OH, Fmoc-D-Val-OH, Fmoc-D-*allo*-Thr(OH)-OH, Fmoc-D-Gln(Trt)-OH, Fmoc-L-Leu-OH and the lipid tail.

Control of the reaction was performed by a small scale cleavage of the peptidyl resin (1 mg) and the obtained peptide was subjected to LC-MS analysis.

LC-MS analysis:

Exact mass calcd. for $C_{51}H_{91}N_9O_{15}$ = 1069.66

LC-MS: (0-100%B in 6 minutes on Kinetex C18 column)

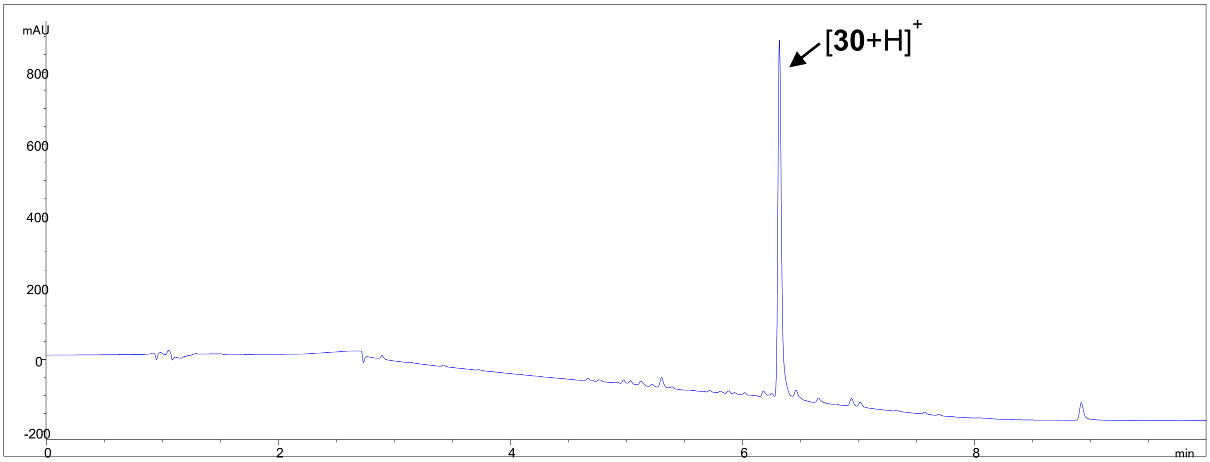

Figure S1. Chromatogram of crude **30**. Kinetex C18 column with elution by a linear gradient over 6 min of 5 mM NH_4OAc in H_2O and CH_3CN from 100:0 to 0:100. Detection at a wavelength of λ=214 nm.

Retention time (min.)	Mass	Interpretation
6.3	1070.6	$[M+H]^+$

Formation of ester bond (31)

For the formation of the ester bond, we found two optimized procedures resulting in a maximal conversion with a minimal amount of epimerization products. In the first case the reaction was carried out at room temperature and required a reaction time of 2×24h. In the second case, reaction took place at 37°C and only required a reaction time of 1×24h to obtain similar conversion. However, this step required an additional transportation step of the peptidyl resin.

Conditions at room temperature

The peptidyl resin was first dried overnight and then swollen in dry DMF under argon atmosphere. In a dry flask, Alloc-L-Ile-OH (0.38 mmol, 5 equiv.) and DIC (0.38 mmol, 5 equiv.) were dissolved in dry DMF at 0°C and this mixture was preactivated for 20 minutes at the same temperature. Thereafter, the reaction mixture was transferred to the peptide reactor and both pyridine (0.38 mmol, 5 equiv.) and DMAP (0.019 mmol, 0.25 equiv.) dissolved in dry DMF, were added. The reaction vessel was shaken for 24 h. After filtration of the reagents, the coupling step was repeated one additional time. Subsequently, the resin was washed with DMF (3x), MeOH (3x) and DCM (3x). A small scale cleavage and LC-MS analysis thereof, were used to monitor the progress of the reaction.

Conditions at 37°C

The peptidyl resin was first dried for 2 hours on the oil pump before it was transported to an eppendorf tube (1.5 ml) and stored under argon atmosphere. In a dry flask, Alloc-L-Ile-OH (10 equiv.) was dissolved in dry THF at 0°C and DIC (10 equiv.) was added and, next, this mixture was stirred for 20 minutes at the same temperature. Thereafter, the reaction mixture was added, together with DMAP (1 equiv.) dissolved in dry THF, to the eppendorf tube containing the peptidyl resin. The eppendorf was placed in the thermoshaker and was shaken for 24h at 37°C. Then, the beads were transferred again to the plastic reaction vessel and, subsequently, the resin was washed with THF (3x), DMF (3x) and DCM (3x). A small scale cleavage and LC-MS analysis confirmed ester bond formation.

LC-MS analysis:

Exact mass calcd. for $C_{61}H_{106}N_{10}O_{18}$ = 1266.77

LC-MS: (0-100%B in 6 minutes on Kinetex C18 column)

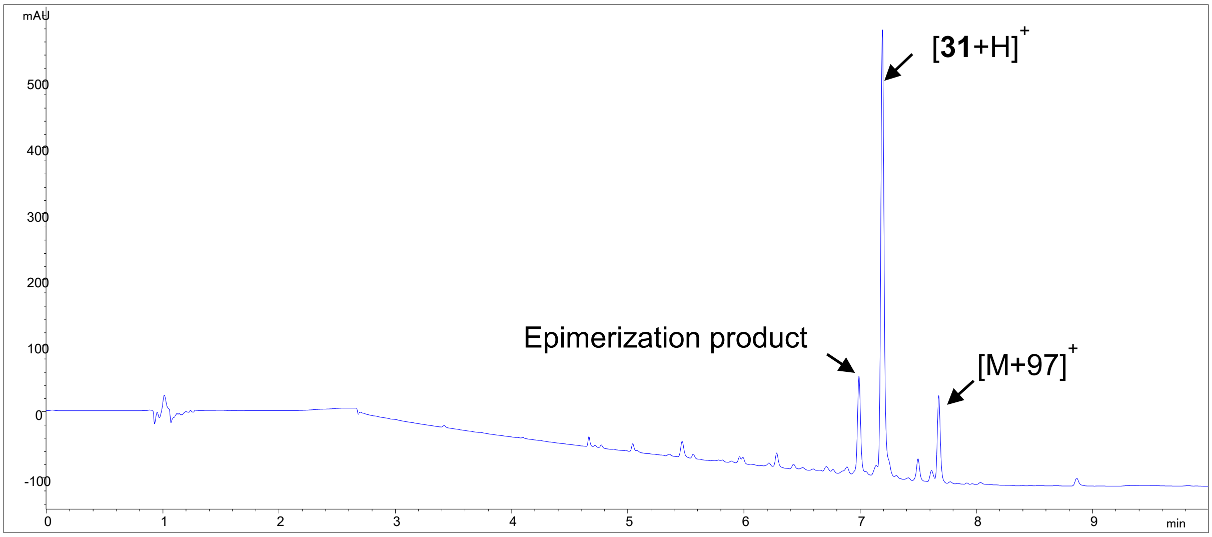

Figure S2. Chromatogram of crude **31**. Kinetex C18 column with elution by a linear gradient over 6 min of 5 mM NH_4OAc in H_2O and CH_3CN from 100:0 to 0:100. Detection at a wavelength of λ=214 nm.

Retention time (min.)	Mass	Interpretation
7.0	1267.7	$[M+H]^+$, epimer
7.2	1267.7	$[M+H]^+$, product
7.5	1363.7	$[M+97]^+$, TFA acylated

Alloc/Allyl deprotection and on-resin cyclization (1)

The beads were dried on the oil pump overnight prior to removal of the Alloc and Allyl protecting groups. The peptidyl resin was swollen under argon atmosphere with DCM. A solution of phenylsilane (4.59 mmol, 60 equiv.) and a catalytic amount of $Pd(PPh_3)_4$ (0.019 mmol, 0.25 equiv.) in DCM (3 ml) was added. The reaction vessel was flushed with argon, shielded from light and shaken for 1h. After filtration, the deprotection step was repeated once more. Subsequently the resin was washed with DCM (3x), MeOH (3x) and DCM (3x) and dried for 2 hours. Next, the resin was swollen with dry DMF and kept under argon atmosphere. A solution of DIPEA (5 equiv.), HATU (5 equiv.) and HOAt (5 equiv.) was added and the reaction vessel was agitated for 4 h at room temperature. After reaction, the resin was washed with DMF (3x), MeOH (3x), and DCM (3x). Small scale cleavage and LC-MS analysis of **1** confirmed successful cyclization.

<u>LC-MS analysis:</u>

Exact mass for $C_{54}H_{96}N_{10}O_{15}$ = 1124.71

LC-MS: (0-100%B in 6 minutes on Kinetex C18 column)

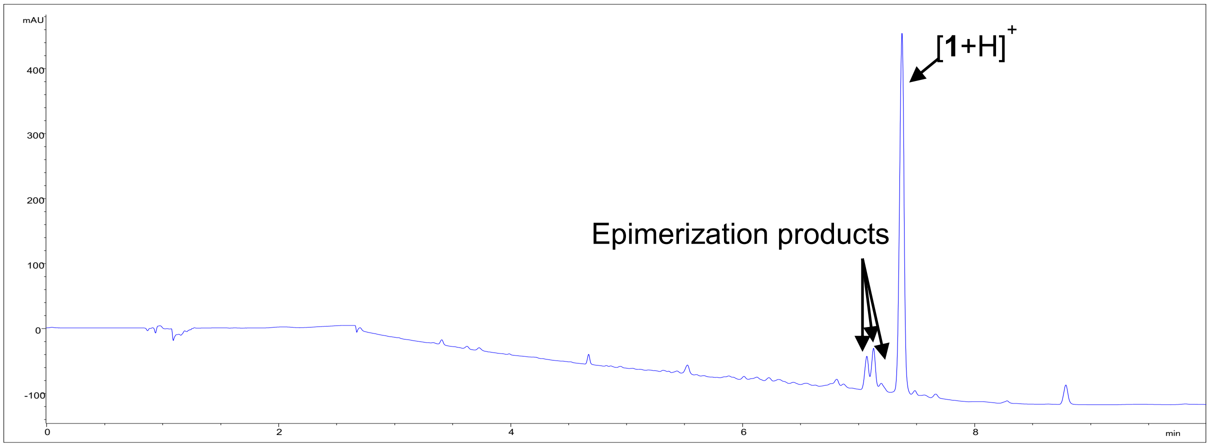

Figure S3. Chromatogram of crude pseudodesmin A (**1**) after cleavage conditions with 0.1M HCl in HFIP (to avoid the formation of TFA-esters). Kinetex C18 column with elution by a linear gradient over 6 min of 5 mM NH_4OAc in H_2O and CH_3CN from 100:0 to 0:100.

Total deprotection and purification

The peptidyl resin (150 mg, 0.0765 mmol) was brought in a plastic reactor vessel equipped with a Teflon frits. A solution of 0.1M HCl in HFIP (equivalent to 1 ml of *ca*. 37% aq. HCl per 99 ml HFIP) was used to which 1% TIS was added to this mixture as scavenger. This solution was added to the resin (10 ml/g resin) and reacted for half an hour. Then, the mixture was filtered and the resin was washed two times with the mixture. All filtrates were collected in a falcon tube. The cleavage reaction and washing steps were repeated two additional times. After a total time of 5h, the combined filtrates were dried to obtain the crude peptide. Thereafter, cold MTBE ether was added to the crude peptide mixture and centrifuged for 5 minutes, after which the solution containing the protecting groups was decanted, leaving the crude peptide **1** as a white precipitate. This work up with MTBE was repeated two more times.

The peptide was then purified by RP-HPLC equipped with a Luna C18(2) column (C18, 250 × 21.2 mm, 5 µm particle size) using elution by a linear gradient over 25 min of H_2O containing 0.1% TFA and CH_3CN from 40:60 to 15:85. The combined product containing fractions were lyophilized to obtain 23.5 mg of the pure peptide. (27% overall yield based on the initial resin loading). This compound was further characterized by HRMS and NMR spectroscopy (see below). The purity of compound **1** was analyzed by LC-MS and is 99.66%.

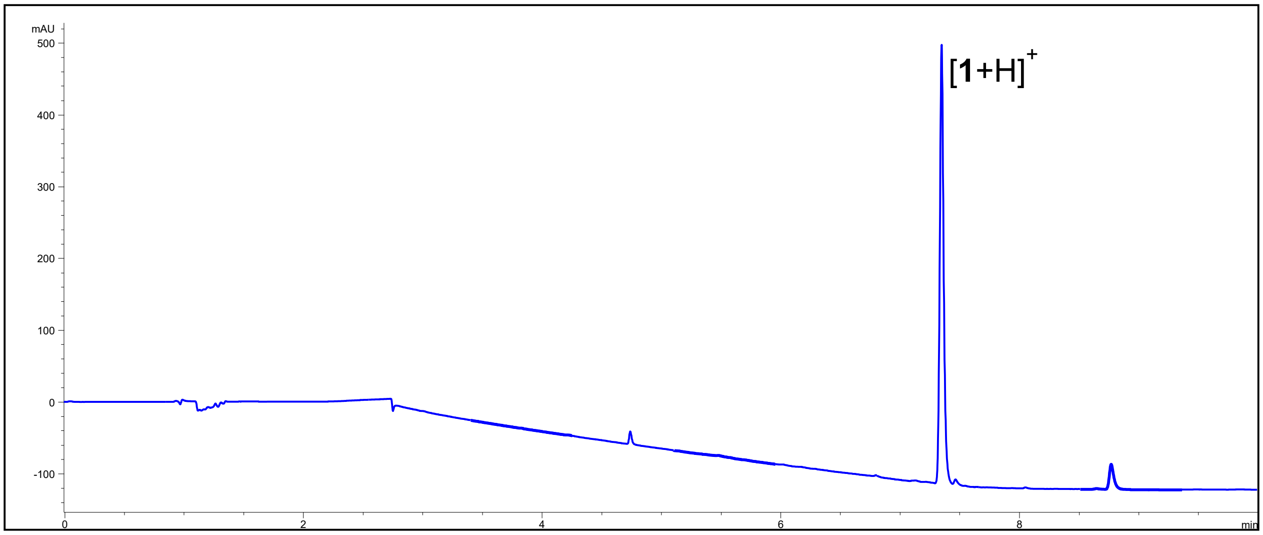

Figure S4. RP-HPLC chromatogram of purified **1**. Kinetex C18 column with elution by a linear gradient over 6 min of 5 mM NH_4OAc in H_2O and CH_3CN from 100:0 to 0:100. Detection at a wavelength of λ=214 nm.

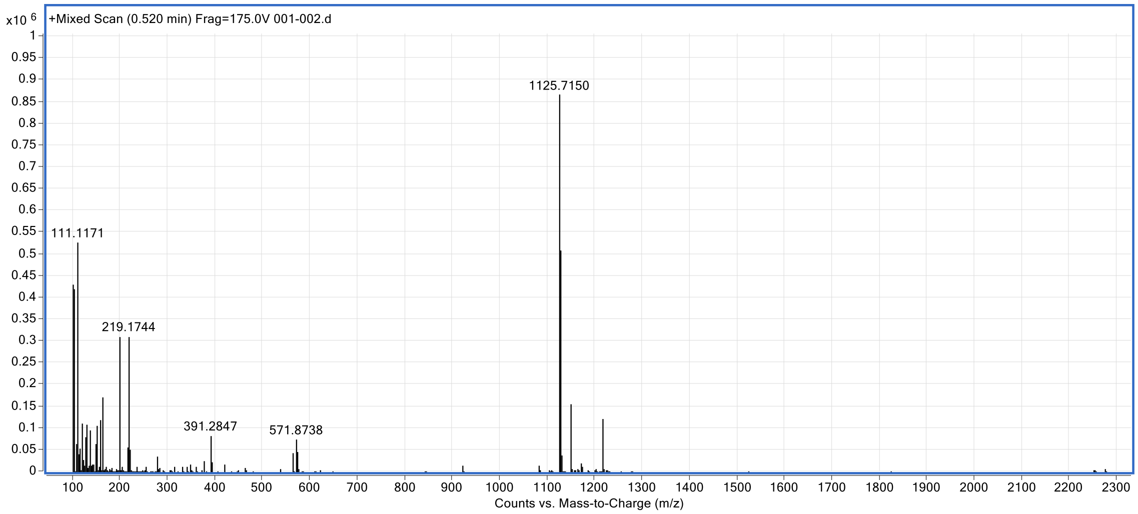

Figure S5. High-resolution full mass spectrum of purified **1**.

Expected exact mass of **1** ($C_{54}H_{96}N_{10}O_{15}$) + H^+: 1125.7129 Da

Observed exact mass of **1** + H^+: 1125.7150 Da

$$\Delta = \frac{1125.7129 - 1125.7150}{1125.7129} \times 10^6 \text{ppm} = 1.8 \text{ ppm}$$

Application of synthesis strategy for the synthesis of CLiP analogues

Enantiomer of pseudodesmin A (2)

The enantiomer of pseudodesmin A (**2**; *ent*-**1**) was synthesized using amino acids and building blocks with inverted stereochemistry compared to pseudodesmin A. All amino acids were commercially available from Iris Biotech GmbH. Fmoc-L-Ser(OH)-OAllyl, Alloc-D-Ile and (*S*)-3-(*tert*-butyldimethylsilyloxy) decanoic acid were prepared in the lab using the same procedures as described for pseudodesmin A.(De Vleeschouwer et al., 2014)

Having all building blocks in hand, an identical procedure as for PsdA (**1**) was followed to obtain *ent*-**1** starting from preloaded resin (0.37 mmol/g; 0.150g, 0.0555 mmol). LC-MS analysis of the lysed final compound confirmed successful synthesis.

LC-MS analysis:

Exact mass for $C_{54}H_{96}N_{10}O_{15}$ = 1124.71

LC-MS: (0-100%B in 6 minutes on Kinetex column)

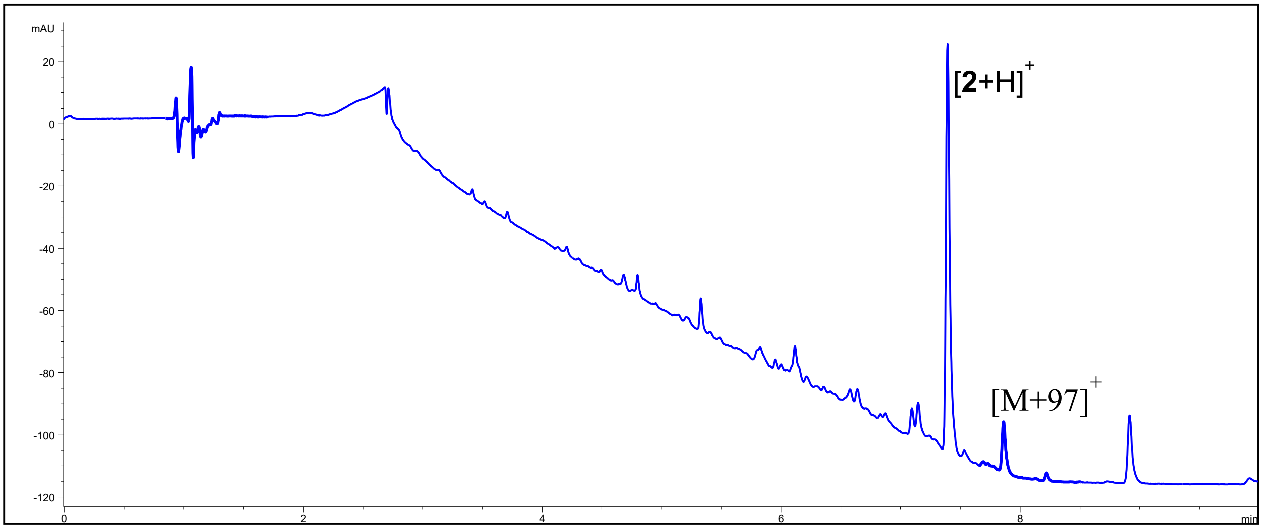

Figure S6. RP-HPLC chromatogram of crude enantiomer of pseudodesmin A (**2**; *ent*-**1**). Kinetex C18 column with elution by a linear gradient over 6 min of 5 mM NH_4OAc in H_2O and CH_3CN from 100:0 to 0:100. Detection at a wavelength of λ=214 nm.

Retention time (min.)	Mass	Interpretation
7.4	1125.6	$[M+H]^+$, product
7.9	1221.6	$[M+97]^+$, TFA acylated

The peptidyl resin (0.0555 mmol) was brought in a plastic reactor vessel equipped with a Teflon frit. 1.5 ml of a mixture of TFA/triisopropylsilane/water (95/2.5/2.5; % v/v/v) was added and the vessel was shaken for 15 minutes. The resin was washed with TFA (1 ml) and the filtrates were

collected. This procedure was repeated two more times (2×15 min.). The combined filtrates were dried to obtain the crude peptide. Thereafter, cold MTBE ether was added and centrifuged for 5 minutes, after which the solution containing the protecting groups was decanted, leaving the crude peptide *ent*-**1** as a white precipitate. This work up with MTBE was repeated two more times.

The peptide was then purified by RP-HPLC equipped with a Kromasil column (C18, 250 × 7.8 mm, 5 μm particle size) using elution by a linear gradient over 24 min of H_2O containing 0.1% HCOOH and CH_3CN from 100:0 to 0:100. The combined product containing fractions were lyophilized to obtain 14.5 mg of the pure peptide. (23% overall yield based on the initial resin loading). This compound was further characterized by HRMS and NMR spectroscopy (see below). The pure compound **2** was analyzed and compared with **1** by LC-MS. As expected, the enantiomeric variant has an identical retention time. The purity is 99.66%.

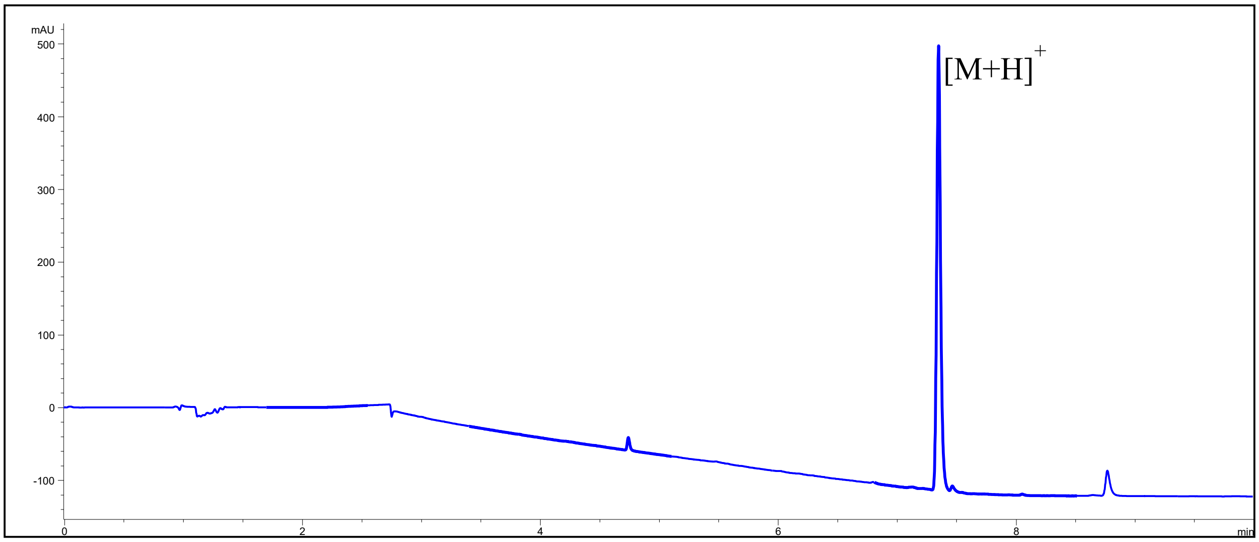

Figure S7. RP-HPLC chromatogram of purified **1**. Kinetex C18 column with elution by a linear gradient over 6 min of 5 mM NH_4OAc in H_2O and CH_3CN from 100:0 to 0:100. Detection at a wavelength of λ=214 nm.

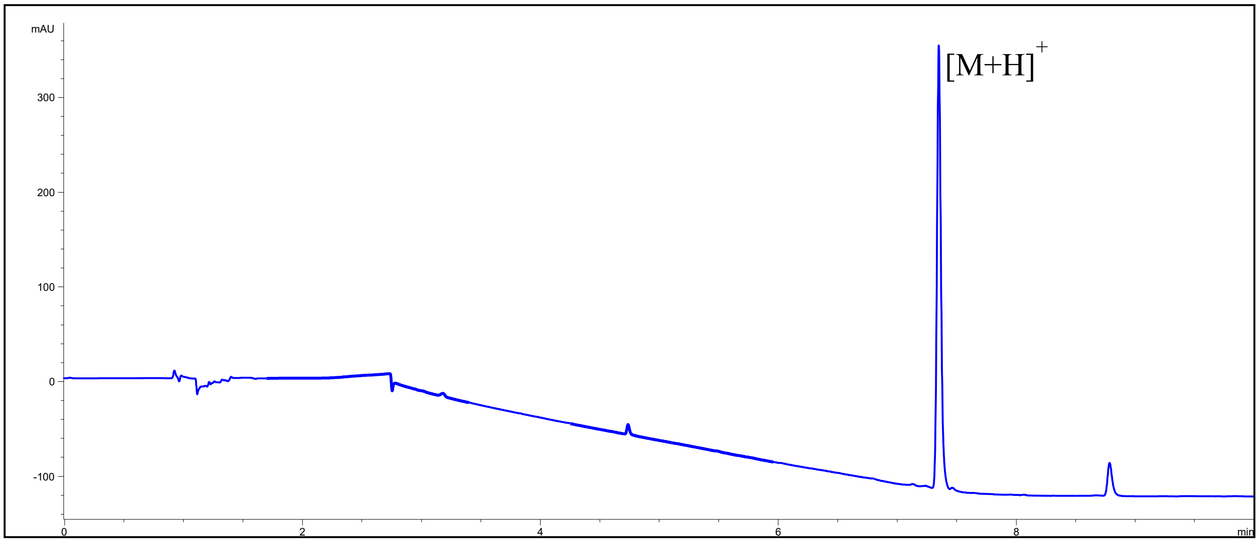

Figure S8. RP-HPLC chromatogram of purified **2** (*ent*-**1**). Kinetex C18 column with elution by a linear gradient over 6 min of 5 mM NH_4OAc in H_2O and CH_3CN from 100:0 to 0:100. Detection at a wavelength of λ=214 nm.

HRMS (ESI+): calculated for **2** (*ent*-**1**) ($C_{54}H_{96}N_{10}O_{15}$) $+H^+$: 1125.71294; found: 1125.7184; Δ = 1.33 ppm

Analogues of the Ala-scan

pseudodesmin L1A (3)

Having all building blocks in hand, an identical procedure was followed to obtain pseudodesmin L1A (**3**) starting from preloaded resin. (0.37 mmol/g; 0.150g, 0.0555 mmol) LC-MS analysis of the cleaved final compound confirmed successful synthesis.

LC-MS analysis:

Exact mass for $C_{51}H_{90}N_{10}O_{15}$ = 1082.66

LC-MS: (0-100%B in 6 minutes on Kinetex column)

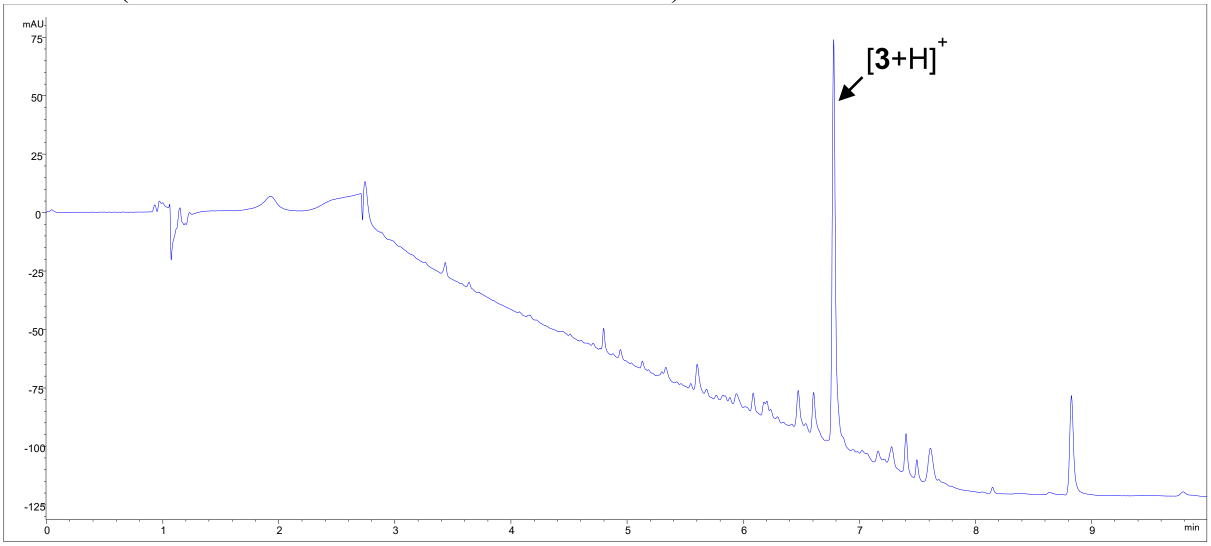

Figure S9. RP-HPLC chromatogram of crude pseudodesmin L1A (**3**). Kinetex C18 column with elution by a linear gradient over 6 min of 5 mM NH_4OAc in H_2O and CH_3CN from 100:0 to 0:100. Detection at a wavelength of λ=214 nm.

Retention time (min)	Mass (Da)	Interpretation
6.8	1083.4	$[M+H]^+$, product
7.4	1179.4	$[M+97]^+$, TFA acylated

The standard procedure for final cleavage with TFA/TIS/H_2O was followed. After workup with MTBE, 57.3 mg of crude pseudodesmin L1A (**3**) was obtained. The peptide was then purified by semi-preparative RP-HPLC using elution by a linear gradient over 26 min of H_2O containing 0.1% HCOOH and CH_3CN from 100:0 to 0:100. The combined product containing fractions were lyophilized to obtain 13.0 mg of the pure peptide which was analyzed again by LC-MS. The overall yield after purification was 22% based on initial resin loading and the purity is 98.63%. This compound was further characterized NMR spectroscopy (see below).

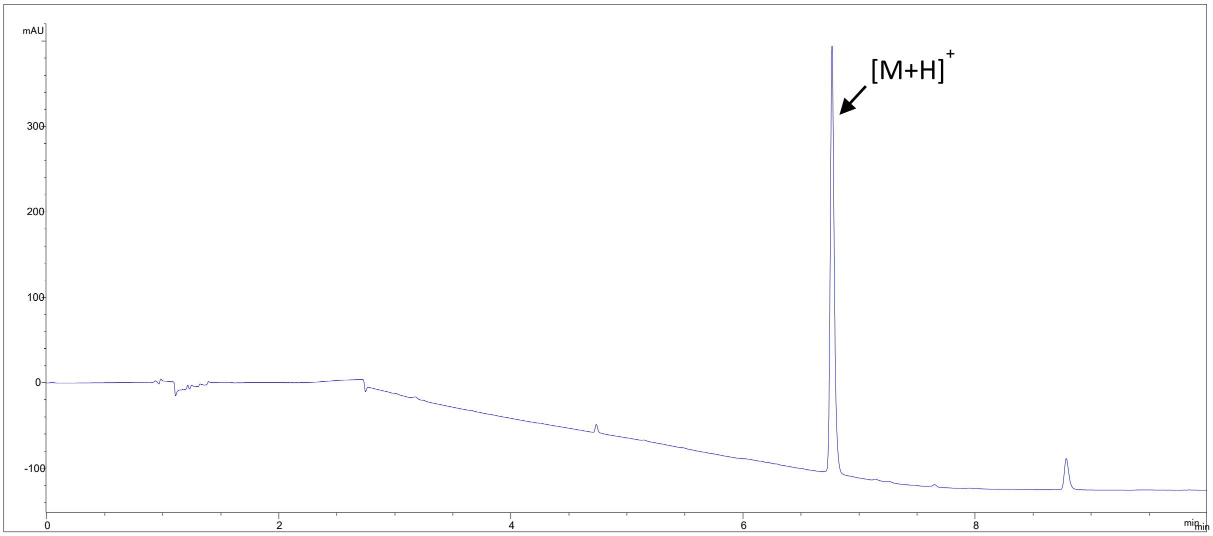

Figure S10. RP-HPLC chromatogram of purified **3**. Kinetex C18 column with elution by a linear gradient over 6 min of 5 mM NH_4OAc in H_2O and CH_3CN from 100:0 to 0:100. Detection at a wavelength of λ=214 nm.

pseudodesmin Q2A (4)

Having all building blocks in hand, an identical procedure was followed to obtain pseudodesmin Q2A (**4**) starting from preloaded resin. (0.37 mmol/g; 0.150g, 0.0555 mmol) LC-MS analysis of the cleaved final compound confirmed successful synthesis.

<u>LC-MS analysis:</u>

Exact mass for $C_{52}H_{93}N_9O_{14}$ = 1067.68

LC-MS: (0-100%B in 6 minutes on Kinetex C18 column)

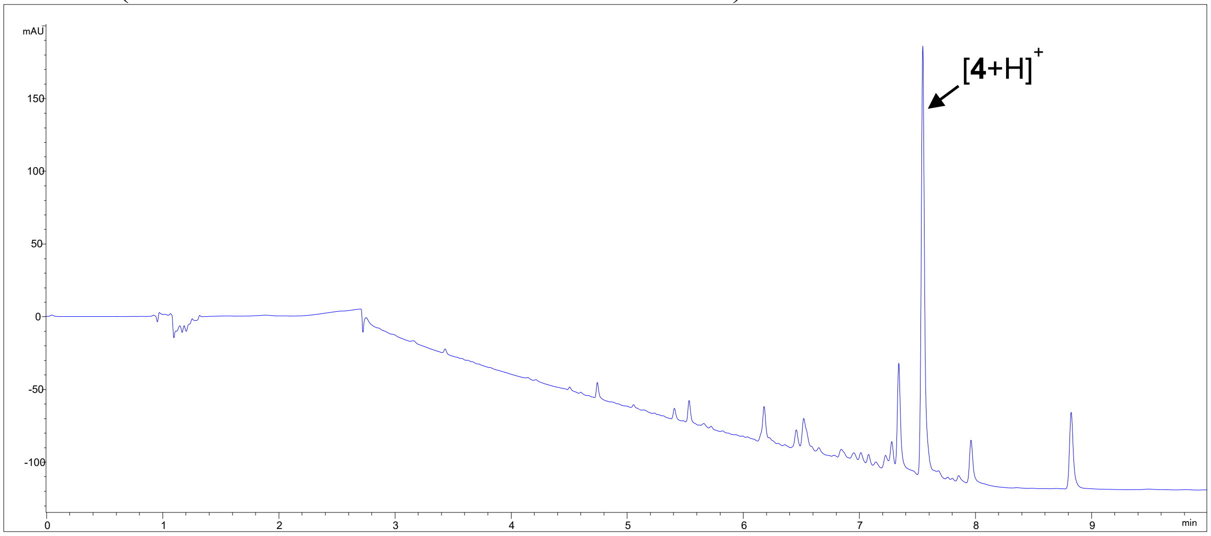

Figure S11. RP-HPLC chromatogram of crude pseudodesmin Q2A (**4**). Kinetex C18 column with elution by a linear gradient over 6 min of 5 mM NH_4OAc in H_2O and CH_3CN from 100:0 to 0:100. Detection at a wavelength of λ=214 nm.

Retention time (min)	Mass (Da)	Interpretation
7.3	1068.4	$[M+H]^+$, epimerization product
7.5	1068.4	$[M+H]^+$, product

The standard procedure for final cleavage with TFA/TIS/H_2O was followed. After workup with MTBE, 42.6 mg of crude pseudodesmin Q2A (**4**) was obtained. The peptide was then purified by semi-preparative RP-HPLC using elution by a linear gradient over 30 min of H_2O containing 0.1% HCOOH and CH_3CN from 100:0 to 0:100. The combined product containing fractions were lyophilized to obtain 7.0 mg of the pure peptide which was analyzed again by LC-MS. The overall yield after purification was 12% based on initial resin loading and the purity is 99.66%. This compound was further characterized by NMR spectroscopy (see below).

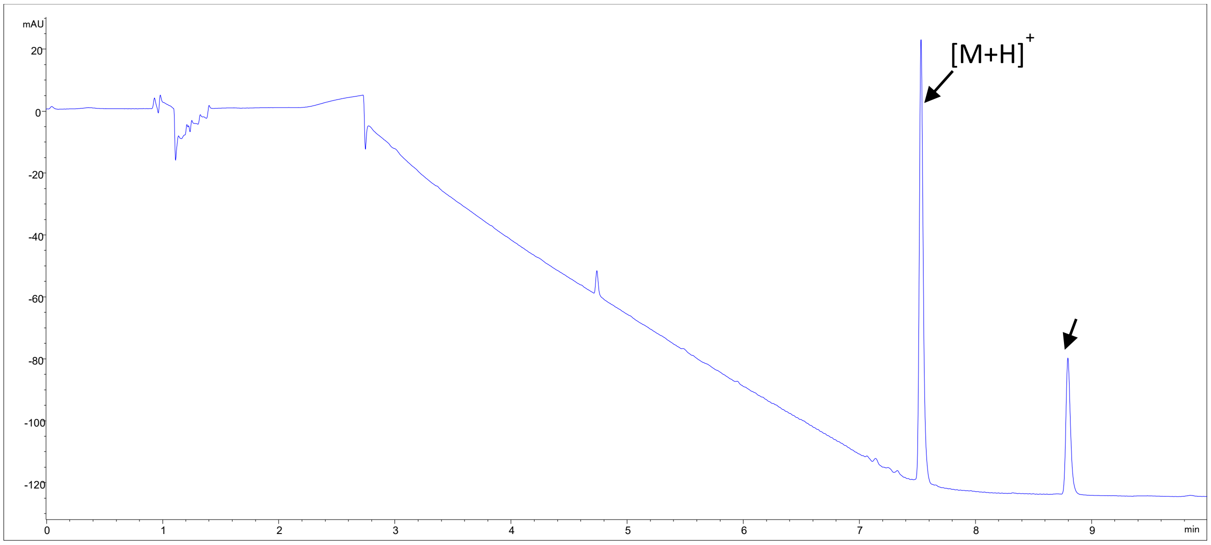

Figure S12. RP-HPLC chromatogram of purified **4**. Kinetex C18 column with elution by a linear gradient over 6 min of 5 mM NH_4OAc in H_2O and CH_3CN from 100:0 to 0:100. Detection at a wavelength of λ=214 nm.

pseudodesmin V4A (5)

Having all building blocks in hand, an identical procedure was followed to obtain pseudodesmin V4A (**5**) starting from preloaded resin. (0.37 mmol/g; 0.150g, 0.0555 mmol) LC-MS analysis of the cleaved final compound confirmed successful synthesis.

<u>LC-MS analysis:</u>

Exact mass for $C_{52}H_{92}N_{10}O_{15}$ = 1096.67

LC-MS: (0-100%B in 6 minutes on Kinetex C18 column)

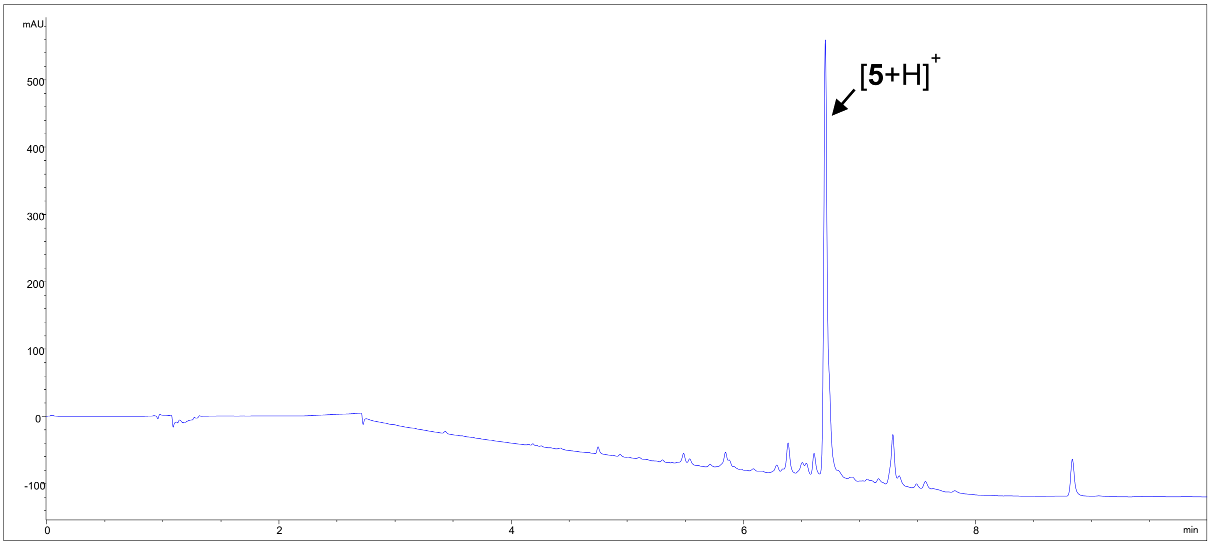

Figure S13. RP-HPLC chromatogram of crude pseudodesmin V4A (**5**). Kinetex C18 column with elution by a linear gradient over 6 min of 5 mM NH_4OAc in H_2O and CH_3CN from 100:0 to 0:100. Detection at a wavelength of λ=214 nm.

Retention time (min)	Mass (Da)	Interpretation
6.7	1097.6	$[M+H]^+$, product
7.3	1193.6	$[M+97]^+$, TFA acylated

The standard procedure for final cleavage with TFA/TIS/H_2O was followed. After workup with MTBE, 54.9 mg of crude pseudodesmin V4A (**5**) was obtained. The peptide was then purified by semi-preparative RP-HPLC using elution by a linear gradient over 26 min of H_2O containing 0.1% HCOOH and CH_3CN from 100:0 to 0:100. The combined product containing fractions were lyophilized to obtain 22.1 mg of the pure peptide which was analyzed again by LC-MS. The overall yield after purification was 36% based on initial resin loading and the purity is 99.71%. This compound was further characterized by NMR spectroscopy (see below).

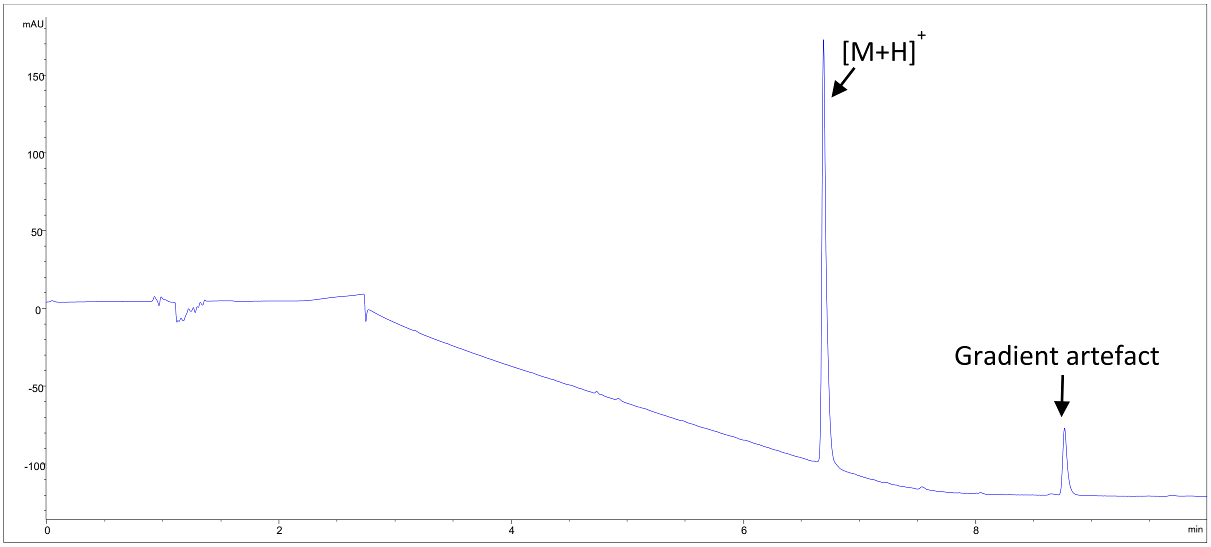

Figure S14. RP-HPLC chromatogram of purified **5**. Kinetex C18 column with elution by a linear gradient over 6 min of 5 mM NH_4OAc in H_2O and CH_3CN from 100:0 to 0:100. Detection at a wavelength of λ=214 nm.

pseudodesmin L5A (6)

Having all building blocks in hand, an identical procedure was followed to obtain pseudodesmin L5A (**6**) starting from preloaded resin (0.37 mmol/g; 0.150g, 0.0555 mmol). LC-MS analysis of the cleaved final compound confirmed successful synthesis.

<u>LC-MS analysis:</u>

Exact mass for $C_{51}H_{90}N_{10}O_{15}$ = 1082.66

LC-MS: (0-100%B in 6 minutes on Kinetex C18 column)

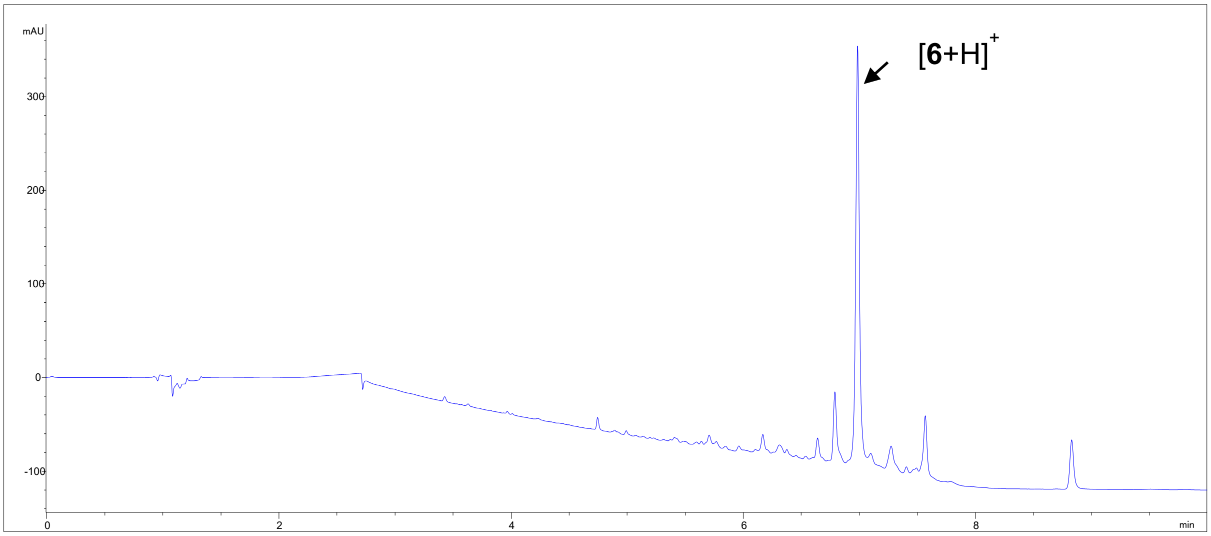

Figure S15. RP-HPLC chromatogram of crude pseudodesmin L5A (**6**). Kinetex C18 column with elution by a linear gradient over 6 min of 5 mM NH_4OAc in H_2O and CH_3CN from 100:0 to 0:100. Detection at a wavelength of λ=214 nm.

Retention time (min)	Mass (Da)	Interpretation
6.8	1083.5	$[M+H]^+$, epimerization product
7.0	1083.5	$[M+H]^+$, product
7.6	1179.4	$[M+97]^+$, TFA acylated

The standard procedure for final cleavage with TFA/TIS/H_2O was followed. After workup with MTBE, 57.7 mg of crude pseudodesmin L5A (**6**) was obtained. The peptide was then purified by semi-preparative RP-HPLC using elution by a linear gradient over 26 min of H_2O containing 0.1% HCOOH and CH_3CN from 100:0 to 0:100. The combined product containing fractions were lyophilized to obtain 15.1 mg of the pure peptide which was analyzed again by LC-MS. The overall yield after purification was 25% based on initial resin loading and the purity is 99.57%. This compound was further characterized using NMR spectroscopy (see below).

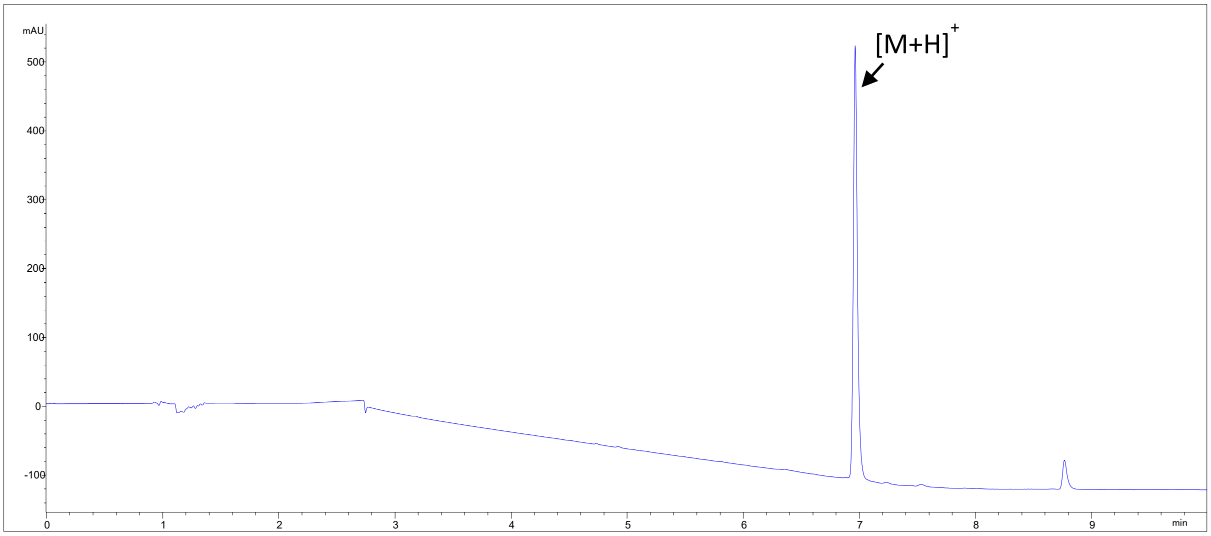

Figure S16. RP-HPLC chromatogram of purified **6**. Kinetex C18 column with elution by a linear gradient over 6 min of 5 mM NH_4OAc in H_2O and CH_3CN from 100:0 to 0:100. Detection at a wavelength of λ=214 nm.

pseudodesmin S6A (7)

Having all building blocks in hand, an identical procedure was followed to obtain pseudodesmin S6A (**7**) starting from preloaded resin (0.37 mmol/g; 0.150g, 0.0555 mmol). LC-MS analysis of the cleaved final compound confirmed successful synthesis.

<u>LC-MS analysis:</u>

Exact mass for $C_{54}H_{96}N_{10}O_{14}$ = 1108.71

LC-MS: (0-100%B in 6 minutes on Kinetex C18 column)

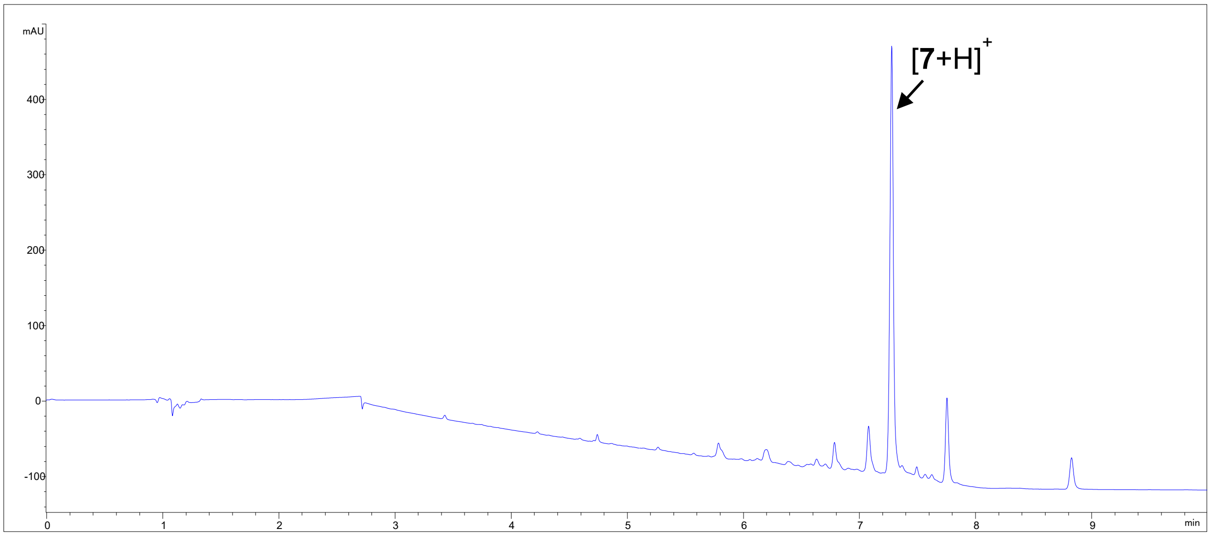

Figure S17. RP-HPLC chromatogram of crude pseudodesmin S6A (**7**). Kinetex C18 column with elution by a linear gradient over 6 min of 5 mM NH_4OAc in H_2O and CH_3CN from 100:0 to 0:100. Detection at a wavelength of λ=214 nm.

Retention time (min.)	Mass	Interpretation
7.1	1109.6	$[M+H]^+$, epimerization product
7.3	1109.6	$[M+H]^+$, product
7.8	1205.6	$[M+97]^+$, TFA acylated

The standard procedure for final cleavage with TFA/TIS/H_2O was followed. After workup with MTBE, 61.9 mg of crude pseudodesmin S6A (**7**) was obtained. The peptide was then purified by semi-preparative RP-HPLC using elution by a linear gradient over 30 min of H_2O containing 0.1% HCOOH and CH_3CN from 100:0 to 0:100. The combined product containing fractions were lyophilized to obtain 15.1 mg of the pure peptide which was analyzed again by LC-MS. The overall yield after purification was 25% based on initial resin loading and the purity is 88.04%. This compound was further characterized by NMR spectroscopy (see below).

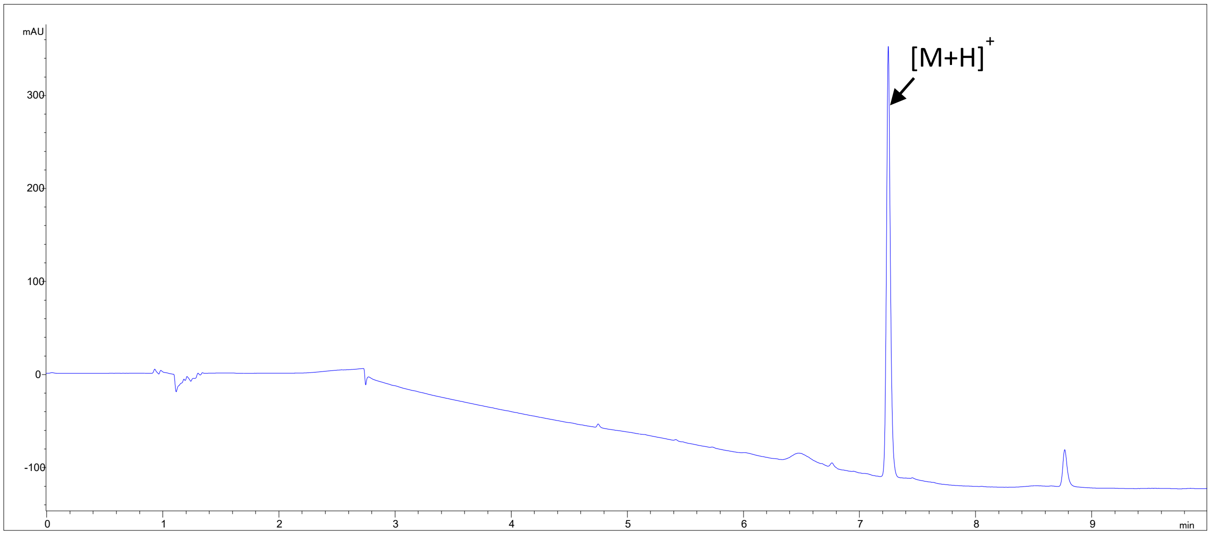

Figure S18. RP-HPLC chromatogram of purified **7**. Kinetex C18 column with elution by a linear gradient over 6 min of 5 mM NH_4OAc in H_2O and CH_3CN from 100:0 to 0:100. Detection at a wavelength of λ=214 nm.

pseudodesmin L7A (8)

Having all building blocks in hand, an identical procedure was followed to obtain pseudodesmin L7A (**8**) starting from preloaded resin (0.37 mmol/g; 0.150g, 0.0555 mmol). LC-MS analysis of the cleaved final compound confirmed successful synthesis.

LC-MS analysis:

Exact mass for $C_{51}H_{90}N_{10}O_{15}$ = 1082.66

LC-MS: (0-100%B in 6 minutes on Kinetex C18 column)

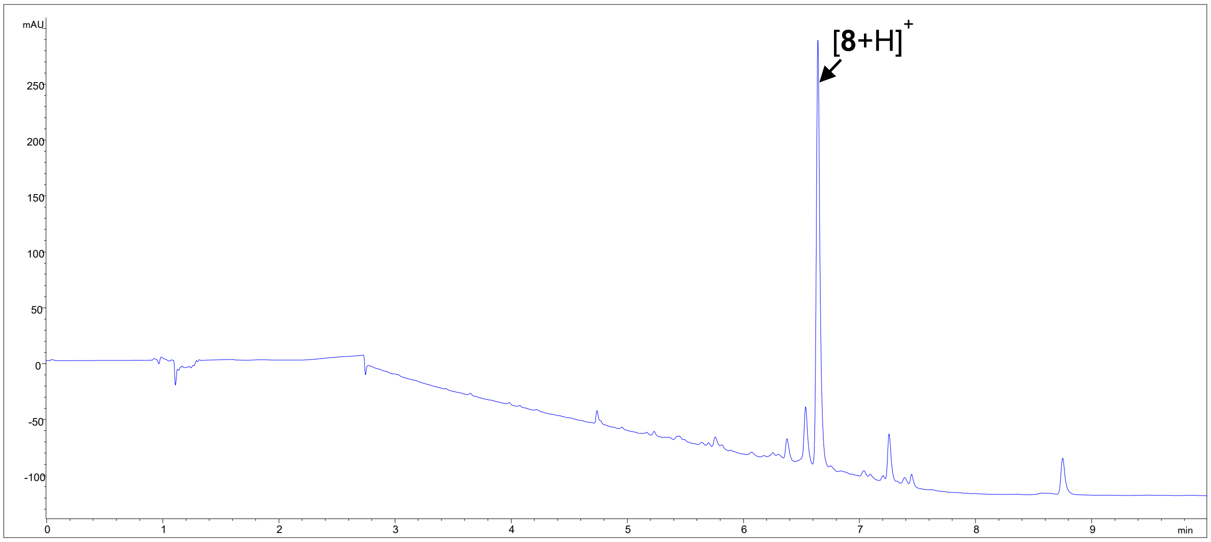

Figure S19. RP-HPLC chromatogram of crude pseudodesmin L7A (**8**). Kinetex C18 column with elution by a linear gradient over 6 min of 5 mM NH_4OAc in H_2O and CH_3CN from 100:0 to 0:100. Detection at a wavelength of λ=214 nm.

Retention time (min)	Mass (Da)	Interpretation
6.5	1083.4	$[M+H]^+$, Epimerization product
6.6	1083.4	$[M+H]^+$, product
7.3	1179.4	$[M+97]^+$, TFA acylated

The standard procedure for final cleavage with TFA/TIS/H_2O was followed. After workup with MTBE, 61.9 mg of crude pseudodesmin L7A (**8**) was obtained. The peptide was then purified by semi-preparative RP-HPLC using elution by a linear gradient over 26 min of H_2O containing 0.1% HCOOH and CH_3CN from 100:0 to 0:100. The combined product containing fractions were lyophilized to obtain 19.8 mg of the pure peptide which was analyzed again by LC-MS. The overall yield after purification was 33% based on initial resin loading and the purity is 94.22%. This compound was further characterized by NMR spectroscopy (see below).

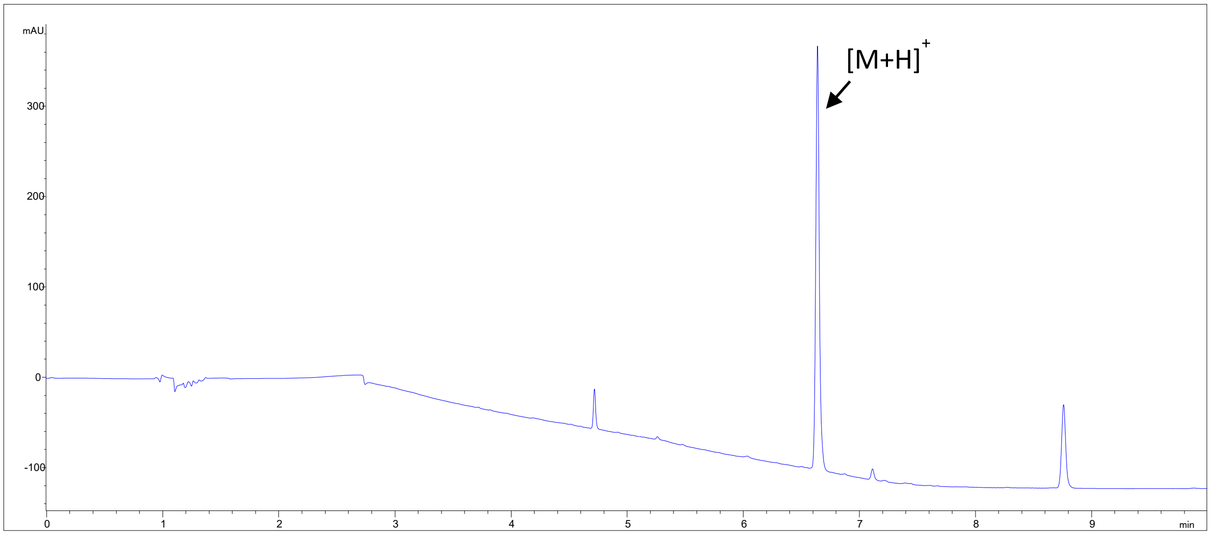

Figure S20. RP-HPLC chromatogram of purified **8**. Kinetex C18 column with elution by a linear gradient over 6 min of 5 mM NH_4OAc in H_2O and CH_3CN from 100:0 to 0:100. Detection at a wavelength of λ=214 nm**.**

pseudodesmin S8A (9)

This analogue required an adapted synthesis route as Ser8 that is normally used for anchoring to the solid support is substituted by alanine. The modified synthesis strategy is described below. For the synthesis of this synthesis of pseudodesmin S8A (**9**), an adapted synthesis route was required based on side-chain attachment of the Ser6 residue. We were able to use the same preloaded resin **29** (0.46 mmol/g; 0.150g, 0.069 mmol). Moreover, the applied synthesis protocols for the synthesis of the linear lipopeptide, esterification, Alloc-deprotection and on-resin cyclization step were identical as before. In between the esterification and Alloc-deprotection steps, two amino acids Ala8 and Leu7 were introduced manually, using the standard procedure for manual couplings. LC-MS analysis of the cleaved final compound confirmed successful synthesis of pseudodesmin S8A **(9).**

LC-MS analysis:

Exact mass for $C_{54}H_{96}N_{10}O_{14}$ = 1108.71

LC-MS: (0-100%B in 6 minutes on Kinetex column)

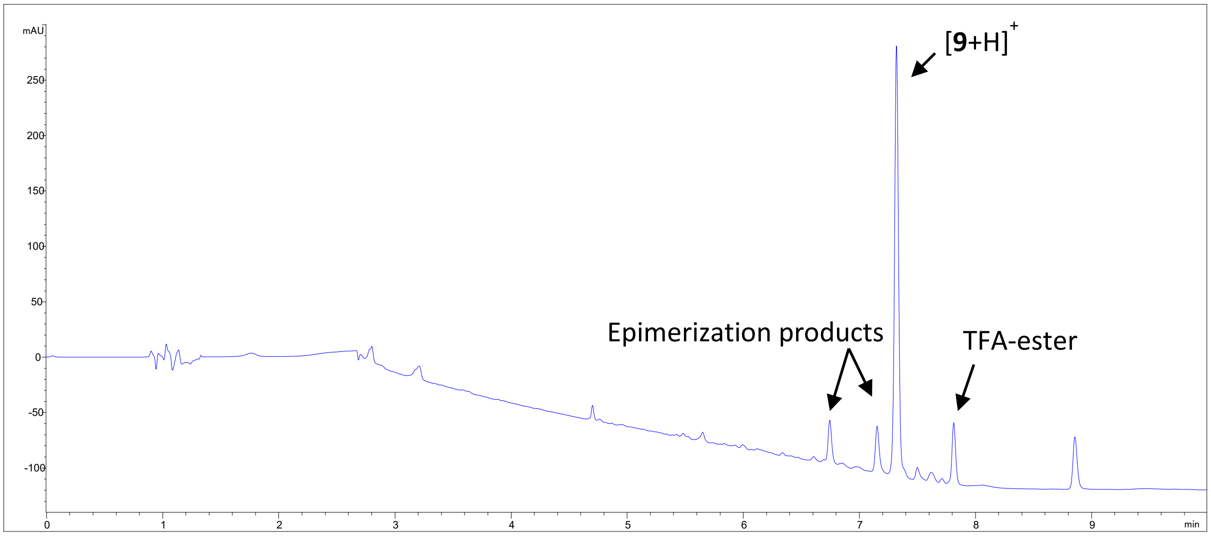

Figure S21. RP-HPLC chromatogram of crude pseudodesmin S8A (**9**). Kinetex C18 column with elution by a linear gradient over 6 min of 5 mM NH4OAc in H2O and CH3CN from 100:0 to 0:100. Detection at a wavelength of λ=214 nm**.**

Retention time (min)	Mass (Da)	Interpretation
7.3	1109.6	$[M+H]^+$, product

The standard procedure for final cleavage with 0.1M HCl in HFIP was followed. After workup with MTBE, 80.5 mg of crude pseudodesmin S8A (**9**) was obtained. The peptide was then purified by semi-preparative RP-HPLC using elution by a linear gradient over 25 min of H_2O containing 0.1% TFA and CH_3CN from 40:60 to 15:85 The combined product containing fractions were lyophilized to obtain 27.0 mg of the pure peptide which was analyzed again by LC-MS. The overall

yield after purification was 35% based on initial resin loading and the purity is 96.64%. This compound was further characterized by HRMS and NMR spectroscopy (see below).

HRMS (ESI+): calculated for pseudodesmin S8A ($C_{54}H_{96}N_{10}O_{14}$) $+H^+$: 1109.71802; found: 1109.7153; Δ = 2.5 ppm

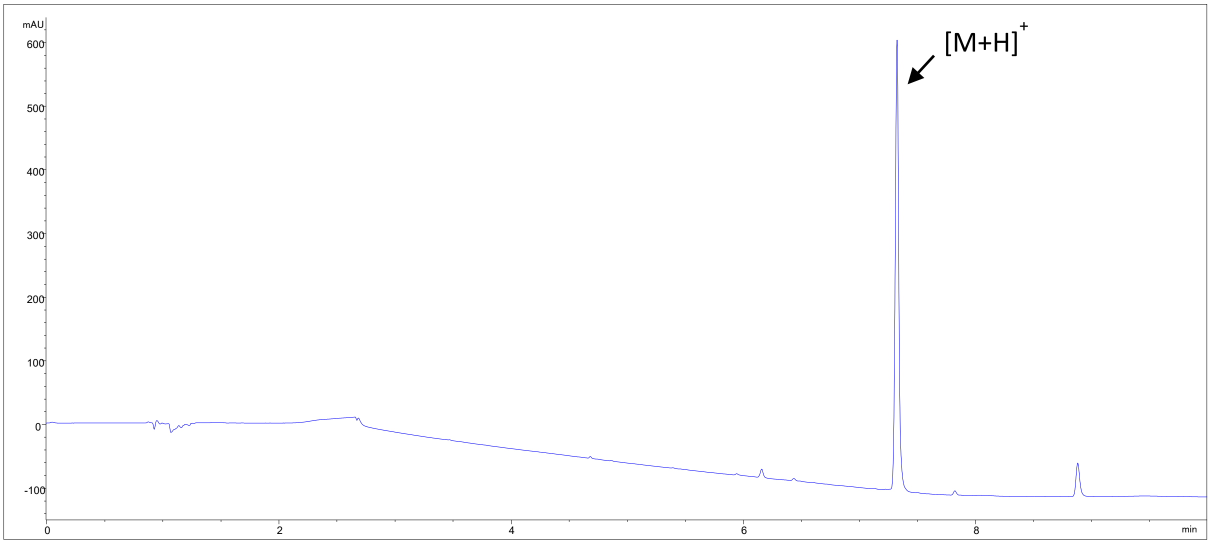

Figure S22. RP-HPLC chromatogram of purified **9**. Kinetex C18 column with elution by a linear gradient over 6 min of 5 mM NH_4OAc in H_2O and CH_3CN from 100:0 to 0:100. Detection at a wavelength of λ=214 nm.

pseudodesmin I9A (10)

Having all building blocks in hand, an identical procedure was followed to obtain pseudodesmin I9A (**10**) starting from preloaded resin (0.37 mmol/g; 0.150g, 0.0555 mmol). LC-MS analysis of the cleaved final compound confirmed successful synthesis.

<u>LC-MS analysis:</u>

Exact mass for $C_{51}H_{90}N_{10}O_{15}$ = 1082.66

LC-MS: (0-100%B in 6 minutes on Kinetex C18 column)

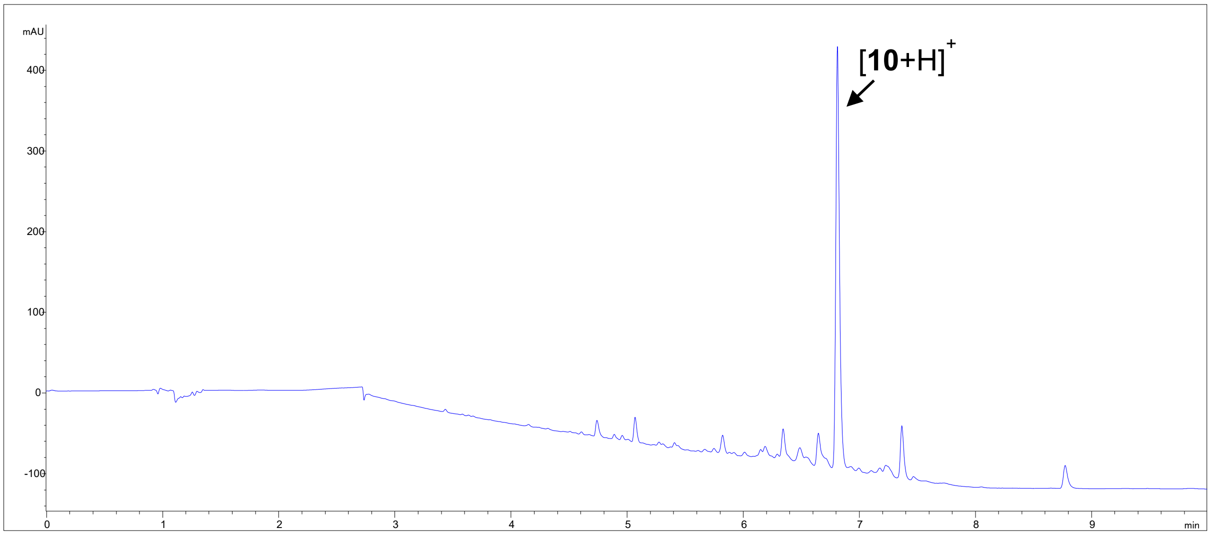

Figure S23. RP-HPLC chromatogram of crude pseudodesmin I9A (**10**). Kinetex C18 column with elution by a linear gradient over 6 min of 5 mM NH_4OAc in H_2O and CH_3CN from 100:0 to 0:100. Detection at a wavelength of λ=214 nm.

Retention time (min)	Mass (Da)	Interpretation
6.8	1083.5	$[M+H]^+$, product
7.4	1179.5	$[M+97]^+$, TFA acylated

The standard procedure for final cleavage with TFA/TIS/H_2O was followed. After workup with MTBE, 56.3 mg of crude pseudodesmin I9A (**10**) was obtained. The peptide was then purified by semi-preparative RP-HPLC using elution by a linear gradient over 26 min of H_2O containing 0.1% HCOOH and CH_3CN from 100:0 to 0:100. The combined product containing fractions were lyophilized to obtain 17.1 mg of the pure peptide which was analyzed again by LC-MS. The overall yield after purification was 28% based on initial resin loading and the purity is 75.28%. This compound was further characterized by NMR spectroscopy (see below).

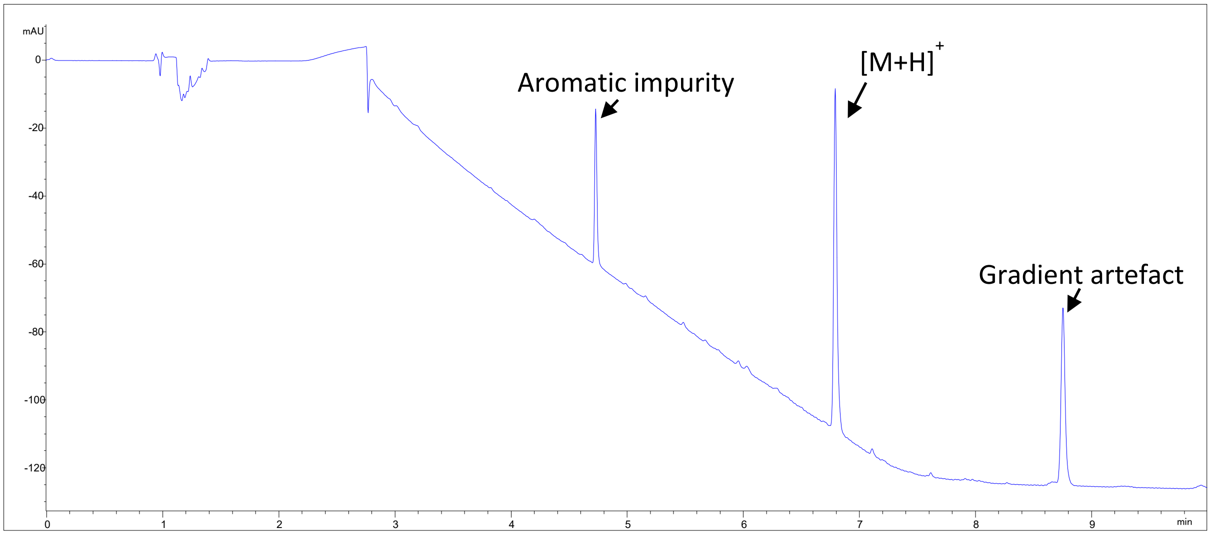

Figure S24. RP-HPLC chromatogram of purified **10**. Kinetex C18 column with elution by a linear gradient over 6 min of 5 mM NH_4OAc in H_2O and CH_3CN from 100:0 to 0:100. Detection at a wavelength of λ=214 nm.

WLIP (11)

For the synthesis of WLIP (**11**) the same strategy is used as for the synthesis of pseudodesmin A. The only difference is that the esterification step proceeded extremely slow and therefore required several repetitions. All other steps were identical and the synthesis started from preloaded resin **29** (0.37 mmol/g; 0.150g, 0.0555 mmol). LC-MS analysis of the cleaved final compound confirmed successful synthesis of WLIP albeit with a significantly decreased crude purity. As described elsewhere, we also developed customized synthesis strategy with optimized protecting group choice.(De Vleeschouwer et al., 2016)

<u>LC-MS analysis:</u>

Exact mass for $C_{54}H_{95}N_9O_{16}$ = 1125.69

LC-MS: (0-100%B in 6 minutes on Kinetex column)

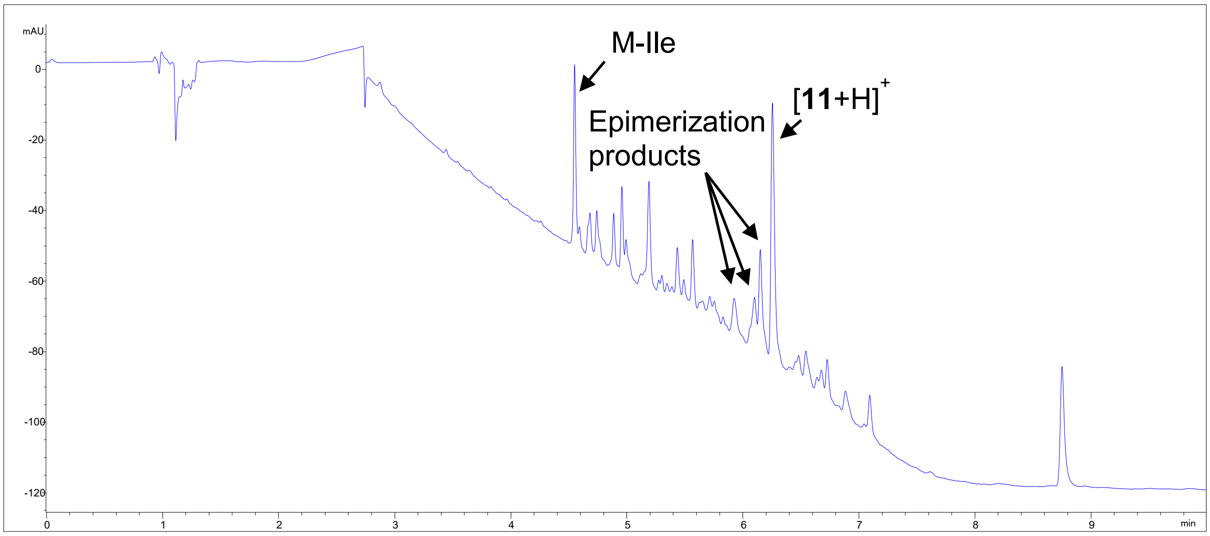

Figure S23. Chromatogram of crude WLIP (**11**)**.** Kinetex C18 column with elution by a linear gradient over 6 min of 5 mM NH_4OAc in H_2O and CH_3CN from 100:0 to 0:100.

Retention time (min)	Mass (Da)	Interpretation
4.6	1031.4	M-Ile
4.9	1144.5	Non-cyclized product
5.0	1144.5	Non-cyclized product
5.9	1126.5	Epimerization product
6.0	1126.5	Epimerization product
6.1	1126.5	Epimerization product
6.3	1126.5	$[M+H]^+$, product

The standard procedure for final cleavage with TFA/TIS/H_2O was followed. After workup with MTBE, 64.2 mg of crude WLIP (**11**) was obtained. The peptide was then purified by semi-preparative RP-HPLC using elution by a linear gradient over 36 min of H_2O containing 0.1% HCOOH and CH_3CN from 100:0 to 0:100. The combined product containing fractions were lyophilized to obtain 6.0 mg of the pure peptide which was analyzed again by LC-MS. The overall yield after purification was 10% based on initial resin loading and the purity is 64.47%. This compound was further characterized by NMR spectroscopy (see below).

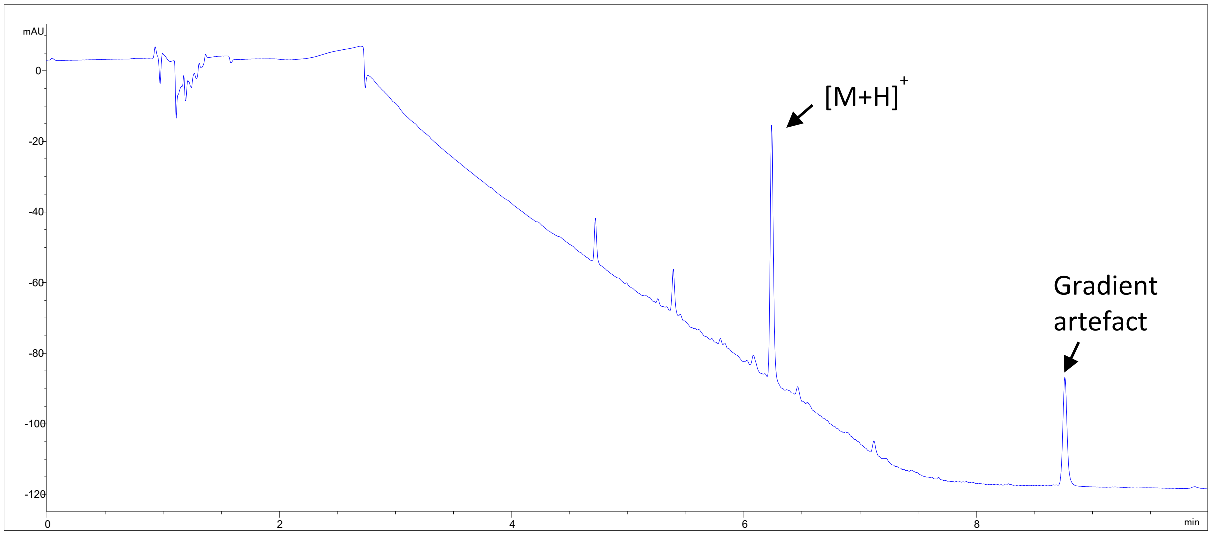

Figure S24. RP-HPLC chromatogram of purified **11**. Kinetex C18 column with elution by a linear gradient over 6 min of 5 mM NH_4OAc in H_2O and CH_3CN from 100:0 to 0:100. Detection at a wavelength of λ=214 nm.

pseudodesmin V4A L5A L7A I9A (12)

Having all building blocks in hand, an identical procedure was followed to obtain pseudodesmin V4A L5A L7A I9A (**12**) starting from preloaded resin (0.46 mmol/g; 0.150g, 0.069 mmol). LC-MS analysis of the cleaved final compound confirmed successful synthesis.

LC-MS analysis:

Exact mass for $C_{43}H_{74}N_{10}O_{15}$ = 970.53

LC-MS: (0-100%B in 6 minutes on Kinetex C18 column)

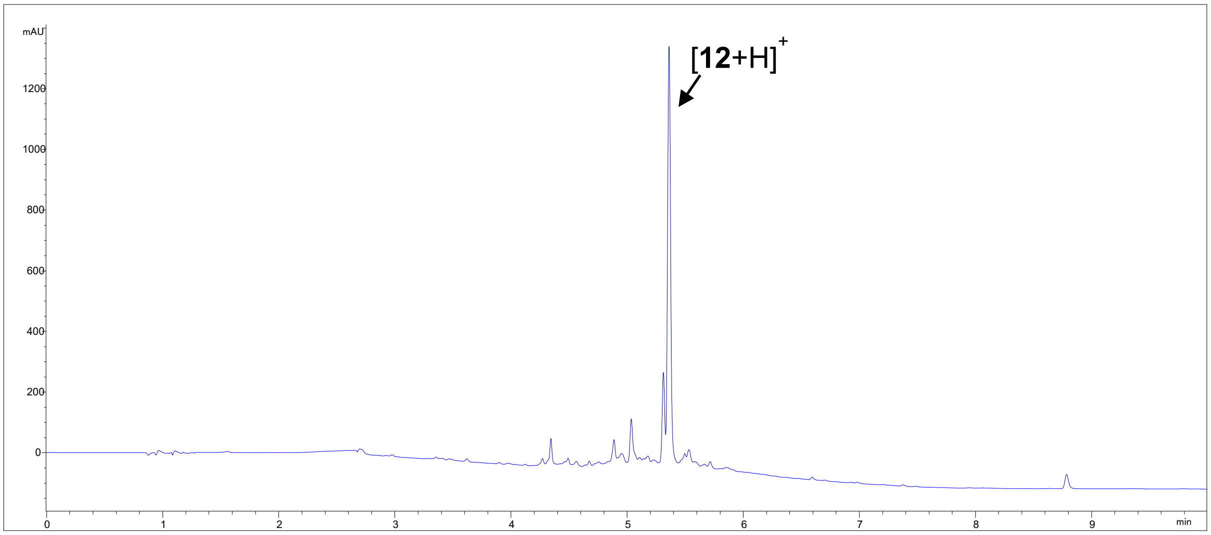

Figure S27. RP-HPLC chromatogram of crude pseudodesmin V4A L5A L7A I9A (**12**). Kinetex C18 column with elution by a linear gradient over 6 min of 5 mM NH_4OAc in H_2O and CH_3CN from 100:0 to 0:100. Detection at a wavelength of λ=214 nm.

Retention time (min)	Mass (Da)	Interpretation
5.3	971.4	$[M+H]^+$, epimerization product
5.4	971.4	$[M+H]^+$, product

The standard procedure for final cleavage with 0.1M HCl in HFIP was followed. After workup with MTBE, 67.7 mg of crude pseudodesmin V4A L5A L7A I9A (**12**) was obtained. The peptide was then purified by preparative RP-HPLC using elution by a linear gradient over 25 min of H_2O containing 0.1% TFA and CH_3CN from 70:30 to 45:55. The combined product containing fractions were lyophilized to obtain 19.1 mg of the pure peptide which was analyzed again by LC-MS. The overall yield after purification was 29% based on initial resin loading and the purity is 85.19%. This compound was further characterized by HRMS and NMR spectroscopy (see below).

HRMS (ESI+): calculated for pseudodesmin V4A L5A L7A I9A ($C_{43}H_{74}N_{10}O_{15}$) $+H^+$: 971.54079; found: 971.5386; Δ = 2.3 ppm

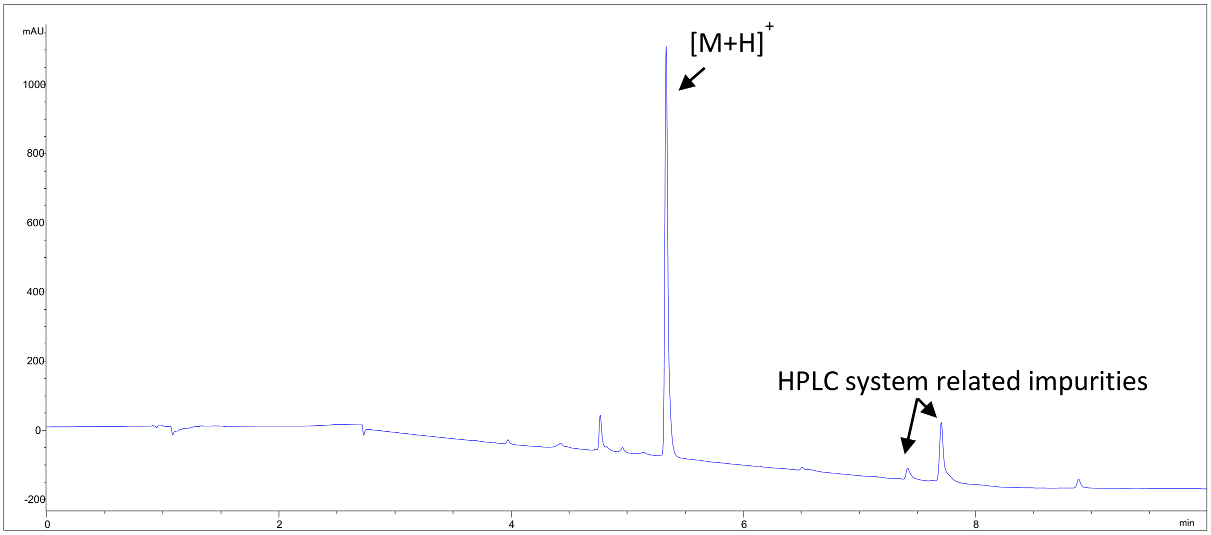

Figure S28. RP-HPLC chromatogram of purified **12**. Kinetex C18 column with elution by a linear gradient over 6 min of 5 mM NH_4OAc in H_2O and CH_3CN from 100:0 to 0:100. Detection at a wavelength of λ=214 nm.

Analogues with modified lipid tails

pseudodesmin C4 (13)

Having all building blocks in hand, an identical procedure was followed to obtain pseudodesmin C4 (**13**) starting from preloaded resin (0.41 mmol/g; 0.150g, 0.0615 mmol). LC-MS analysis of the cleaved final compound confirmed successful synthesis.

LC-MS analysis:

Exact mass for $C_{48}H_{84}N_{10}O_{15}$ = 1040.61

LC-MS: (0-100%B in 6 minutes on Kinetex C18 column)

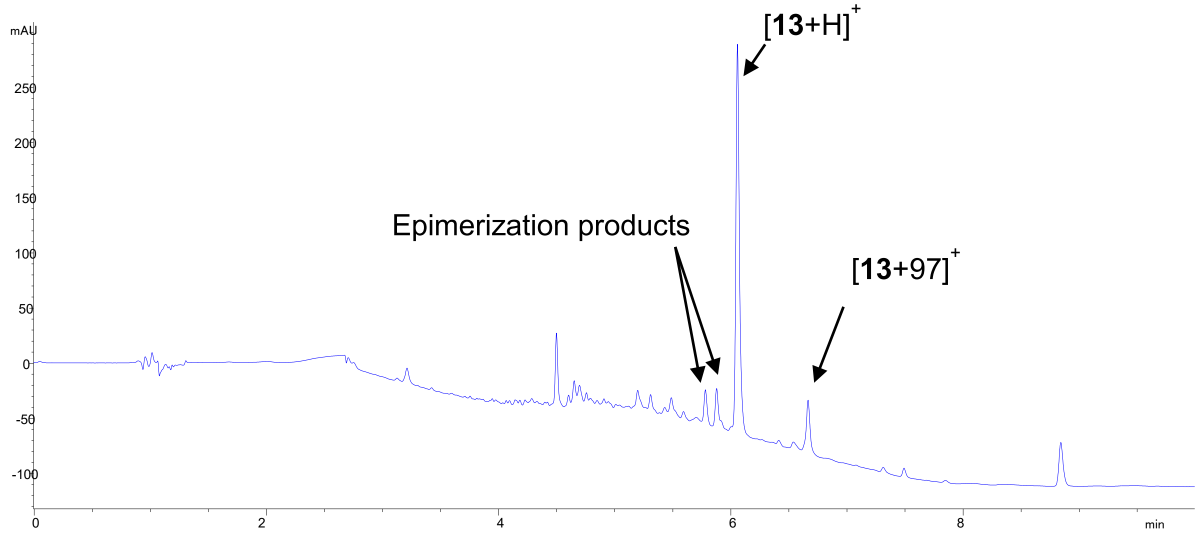

Figure S29. RP-HPLC chromatogram of crude pseudodesmin C4 (**13**). Kinetex C18 column with elution by a linear gradient over 6 min of 5 mM NH_4OAc in H_2O and CH_3CN from 100:0 to 0:100. Detection at a wavelength of λ=214 nm.

Retention time (min)	Mass (Da)	Interpretation
5.8	1041.5	$[M+H]^+$, epimerization product
5.9	1041.5	$[M+H]^+$, epimerization product
6.1	1041.5	$[M+H]^+$, product
6.7	1137.6	$[M+97]^+$, TFA acylated

The standard procedure for final cleavage with 0.1M HCl in HFIP was followed. The peptide was then purified by preparative RP-HPLC using elution by a linear gradient over 25 min of H_2O containing 0.1% TFA and CH_3CN from 60:40 to 35:65. The combined product containing fractions were lyophilized to obtain 5.2 mg of the pure pseudodesmin C4 (**13**) which was analyzed again by LC-MS. The overall yield after purification was 8% based on initial resin loading and the purity is 90.99%. This compound was further characterized by HRMS and NMR spectroscopy (see below).

HRMS (ESI+): calculated for pseudodesmin C4 ($C_{48}H_{84}N_{10}O_{15}$) $+H^+$: 1041.6190; found: 1041.6211; Δ = 2.0 ppm

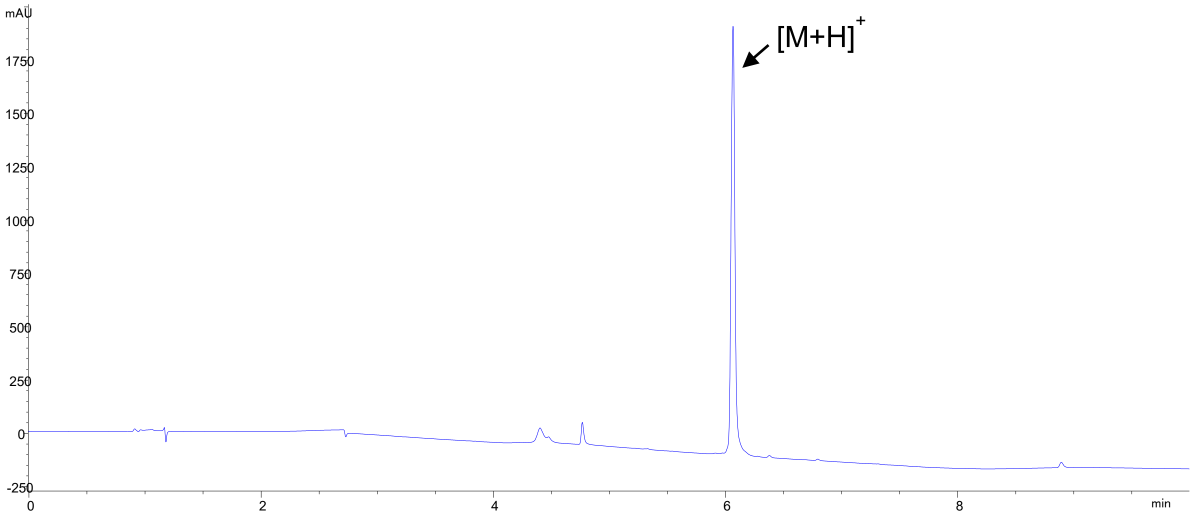

Figure S30. RP-HPLC chromatogram of purified **13**. Kinetex C18 column with elution by a linear gradient over 6 min of 5 mM NH_4OAc in H_2O and CH_3CN from 100:0 to 0:100. Detection at a wavelength of λ=214 nm.

pseudodesmin C6 (14)

Having all building blocks in hand, an identical procedure was followed to obtain pseudodesmin C6 (**14**) starting from preloaded resin (0.41 mmol/g; 0.150g, 0.0615 mmol). LC-MS analysis of the cleaved final compound confirmed successful synthesis.

LC-MS analysis:

Exact mass for $C_{50}H_{88}N_{10}O_{15}$ = 1068.64

LC-MS: (0-100%B in 6 minutes on Kinetex C18 column)

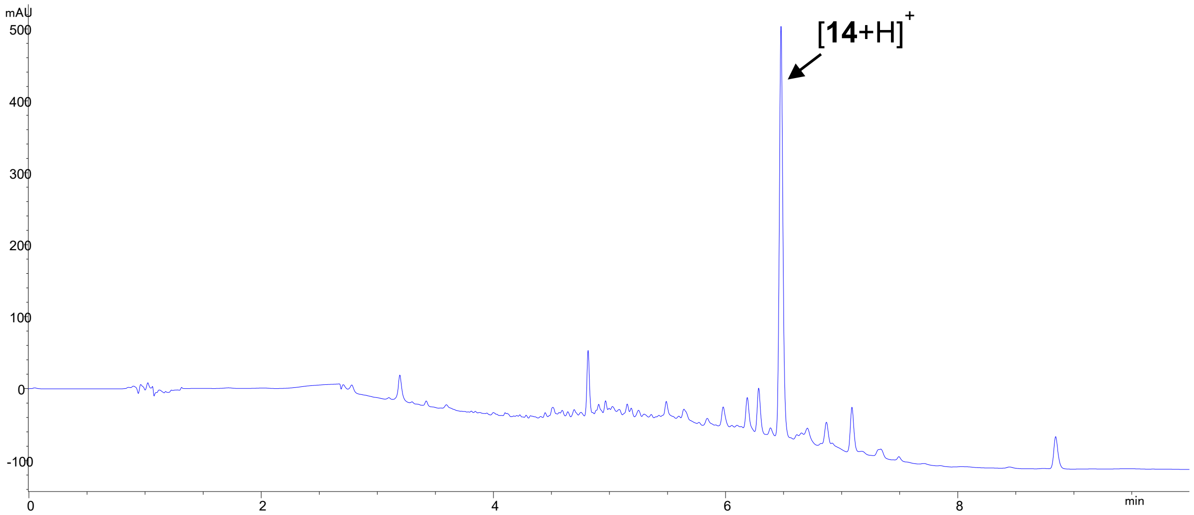

Figure S31. RP-HPLC chromatogram of crude pseudodesmin C6 (**14**). Kinetex C18 column with elution by a linear gradient over 6 min of 5 mM NH_4OAc in H_2O and CH_3CN from 100:0 to 0:100. Detection at a wavelength of λ=214 nm.

Retention time (min)	Mass (Da)	Interpretation
6.0	1069.5	$[M+H]^+$, epimerization product
6.2	1069.5	$[M+H]^+$, epimerization product
6.3	1069.5	$[M+H]^+$, epimerization product
6.5	1069.5	$[M+H]^+$, product
6.7	1165.5	$[M+97]^+$, TFA acylated

The standard procedure for final cleavage with 0.1M HCl in HFIP was followed. The peptide was then purified by preparative RP-HPLC using elution by a linear gradient over 25 min of H_2O containing 0.1% TFA and CH_3CN from 50:50 to 25:75. The combined product containing fractions were lyophilized to obtain 8.8 mg of the pure pseudodesmin C6 (**14**) which was analyzed again by LC-MS. The overall yield after purification was 13% based on initial resin loading and the purity

is 91.31%. This compound was further characterized by HRMS and NMR spectroscopy (see below).

HRMS (ESI+): calculated for pseudodesmin C6 ($C_{50}H_{88}N_{10}O_{15}$) $+H^+$: 1069.6503; found: 1069.6493; $\Delta = 0.9$ ppm

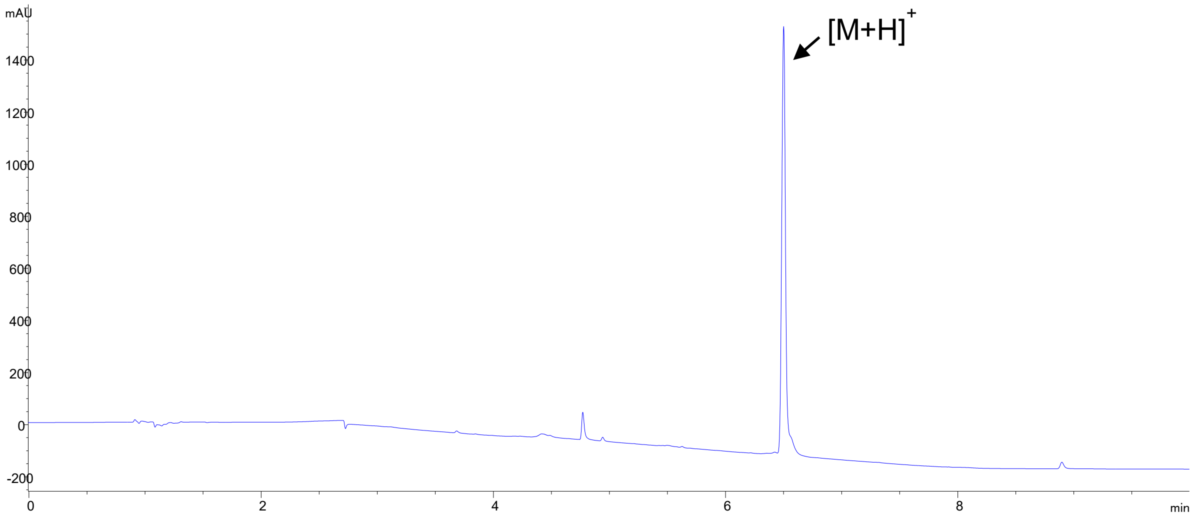

Figure S32. RP-HPLC chromatogram of purified **14**. Kinetex C18 column with elution by a linear gradient over 6 min of 5 mM NH_4OAc in H_2O and CH_3CN from 100:0 to 0:100. Detection at a wavelength of λ=214 nm.

pseudodesmin C8 (15)

Having all building blocks in hand, an identical procedure was followed to obtain pseudodesmin C8 (**15**) starting from preloaded resin (0.41 mmol/g; 0.150g, 0.0615 mmol). LC-MS analysis of the cleaved final compound confirmed successful synthesis.

<u>LC-MS analysis:</u>

Exact mass for $C_{52}H_{92}N_{10}O_{15}$ = 1096.67

LC-MS: (0-100%B in 6 minutes on Kinetex C18 column)

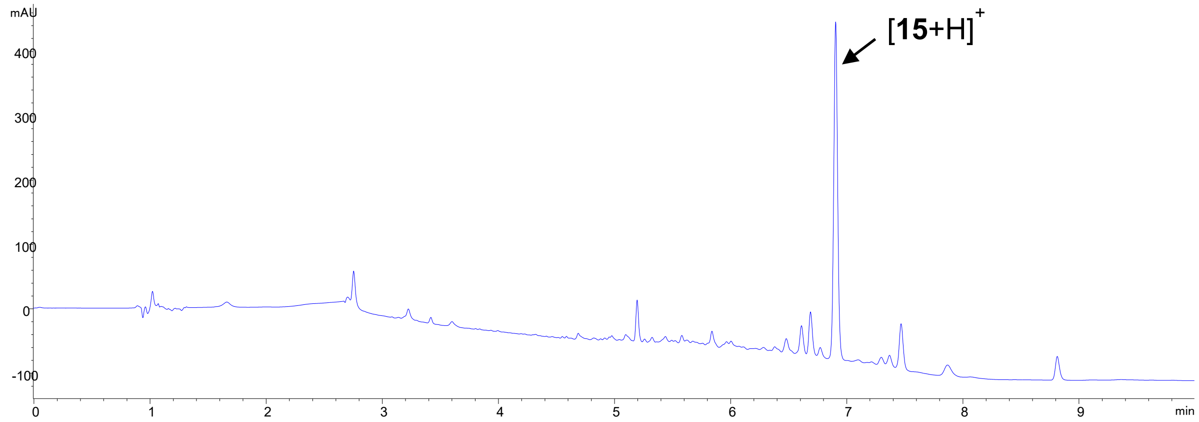

Figure S33. RP-HPLC chromatogram of crude pseudodesmin C8 (**15**). Kinetex C18 column with elution by a linear gradient over 6 min of 5 mM NH_4OAc in H_2O and CH_3CN from 100:0 to 0:100. Detection at a wavelength of λ=214 nm.

Retention time (min)	Mass (Da)	Interpretation
6.6	1097.6	$[M+H]^+$, epimerization product
6.7	1097.6	$[M+H]^+$, epimerization product
6.9	1097.6	$[M+H]^+$, product
7.5	1193.6	$[M+97]^+$, TFA acylated

The standard procedure for final cleavage with 0.1M HCl in HFIP was followed. The peptide was then purified by preparative RP-HPLC using elution by a linear gradient over 25 min of H_2O containing 0.1% TFA and CH_3CN from 45:55 to 20:80. The combined product containing fractions were lyophilized to obtain 6.7 mg of the pure pseudodesmin C8 (**15**) which was analyzed again by LC-MS. The overall yield after purification was 10% based on initial resin loading and the purity is 92.62%. This compound was further characterized by HRMS and NMR spectroscopy (see below).

HRMS (ESI+): calculated for pseudodesmin C8 ($C_{52}H_{92}N_{10}O_{15}$) $+H^{+}$: 1097.6816; found: 1069.6769; Δ = 4.3 ppm

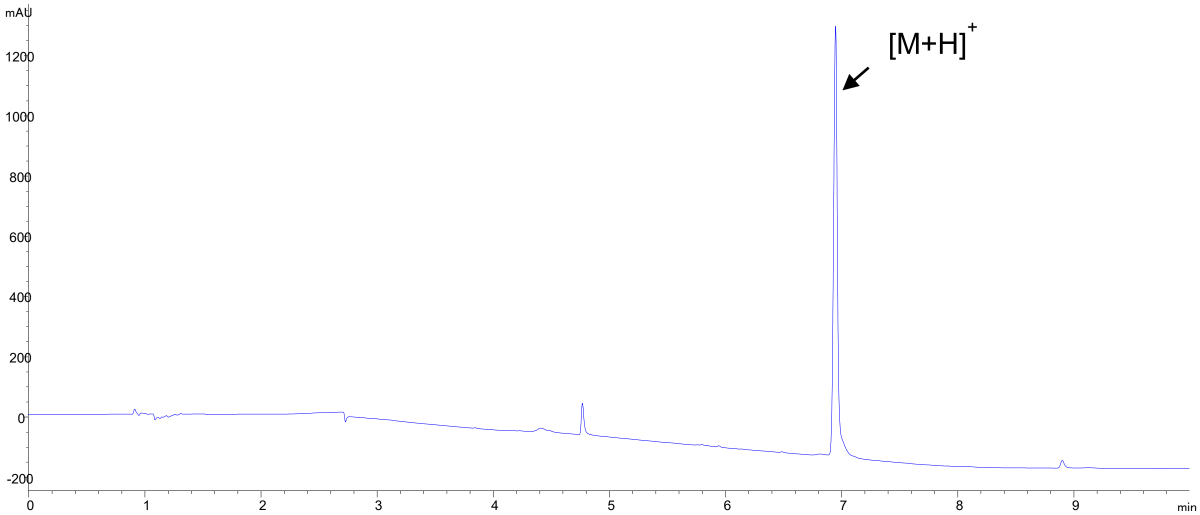

Figure S34. RP-HPLC chromatogram of purified **15**. Kinetex C18 column with elution by a linear gradient over 6 min of 5 mM NH_4OAc in H_2O and CH_3CN from 100:0 to 0:100. Detection at a wavelength of λ=214 nm.

pseudodesmin C12 (16)

Having all building blocks in hand, an identical procedure was followed to obtain pseudodesmin C12 (**16**) starting from preloaded resin (0.41 mmol/g; 0.150g, 0.0615 mmol). LC-MS analysis of the cleaved final compound confirmed successful synthesis.

LC-MS analysis:

Exact mass for $C_{56}H_{100}N_{10}O_{15}$ = 1152.74

LC-MS: (0-100%B in 6 minutes on Kinetex C18 column)

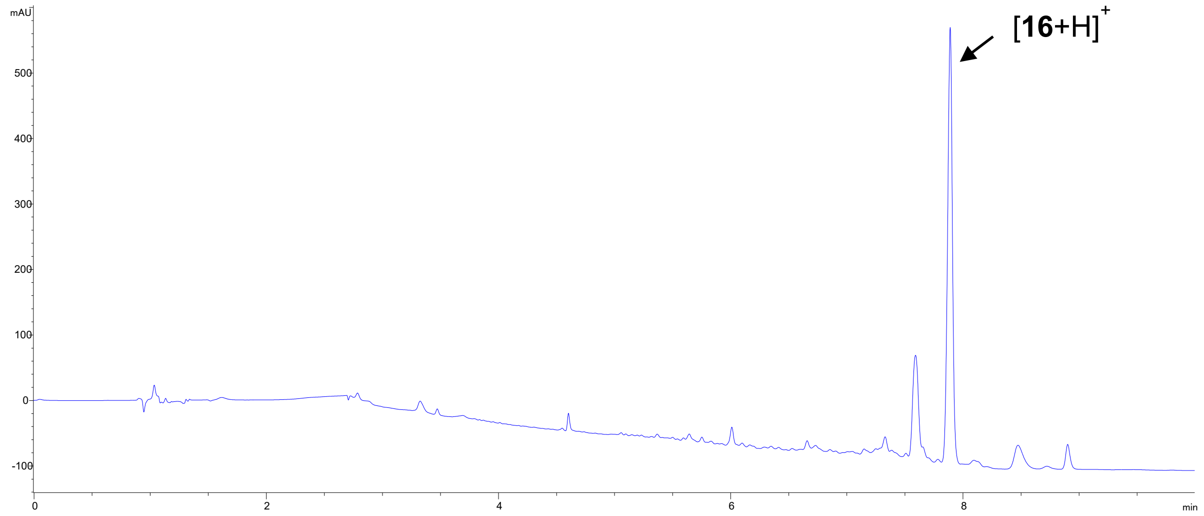

Figure S35. RP-HPLC chromatogram of crude pseudodesmin C12 (**16**). Kinetex C18 column with elution by a linear gradient over 6 min of 5 mM NH_4OAc in H_2O and CH_3CN from 100:0 to 0:100. Detection at a wavelength of λ=214 nm.

Retention time (min)	Mass (Da)	Interpretation
7.3	1153.7	$[M+H]^+$, epimerization product
7.6	1153.7	$[M+H]^+$, epimerization product
7.9	1153.7	$[M+H]^+$, product

The standard procedure for final cleavage with 0.1M HCl in HFIP was followed. The peptide was then purified by preparative RP-HPLC using elution by a linear gradient over 25 min of H_2O containing 0.1% TFA and CH_3CN from 30:70 to 5:95. The combined product containing fractions were lyophilized to obtain 12.8 mg of the pure pseudodesmin C12 (**16**) which was analyzed again by LC-MS. The overall yield after purification was 18% based on initial resin loading and the purity is 99.09%. This compound was further characterized by HRMS and NMR spectroscopy (see below).

HRMS (ESI+): calculated for pseudodesmin C12 ($C_{56}H_{100}N_{10}O_{15}$) +$H^+$: 1153.7442; found: 1153.7425; Δ = 1.5 ppm

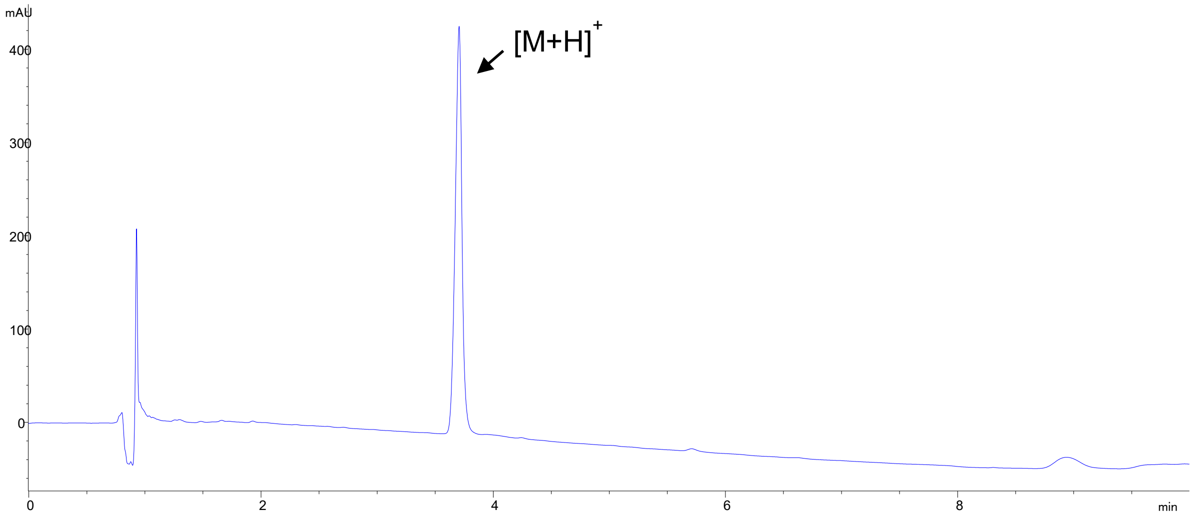

Figure S36. RP-HPLC chromatogram of purified **16**. Kinetex C18 column with elution by a linear gradient over 6 min of 5 mM NH_4OAc in H_2O and CH_3CN from 25:75 to 0:100. Detection at a wavelength of λ=214 nm.

pseudodesmin C14 (17)

Having all building blocks in hand, an identical procedure was followed to obtain pseudodesmin C14 (**17**) starting from preloaded resin (0.41 mmol/g; 0.150g, 0.0615 mmol). LC-MS analysis of the cleaved final compound confirmed successful synthesis.

<u>LC-MS analysis:</u>

Exact mass for $C_{58}H_{104}N_{10}O_{15}$ = 1180.77

LC-MS: (75-100%B in 6 minutes on Kinetex C18 column)

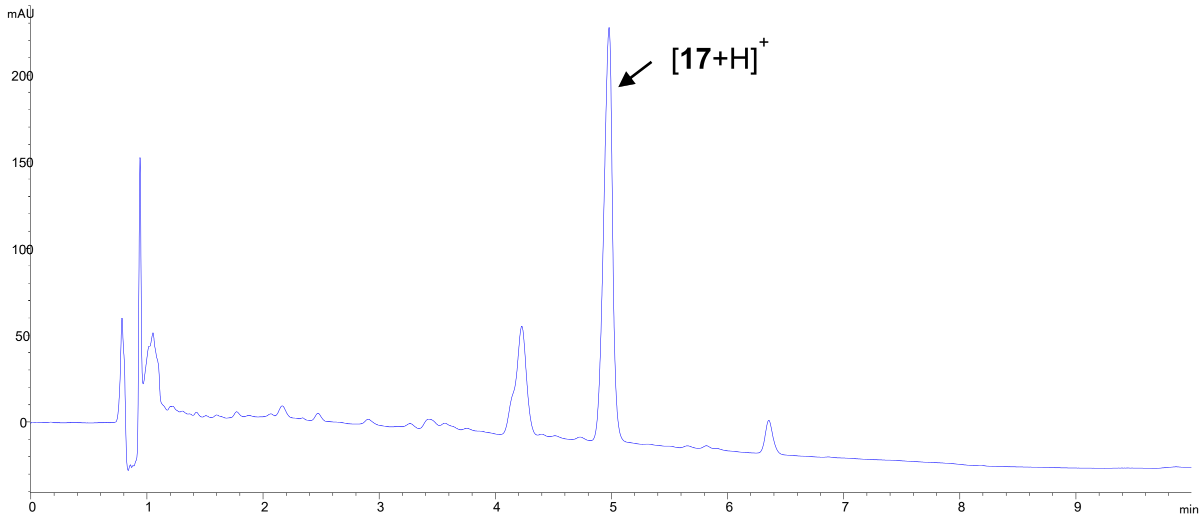

Figure S37. RP-HPLC chromatogram of crude pseudodesmin C14 (**17**). Kinetex C18 column with elution by a linear gradient over 6 min of 5 mM NH_4OAc in H_2O and CH_3CN from 25:75 to 0:100. Detection at a wavelength of λ=214 nm.

Retention time (min)	Mass (Da)	Interpretation
4.2	1181.7	$[M+H]^+$, epimerization product
5.0	1181.7	$[M+H]^+$, product
6.4	1277.6	$[M+97]^+$, TFA acylated

The standard procedure for final cleavage with 0.1M HCl in HFIP was followed. The peptide was then purified by preparative RP-HPLC using elution by a linear gradient over 25 min of H_2O containing 0.1% TFA and CH_3CN from 25:75 to 0:100. The combined product containing fractions were lyophilized to obtain 10.5 mg of the pure pseudodesmin C14 (**17**) which was analyzed again by LC-MS. The overall yield after purification was 14% based on initial resin loading and the purity is 99.31%. This compound was further characterized by HRMS and NMR spectroscopy (see below).

HRMS (ESI+): calculated for pseudodesmin C14 ($C_{58}H_{104}N_{10}O_{15}$) $+H^+$: 1181.7755; found: 1181.7749; Δ = 0.5 ppm

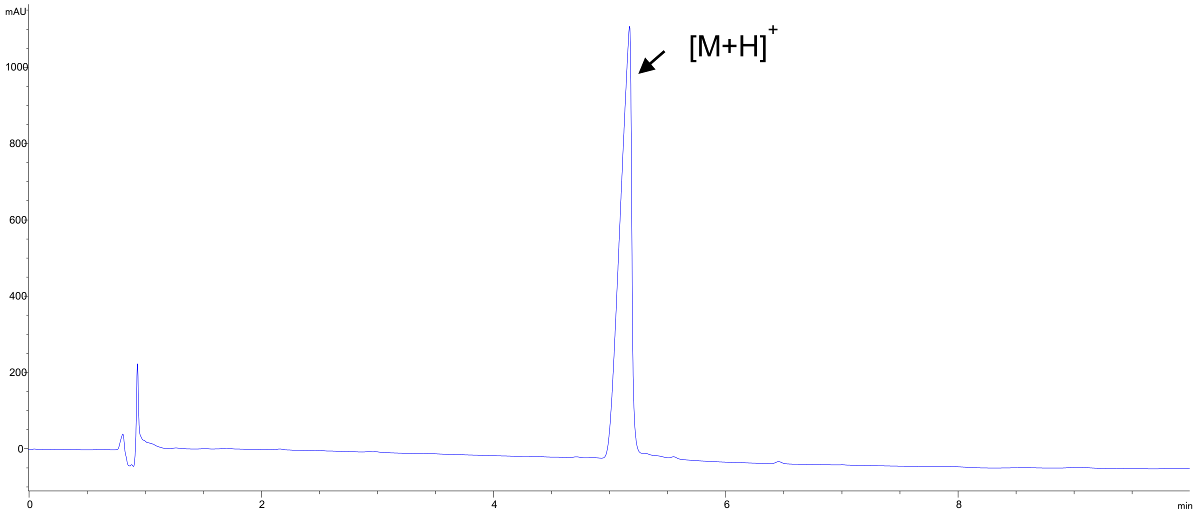

Figure S38. RP-HPLC chromatogram of purified **17**. Kinetex C18 column with elution by a linear gradient over 6 min of 5 mM NH_4OAc in H_2O and CH_3CN from 25:75 to 0:100. Detection at a wavelength of λ=214 nm.

3*S*-epi-pseudodesmin (18)

Having all building blocks in hand, an identical procedure was followed to obtain 3*S*-epi-pseudodesmin (**18**) starting from preloaded resin (0.46 mmol/g; 0.150g, 0.069 mmol). LC-MS analysis of the cleaved final compound confirmed successful synthesis.

LC-MS analysis:

Exact mass for $C_{54}H_{96}N_{10}O_{15}$ = 1124.71

LC-MS: (0-100%B in 6 minutes on Kinetex C18 column)

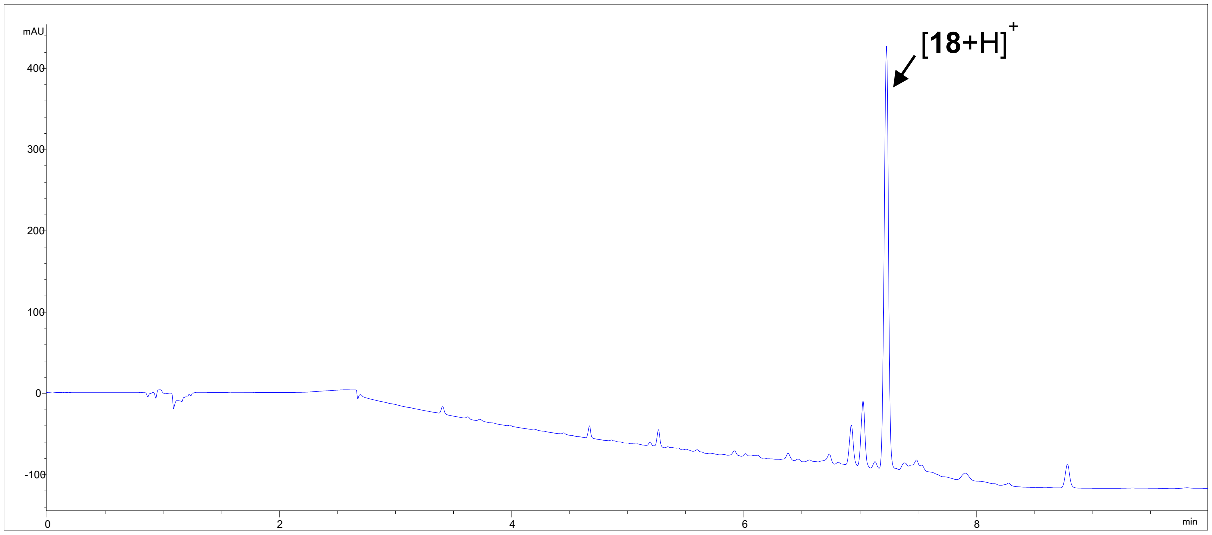

Figure S39. RP-HPLC chromatogram of crude 3*S*-epi-pseudodesmin (**18**). Kinetex C18 column with elution by a linear gradient over 6 min of 5 mM NH_4OAc in H_2O and CH_3CN from 100:0 to 0:100. Detection at a wavelength of λ=214 nm.

Retention time (min)	Mass (Da)	Interpretation
7.0	1125.6	$[M+H]^+$, epimerization product
7.2	1125.6	$[M+H]^+$, product

The standard procedure for final cleavage with 0.1M HCl in HFIP was followed. After workup with MTBE, 82.1 mg of crude 3*S*-epi-pseudodesmin (**18**) was obtained. The peptide was then purified by preparative RP-HPLC using elution by a linear gradient over 25 min of H_2O containing 0.1% TFA and CH_3CN from 40:60 to 10:90. The combined product containing fractions were lyophilized to obtain 19.9 mg of the pure peptide which was analyzed again by LC-MS. The overall yield after purification was 26% based on initial resin loading and the purity is 92.59%. This compound was further characterized by HRMS and NMR spectroscopy (see below).

HRMS (ESI+): calculated for 3*S*-epi-pseudodesmin ($C_{54}H_{96}N_{10}O_{15}$) $+H^+$: 1125.71294; found: 1125.7184; Δ = 4.9 ppm

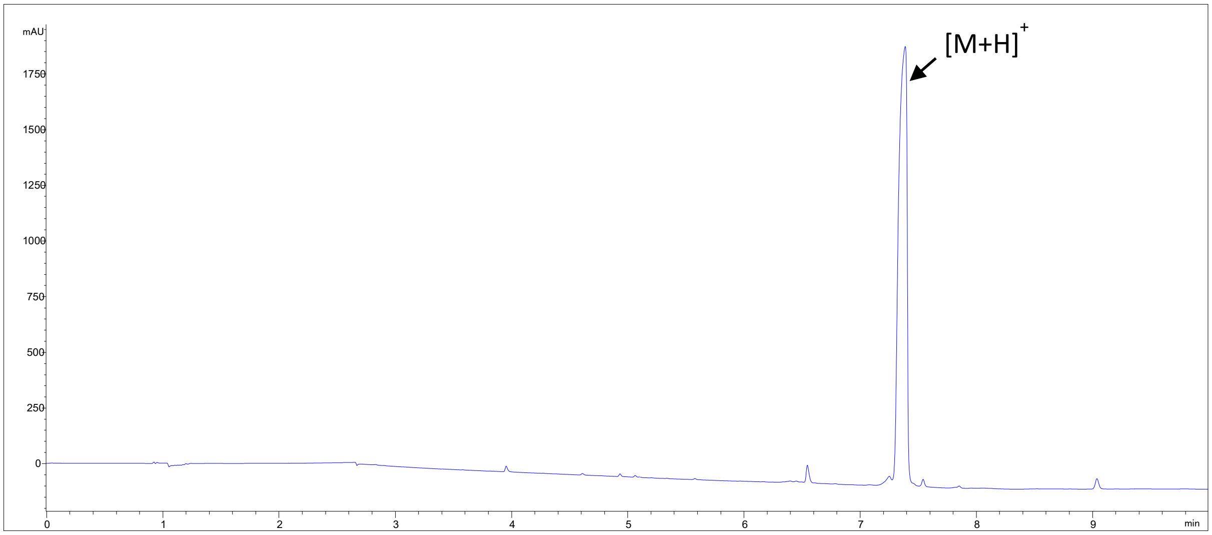

Figure S40. RP-HPLC chromatogram of purified **18**. Kinetex C18 column with elution by a linear gradient over 6 min of 5 mM NH_4OAc in H_2O and CH_3CN from 100:0 to 0:100. Detection at a wavelength of λ=214 nm.

3-deoxy-pseudodesmin (19)

Having all building blocks in hand, an identical procedure was followed to obtain 3-deoxy-pseudodesmin (**19**) starting from preloaded resin (0.51 mmol/g; 0.150g, 0.0765 mmol). LC-MS analysis of the cleaved final compound confirmed successful synthesis.

LC-MS analysis:

Exact mass for $C_{54}H_{96}N_{10}O_{14}$ = 1108.71

LC-MS: (0-100%B in 6 minutes on Kinetex C18 column)

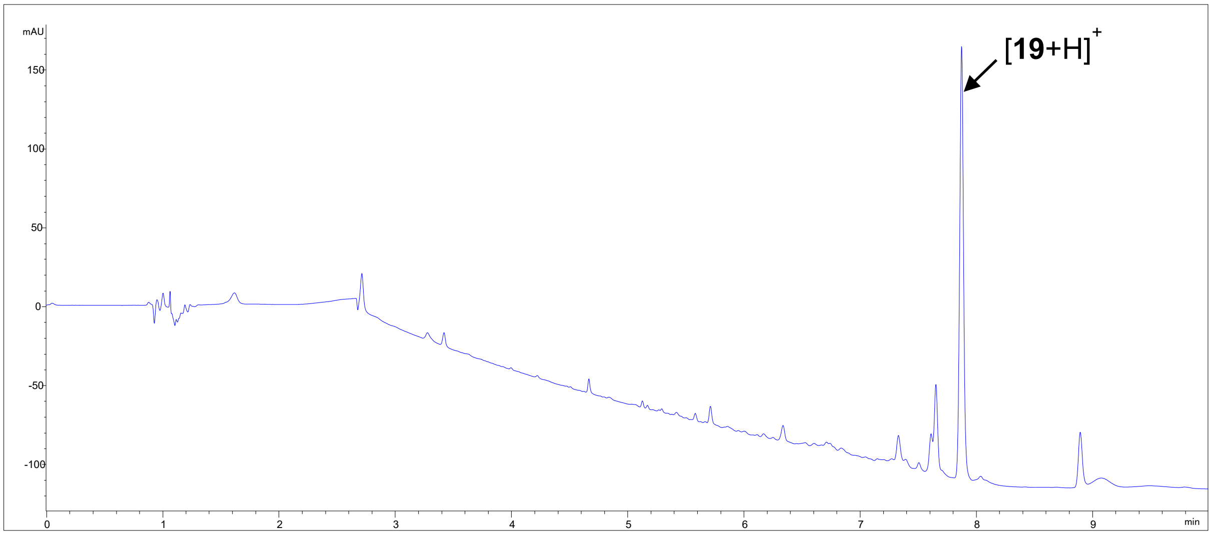

Figure S41. RP-HPLC chromatogram of crude 3-deoxy-pseudodesmin (**19**). Kinetex C18 column with elution by a linear gradient over 6 min of 5 mM NH_4OAc in H_2O and CH_3CN from 100:0 to 0:100. Detection at a wavelength of λ=214 nm.

Retention time (min)	Mass (Da)	Interpretation
7.7	1109.6	$[M+H]^+$, epimerization product
7.9	1109.6	$[M+H]^+$, product

The standard procedure for final cleavage with 0.1M HCl in HFIP was followed. After workup with MTBE, 86.1 mg of crude 3-deoxy-pseudodesmin (**19**) was obtained. The peptide was then purified by preparative RP-HPLC using elution by a linear gradient over 25 min of H_2O containing 0.1% TFA and CH_3CN from 30:70 to 05:95. The combined product containing fractions were lyophilized to obtain 16.1 mg of the pure peptide which was analyzed again by LC-MS. The overall yield after purification was 19% based on initial resin loading and the purity is 99.13%. This compound was further characterized by HRMS and NMR spectroscopy (see below).

HRMS (ESI+): calculated for 3-deoxy-pseudodesmin ($C_{54}H_{96}N_{10}O_{14}$) $+H^+$: 1109.71802; found: 1109.7164; Δ = 1.5 ppm

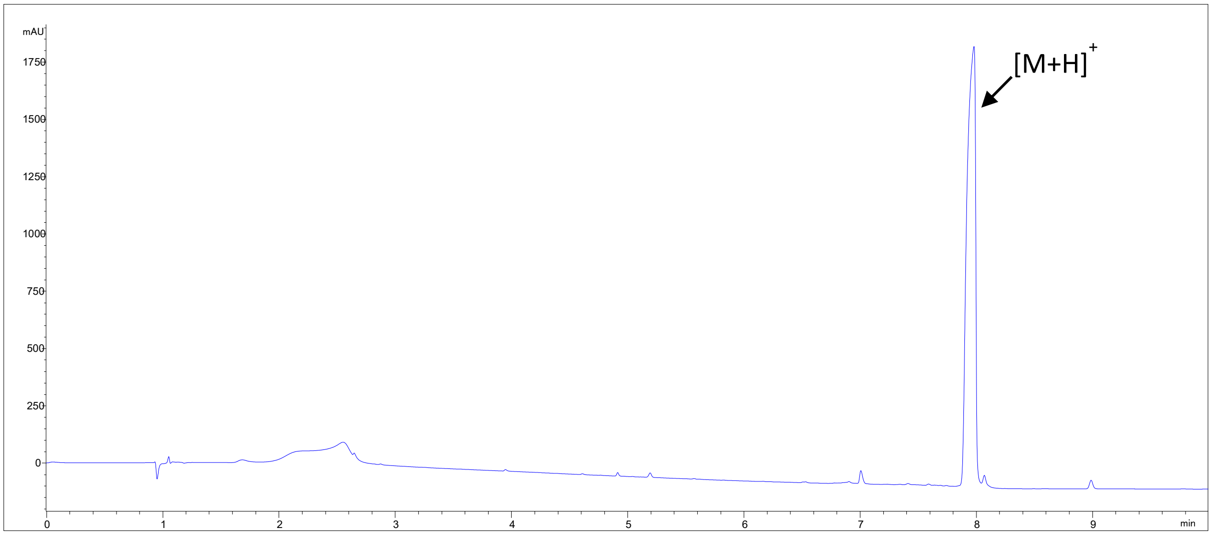

Figure S42. RP-HPLC chromatogram of purified **19**. Kinetex C18 column with elution by a linear gradient over 6 min of 5 mM NH_4OAc in H_2O and CH_3CN from 100:0 to 0:100. Detection at a wavelength of λ=214 nm.

pseudodesmin D-Dap3 (20; = substitution of ester bond by amide bond)

In this CLiP, the D-*allo*-Thr is replaced by 2,3-diaminopropionic acid (Dap). As this CLiP cannot be made with the same strategy, a new synthesis route towards pseudodesmin D-Dap3 analogues was drafted taking into account the features of the D-Dap(ivDde) building block (Scheme 2).

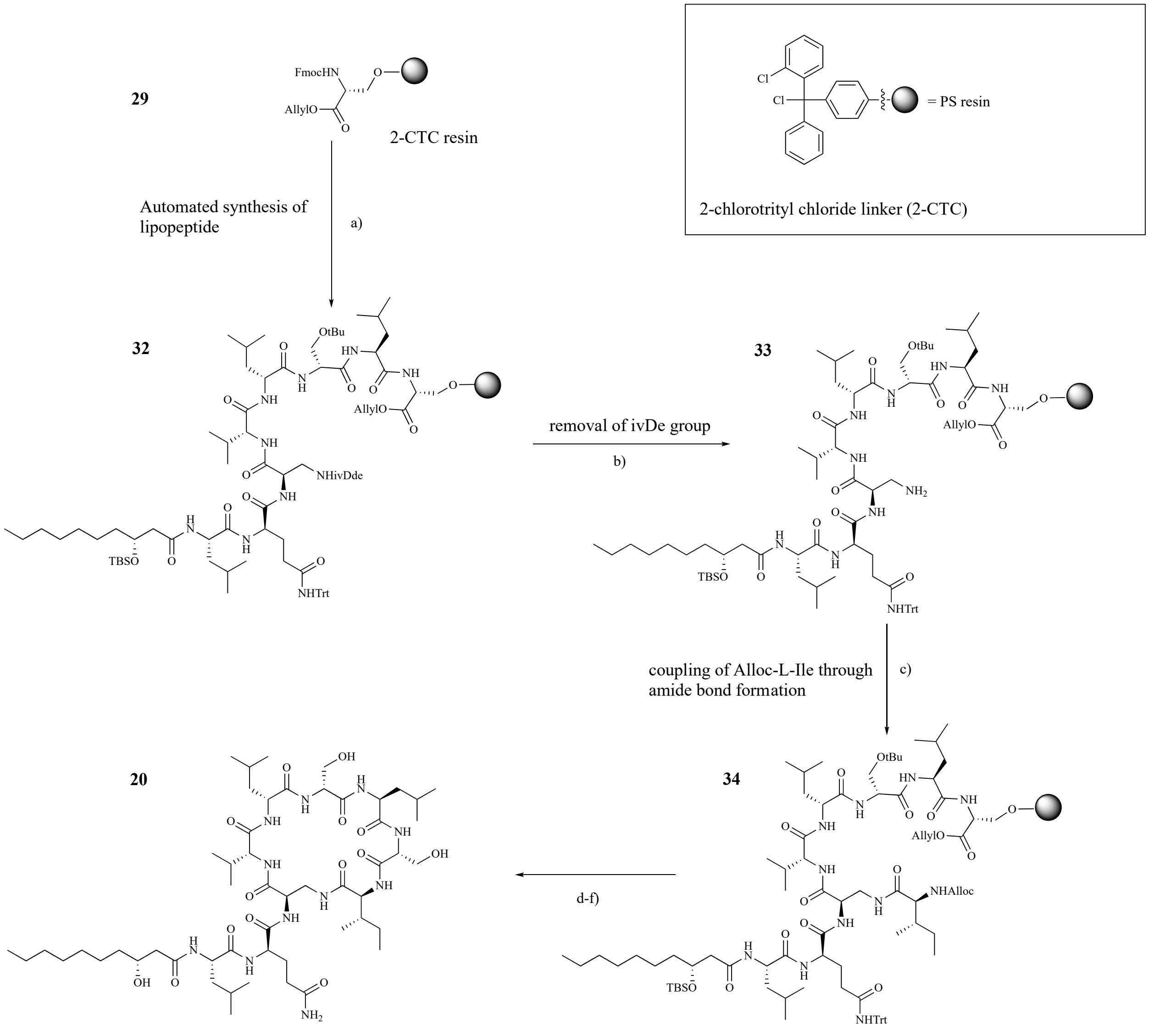

Scheme S2. Proposed route towards pseudodesmin D-Dap3 (**20**): a) (i) 20% piperidine, NMP (ii) Fmoc-AA-OH, HBTU, DIPEA, NMP; b) 2% hydrazine, allylalcohol, NMP; c) Alloc-L-Ile-OH, HBTU, DIPEA, DMF; d) $[Pd(PPh_3)_4]$, $PhSiH_3$, DCM; e) HATU, HOBt, DIPEA, DMF; f) 0.1M HCl in HFIP + 1% TIS.

Automated synthesis of lipopeptide (32)

The synthesis of the linear lipopeptide **32** was very similar as in previous strategies and started from preloaded resin **29** (0.46 mmol/g; 0.150g, 0.069 mmol). However, in this case instead of an unprotected Thr residue, an ivDde protected D-Dap was used. The attachment of the next 7 residues

and the protected lipid tail happened in an automated and iterative fashion in which each cycle consists of two steps: removal of the Fmoc group and coupling of the next building block. The preloaded resin (0.27 mmol/g; 0.100g, 0.027 mmol, 1 equiv.) is swollen in NMP prior to coupling. In this way following building blocks are coupled respectively: Fmoc-L-Leu-OH, Fmoc-D-Ser(OtBu)-OH, Fmoc-D-Leu-OH, Fmoc-D-Val-OH, Fmoc-D-Dap(ivDde)-OH, Fmoc-D-Gln(NTrt)-OH, Fmoc-L-Leu-OH and the lipid tail (*R*)-HDA(OTBS).

Control of the reaction was performed by a small scale cleavage of the peptidyl resin (1 mg) and the obtained peptide was subjected to LC-MS analysis. Upon analysis, it was noted that an isomer was present in the cleavage mixture. This isomer results from the migration of the ivDde group from the side chain of D-Dap to the alpha amino group of the main chain during automated peptide synthesis. Subsequently, the incoming Fmoc-protected amino acid reacted with the free D-Dap side chain amine. The synthesis was however continued and the isomeric product could easily be separated from the main desired one (*vide infra*).

<u>LC-MS analysis:</u>

Exact mass calcd. for $C_{63}H_{108}N_{10}O_{16}$ = 1260.79

LC-MS: (0-100%B in 6 minutes on Kinetex C18 column)

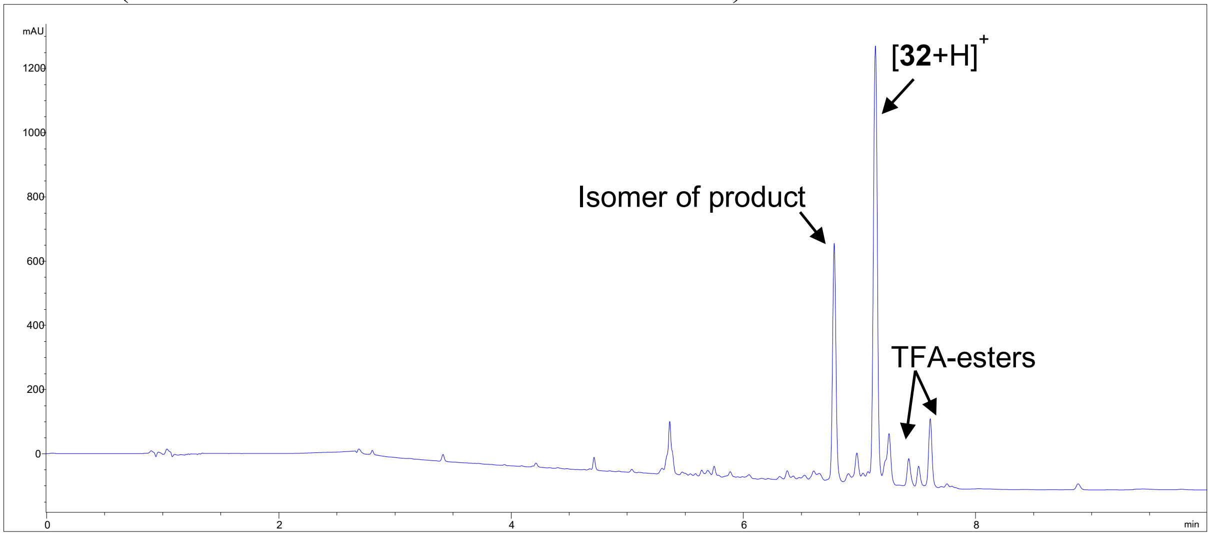

Figure S43. Chromatogram of crude **32**. Kinetex C18 column with elution by a linear gradient over 6 min from 0-100% B. Detection at a wavelength of λ=214 nm.

Retention time (min.)	Mass	Interpretation
6.8	1261.7	$[M+H]^+$, isomer of product
7.1	1261.7	$[M+H]^+$, product
7.4	1357.7	$[M+97]^+$, TFA acylated
7.6	1357.7	$[M+97]^+$, TFA acylated

Removal of ivDde protecting group (33)

The peptidyl resin was washed subsequently with DCM (3x) and NMP(3x). Next, a solution of hydrazine hydrate/NMP/allyl alcohol (2/48/50 %V, 10 ml/g resin) was added to the resin and the reaction vessel was shaken for 10 minutes (Rohwedder et al., 1998). The deprotection step was repeated twice and the resin was subsequently washed with NMP (3x), MeOH (3x) and DCM (3x). Small scale cleavage and LC-MS analysis on the obtained peptide confirmed complete removal of the ivDde protecting group.

<u>LC-MS analysis:</u>

Exact mass calcd. for $C_{50}H_{90}N_{10}O_{14}$ = 1054.66

LC-MS: (0-100%B in 6 minutes on Kinetex C18 column)

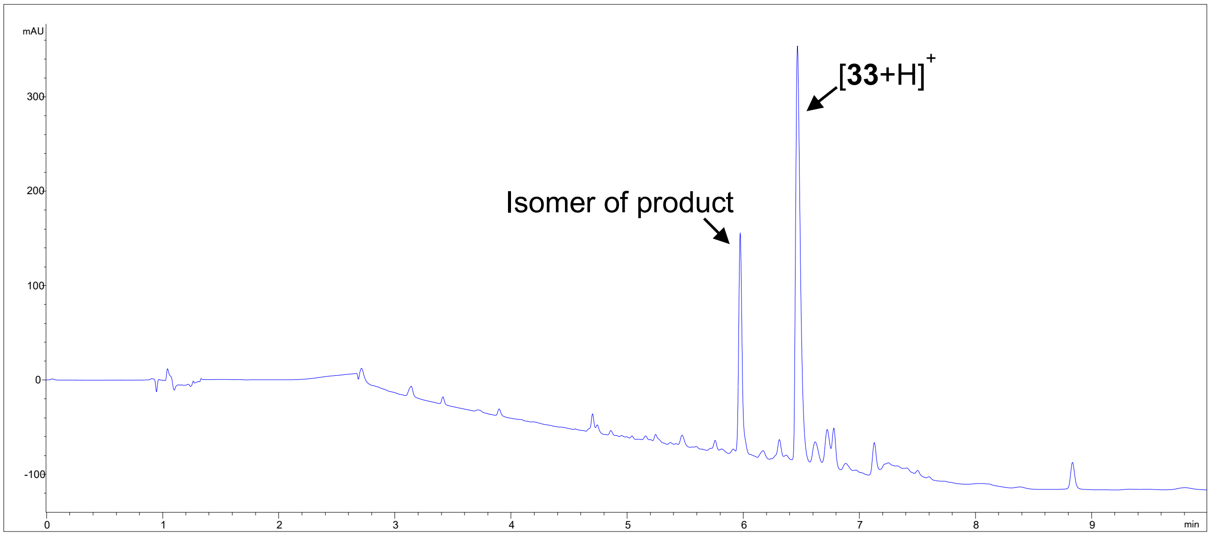

Figure S44. Chromatogram of crude **33**. Kinetex C18 column with elution by a linear gradient over 6 min from 0 to 100 %B. Detection at a wavelength of λ=214 nm.

Retention time (min.)	Mass	Interpretation
5.9	1055.6	$[M+H]^+$, isomer of product
6.5	1055.6	$[M+H]^+$, product

Coupling of Alloc-L-Ile (34)

This time Alloc-L-Ile was coupled through amide bond formation. The standard for the manual coupling of an amino acid was used with elongated reaction time. In brief, 5 equiv. of Alloc-L-Ile in dry DMF (0.5 M), 4.9 equiv. HBTU in dry DMF (0.5 M) and 10 equiv. DIPEA (2M in NMP) were added to the resin. After 4 hours of shaking, the reagent is filtered and the resin is washed

with DMF (3x), MeOH (3x) and DCM (3x). Small scale cleavage and LC-MS analysis on the obtained peptide confirmed a complete coupling.

LC-MS analysis:

Exact mass calcd. for $C_{60}H_{105}N_{11}O_{17}$ = 1251.77

LC-MS: (0-100%B in 6 minutes on Kinetex C18 column)

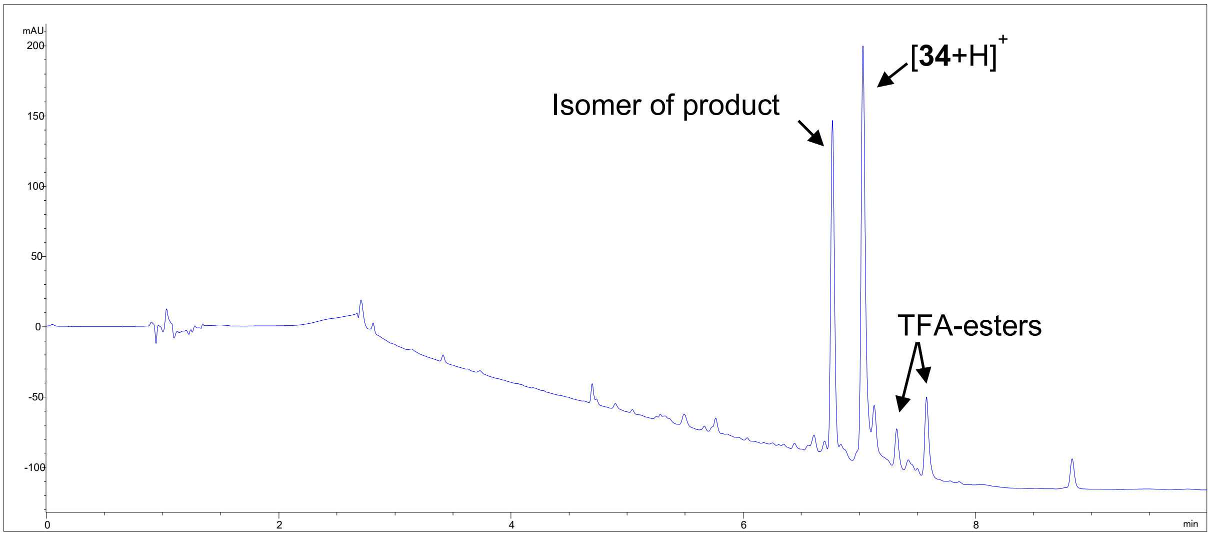

Figure S45. Chromatogram of crude **34**. Kinetex C18 column with elution by a linear gradient over 6 min from 0 to 100 %B. Detection at a wavelength of λ=214 nm.

Retention time (min.)	Mass	Interpretation
6.8	1252.7	$[M+H]^+$, isomer of product
7.0	1252.7	$[M+H]^+$, product
7.3	1348.7	$[M+97]^+$, TFA acylated
7.5	1348.7	$[M+97]^+$, TFA acylated

Alloc-deprotection and on-resin cyclization (20)

Beads were dried on the oil pump overnight prior to removal of Alloc and Allyl protecting groups. Peptidyl resin was swollen under argon atmosphere with DCM. A solution of phenylsilane (4.14 mmol, 60 equiv.) and a catalytic amount of $Pd(PPh_3)_4$ (0.017 mmol, 0.25 equiv.) in DCM (3 ml) was added. The reaction vessel was flushed with argon and shielded from light and shaken for 1h. This step was repeated once more. Subsequently, the resin was washed with DCM (3x), MeOH (3x) and DCM (3x) and dried for 2 hours. Next, the resin was swollen with dry DMF and kept under argon atmosphere. A solution of DIPEA (5 equiv.), HATU (5 equiv.) and HOBt (5 equiv.) was added and the reaction vessel was agitated for 4 h at room temperature. After reaction, the

resin was washed with DMF (3x), MeOH (3x), and DCM (3x). Small scale cleavage and LC-MS analysis of **20** confirmed successful cyclization.

<u>LC-MS analysis:</u>

Exact mass for $C_{53}H_{95}N_{11}O_{14}$ = 1109.71

LC-MS: (0-100%B in 6 minutes on Kinetex C18 column)

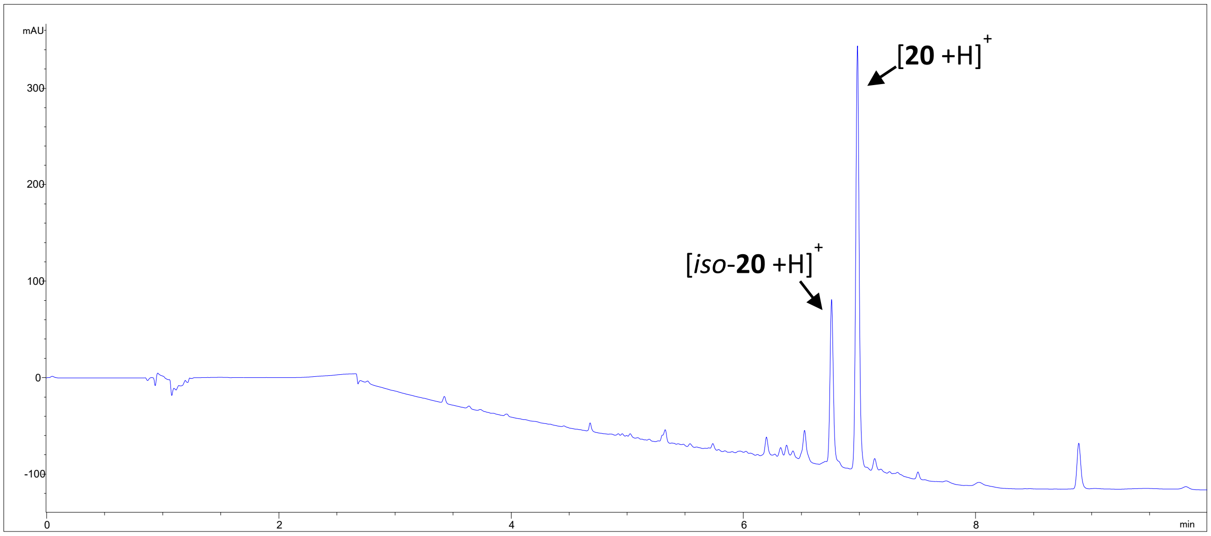

Figure S46. RP-HPLC chromatogram of crude pseudodesmin **D**-Dap3 **(20)**. Kinetex C18 column with elution by a linear gradient over 6 min of 5 mM NH_4OAc in H_2O and CH_3CN from 100:0 to 0:100. Detection at a wavelength of λ=214 nm.

Retention time (min.)	Mass	Interpretation
6.8	1110.6	$[M+H]^+$, isomer of product
7.0	1110.6	$[M+H]^+$, product

Total deprotection and purification

The standard procedure for final cleavage with 0.1M HCl in HFIP was followed. After workup with MTBE, 73.4 mg of crude pseudodesmin D-Dap3 (**20**) was obtained. The peptide was then purified by preparative RP-HPLC using elution by a linear gradient over 25 min of H_2O containing 0.1% TFA and CH_3CN from 50:50 to20:80. The combined product containing fractions were lyophilized to obtain 19.1 mg of the pure peptide which was analyzed again by LC-MS. The overall yield of **20** after purification was 25% based on initial resin loading and the purity is 92.06%. This compound was further characterized by HRMS and NMR spectroscopy (see below).

HRMS (ESI+): calculated for pseudodesmin D-Dap3 **(20)** ($C_{53}H_{95}N_{11}O_{14}$) $+H^+$: 1110.71327; found: 1110.7109; Δ = 2.1 ppm

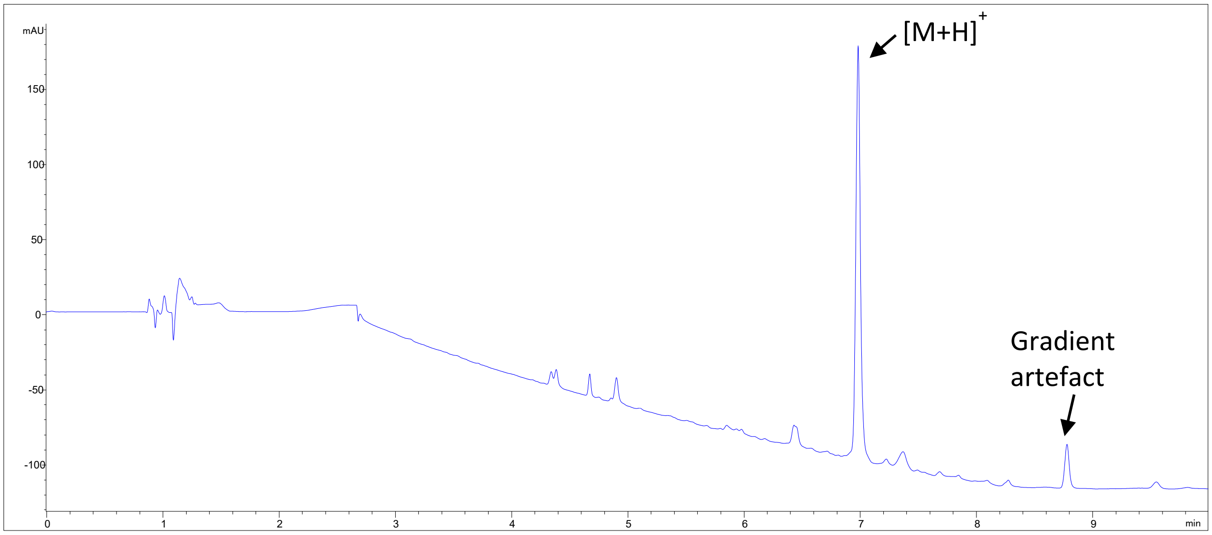

Figure S47. RP-HPLC chromatogram of purified **20**. Kinetex C18 column with elution by a linear gradient over 6 min of 5 mM NH_4OAc in H_2O and CH_3CN from 100:0 to 0:100. Detection at a wavelength of λ=214 nm.

Single amino acid substitutions

Pseudodesmin D-Ser3 (21)

Having all building blocks in hand, an identical procedure to the synthesis strategy towards pseudodesmin A was followed to obtain pseudodesmin D-Ser3 (**21**) starting from preloaded resin (0.46 mmol/g; 0.150g, 0.069 mmol). LC-MS analysis of the cleaved final compound confirmed successful synthesis.

LC-MS analysis:

Exact mass for $C_{53}H_{94}N_{10}O_{15}$ = 1110.69 Da

LC-MS: (0-100%B in 6 minutes on Kinetex C18 column)

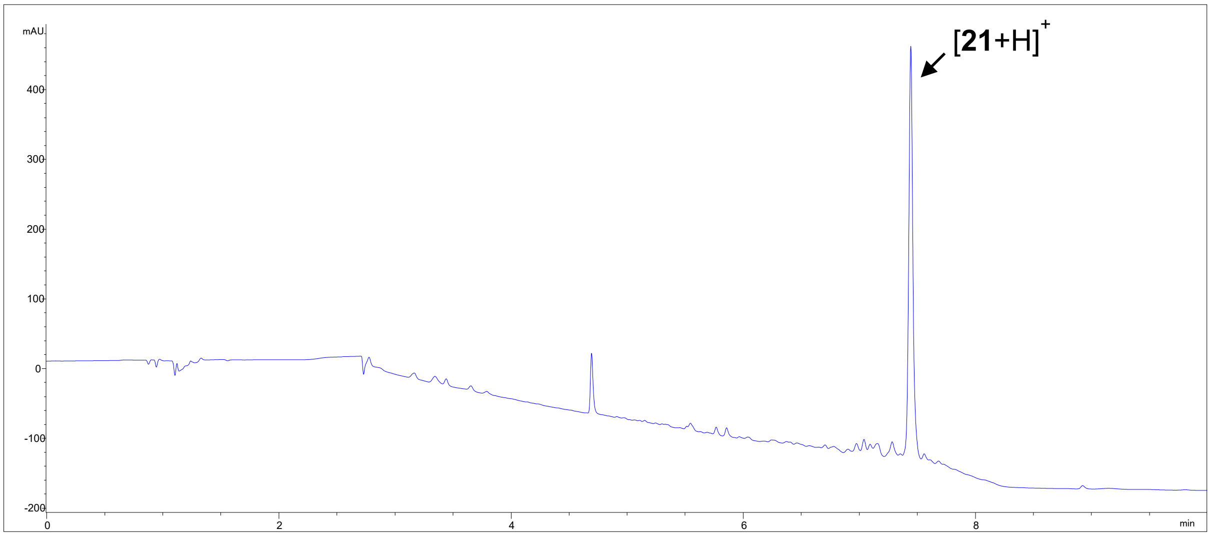

Figure S48. RP-HPLC chromatogram of crude pseudodesmin D-Ser3 (**21**). Kinetex C18 column with elution by a linear gradient over 6 min of 5 mM NH_4OAc in H_2O and CH_3CN from 100:0 to 0:100. Detection at a wavelength of λ=214 nm.

Retention time (min)	Mass (Da)	Interpretation
7.4	1111.6	$[M+H]^+$, product

The standard procedure for final cleavage with 0.1M HCl in HFIP was followed. After workup with MTBE, 75.9 mg of crude pseudodesmin D-Ser3 (**21**) was obtained. The peptide was then purified by semi-preparative RP-HPLC using elution by a linear gradient over 25 min of H_2O containing 0.1% TFA and CH_3CN from 35:65 to 10:90 The combined product containing fractions were lyophilized to obtain 15.2 mg of the pure peptide which was analyzed again by LC-MS. The overall yield after purification was 20% based on initial resin loading and the purity is 99.33%. This compound was further characterized by HRMS and NMR spectroscopy (see below).

HRMS (ESI+): calculated for pseudodesmin D-Ser3 ($C_{53}H_{94}N_{10}O_{15}$) $+H^+$: 1111.69729; found: 1125.6965; Δ = 0.7 ppm

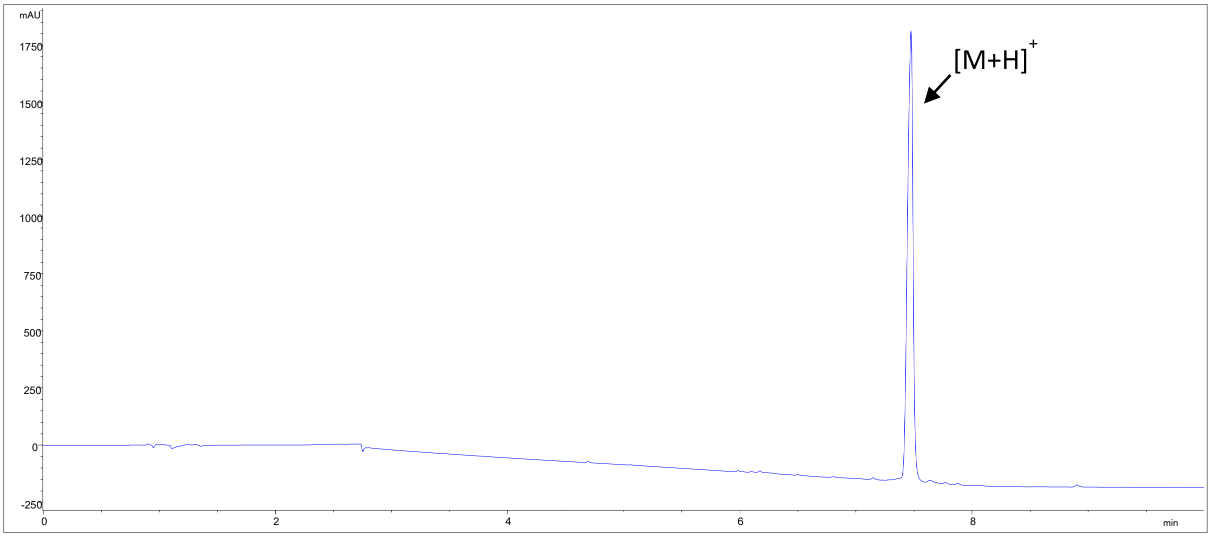

Figure S49. RP-HPLC chromatogram of purified **21**. Kinetex C18 column with elution by a linear gradient over 6 min of 5 mM NH_4OAc in H_2O and CH_3CN from 100:0 to 0:100. Detection at a wavelength of λ=214 nm.

Viscosinamide (22)

Having all building blocks in hand, an identical procedure was followed to obtain viscosinamide (**22**) starting from the preloaded resin (0.46 mmol/g; 0.150g, 0.069 mmol). LC-MS analysis of the cleaved final compound confirmed successful synthesis.

LC-MS analysis:

Exact mass for $C_{54}H_{96}N_{10}O_{15}$ = 1124.71

LC-MS: (0-100%B in 6 minutes on Kinetex C18 column)

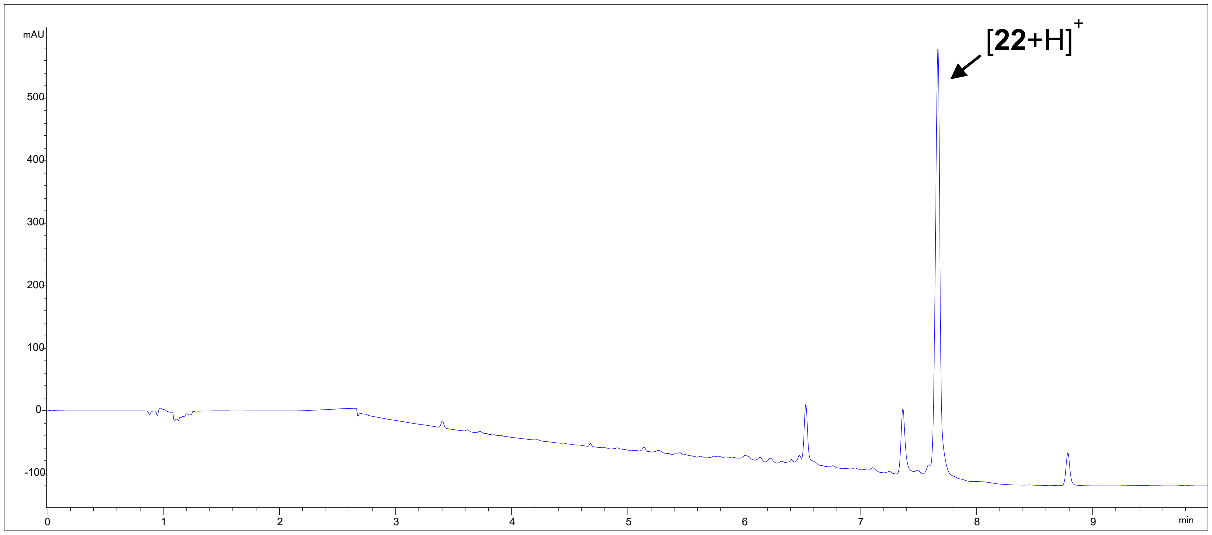

Figure S50. RP-HPLC chromatogram of crude viscosinamide (**22**). Kinetex C18 column with elution by a linear gradient over 6 min of 5 mM NH_4OAc in H_2O and CH_3CN from 100:0 to 0:100. Detection at a wavelength of λ=214 nm.

Retention time (min)	Mass (Da)	Interpretation
7.4	1125.6	$[M+H]^+$, epimerization product
7.7	1125.6	$[M+H]^+$, product

The standard procedure for final cleavage with 0.1M HCl in HFIP was followed. After workup with MTBE, 85.6 mg of crude viscosinamide was obtained. The peptide was then purified by preparative RP-HPLC using elution by a linear gradient over 25 min of H_2O containing 0.1% TFA and CH_3CN from 35:65 to 10:90. The combined product containing fractions were lyophilized to obtain 27.4 mg of the pure peptide which was analyzed again by LC-MS. The overall yield after purification was 35% based on initial resin loading and the purity is 96.23%. This compound was further characterized by HRMS and NMR spectroscopy (see below).

HRMS (ESI+): calculated for viscosinamide ($C_{54}H_{96}N_{10}O_{15}$) $+H^+$: 1125.71294; found: 1125.7139; Δ = 0.9 ppm

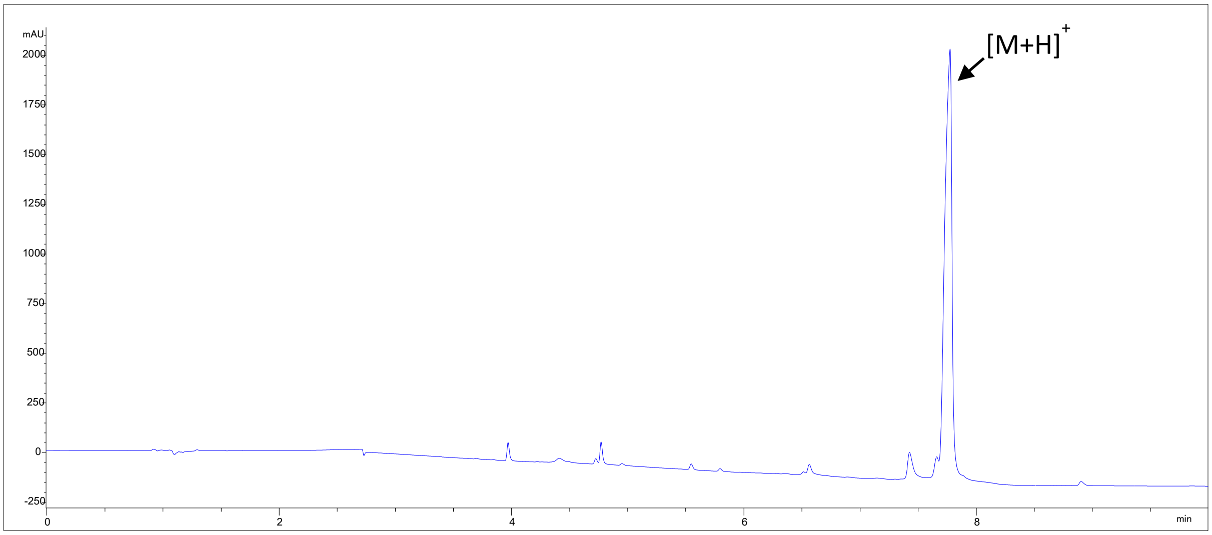

Figure S51. RP-HPLC chromatogram of purified **22**. Kinetex C18 column with elution by a linear gradient over 6 min of 5 mM NH_4OAc in H_2O and CH_3CN from 100:0 to 0:100. Detection at a wavelength of λ=214 nm.

Viscosinamide L5I (23)

Having all building blocks in hand, an identical procedure was followed to obtain viscosinamide L5I (**23**) starting from preloaded resin (0.46 mmol/g; 0.150g, 0.069 mmol). LC-MS analysis of the cleaved final compound confirmed successful synthesis.

LC-MS analysis:

Exact mass for $C_{54}H_{96}N_{10}O_{15}$ = 1124.71

LC-MS: (0-100%B in 6 minutes on Kinetex C18 column)

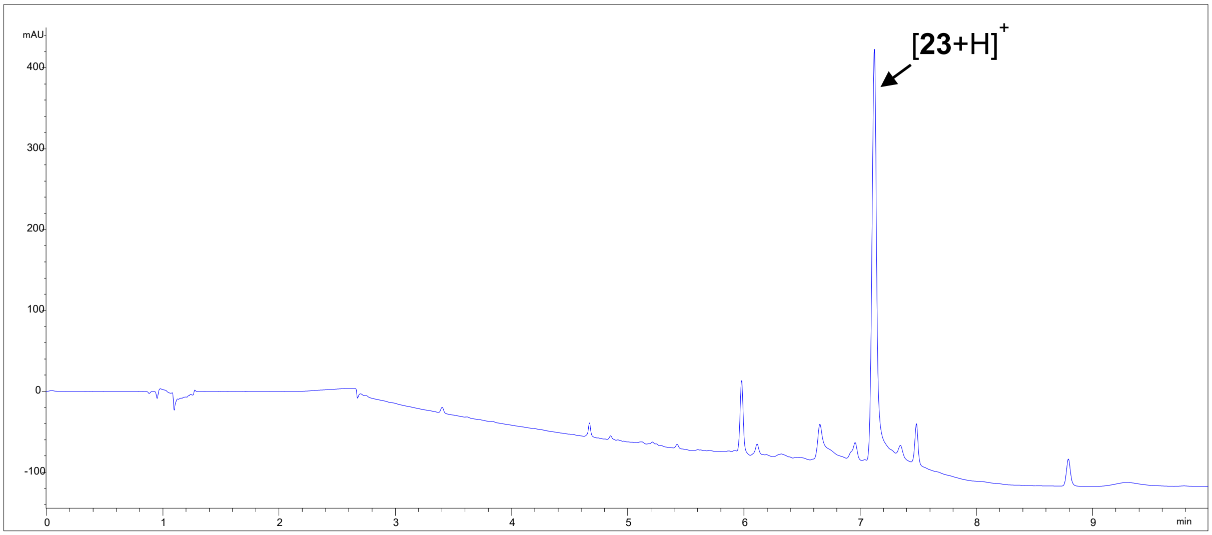

Figure S52. RP-HPLC chromatogram of crude viscosinamide L5I (**23**). Kinetex C18 column with elution by a linear gradient over 6 min of 5 mM NH_4OAc in H_2O and CH_3CN from 100:0 to 0:100. Detection at a wavelength of λ=214 nm.

Retention time (min)	Mass (Da)	Interpretation
6.7	1125.6	$[M+H]^+$, epimerization product
7.1	1125.6	$[M+H]^+$, product

The standard procedure for final cleavage with 0.1M HCl in HFIP was followed. After workup with MTBE, 82.6 mg of crude viscosinamide L5I (**23**) was obtained. The peptide was then purified by preparative RP-HPLC using elution by a linear gradient over 21 min of H_2O containing 0.1% TFA and CH_3CN from 40:60 to 19:81. The combined product containing fractions were lyophilized to obtain 9.0 mg of the pure peptide which was analyzed again by LC-MS. The overall yield after purification was 12% based on initial resin loading and the purity is 75.2%. This compound was further characterized by HRMS and NMR spectroscopy (see below).

HRMS (ESI+): calculated for viscosinamide L5I ($C_{54}H_{96}N_{10}O_{15}$) $+H^+$: 1125.71294; found: 1125.7115; Δ = 1.3 ppm

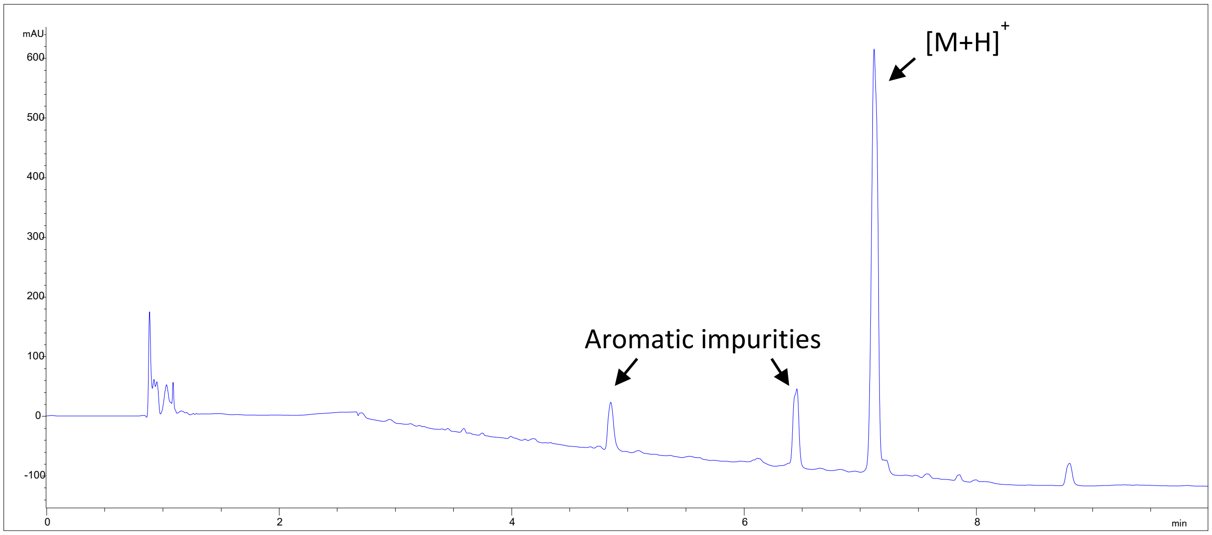

Figure S53. RP-HPLC chromatogram of purified **23**. Kinetex C18 column with elution by a linear gradient over 6 min of 5 mM NH_4OAc in H_2O and CH_3CN from 100:0 to 0:100. Detection at a wavelength of λ=214 nm.

pseudodesmin NMe1 (24)

Having all building blocks in hand, an identical procedure, as previously reported (De Vleeschouwer et al., 2017), was followed to obtain pseudodesmin NMe1 (**24**) starting from preloaded resin (0.51 mmol/g; 0.150g, 0.0765 mmol). For the coupling of the residue (the lipid tail) after the introduction of the N-methylated building block, we used adapted coupling conditions as mentioned before. LC-MS analysis of the cleaved final compound confirmed successful synthesis.

<u>LC-MS analysis:</u>

Exact mass calcd. for $C_{55}H_{98}N_{10}O_{15}$ = 1138.72

LC-MS: (0-100%B in 6 minutes on Kinetex C18 column)

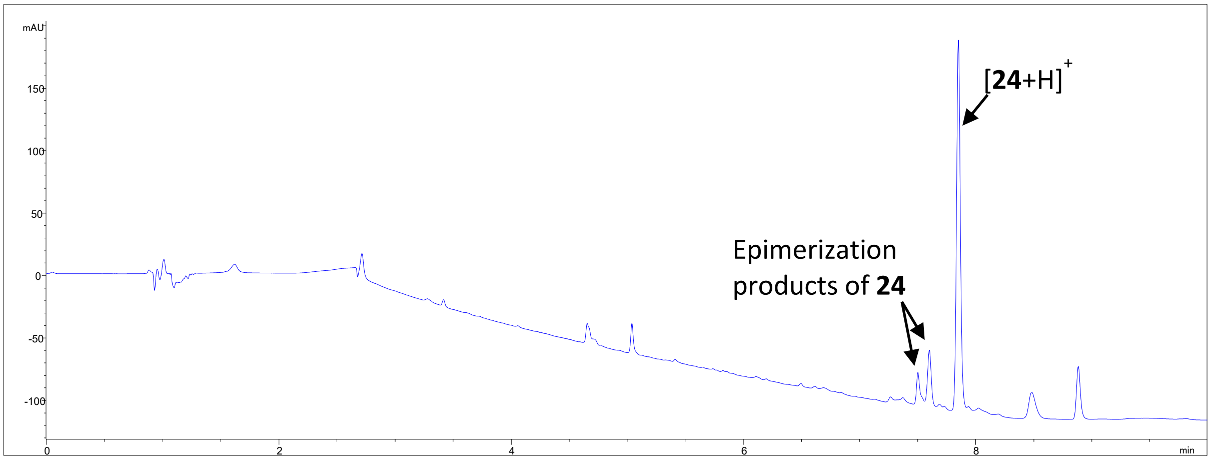

Figure S54. Chromatogram of crude pseudodesmin NMe1 (**24**) after cleavage with 95% TFA. Kinetex C18 column with elution by a linear gradient over 6 min of 5 mM NH_4OAc in H_2O and CH_3CN from 100:0 to 0:100.

Retention time (min.)	Mass	Interpretation
7.9	1139.6	$[M+H]^+$, product

The standard procedure for final cleavage with 0.1M HCl in HFIP was followed. After workup with MTBE, 57.3 mg of crude pseudodesmin NMe1 (**24**) was obtained. The peptide was then purified by semi-preparative RP-HPLC using elution by a linear gradient over 25 min of H_2O containing 0.1% TFA and CH_3CN from 30:70 to 5:95. The combined product containing fractions were lyophilized to obtain 15.0 mg of the pure peptide which was analyzed again by LC-MS. The overall yield after purification was 17% based on initial resin loading and the purity is 94.03%. This compound was further characterized NMR spectroscopy (see below).

HRMS (ESI+): calculated for pseudodesmin NMe1 ($C_{54}H_{98}N_{10}O_{15}$) $+H^+$: 1139.72859; found: 1139.7292; $\Delta = 0.5$ ppm

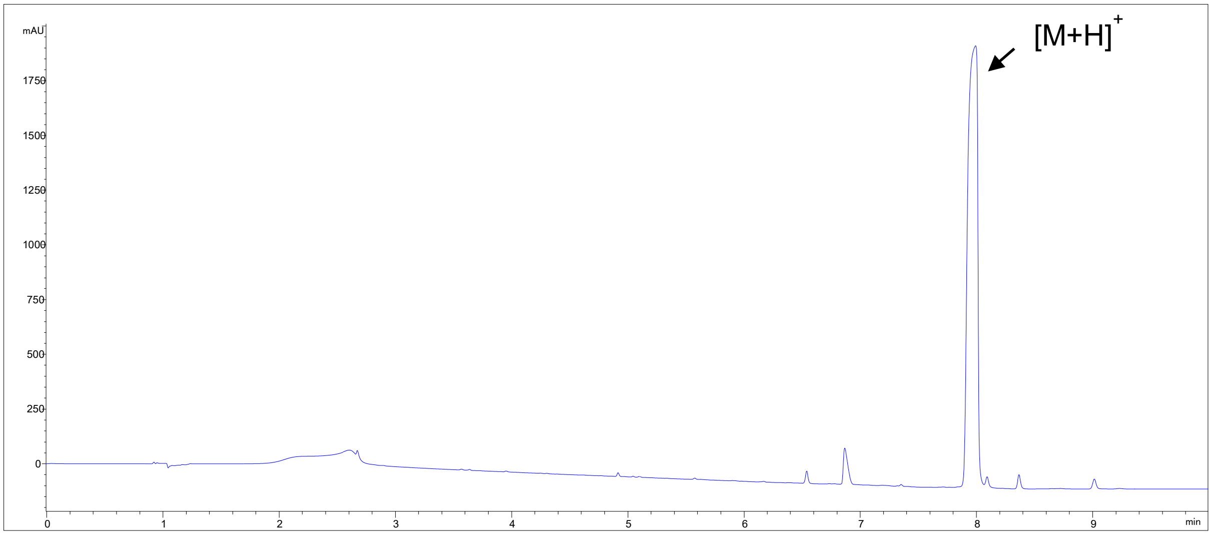

Figure S55. RP-HPLC chromatogram of purified **24**. Kinetex C18 column with elution by a linear gradient over 6 min of 5 mM NH_4OAc in H_2O and CH_3CN from 100:0 to 0:100. Detection at a wavelength of λ=214 nm.

pseudodesmin NMe7 (25)

Having all building blocks in hand, an identical procedure, as previously reported (De Vleeschouwer et al., 2017), was followed to obtain pseudodesmin NMe7 (**25**) starting from preloaded resin (0.51 mmol/g; 0.150g, 0.0765 mmol). For the coupling of the residue (Fmoc-D-Ser(O*t*Bu)-OH) after the introduction of the N-methylated building block, we used adapted coupling conditions as mentioned before. LC-MS analysis of the cleaved final compound confirmed successful synthesis.

LC-MS analysis:

Exact mass calcd. for $C_{55}H_{98}N_{10}O_{15}$ = 1138.72

LC-MS: (0-100%B in 6 minutes on Kinetex C18 column)

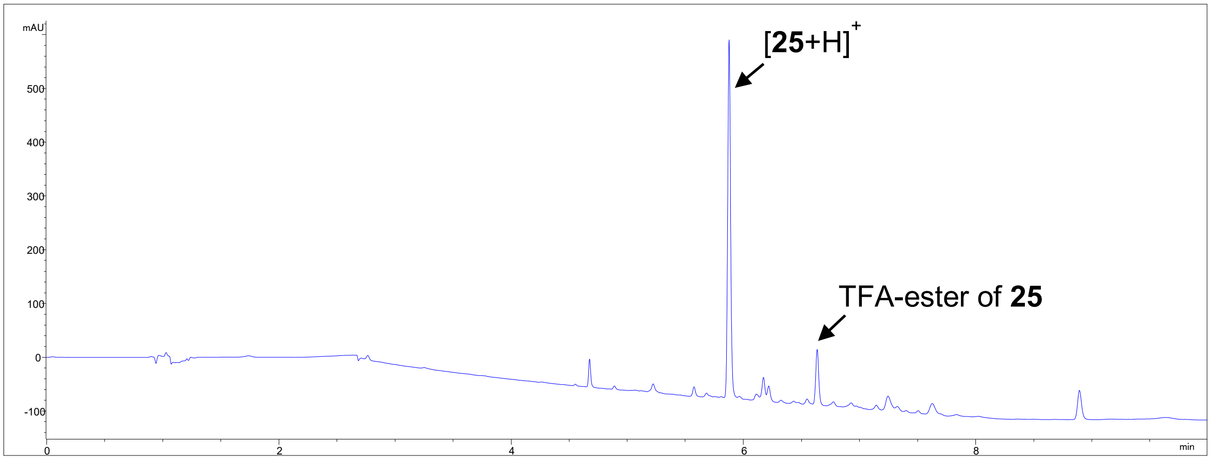

Figure S56. The chromatogram of crude pseudodesmin NMe7 (**25**) after small scale cleavage conditions with 95% TFA. Kinetex C18 column with elution by a linear gradient over 6 min of 5 mM NH_4OAc in H_2O and CH_3CN from 100:0 to 0:100.

Retention time (min.)	Mass	Interpretation
5.9	1139.6	$[M+H]^+$, product
6.6	1235.6	$[M+97]^+$, TFA acylated

Based on our experience with the synthesis of pseudodesmin NMe1, we decided to use the conditions with 0.1M HCl in HFIP for the final cleavage of the peptide and the standard procedure for final cleavage with 0.1M HCl in HFIP was followed. After workup with MTBE, 80.4 mg of crude pseudodesmin NMe7 (**25**) was obtained. However, LC-MS analysis clearly indicated that during the cleavage reaction, compound **25** was almost completely degraded (Figure S55).

LC-MS analysis:

Exact mass calcd. for $C_{55}H_{98}N_{10}O_{15}$ = 1138.72

LC-MS: (0-100%B in 6 minutes on Kinetex C18 column)

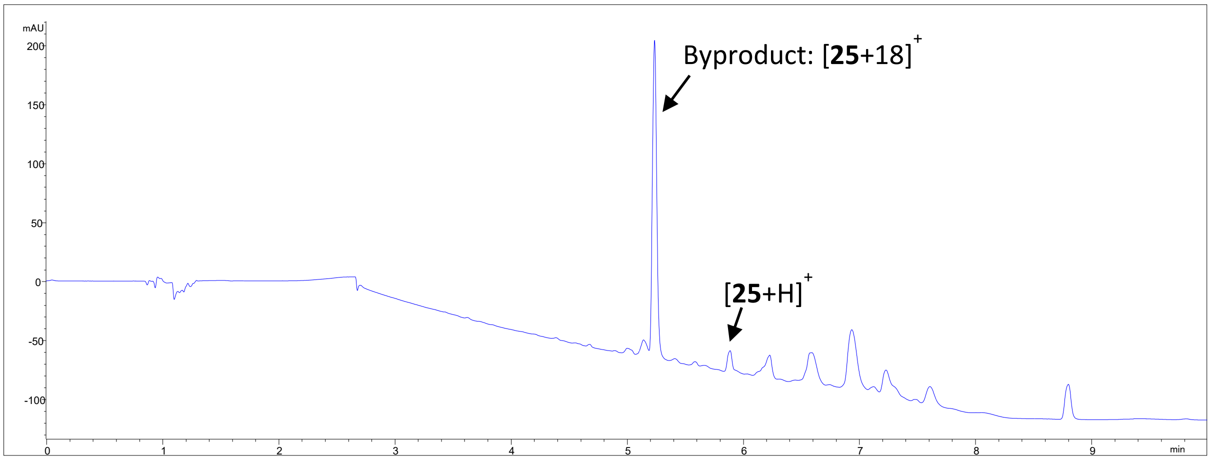

Figure S57. Chromatogram of crude pseudodesmin NMe7 (**25**) after small scale cleavage conditions with 0.1M HCl in HFIP. Kinetex C18 column with elution by a linear gradient over 6 min of 5 mM NH_4OAc in H_2O and CH_3CN from 100:0 to 0:100.

Retention time (min.)	Mass	Interpretation
5.2	1157.6	$[M+18]^+$, hydrolysis product
5.9	1139.6	$[M+H]^+$, product

The peptide was then purified by preparative RP-HPLC using elution by a linear gradient over 25 min of H_2O containing 0.1% TFA and CH_3CN from 60:40 to 35:65. The combined product containing fractions were lyophilized to obtain 1.4 mg of the pure pseudodesmin NMe7 which was analyzed again by LC-MS. The overall yield after purification was 1.6% based on initial resin loading and the purity is 91.60%. This compound was further characterized by HRMS and NMR spectroscopy (see below).

HRMS (ESI+): calculated for pseudodesmin NMe7 ($C_{54}H_{98}N_{10}O_{15}$) $+H^+$: 1139.72859; found: 1139.7281; Δ = 0.4 ppm

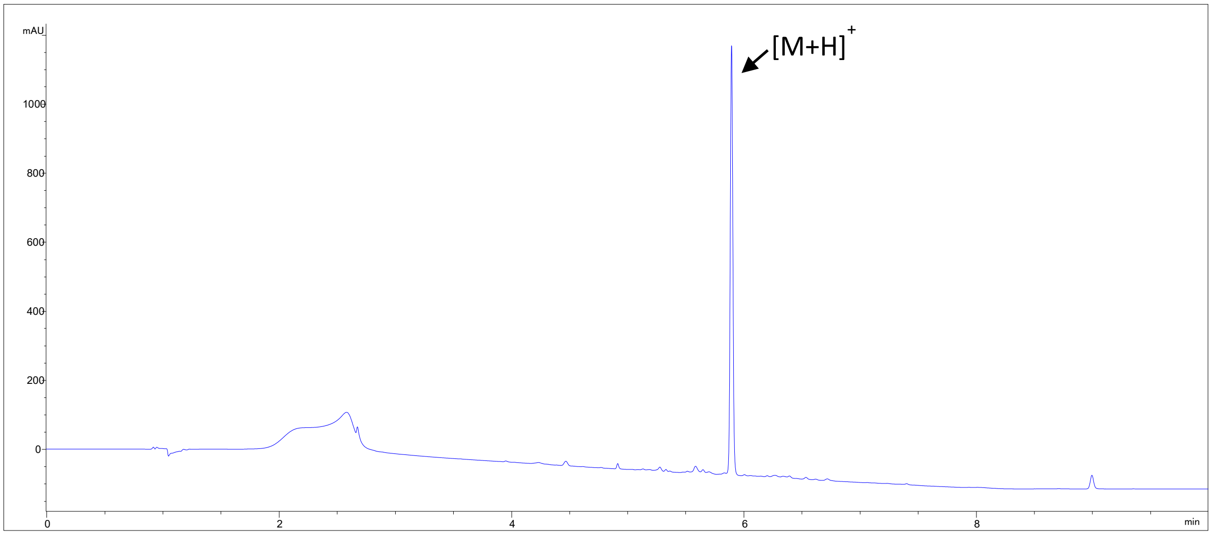

Figure S58. RP-HPLC chromatogram of purified **25**. Kinetex C18 column with elution by a linear gradient over 6 min of 5 mM NH_4OAc in H_2O and CH_3CN from 100:0 to 0:100. Detection at a wavelength of λ=214 nm.

Trp-analogues of pseudodesmin

pseudodesmin L1W (26)

Having all building blocks in hand, an identical procedure was followed to obtain pseudodesmin L1W (**26**) starting from preloaded resin (0.46 mmol/g; 0.150g, 0.069 mmol). LC-MS analysis of the cleaved final compound confirmed successful synthesis.

LC-MS analysis:

Exact mass for $C_{59}H_{95}N_{11}O_{15}$ = 1197.70

LC-MS: (0-100%B in 6 minutes on Kinetex C18 column)

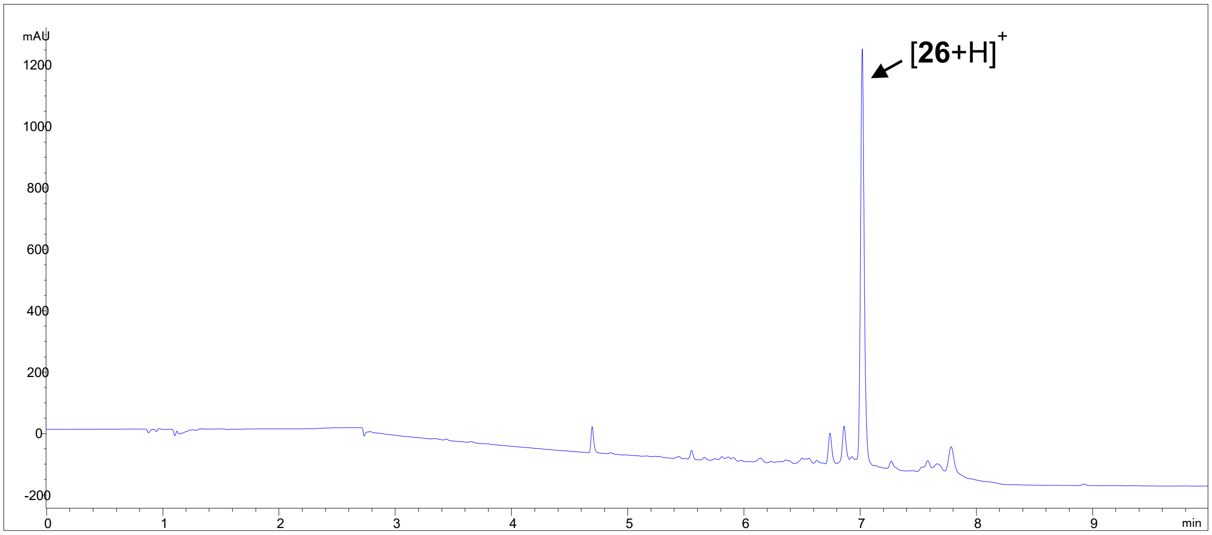

Figure S59. RP-HPLC chromatogram of crude pseudodesmin L1W (**26**). Kinetex C18 column with elution by a linear gradient over 6 min of 5 mM NH_4OAc in H_2O and CH_3CN from 100:0 to 0:100. Detection at a wavelength of λ=214 nm.

Retention time (min)	Mass (Da)	Interpretation
6.9	1198.7	$[M+H]^+$, epimerization product
7.0	1198.7	$[M+H]^+$, product

The standard procedure for final cleavage with 0.1M HCl in HFIP was followed. After workup with MTBE, 61.7 mg of crude pseudodesmin L1W (**26**) was obtained. The peptide was then purified by preparative RP-HPLC using elution by a linear gradient over 21 min of H_2O containing 0.1% TFA and CH_3CN from 40:60 to 19:81. The combined product containing fractions were lyophilized to obtain 7.7 mg of the pure peptide which was analyzed again by LC-MS. The overall yield after purification was 9% based on initial resin loading and the purity is 95.79%. This compound was further characterized by HRMS and NMR spectroscopy (see below).

HRMS (ESI+): calculated for pseudodesmin L1W ($C_{59}H_{95}N_{11}O_{15}$) $+H^{+}$: 1198.70819; found: 1198.7060; Δ = 1.8 ppm

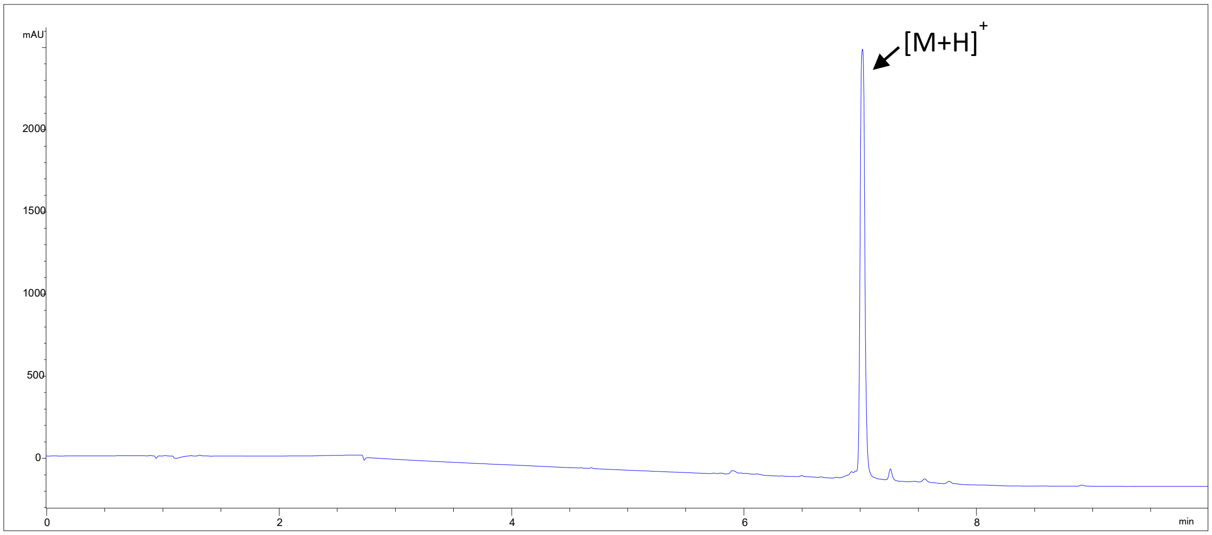

Figure S60. RP-HPLC chromatogram of purified **26**. Kinetex C18 column with elution by a linear gradient over 6 min of 5 mM NH_4OAc in H_2O and CH_3CN from 100:0 to 0:100. Detection at a wavelength of λ=214 nm.

pseudodesmin L5W (27)

Having all building blocks in hand, an identical procedure was followed to obtain pseudodesmin L5W (**27**) starting from preloaded resin (0.46 mmol/g; 0.150g, 0.069 mmol). LC-MS analysis of the cleaved final compound confirmed successful synthesis.

LC-MS analysis:

Exact mass for $C_{59}H_{95}N_{11}O_{15}$ = 1197.70

LC-MS: (0-100%B in 6 minutes on Kinetex C18 column)

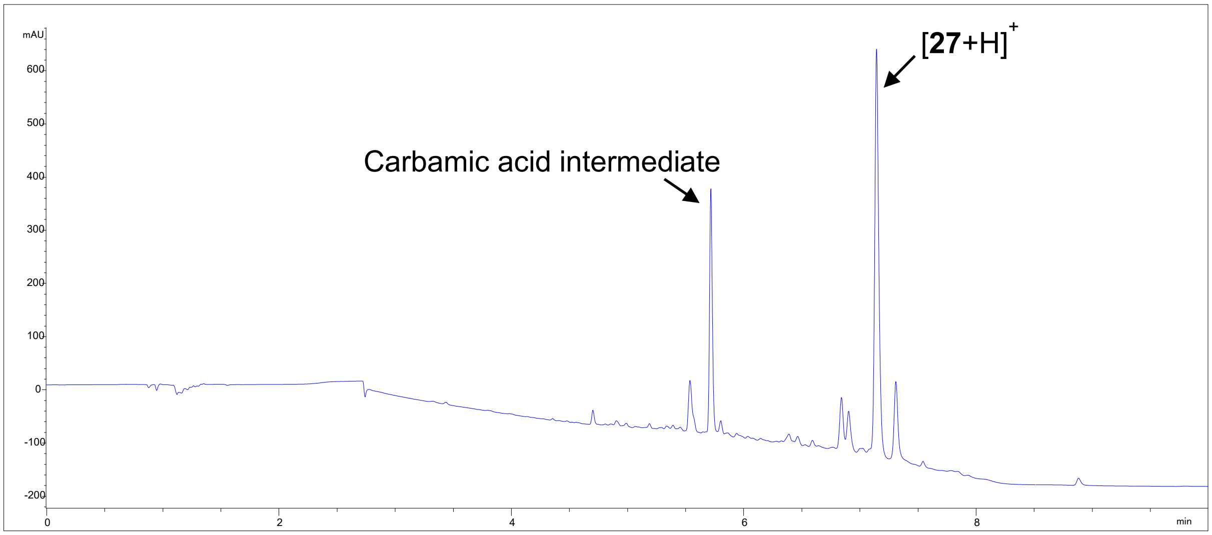

Figure S61. RP-HPLC chromatogram of crude pseudodesmin L5W (**27**). Kinetex C18 column with elution by a linear gradient over 6 min of 5 mM NH_4OAc in H_2O and CH_3CN from 100:0 to 0:100. Detection at a wavelength of λ=214 nm.

Retention time (min)	Mass (Da)	Interpretation
5.7	1242.5	$[M+44]^+$, Trp- carbamic acid intermediate
7.1	1198.5	$[M+H]^+$, product

The standard procedure for final cleavage with 0.1M HCl in HFIP was followed. After workup with MTBE, 82.8 mg of crude pseudodesmin L5W (**27**) was obtained. The peptide was then purified by preparative RP-HPLC using elution by a linear gradient over 22 min of H_2O containing 0.1% TFA and CH_3CN from 40:60 to 18:82. The combined product containing fractions were lyophilized to obtain 19.9 mg of the pure peptide which was analyzed again by LC-MS. The overall yield after purification was 24% based on initial resin loading and the purity is 97.36%. This compound was further characterized by HRMS and NMR spectroscopy (see below).

HRMS (ESI+): calculated for pseudodesmin L5W ($C_{59}H_{95}N_{11}O_{15}$) $+H^+$: 1198.70819; found: 1198.7071; Δ = 0.9 ppm

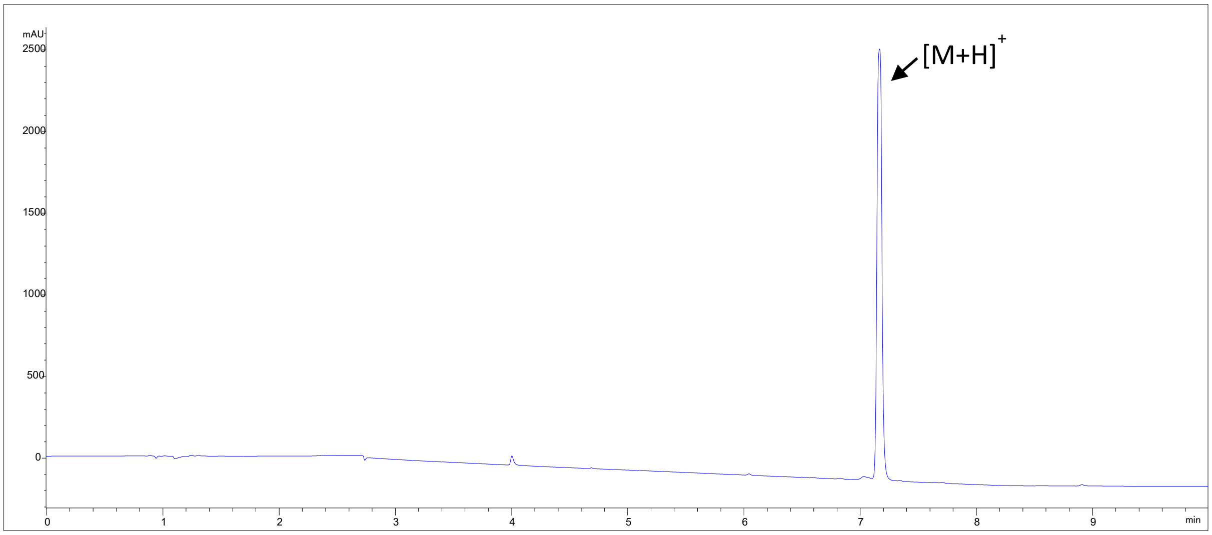

Figure S62. RP-HPLC chromatogram of purified **27**. Kinetex C18 column with elution by a linear gradient over 6 min of 5 mM NH_4OAc in H_2O and CH_3CN from 100:0 to 0:100. Detection at a wavelength of λ=214 nm.

pseudodesmin L7W (28)

Having all building blocks in hand, an identical procedure was followed to obtain pseudodesmin L7W (**28**) starting from preloaded resin (0.46 mmol/g; 0.150g, 0.069 mmol). LC-MS analysis of the cleaved final compound confirmed successful synthesis.

LC-MS analysis:

Exact mass for $C_{59}H_{95}N_{11}O_{15}$ = 1197.70

LC-MS: (0-100%B in 6 minutes on Kinetex C18 column)

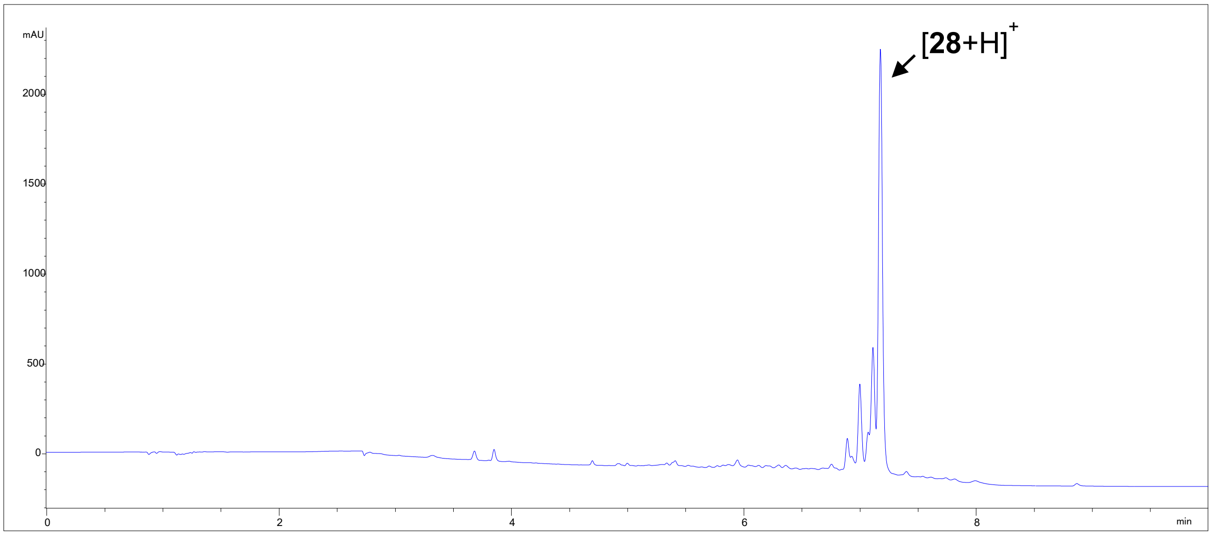

Figure S63. RP-HPLC chromatogram of crude pseudodesmin L7W (**28**). Kinetex C18 column with elution by a linear gradient over 6 min of 5 mM NH_4OAc in H_2O and CH_3CN from 100:0 to 0:100. Detection at a wavelength of λ=214 nm.

Retention time (min)	Mass (Da)	Interpretation
7.1	1198.5	$[M+H]^+$, epimerization product
7.2	1198.5	$[M+H]^+$, product

The standard procedure for final cleavage with 0.1M HCl in HFIP was followed. After workup with MTBE, 80.1 mg of crude pseudodesmin L7W (**28**) was obtained. The peptide was then purified by preparative RP-HPLC using elution by a linear gradient over 25 min of H_2O containing 0.1% TFA and CH_3CN from 40:60 to 15:85. The combined product containing fractions were lyophilized to obtain 22.3 mg of the pure peptide which was analyzed again by LC-MS. The overall yield after purification was 27% based on initial resin loading and the purity is 98.38%. This compound was further characterized by HRMS and NMR spectroscopy (see below).

HRMS (ESI+): calculated for pseudodesmin L7W ($C_{59}H_{95}N_{11}O_{15}$) $+H^+$: 1198.70819; found: 1198.7068; Δ = 1.2 ppm

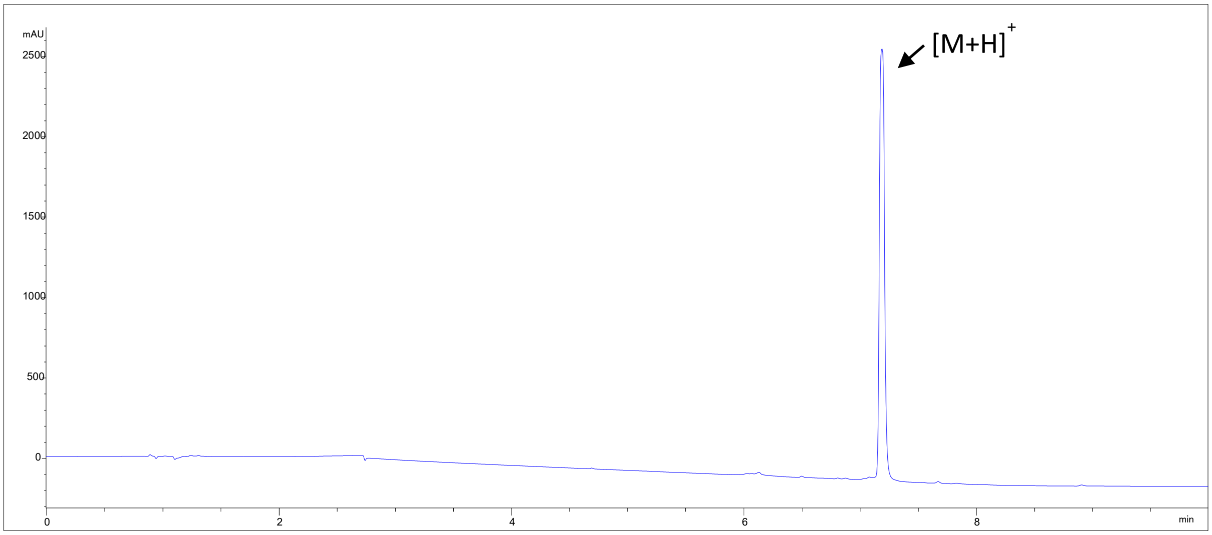

Figure S64. RP-HPLC chromatogram of purified **28**. Kinetex C18 column with elution by a linear gradient over 6 min of 5 mM NH_4OAc in H_2O and CH_3CN from 100:0 to 0:100. Detection at a wavelength of λ=214 nm.

NMR characterization of cyclic lipodepsipeptides 1-28

For comparison purposes, the NMR spectra of pseudodesmin A in CD_3CN were also included below.

pseudodesmin A (1)

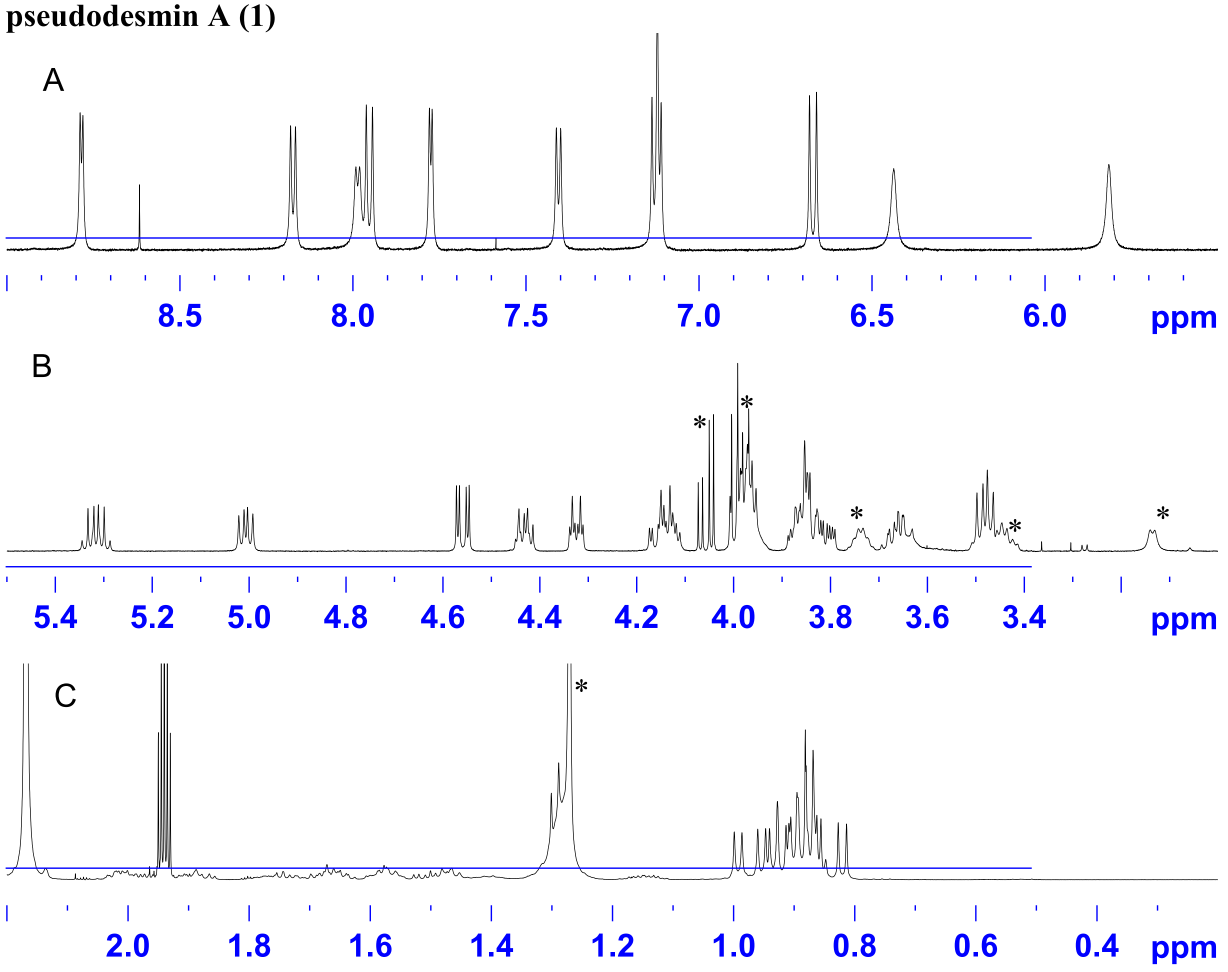

Figure S65. ^1H spectrum of pseudodesmin A (**1**) (CD_3CN, 25°C, 500MHz) A) H^N region, B) H^α region and C) aliphatic region. Asterisks indicate the resonances of an unidentified impurity.

Table S2. ^{1}H and ^{13}C assignment of **psdA** (CD$_3$CN, 25°C, 500 MHz). Scalar couplings in Hz.

			^{1}H δ [ppm]	^{13}C δ [ppm]
**(*R*)-HDA**				
		CO		175.06
		CH2 α1	2.36	44.66
		CH2 α2	2.43	44.66
		CH β	3.96	69.47
		CH2 γ	1.47	38.17
		CH2 δ1	1.29	26.25
		CH2 δ2	1.41	26.25
		CH2 ε	1.27	30.30
		CH2 ζ	1.27	30.30
		CH2 η	1.27	32.58
		CH2 θ	1.28	23.33
		CH3 ι	0.88	14.33
		OH		3.63
**L-Leu1**				
$^3J_{HNH\alpha}$	5.43	NH	7.99	
		CH α	3.86	53.68
		CO		175.26
		CH$_2$ β1	1.67	39.24
		CH$_2$ β2	1.74	39.24
		CH γ	1.67	25.32
		CH$_3$ δ	0.90	22.03
		CH$_3$ δ	0.94	23.13
**D-Gln2**				
$^3J_{HNH\alpha}$	3.98	NH	8.78	
		CH α	3.97	57.48
		CO		176.61
		CH$_2$ β	2.01	26.39
		CH$_2$ γ	2.37	31.97
		CO δ		176.01
		NH$_2$	5.82/6.44	
**D-*allo*-Thr3**				
$^3J_{HNH\alpha}$	6.98	NH	8.17	
$^3J_{H\alpha H\beta}$	ND	CH α	3.99	61.67
		CO		174.25
		CH β	5.32	70.22
		CH$_3$ γ	1.30	18.47
**D-Val4**				
$^3J_{HNH\alpha}$	6.23	NH	7.41	
		CH α	3.48	65.01
		CO		174.55
		CH β	2.17	29.88
		CH$_3$ γ	0.92	19.44
		CH$_3$ γ	0.95	20.99
**D-Leu5**				
$^3J_{HNH\alpha}$	4.05	NH	7.78	
		CH α	3.97	55.64
		CO		173.54
		CH$_2$ β1	1.50	40.56
		CH$_2$ β2	1.67	40.56
		CH γ	1.77	25.42
		CH$_3$ δ	0.88	21.21
		CH$_3$ δ	0.86	23.25
**D-Ser6**				
$^3J_{HNH\alpha}$	7.36	NH	7.13	
		CH α	4.32	56.32
		CO		171.89
		CH$_2$ β1	3.81	64.66
		CH$_2$ β2	4.15	64.66
		OH γ	5.01	
**L-Leu7**				
$^3J_{HNH\alpha}$	5.04	NH	7.11	
		CH α	4.13	54.79
		CO		173.78
		CH$_2$ β1	1.58	41.97
		CH$_2$ β2	1.89	41.97
		CH γ	1.89	25.42
		CH$_3$ δ	0.90	21.27
		CH$_3$ δ	0.99	23.40
**D-Ser8**				
$^3J_{HNH\alpha}$	8.96	NH	7.95	
		CH α	4.43	56.90
		CO		171.81
		CH$_2$ β1	3.66	63.10
		CH$_2$ β2	3.86	63.10
		OH γ	not allocated	
**L-Ile9**				
$^3J_{HNH\alpha}$	10.04	NH	6.67	
		CH α	4.56	57.11
		CO		169.99
		CH β	1.98	36.81
		CH$_3$ γ	0.82	16.15
		CH$_2$ γ1	0.97	25.16
		CH$_2$ γ2	1.15	25.16
		CH$_3$ δ	0.86	12.23

ND= not determined

Enantiomer of pseudodesmin (2; *ent*-1)

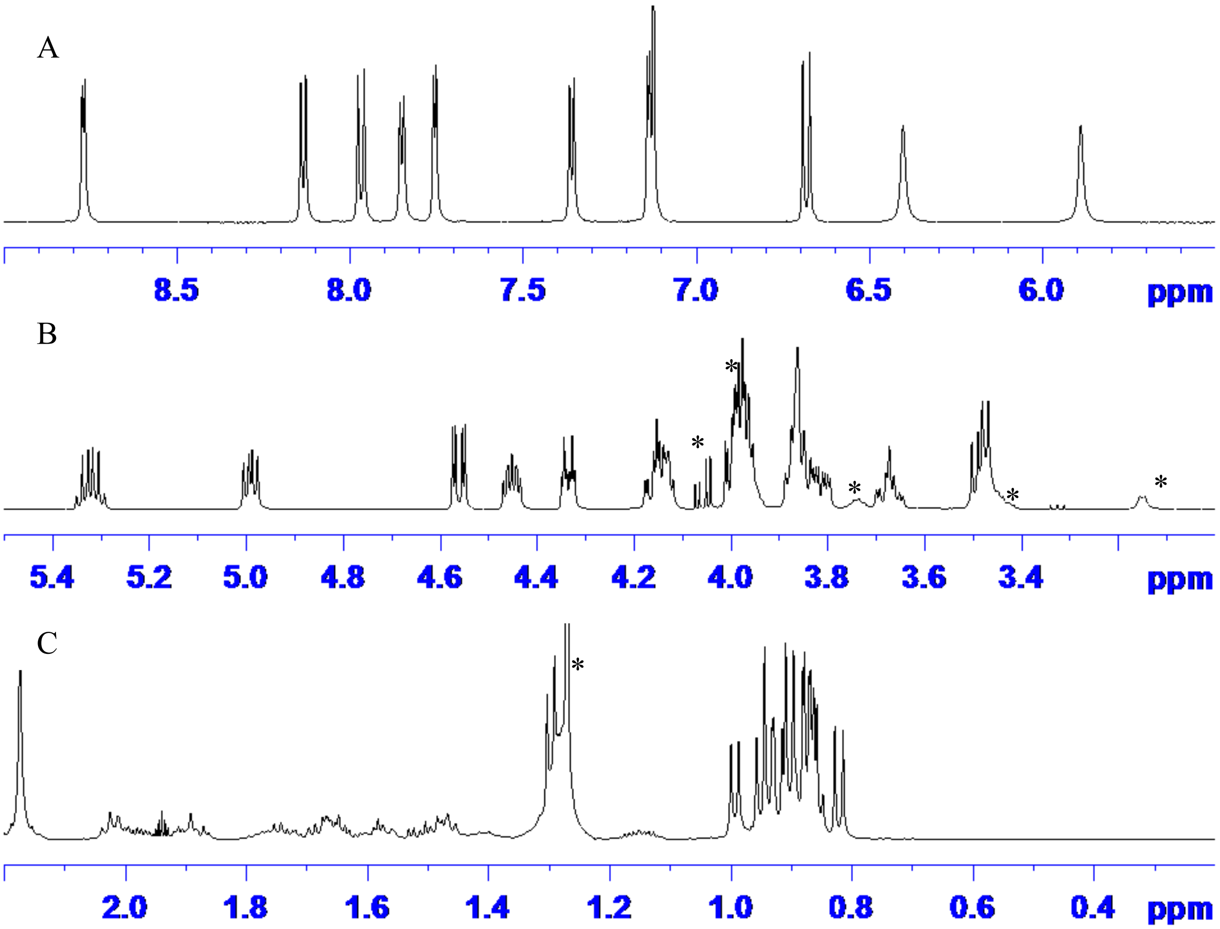

Figure S66. ^{1}H spectrum of enantiomer of pseudodesmin (**2**) (CD_3CN, 25°C, 500MHz) A) H^N region, B) H^{α} region and C) aliphatic region. Asterisks indicate the resonances of an unidentified impurity**.**

Table S3. ^{1}H and ^{13}C assignment of ***ent*-1** (**2**) (CD_3CN, 25°C, 500 MHz). Scalar couplings in Hz.

			^{1}H δ [ppm]	^{13}C δ [ppm]
**(*S*)-HDA**				
		CO		175.12
		CH2 α1	2.36	44.71
		CH2 α2	2.44	44.71
		CH β	3.96	69.48
		CH2 γ	1.47	38.15
		CH2 δ1	1.30	26.27
		CH2 δ2	1.41	26.27
		CH2 ε	1.28	30.32
		CH2 ζ	1.28	30.32
		CH2 η	1.27	32.53
		CH2 θ	1.29	23.34
		CH3 ι	0.88	14.36
		OH	3.47	
**D-Leu1**				
$^{3}J_{HNH\alpha}$	5.68	NH	7.85	
		CH α	3.86	53.80
		CO		175.25
		CH2 β1	1.65	39.29
		CH2 β2	1.74	39.29
		CH γ	1.67	25.34
		CH3 δ	0.90	22.09
		CH3 δ	0.94	23.13
**L-Gln2**				
$^{3}J_{HNH\alpha}$	3.97	NH	8.77	
		CH α	3.98	57.48
		CO		176.57
		CH2 β	2.02	26.27
		CH2 γ	2.38	31.88
		CO δ		176.08
		NH2	6.40/5.89	
**L-*allo*-Thr3**				
$^{3}J_{HNH\alpha}$	7.17	NH	8.14	
$^{3}J_{H\alpha H\beta}$	ND	CH α	4.00	61.66
		CO		174.25
		CH β	5.32	70.24
		CH3 γ	1.30	18.49
**L-Val4**				
$^{3}J_{HNH\alpha}$	6.36	NH	7.36	
		CH α	3.49	64.99
		CO		174.57
		CH β	2.17	29.90
		CH3 γ	0.92	19.46
		CH3 γ	0.95	21.00

			^{1}H δ [ppm]	^{13}C δ [ppm]
**L-Leu5**				
$^{3}J_{HNH\alpha}$	4.09	NH	7.76	
		CH α	3.98	55.64
		CO		173.55
		CH2 β1	1.50	40.57
		CH2 β2	1.66	40.57
		CH γ	1.77	25.42
		CH3 δ	0.88	21.24
		CH3 δ	0.86	23.27
**L-Ser6**				
$^{3}J_{HNH\alpha}$	ND	NH	7.13	
		CH α	4.34	56.34
		CO		171.98
		CH2 β1	3.81	64.67
		CH2 β2	4.14	64.67
		OH γ	4.99	
**D-Leu7**				
$^{3}J_{HNH\alpha}$	ND	NH	7.13	
		CH α	4.14	54.87
		CO		173.86
		CH2 β1	1.58	41.98
		CH2 β2	1.89	41.98
		CH γ	1.89	25.43
		CH3 δ	0.90	21.32
		CH3 δ	0.99	23.42
**L-Ser8**				
$^{3}J_{HNH\alpha}$	8.97	NH	7.97	
		CH α	4.45	56.94
		CO		171.79
		CH2 β1	3.67	63.11
		CH2 β2	3.86	63.11
		OH γ	not allocated	
**D-Ile9**				
$^{3}J_{HNH\alpha}$	10.11	NH	6.68	
		CH α	4.56	57.10
		CO		169.97
		CH β	1.98	36.82
		CH3 γ	0.82	16.18
		CH2 γ1	0.98	25.19
		CH2 γ2	1.14	25.19
		CH3 δ	0.86	12.25

ND= not determined

pseudodesmin L1A (3)

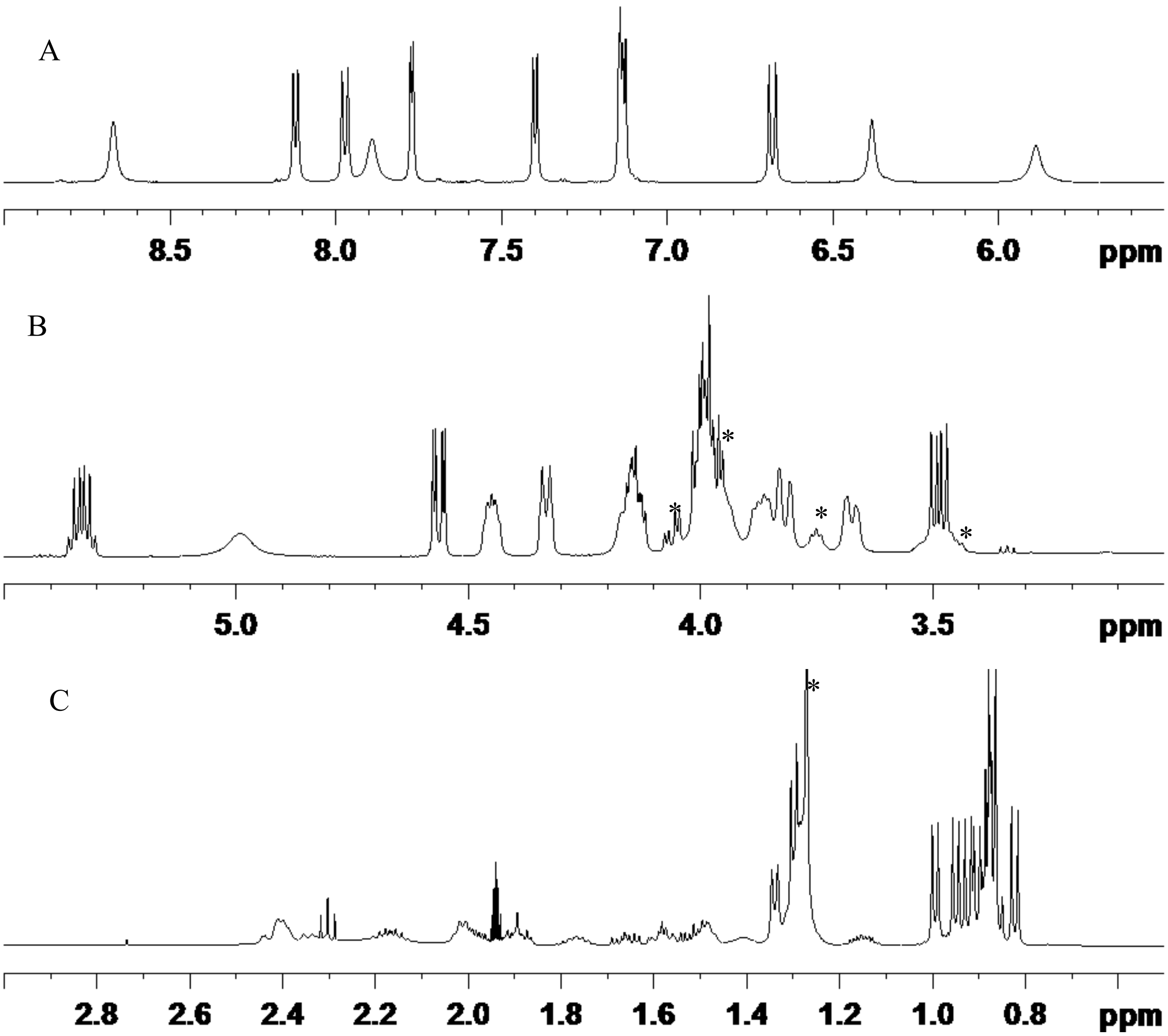

Figure S67. ^{1}H spectrum of pseudodesmin L1A (**3**) (CD_3CN, 25°C, 500MHz) A) H^N region, B) H^α region and C) aliphatic region. Asterisks indicate the resonances of an unidentified impurity.

Table S4. ^{1}H and ^{13}C assignment of synthetic **pseudodesmin L1A** (CD_3CN, 25°C, 500 MHz). Scalar couplings in Hz.

			^{1}H δ [ppm]	^{13}C δ [ppm]
**(*R*)-HDA**				
		CO		not allocated
		CH_2 α1	2.33	44.72
		CH_2 α2	2.43	44.72
		CH β	4.00	69.46
		CH_2 γ	1.49	38.22
		CH_2 δ1	1.30	26.29
		CH_2 δ2	1.41	26.29
		CH_2 ε	1.27	30.31
		CH_2 ζ	1.27	30.31
		CH_2 η	1.27	32.55
		CH_2 θ	1.28	23.33
		CH_3 ι	0.88	14.35
		OH		not allocated
**L-Ala1**				
$^{3}J_{HNH\alpha}$	ND	NH	7.89	
		CH α	3.94	51.03
		CO		175.28
		CH_2 β1	1.34	15.84
		CH_2 β2		
		CH γ		
		CH_3 δ		
		CH_3 δ		
**D-Gln2**				
$^{3}J_{HNH\alpha}$	ND	NH	8.67	
		CH α	3.99	57.39
		CO		176.60
		CH_2 β	2.01	26.32
		CH_2 γ	3.40	32.58
		CO δ		176.03
		NH_2	6.38/5.89	
**D-*allo*-Thr3**				
$^{3}J_{HNH\alpha}$	7.00	NH	8.12	
$^{3}J_{H\alpha H\beta}$	ND	CH α	3.99	61.66
		CO		174.22
		CH β	5.33	70.24
		CH_3 γ	1.30	18.50
**D-Val4**				
$^{3}J_{HNH\alpha}$	6.32	NH	7.40	
		CH α	3.49	65.02
		CO		174.57
		CH β	2.17	29.88
		CH_3 γ	0.92	19.49
		CH_3 γ	0.95	20.80
**D-Leu5**				
$^{3}J_{HNH\alpha}$	4.00	NH	7.77	
		CH α	3.97	55.66
		CO		173.54
		CH_2 β1	1.51	40.67
		CH_2 β2	1.66	40.67
		CH γ	1.77	25.49
		CH_3 δ	0.88	21.45
		CH_3 δ	0.87	23.15
**D-Ser6**				
$^{3}J_{HNH\alpha}$	4.45	NH	7.13	
		CH α	4.33	56.33
		CO		171.97
		CH_2 β1	3.82	64.66
		CH_2 β2	4.16	64.66
		OH γ	4.99	
**L-Leu7**				
$^{3}J_{HNH\alpha}$	2.23	NH	7.14	
		CH α	4.14	54.84
		CO		173.85
		CH_2 β1	1.58	41.97
		CH_2 β2	1.90	41.97
		CH γ	1.90	25.42
		CH_3 δ	0.90	21.32
		CH_3 δ	0.99	23.41
**D-Ser8**				
$^{3}J_{HNH\alpha}$	8.80	NH	7.97	
		CH α	4.45	56.93
		CO		171.82
		CH_2 β1	3.67	63.10
		CH_2 β2	3.87	63.10
		OH γ	not allocated	
**L-Ile9**				
$^{3}J_{HNH\alpha}$	10.09	NH	6.68	
		CH α	4.56	57.10
		CO		169.96
		CH β	1.98	36.82
		CH_3 γ	0.82	16.17
		CH_2 γ1	0.97	25.17
		CH_2 γ2	1.15	25.17
		CH_3 δ	0.86	12.24

ND= not determined

pseudodesmin Q2A (4)

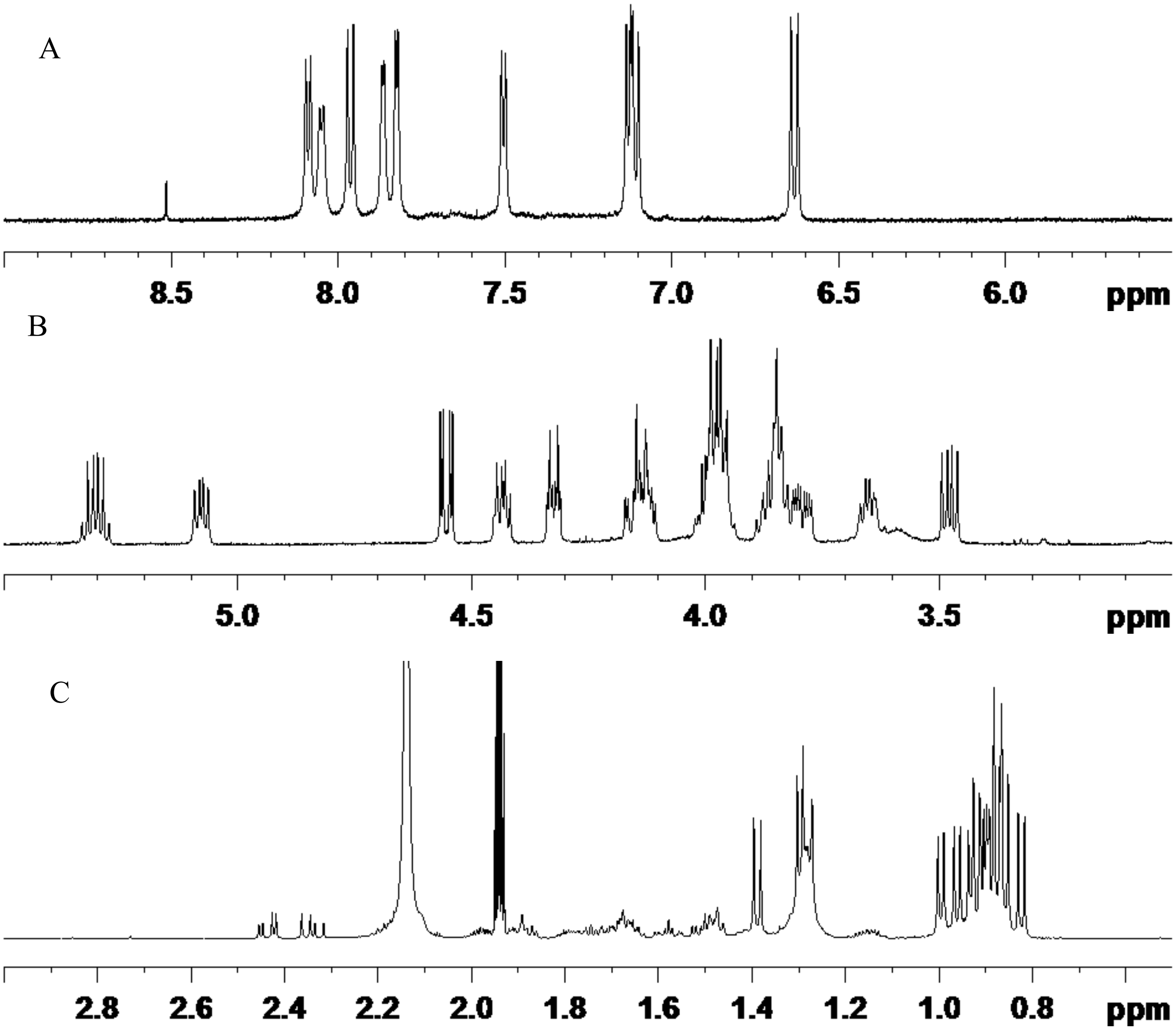

Figure S68. ^{1}H spectrum of pseudodesmin Q2A (**4**) (CD_3CN, 25°C, 500MHz) A) H^N region, B) H^{α} region and C) aliphatic region.

Table S5. ^{1}H and ^{13}C assignment of synthetic **pseudodesmin Q2A** (CD_3CN, 25°C, 500 MHz). Scalar couplings in Hz.

			^{1}H δ [ppm]	^{13}C δ [ppm]
**(*R*)-HDA**				
		CO		174.85
		CH_2 α1	2.34	44.55
		CH_2 α2	2.44	44.55
		CH β	3.96	69.74
		CH_2 γ	1.48	38.15
		CH_2 δ1	1.30	26.30
		CH_2 δ2	1.41	26.30
		CH_2 ε	1.27	30.29
		CH_2 ζ	1.27	30.29
		CH_2 η	1.28	32.51
		CH_2 θ	1.29	23.33
		CH_3 ι	0.88	14.34
		OH	3.60	
**L-Leu1**				
$^{3}J_{HNH\alpha}$		NH	8.05	
		CH α	3.84	53.38
		CO		175.12
		CH_2 β1	1.66	39.24
		CH_2 β2	1.75	39.24
		CH γ	1.68	25.31
		CH_3 δ	0.90	22.00
		CH_3 δ	0.93	23.20
**D-Ala2**				
$^{3}J_{HNH\alpha}$	3.60	NH	7.87	
		CH α	3.99	53.41
		CO		177.94
		CH_3 β	1.39	16.92
**D-*allo*-Thr3**				
$^{3}J_{HNH\alpha}$	7.18	NH	8.09	
$^{3}J_{H\alpha H\beta}$	ND	CH α	3.97	61.70
		CO		174.28
		CH β	5.30	70.22
		CH_3 γ	1.30	18.48
**D-Val4**				
$^{3}J_{HNH\alpha}$	6.06	NH	7.50	
		CH α	3.48	65.14
		CO		174.59
		CH β	2.17	29.90
		CH_3 γ	0.92	19.45
		CH_3 γ	0.96	20.99
**D-Leu5**				
$^{3}J_{HNH\alpha}$	4.08	NH	7.82	
		CH α	3.98	55.62
		CO		173.50
		CH_2 β1	1.50	40.59
		CH_2 β2	1.68	40.59
		CH γ	1.78	25.47
		CH_3 δ	0.88	21.20
		CH_3 δ	0.86	23.25
**D-Ser6**				
$^{3}J_{HNH\alpha}$	8.50	NH	7.11	
		CH α	4.32	56.29
		CO		171.88
		CH_2 β1	3.79	64.66
		CH_2 β2	4.15	64.66
		OH γ	5.08	
**L-Leu7**				
$^{3}J_{HNH\alpha}$	6.21	NH	7.13	
		CH α	4.13	54.85
		CO		173.79
		CH_2 β1	1.58	42.00
		CH_2 β2	1.89	41.99
		CH γ	1.89	25.44
		CH_3 δ	0.90	21.32
		CH_3 δ	1.00	23.42
**D-Ser8**				
$^{3}J_{HNH\alpha}$	9.04	NH	7.96	
		CH α	4.43	56.83
		CO		171.86
		CH_2 β1	3.65	63.12
		CH_2 β2	3.86	63.12
		OH γ	not allocated	
**L-Ile9**				
$^{3}J_{HNH\alpha}$	10.08	NH	6.63	
		CH α	4.55	57.16
		CO		169.97
		CH β	1.98	36.81
		CH_3 γ	0.82	16.17
		CH_2 γ1	0.98	25.19
		CH_2 γ2	1.15	25.19
		CH_3 δ	0.87	12.25

ND= not determined

pseudodesmin V4A (5)

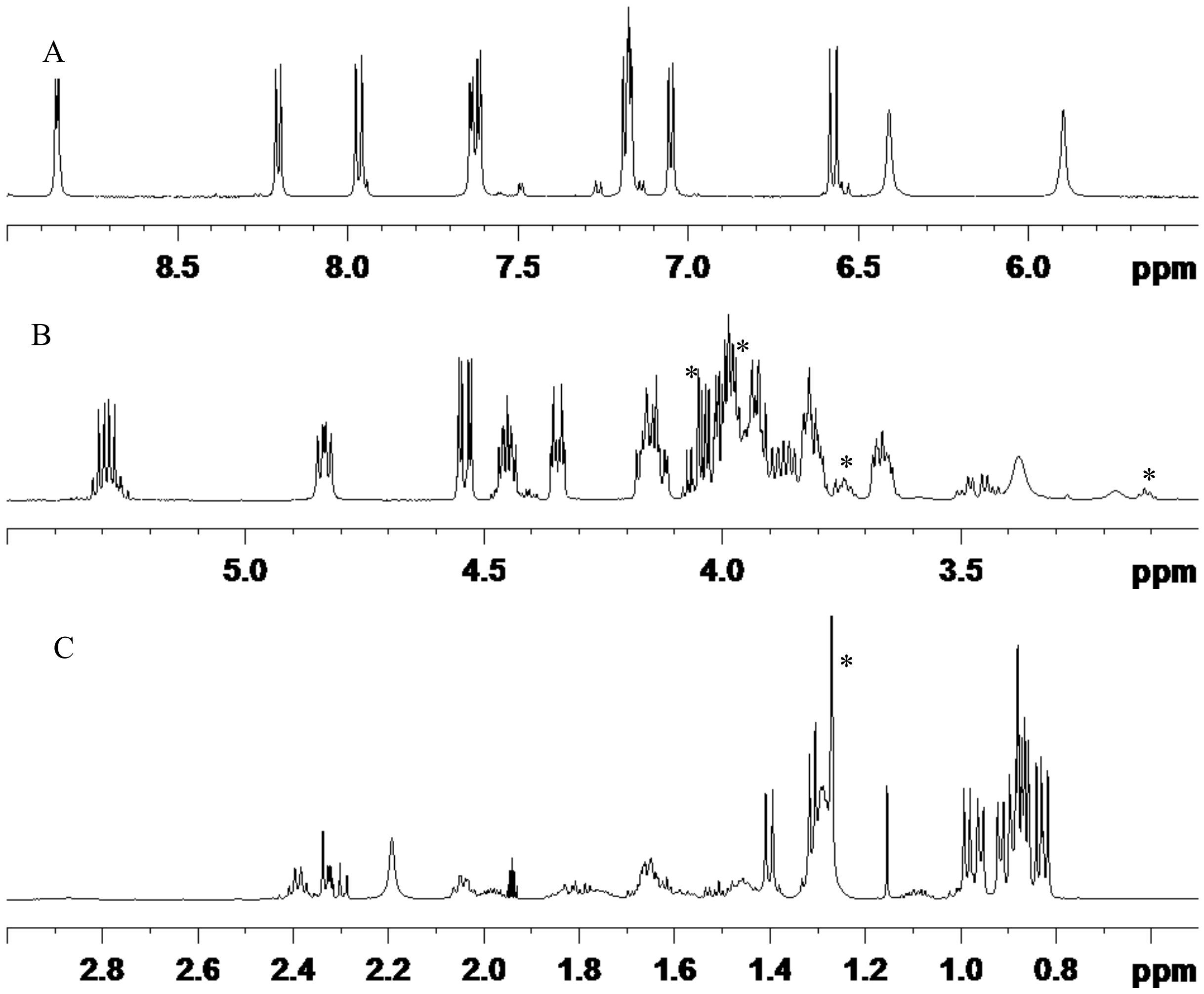

Figure S69. ^{1}H spectrum of pseudodesmin V4A (**5**) (CD_3CN, 25°C, 500MHz) A) H^N region, B) H^α region and C) aliphatic region. Asterisks indicate the resonances of an unidentified impurity.

Table S6. ^{1}H and ^{13}C assignment of synthetic **pseudodesmin V4A** (CD_3CN, 25°C, 500 MHz). Scalar couplings in Hz.

			^{1}H δ [ppm]	^{13}C δ [ppm]
**(*R*)-HDA**				
		CO		175.09
		CH_2 α1	2.30	44.52
		CH_2 α2	2.33	44.52
		CH β	3.97	69.43
		CH_2 γ	1.46	38.32
		CH_2 δ1	1.30	26.21
		CH_2 δ2	1.42	26.21
		CH_2 ε	1.27	30.32
		CH_2 ζ	1.27	30.32
		CH_2 η	1.28	32.58
		CH_2 θ	1.29	23.35
		CH_3 ι	0.88	14.36
		OH	3.38	
**L-Leu1**				
$^{3}J_{HNH\alpha}$	4.30	NH	7.64	
		CH α	3.93	54.40
		CO		175.73
		CH_2 β1	1.66	39.79
		CH_2 β2	1.66	39.79
		CH γ	1.65	25.39
		CH_3 δ	0.92	22.46
		CH_3 δ	0.96	22.83
**D-Gln2**				
$^{3}J_{HNH\alpha}$	4.13	NH	8.85	
		CH α	3.98	57.45
		CO		176.62
		CH_2 β	2.04	26.27
		CH_2 γ	2.39	31.92
		CO δ		175.90
		NH_2	6.41/5.90	
**D-*allo*-Thr3**				
$^{3}J_{HNH\alpha}$	7.16	NH	8.20	
$^{3}J_{H\alpha H\beta}$	ND	CH α	4.03	61.65
		CO		174.20
		CH β	5.29	70.50
		CH_3 γ	1.31	18.47
**D-Ala4**				
$^{3}J_{HNH\alpha}$	3.64	NH	7.17	
		CH α	3.92	53.51
		CO		175.64
		CH_3 β	1.40	16.47
**D-Leu5**				
$^{3}J_{HNH\alpha}$	4.63	NH	7.61	
		CH α	4.00	55.29
		CO		173.61
		CH_2 β1	1.50	40.76
		CH_2 β2	1.67	40.76
		CH γ	1.76	25.32
		CH_3 δ	0.87	21.28
		CH_3 δ	0.86	23.30
**D-Ser6**				
$^{3}J_{HNH\alpha}$	5.87	NH	7.19	
		CH α	4.35	56.32
		CO		171.89
		CH_2 β1	3.81	64.53
		CH_2 β2	4.14	64.53
		OH γ	4.84	
**L-Leu7**				
$^{3}J_{HNH\alpha}$	6.51	NH	7.05	
		CH α	4.15	54.67
		CO		173.84
		CH_2 β1	1.61	41.91
		CH_2 β2	1.81	41.91
		CH γ	1.83	25.42
		CH_3 δ	0.89	21.48
		CH_3 δ	0.99	23.34
**D-Ser8**				
$^{3}J_{HNH\alpha}$	8.93	NH	7.97	
		CH α	4.45	56.81
		CO		171.89
		CH_2 β1	3.67	63.09
		CH_2 β2	3.87	63.09
		OH γ	3.11	
**L-Ile9**				
$^{3}J_{HNH\alpha}$	10.09	NH	6.57	
		CH α	4.54	57.23
		CO		170.11
		CH β	1.98	37.11
		CH_3 γ	0.82	16.19
		CH_2 γ1	1.00	25.15
		CH_2 γ2	1.10	25.15
		CH_3 δ	0.84	12.10

ND= not determined

pseudodesmin L5A (6)

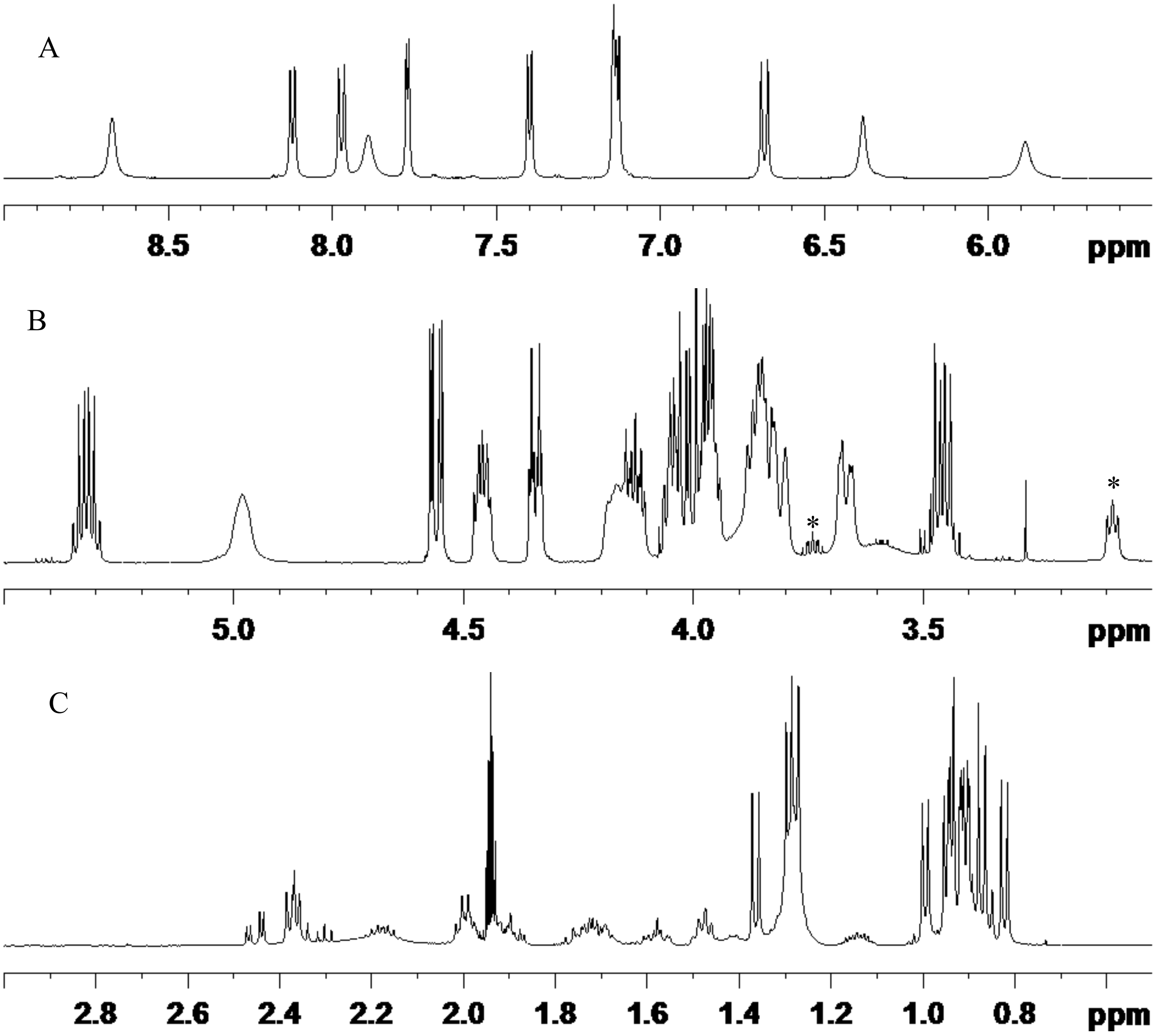

Figure S70. ^{1}H spectrum of pseudodesmin L5A (**6**) (CD_3CN, 25°C, 500MHz) A) H^N region, B) H^α region and C) aliphatic region. Asterisks indicate the resonances of an unidentified impurity.

Table S7. ^{1}H and ^{13}C assignment of synthetic **pseudodesmin L5A** (CD_3CN, 25°C, 500 MHz). Scalar couplings in Hz.

			^{1}H δ [ppm]	^{13}C δ [ppm]
**(*R*)-HDA**				
		CO		175.14
		CH_2 α1	2.36	44.78
		CH_2 α2	2.46	44.78
		CH β	3.97	69.52
		CH_2 γ	1.48	38.16
		CH_2 δ1	1.30	26.30
		CH_2 δ2	1.41	26.30
		CH_2 ε	1.28	30.28
		CH_2 ζ	1.28	30.28
		CH_2 η	1.27	32.55
		CH_2 θ	1.28	23.34
		CH_3 ι	0.88	14.36
		OH	not allocated	
**L-Leu1**				
$^{3}J_{HNH\alpha}$	6.23	NH	8.01	
		CH α	3.85	53.71
		CO		175.18
		CH_2 β1	1.73	38.75
		CH_2 β2	1.73	38.75
		CH γ	1.70	25.26
		CH_3 δ	0.90	21.61
		CH_3 δ	0.94	23.59
**D-Gln2**				
$^{3}J_{HNH\alpha}$	4.26	NH	8.79	
		CH α	4.05	57.26
		CO		176.68
		CH_2 β	2.00	26.34
		CH_2 γ	2.37	31.88
		CO δ		176.22
		NH_2	6.45/5.91	
**D-*allo*-Thr3**				
$^{3}J_{HNH\alpha}$	7.28	NH	8.11	
$^{3}J_{H\alpha H\beta}$	10.72	CH α	4.01	61.62
		CO		174.38
		CH β	5.32	70.25
		CH_3 γ	1.29	18.50
**D-Val4**				
$^{3}J_{HNH\alpha}$				
	6.25	NH	7.42	
		CH α	3.46	65.08
		CO		174.57
		CH β	2.18	29.77
		CH_3 γ	0.93	19.53
		CH_3 γ	0.95	20.99
**D-Ala5**				
$^{3}J_{HNH\alpha}$	3.40	NH	7.94	
		CH α	3.97	52.96
		CO		173.87
		CH_3 β	1.36	17.28
**D-Ser6**				
$^{3}J_{HNH\alpha}$	8.53	NH	7.07	
		CH α	4.34	56.02
		CO		171.99
		CH_2 β1	3.81	64.72
		CH_2 β2	4.17	64.72
		OH γ	4.98	
**L-Leu7**				
$^{3}J_{HNH\alpha}$	6.02	NH	7.20	
		CH α	4.13	54.98
		CO		173.89
		CH_2 β1	1.58	42.01
		CH_2 β2	1.90	42.01
		CH γ	1.91	25.43
		CH_3 δ	0.91	21.32
		CH_3 δ	1.00	23.43
**D-Ser8**				
$^{3}J_{HNH\alpha}$	9.02	NH	7.98	
		CH α	4.46	56.88
		CO		171.84
		CH_2 β1	3.67	63.13
		CH_2 β2	3.87	63.13
		OH γ	not allocated	
**L-Ile9**				
$^{3}J_{HNH\alpha}$	10.13	NH	6.62	
		CH α	4.56	57.15
		CO		169.96
		CH β	1.99	36.81
		CH_3 γ	0.82	16.19
		CH_2 γ1	0.98	25.18
		CH_2 γ2	1.14	25.18
		CH_3 δ	0.86	12.27

ND= not determined

pseudodesmin S6A (7)

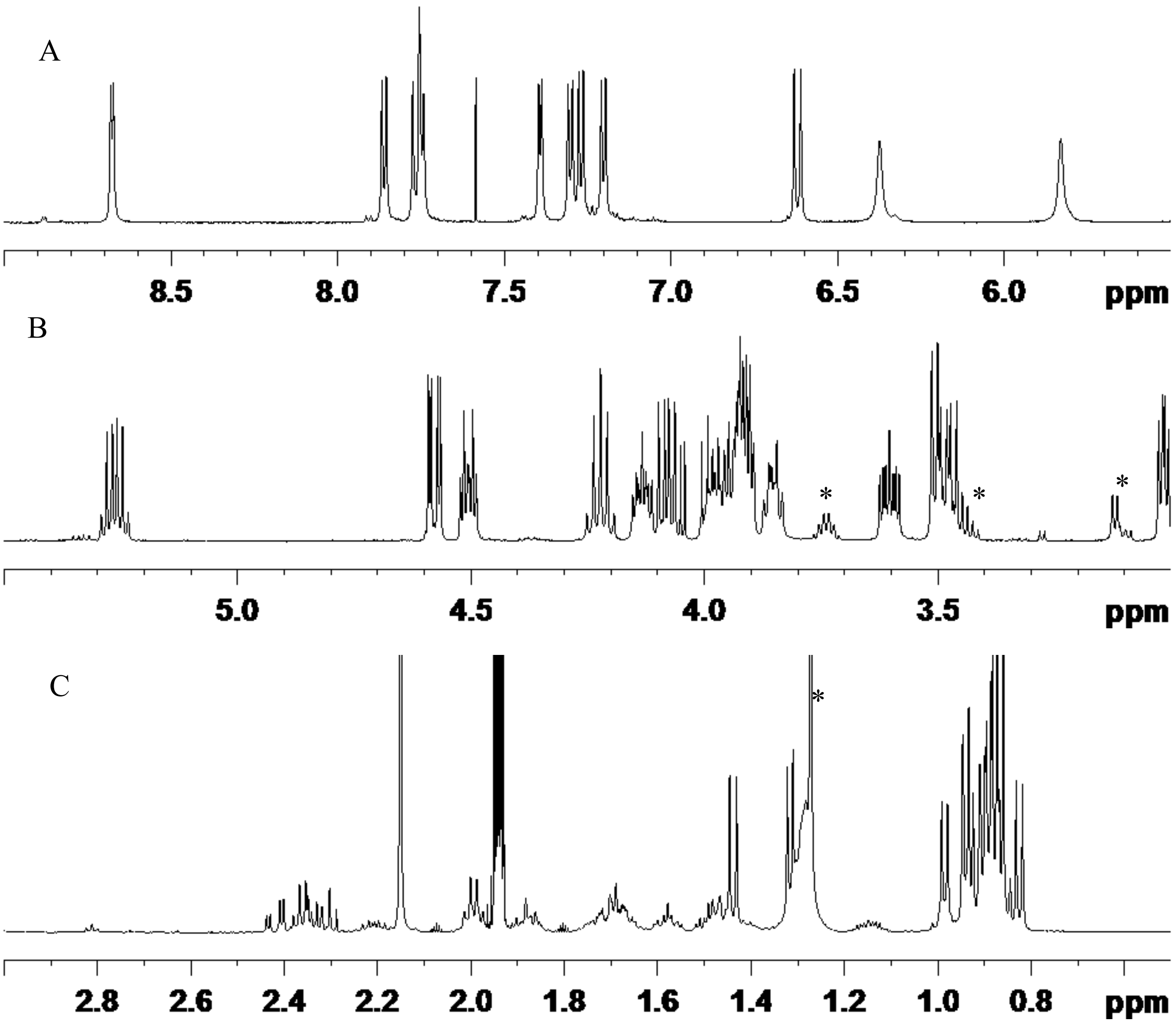

Figure S71. ^{1}H spectrum of pseudodesmin S6A (**7**) (CD_3CN, 25°C, 500MHz) A) H^N region, B) H^{α} region and C) aliphatic region. Asterisks indicate the resonances of an unidentified impurity.

Table S8. ^{1}H and ^{13}C assignment of synthetic **pseudodesmin S6A** (CD_3CN, 25°C, 500 MHz). Scalar couplings in Hz.

			^{1}H δ [ppm]	^{13}C δ [ppm]				^{1}H δ [ppm]	^{13}C δ [ppm]
**(*R*)-HDA**					**D-Leu5**				
		CO		174.88	$^{3}J_{HNH\alpha}$	4.56	NH	7.39	
		CH_2 α1	2.33	44.64			CH α	3.94	55.72
		CH_2 α2	2.42	44.64			CO		173.89
		CH β	3.98	69.54			CH_2 β1	1.49	40.35
		CH_2 γ	1.47	38.24			CH_2 β2	1.70	40.35
		CH_2 δ1	1.30	26.24			CH γ	1.73	25.39
		CH_2 δ2	1.41	26.24			CH_3 δ	0.86	21.38
		CH_2 ε	1.27	30.30			CH_3 δ	0.88	23.26
		CH_2 ζ	1.27	30.30	**D-Ala6**				
		CH_2 η	1.28	32.55	$^{3}J_{HNH\alpha}$	6.87	NH	7.27	
		CH_2 θ	1.28	23.34			CH α	4.22	50.30
		CH_3 ι	0.88	14.36			CO		174.84
		OH	3.51				CH3 β	1.44	18.18
**L-Leu1**									
$^{3}J_{HNH\alpha}$	5.74	NH	7.75						
		CH α	3.85	53.88	**L-Leu7**				
		CO		175.39	$^{3}J_{HNH\alpha}$	6.40	NH	7.20	
		CH_2 β1	1.69	39.12			CH α	4.13	54.46
		CH_2 β2	1.69	39.12			CO		173.87
		CH γ	1.68	25.37			CH_2 β1	1.58	41.82
		CH_3 δ	0.90	22.02			CH_2 β2	1.88	41.82
		CH_3 δ	0.94	23.18			CH γ	1.87	25.41
**D-Gln2**							CH_3 δ	0.89	21.49
$^{3}J_{HNH\alpha}$	3.96	NH	8.68				CH_3 δ	0.99	23.37
		CH α	3.91	57.72	**D-Ser8**				
		CO		175.19	$^{3}J_{HNH\alpha}$	9.46	NH	7.77	
		CH_2 β	1.99	26.48			CH α	4.51	55.78
		CH_2 γ	2.36	32.03			CO		171.75
		CO δ		176.00			CH_2 β1	3.60	63.63
		NH_2	6.37/5.83				CH_2 β2	3.91	63.63
**D-*allo*-Thr3**							OH γ	3.02	
$^{3}J_{HNH\alpha}$	6.63	NH	7.86		**L-Ile9**				
$^{3}J_{H\alpha H\beta}$	10.83	CH α	4.08	61.68	$^{3}J_{HNH\alpha}$	10.00	NH	6.62	
		CO		174.71			CH α	4.58	57.17
		CH β	5.26	70.31			CO		169.97
		CH_3 γ	1.32	18.80			CH β	1.99	37.09
**D-Val4**							CH_3 γ	0.83	16.17
$^{3}J_{HNH\alpha}$	6.65	NH	7.30				CH_2 γ1	0.99	25.13
		CH α	3.48	64.71			CH_2 γ2	1.15	25.13
		CO		174.20			CH_3 δ	0.86	12.27
		CH β	2.21	29.72					
		CH_3 γ	0.92	19.49					
		CH_3 γ	0.94	21.11					

ND= not determined

pseudodesmin L7A (8)

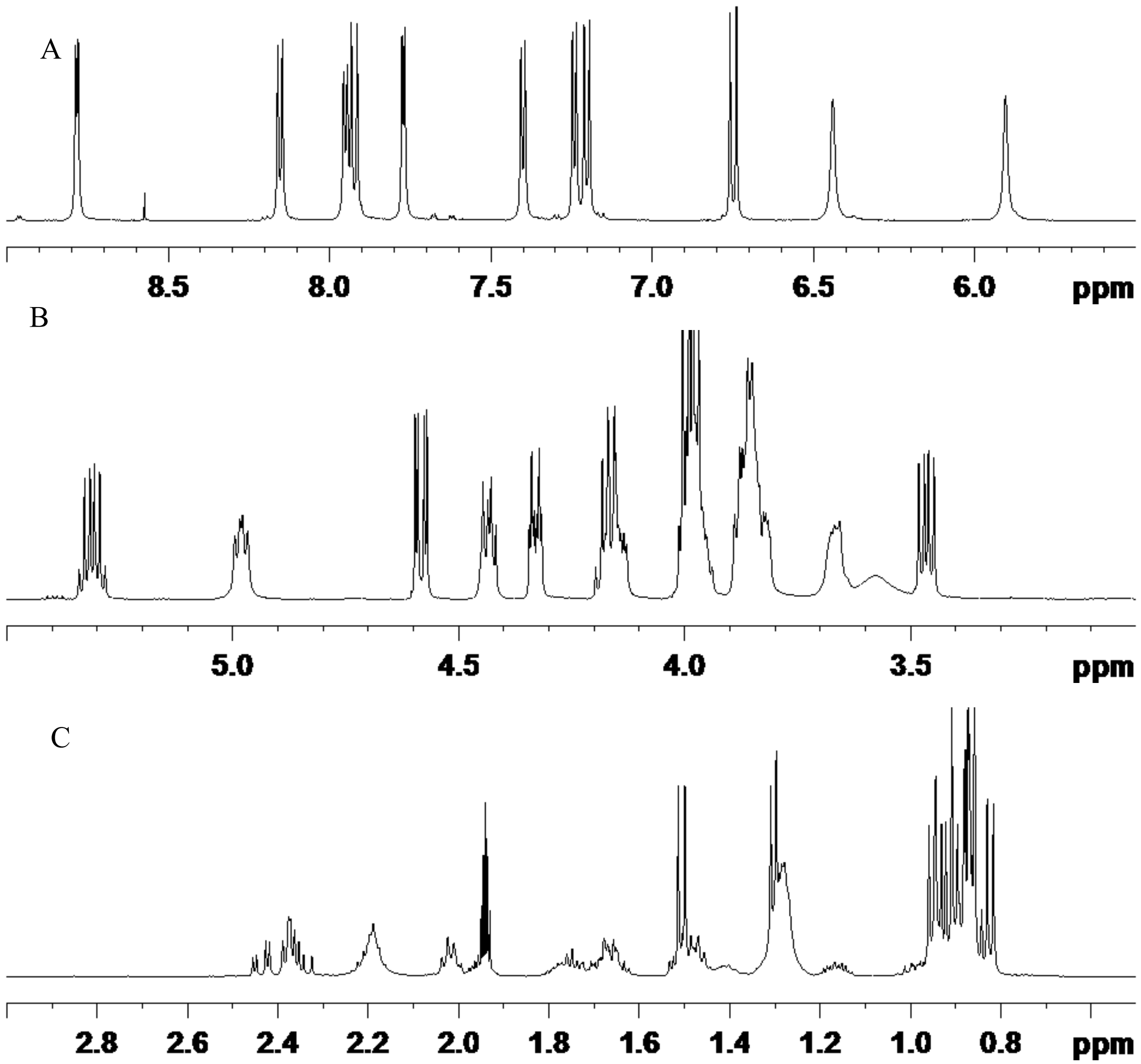

Figure S72. ^{1}H spectrum of pseudodesmin L7A (**8**) (CD_3CN, 25°C, 500MHz) A) H^N region, B) H^α region and C) aliphatic region.

Table S9. ^{1}H and ^{13}C assignment of synthetic **pseudodesmin L7A** (CD_3CN, 25°C, 500 MHz). Scalar couplings in Hz.

			^{1}H δ [ppm]	^{13}C δ [ppm]
**(*R*)-HDA**				
		CO		175.08
		CH_2 α1	2.35	44.71
		CH_2 α2	2.44	44.71
		CH β	3.96	69.52
		CH_2 γ	1.47	38.17
		CH_2 δ1	1.30	26.29
		CH_2 δ2	1.41	26.29
		CH_2 ε	1.29	30.21
		CH_2 ζ	1.29	29.98
		CH_2 η	1.27	32.54
		CH_2 θ	1.28	23.34
		CH_3 ι	0.88	14.37
		OH	not allocated	
**L-Leu1**				
$^{3}J_{HNH\alpha}$	5.79	NH	7.95	
		CH α	3.87	53.75
		CO		175.28
		CH_2 β1	1.66	39.27
		CH_2 β2	1.75	39.27
		CH γ	1.67	25.35
		CH_3 δ	0.91	22.09
		CH_3 δ	0.94	23.15
**D-Gln2**				
$^{3}J_{HNH\alpha}$	4.02	NH	8.78	
		CH α	3.99	57.50
		CO		176.65
		CH_2 β	2.02	26.36
		CH_2 γ	2.38	31.92
		CO δ		176.05
		NH_2	6.44/5.91	
**D-*allo*-Thr3**				
$^{3}J_{HNH\alpha}$	7.18	NH	8.15	
$^{3}J_{H\alpha H\beta}$	10.75	CH α	3.99	61.67
		CO		174.19
		CH β	5.31	70.19
		CH_3 γ	1.30	18.55
**D-Val4**				
$^{3}J_{HNH\alpha}$	6.24	NH	7.40	
		CH α	3.46	65.14
		CO		174.75
		CH β	2.20	29.98
		CH_3 γ	0.92	19.35
		CH_3 γ	0.96	20.99
**D-Leu5**				
$^{3}J_{HNH\alpha}$	4.18	NH	7.77	
		CH α	3.99	55.66
		CO		173.71
		CH_2 β1	1.51	40.53
		CH_2 β2	1.68	40.53
		CH γ	1.78	25.46
		CH_3 δ	0.88	21.19
		CH_3 δ	0.87	23.28
**D-Ser6**				
$^{3}J_{HNH\alpha}$	8.26	NH	7.20	
		CH α	4.33	56.40
		CO		171.59
		CH_2 β1	3.83	64.44
		CH_2 β2	4.15	64.44
		OH γ	4.98	
**L-Ala7**				
$^{3}J_{HNH\alpha}$	5.94	NH	7.24	
		CH α	4.17	52.00
		CO		
		CH_3 β	1.51	17.96
**D-Ser8**				
$^{3}J_{HNH\alpha}$	8.99	NH	7.92	
		CH α	4.43	56.90
		CO		171.68
		CH_2 β1	3.66	63.26
		CH_2 β2	3.86	63.26
		OH γ	not allocated	
**L-Ile9**				
$^{3}J_{HNH\alpha}$	9.99	NH	6.75	
		CH α	4.58	56.95
		CO		169.96
		CH β	1.95	37.12
		CH_3 γ	0.82	16.22
		CH_2 γ1	0.99	25.35
		CH_2 γ2	1.17	25.35
		CH_3 δ	0.86	12.38

ND= not determined

pseudodesmin S8A (9)

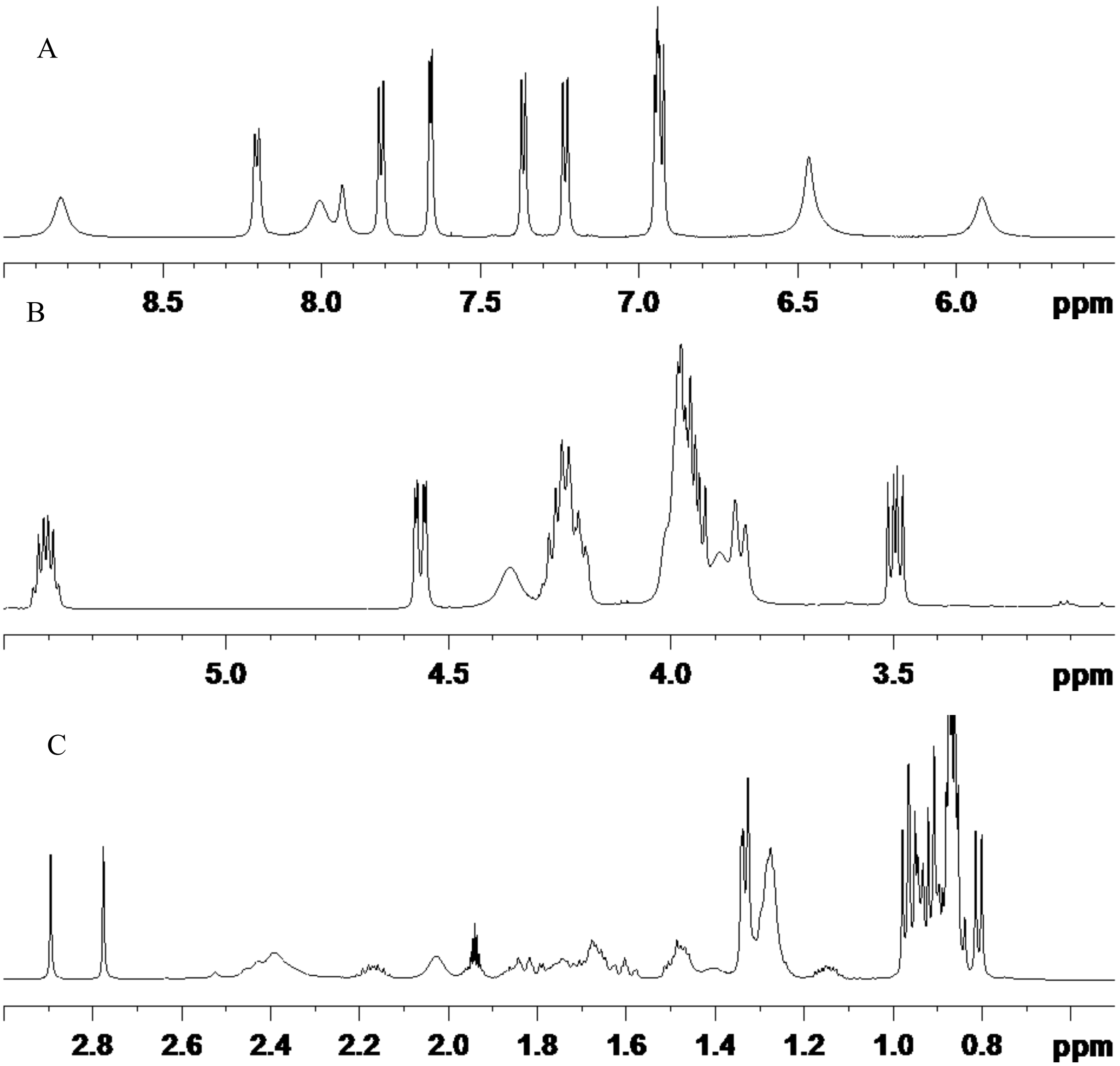

Figure S73. ^{1}H spectrum of pseudodesmin S8A (**9**) (CD_3CN, 25°C, 500MHz) A) H^N region, B) H^{α} region and C) aliphatic region.

Table S10. ^{1}H and ^{13}C assignment of synthetic **pseudodesmin S8A** (CD_3CN, 25°C, 500 MHz). Scalar couplings in Hz.

			^{1}H δ [ppm]	^{13}C δ [ppm]
**(*R*)-HDA**				
		CO		not allocated
		CH_2 α1	2.36	44.68
		CH_2 α2	2.44	44.68
		CH β	3.98	69.58
		CH_2 γ	1.48	38.20
		CH_2 δ1	1.30	26.27
		CH_2 δ2	1.41	26.27
		CH_2 ε	1.28	30.22
		CH_2 ζ	1.28	29.98
		CH_2 η	1.27	32.54
		CH_2 θ	1.28	23.34
		CH_3 ι	0.88	14.37
		OH		not allocated
**L-Leu1**				
$^3J_{HNH\alpha}$	ND	NH	8.01	
		CH α	3.89	53.79
		CO		175.46
		CH_2 β1	1.67	39.31
		CH_2 β2	1.74	39.31
		CH γ	1.67	25.36
		CH_3 δ	0.90	22.12
		CH_3 δ	0.94	23.15
**D-Gln2**				
$^3J_{HNH\alpha}$	ND	NH	8.82	
		CH α	3.98	57.55
		CO		176.43
		CH_2 β	2.03	26.49
		CH_2 γ	2.39	32.07
		CO δ		not allocated
		NH_2	6.47/5.92	
**D-*allo*-Thr3**				
$^3J_{HNH\alpha}$	6.79	NH	8.20	
$^3J_{H\alpha H\beta}$	10.70	CH α	3.94	62.26
		CO		174.27
		CH β	5.40	70.12
		CH_3 γ	1.33	18.67
**D-Val4**				
$^3J_{HNH\alpha}$	6.58	NH	7.36	
		CH α	3.50	64.70
		CO		174.07
		CH β	2.17	29.91
		CH_3 γ	0.92	19.50
		CH_3 γ	0.96	21.01

			^{1}H δ [ppm]	^{13}C δ [ppm]
**D-Leu5**				
$^3J_{HNH\alpha}$	4.29	NH	7.66	
		CH α	3.97	55.61
		CO		173.96
		CH_2 β1	1.49	40.62
		CH_2 β2	1.68	40.62
		CH γ	1.76	25.42
		CH_3 δ	0.87	21.27
		CH_3 δ	0.86	23.28
**D-Ser6**				
$^3J_{HNH\alpha}$	7.40	NH	7.23	
		CH α	4.24	57.13
		CO		172.19
		CH_2 β1	3.84	64.25
		CH_2 β2	4.00	64.25
		OH γ	4.36	
**L-Leu7**				
$^3J_{HNH\alpha}$	7.43	NH	6.94	
		CH α	4.20	53.76
		CO		173.26
		CH_2 β1	1.60	41.89
		CH_2 β2	1.82	41.89
		CH γ	1.84	25.28
		CH_3 δ	0.87	21.08
		CH_3 δ	0.97	23.59
**D-Ala8**				
$^3J_{HNH\alpha}$	7.13	NH	7.81	
		CH α	4.26	51.06
		CO		173.96
		CH_3 β	1.34	18.37
**L-Ile9**				
$^3J_{HNH\alpha}$	9.86	NH	6.93	
		CH α	4.56	56.97
		CO		170.17
		CH β	1.95	37.24
		CH_3 γ	0.81	16.06
		CH_2 γ1	0.93	25.10
		CH_2 γ2	1.15	25.10
		CH_3 δ	0.86	12.17

pseudodesmin I9A (10)

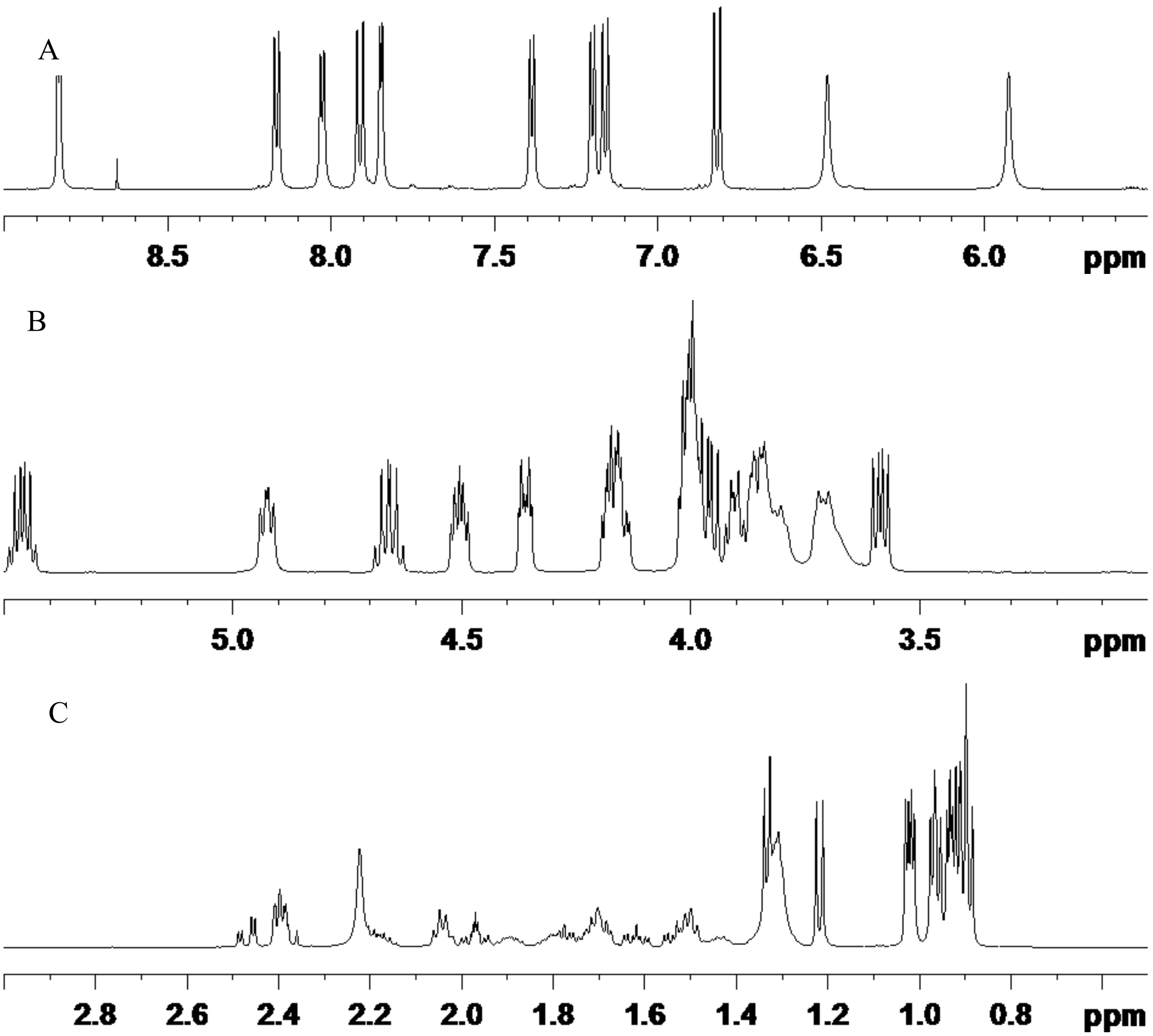

Figure S74. ^{1}H spectrum of pseudodesmin I9A (**10**) (CD_3CN, 25°C, 500MHz) A) H^N region, B) H^α region and C) aliphatic region.

Table S11. ^{1}H and ^{13}C assignment of synthetic **pseudodesmin I9A** (CD_3CN, 25°C, 500 MHz). Scalar couplings in Hz.

				^{1}H δ [ppm]	^{13}C δ [ppm]
**(*R*)-HDA**					
			CO		175.08
			CH_2 α1	2.36	44.69
			CH_2 α2	2.44	44.69
			CH β	3.97	69.51
			CH_2 γ	1.48	38.19
			CH_2 δ1	1.29	26.24
			CH_2 δ2	1.41	26.24
			CH_2 ε	1.30	30.19
			CH_2 ζ	1.28	29.97
			CH_2 η	1.28	32.52
			CH_2 θ	1.28	23.33
			CH_3 ι	0.88	14.36
			OH	3.65	
**L-Leu1**					
	$^{3}J_{HNH\alpha}$	5.67	NH	8.00	
			CH α	3.87	53.78
			CO		175.30
			CH_2 β1	1.68	39.31
			CH_2 β2	1.75	39.31
			CH γ	1.67	25.35
			CH_3 δ	0.91	22.10
			CH_3 δ	0.95	23.14
**D-Gln2**					
	$^{3}J_{HNH\alpha}$	4.03	NH	8.80	
			CH α	3.97	57.49
			CO		176.44
			CH_2 β	2.01	26.41
			CH_2 γ	2.37	31.96
			CO δ		176.02
			NH_2	6.45/5.90	
**D-*allo*-Thr3**					
	$^{3}J_{HNH\alpha}$	6.97	NH	8.14	
	$^{3}J_{H\alpha H\beta}$	10.59	CH α	3.93	61.78
			CO		174.11
			CH β	5.43	69.73
			CH_3 γ	1.30	18.57
**D-Val4**					
	$^{3}J_{HNH\alpha}$	6.25	NH	7.36	
			CH α	3.55	64.82
			CO		174.95
			CH β	2.15	30.01
			CH_3 γ	0.93	19.37
			CH_3 γ	0.98	20.98
**D-Leu5**					
	$^{3}J_{HNH\alpha}$	4.18	NH	7.82	
			CH α	3.98	55.59
			CO		173.66
			CH_2 β1	1.50	40.50
			CH_2 β2	1.67	40.50
			CH γ	1.77	25.43
			CH_3 δ	0.88	21.20
			CH_3 δ	0.86	23.29
**D-Ser6**					
	$^{3}J_{HNH\alpha}$	8.32	NH	7.13	
			CH α	4.33	56.43
			CO		172.12
			CH_2 β1	3.82	64.32
			CH_2 β2	4.13	64.32
			OH γ	4.90	
**L-Leu7**					
	$^{3}J_{HNH\alpha}$	6.24	NH	7.17	
			CH α	4.14	54.89
			CO		174.08
			CH_2 β1	1.59	41.14
			CH_2 β2	1.94	41.14
			CH γ	1.87	25.48
			CH_3 δ	0.89	21.41
			CH_3 δ	1.00	23.40
**D-Ser8**					
	$^{3}J_{HNH\alpha}$	9.24	NH	7.88	
			CH α	4.47	56.59
			CO		171.04
			CH_2 β1	3.68	63.36
			CH_2 β2	3.81	63.36
			OH γ	not allocated	
**L-Ala9**					
	$^{3}J_{HNH\alpha}$	9.45	NH	6.79	
			CH α	4.63	47.76
			CO		171.31
			CH3 β	1.19	17.36

ND= not determined

WLIP (11)

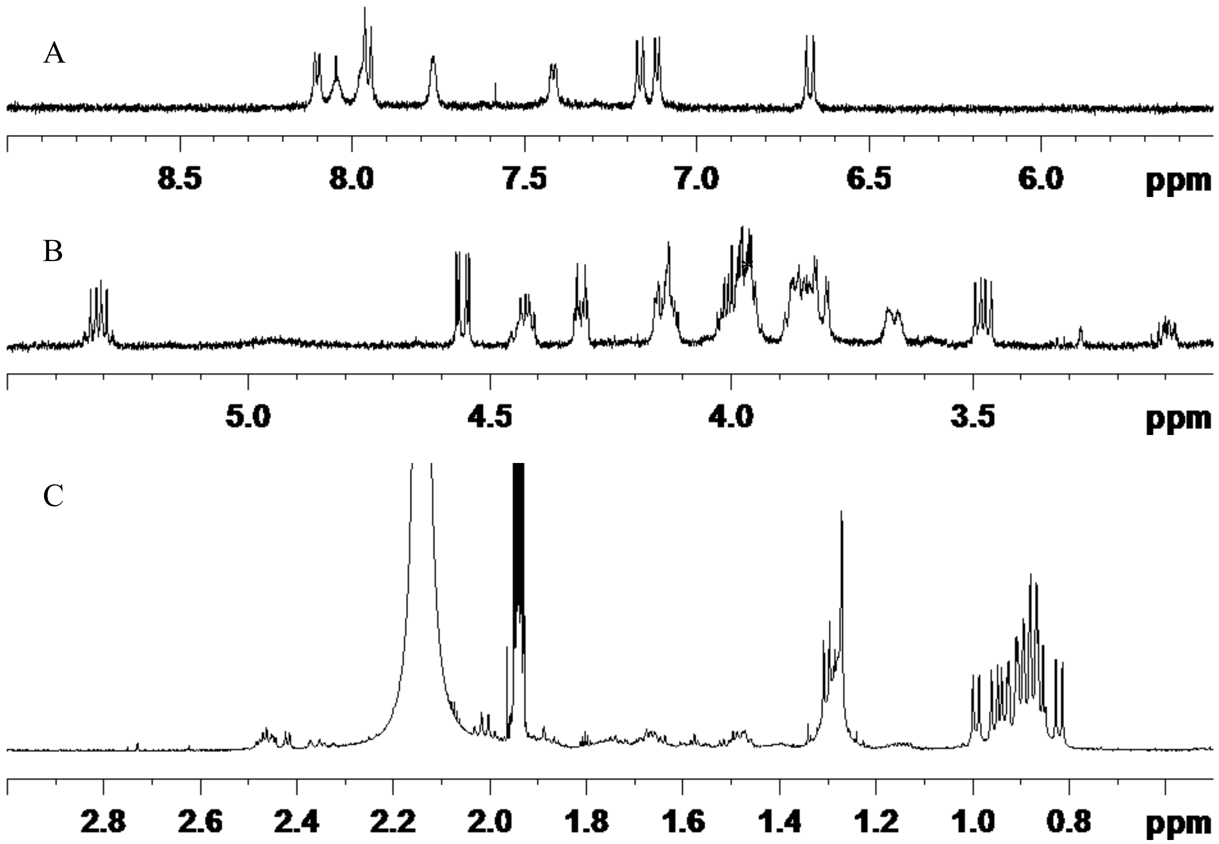

Figure S75. ^{1}H spectrum of WLIP (**11**) (CD_3CN, 25°C, 500MHz) A) H^N region, B) H^{α} region and C) aliphatic region.

Table S12. ^{1}H and ^{13}C assignment of **WLIP** (CD_3CN, 25°C, 500 MHz). Scalar couplings in Hz.

			^{1}H δ [ppm]	^{13}C δ [ppm]
**(*R*)-HDA**				
		CO		not allocated
		CH_2 α1	2.35	44.58
		CH_2 α2	2.42	44.58
		CH β	3.96	69.67
		CH_2 γ	1.48	38.15
		CH_2 δ1	1.31	26.29
		CH_2 δ2	1.41	26.29
		CH_2 ε	1.28	30.20
		CH_2 ζ	1.28	30.20
		CH_2 η	1.27	32.55
		CH_2 θ	1.28	23.33
		CH_3 ι	0.88	14.36
		OH	not allocated	
**L-Leu1**				
$^3J_{HNH\alpha}$	ND	NH	7.97	
		CH α	3.87	53.51
		CO		not allocated
		CH_2 β1	1.66	39.20
		CH_2 β2	1.73	39.20
		CH γ	1.67	25.34
		CH_3 δ	0.90	22.02
		CH_3 δ	0.93	23.19
**D-Glu2**				
$^3J_{HNH\alpha}$	ND	NH	8.05	
		CH α	4.01	57.04
		CO		not allocated
		CH_2 β	2.01	26.27
		CH_2 γ	2.47	30.72
		CO δ		not allocated
		COOH	not allocated	
**D-*allo*-Thr3**				
$^3J_{HNH\alpha}$	6.79	NH	8.10	
$^3J_{H\alpha H\beta}$	ND	CH α	3.98	61.76
		CO		not allocated
		CH β	5.31	70.21
		CH_3 γ	1.30	18.52
**D-Val4**				
$^3J_{HNH\alpha}$	5.30	NH	7.42	
		CH α	3.48	65.07
		CO		not allocated
		CH β	2.17	29.91
		CH_3 γ	0.91	19.46
		CH_3 γ	0.95	21.00
**D-Leu5**				
$^3J_{HNH\alpha}$	ND	NH	7.76	
		CH α	3.97	55.65
		CO		not allocated
		CH_2 β1	1.50	40.56
		CH_2 β2	1.67	40.56
		CH γ	1.76	25.43
		CH_3 δ	0.87	21.25
		CH_3 δ	0.86	23.26
**D-Ser6**				
$^3J_{HNH\alpha}$	8.56	NH	7.17	
		CH α	4.31	56.37
		CO		not allocated
		CH_2 β1	3.82	64.63
		CH_2 β2	4.14	64.63
		OH γ	4.94	
**L-Leu7**				
$^3J_{HNH\alpha}$	6.08	NH	7.12	
		CH α	4.13	54.79
		CO		not allocated
		CH_2 β1	1.58	42.02
		CH_2 β2	1.89	42.02
		CH γ	1.89	25.44
		CH_3 δ	0.90	21.28
		CH_3 δ	0.99	23.43
**D-Ser8**				
$^3J_{HNH\alpha}$	8.92	NH	7.95	
		CH α	4.42	56.96
		CO		not allocated
		CH_2 β1	3.66	63.10
		CH_2 β2	3.86	63.10
		OH γ	not allocated	
**L-Ile9**				
$^3J_{HNH\alpha}$	10.04	NH	6.67	
		CH α	4.56	57.12
		CO		not allocated
		CH β	1.97	36.84
		CH_3 γ	0.81	16.16
		CH_2 γ1	0.96	25.19
		CH_2 γ2	1.16	25.19
		CH_3 δ	0.86	12.25

ND= not determined

pseudodesmin V4A L5A L7A I9A (12)

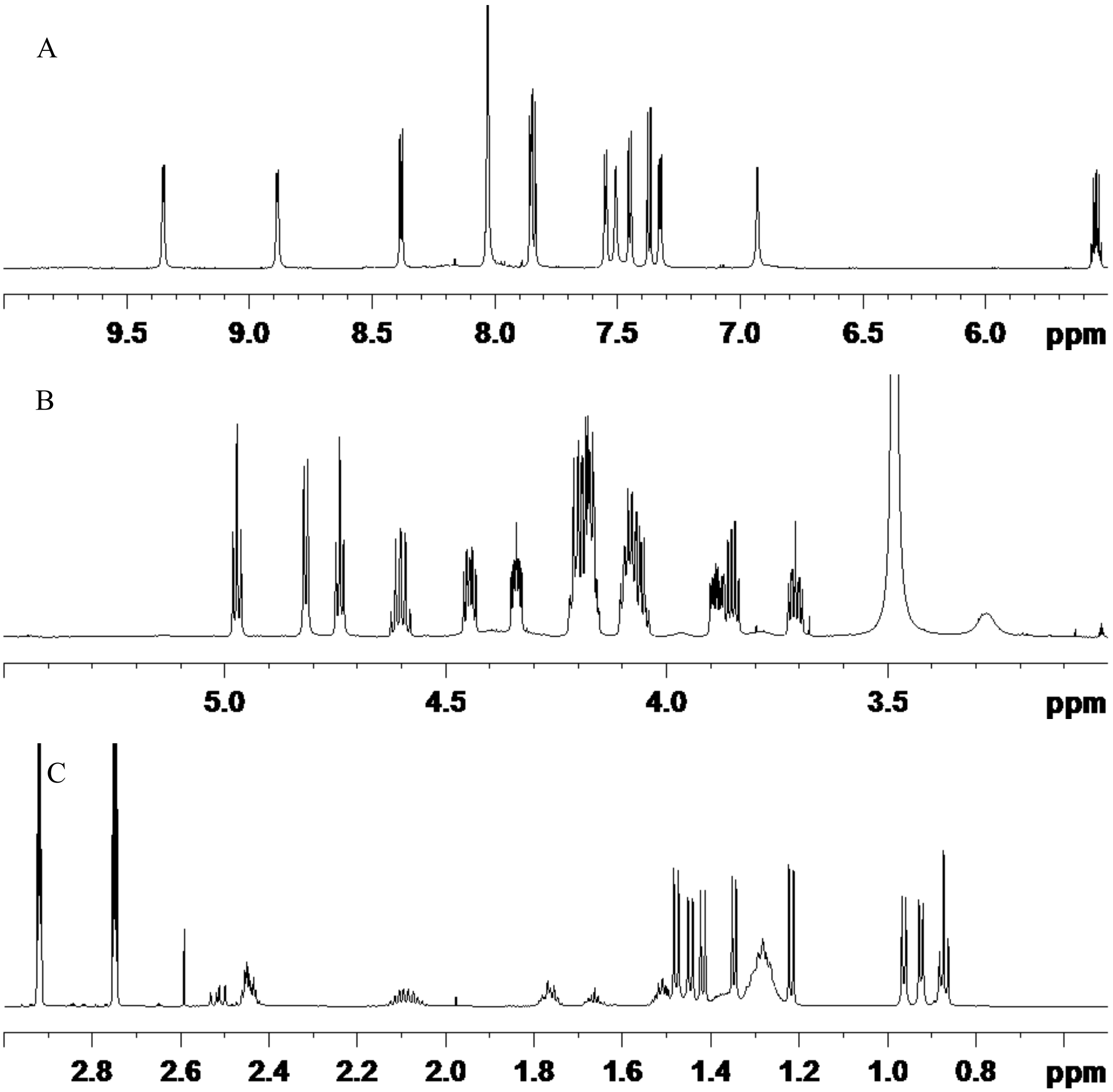

Figure S76. ^{1}H spectrum of pseudodesmin V4A L5A L7A I9A (**12**) (DMF-d7, 25°C, 700MHz) A) H^{N} region, B) H^{α} region and C) aliphatic region.

Table S13. ^{1}H and ^{13}C assignment of synthetic **pseudodesmin V4A L5A L7A I9A** (DMF-d7, 25°C, 700 MHz). Scalar couplings in Hz.

			^{1}H δ [ppm]	^{13}C δ [ppm]
**(*R*)-HDA**				
		CO		174.12
		CH_2 α1	2.45	44.20
		CH_2 α2	2.52	44.20
		CH β	4.08	68.63
		CH_2 γ	1.51	37.98
		CH_2 δ1	1.37	25.73
		CH_2 δ2	1.48	25.73
		CH_2 ε	1.28	29.50
		CH_2 ζ	1.29	29.82
		CH_2 η	1.26	32.02
		CH_2 θ	1.28	22.77
		CH_3 ι	0.87	13.94
		OH	4.82	
**L-Leu1**				
$^{3}J_{HNH\alpha}$	4.89	NH	8.89	
		CH α	4.21	53.40
		CO		175.69
		CH_2 β1	1.66	39.61
		CH_2 β2	1.77	39.61
		CH γ	1.77	24.79
		CH_3 δ	0.92	21.65
		CH_3 δ	0.96	22.91
**D-Gln2**				
$^{3}J_{HNH\alpha}$	4.88	NH	9.35	
		CH α	4.17	56.39
		CO		175.54
		CH_2 β	2.09	26.73
		CH_2 γ	2.45	31.64
		CO δ		174.29
		NH_2	6.93/7.51	
**D-*allo*-Thr3**				
$^{3}J_{HNH\alpha}$	7.18	NH	8.38	
$^{3}J_{H\alpha H\beta}$	10.54	CH α	4.18	60.67
		CO		173.34
		CH β	5.55	69.23
		CH_3 γ	1.35	18.06
**D-Ala4**				
$^{3}J_{HNH\alpha}$	6.10	NH	7.55	
		CH α	4.17	52.23
		CO		175.08
		CH_3 β	1.45	16.19
**D-Ala5**				
$^{3}J_{HNH\alpha}$	4.60	NH	7.85	
		CH α	4.06	51.79
		CO		173.32
		CH_3 β	1.42	16.76
**D-Ser6**				
$^{3}J_{HNH\alpha}$	8.71	NH	7.37	
		CH α	4.34	56.18
		CO		170.81
		CH_2 β1	3.88	62.87
		CH_2 β2	4.09	62.87
		OH γ	4.97	
**L-Ala7**				
$^{3}J_{HNH\alpha}$	6.67	NH	7.33	
		CH α	4.20	50.79
		CO		173.12
		CH_3 β	1.48	17.10
**D-Ser8**				
$^{3}J_{HNH\alpha}$	8.83	NH	7.84	
		CH α	4.45	55.89
		CO		170.38
		CH_2 β1	3.71	62.59
		CH_2 β2	3.85	62.59
		OH γ	4.74	
**L-Ala9**				
$^{3}J_{HNH\alpha}$	8.72	NH	7.37	
		CH α	4.60	47.57
		CO		171.05
		CH_3 β	1.22	16.98

ND= not determined

pseudodesmin C4 (13)

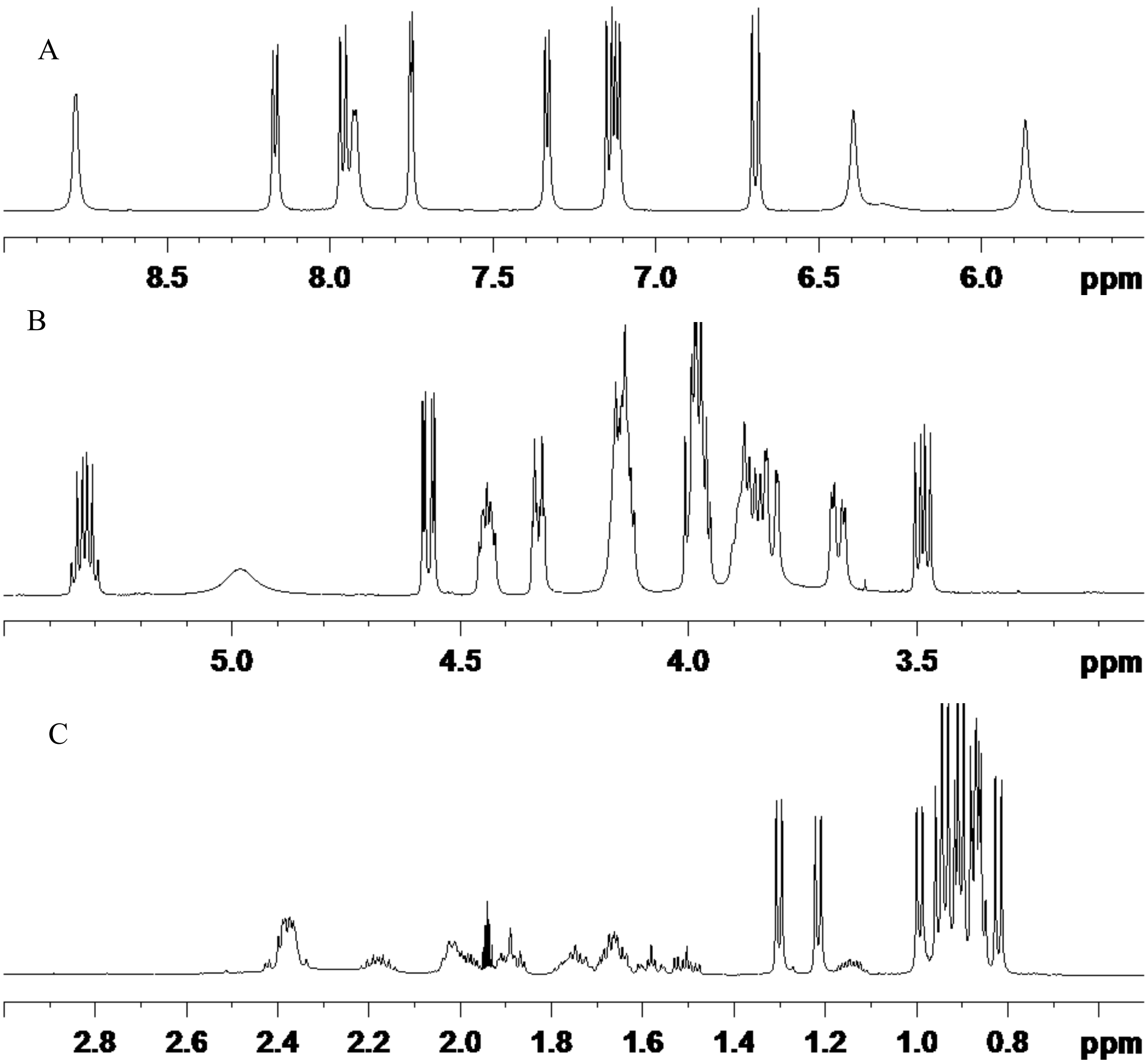

Figure S77. ^{1}H spectrum of pseudodesmin C4 (**13**) (CD_3CN, 25°C, 500MHz) A) H^N region, B) H^α region and C) aliphatic region.

Table S14. ^{1}H and ^{13}C assignment of **pseudodesmin C4** (CD_3CN, 25°C, 500 MHz). Scalar couplings in Hz.

			^{1}H δ [ppm]	^{13}C δ [ppm]
**(*R*)-C4**				
		CO		174.96
		CH_2 α1	2.41	46.17
		CH_2 α2	2.41	46.17
		CH β	4.18	65.69
		CH_3 γ	1.23	23.75
		OH	not allocated	
**L-Leu1**				
$^{3}J_{HNH\alpha}$	4.49	NH	7.94	
		CH α	3.90	53.73
		CO		175.34
		CH_2 β1	1.68	39.28
		CH_2 β2	1.76	39.28
		CH γ	1.69	25.35
		CH_3 δ	0.92	22.06
		CH_3 δ	0.96	23.12
**D-Gln2**				
$^{3}J_{HNH\alpha}$	2.35	NH	8.80	
		CH α	4.00	57.41
		CO		176.61
		CH_2 β	2.04	26.33
		CH_2 γ	2.39	31.96
		CO δ		175.99
		NH_2	5.89/6.41	
**D-*allo*-Thr3**				
$^{3}J_{HNH\alpha}$	6.94	NH	8.19	
		CH α	4.01	61.67
		CO		174.26
		CH β	5.34	70.26
		CH_3 γ	1.32	18.38
**D-Val4**				
$^{3}J_{HNH\alpha}$	6.42	NH	7.35	
		CH α	3.50	64.96
		CO		174.55
		CH β	2.20	29.89
		CH_3 γ	0.94	19.43
		CH_3 γ	0.97	20.95
**D-Leu5**				
$^{3}J_{HNH\alpha}$	3.97	NH	7.77	
		CH α	3.98	55.62
		CO		173.56
		CH_2 β1	1.52	40.58
		CH_2 β2	1.68	40.58
		CH γ	1.78	25.42
		CH_3 δ	0.88	23.27
		CH_3 δ	0.90	21.23
**D-Ser6**				
$^{3}J_{HNH\alpha}$	8.34	NH	7.16	
		CH α	4.35	56.35
		CO		171.97
		CH_2 β1	3.84	64.69
		CH_2 β2	4.17	64.66
		OH γ	5.00	
**L-Leu7**				
$^{3}J_{HNH\alpha}$	6.20	NH	7.14	
		CH α	4.15	54.82
		CO		173.82
		CH_2 β1	1.60	41.99
		CH_2 β2	1.91	41.99
		CH γ	1.91	25.43
		CH_3 δ	0.92	21.31
		CH_3 δ	1.01	23.41
**D-Ser8**				
$^{3}J_{HNH\alpha}$	8.87	NH	7.98	
		CH α	4.46	56.93
		CO		171.77
		CH_2 β1	3.69	63.10
		CH_2 β2	3.88	63.10
		OH γ	not allocated	
**L-Ile9**				
$^{3}J_{HNH\alpha}$	10.08	NH	6.71	
		CH α	4.58	57.07
		CO		170.05
		CH β	2.00	36.78
		CH_3 γ	0.84	16.18
		CH_2 γ1	0.97	25.16
		CH_2 γ2	1.17	25.16
		CH_3 δ	0.88	12.23

ND= not determined

pseudodesmin C6 (14)

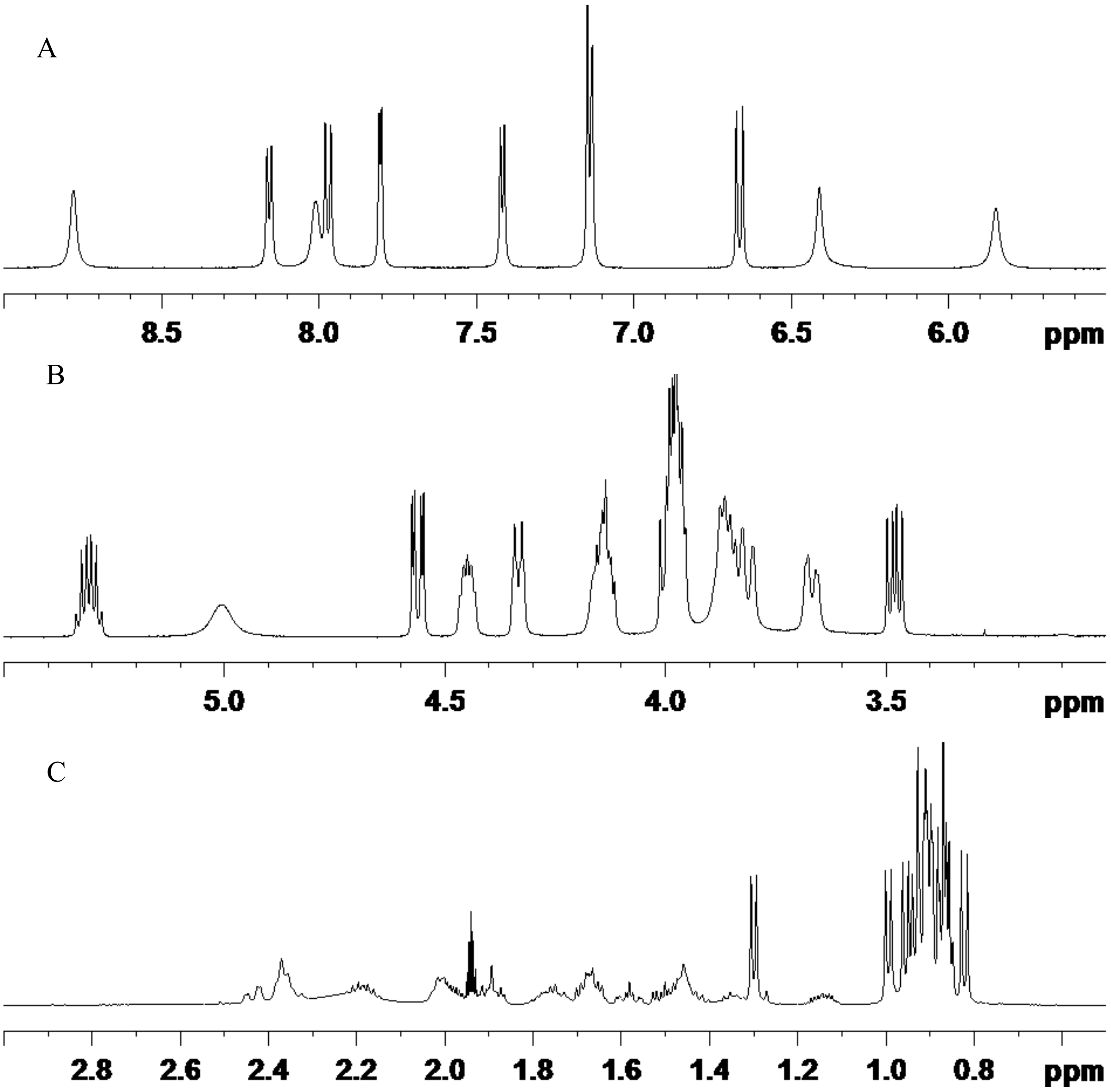

Figure S78. ^{1}H spectrum of pseudodesmin C6 (**14**) (CD_3CN, 25°C, 500MHz) A) H^N region, B) H^α region and C) aliphatic region.

Table S15. ^{1}H and ^{13}C assignment of **pseudodesmin C6** (CD_3CN, 25°C, 500 MHz). Scalar couplings in Hz.

			^{1}H δ [ppm]	^{13}C δ [ppm]
**(*R*)-C6**				
		CO		175.03
		CH_2 α1	2.37	44.71
		CH_2 α2	2.46	44.71
		CH β	4.00	69.36
		CH_2 γ	1.48	40.44
		CH_2 δ1	1.37	19.48
		CH_2 δ2	1.48	19.48
		CH_3 ε	0.93	14.26
		OH	not allocated	
**L-Leu1**				
$^3J_{HNH\alpha}$	ND	NH	8.03	
		CH α	3.89	53.74
		CO		175.31
		CH_2 β1	1.70	39.23
		CH_2 β2	1.77	39.23
		CH γ	1.69	25.37
		CH_3 δ	0.92	22.04
		CH_3 δ	0.96	23.20
**D-Gln2**				
$^3J_{HNH\alpha}$	ND	NH	8.80	
		CH α	4.00	57.54
		CO		176.69
		CH_2 β	2.03	26.44
		CH_2 γ	2.39	31.98
		CO δ		175.95
		NH_2	5.87/6.43	
**D-*allo*-Thr3**				
$^3J_{HNH\alpha}$	6.97	NH	8.18	
		CH α	4.01	61.75
		CO		174.27
		CH β	5.33	70.30
		CH_3 γ	1.32	18.48
**D-Val4**				
$^3J_{HNH\alpha}$	6.05	NH	7.44	
		CH α	3.50	65.16
		CO		174.62
		CH β	2.21	29.94
		CH_3 γ	0.94	19.45
		CH_3 γ	0.98	21.02
**D-Leu5**				
$^3J_{HNH\alpha}$	4.04	NH	7.83	
		CH α	3.99	55.68
		CO		173.57
		CH_2 β1	1.52	40.58
		CH_2 β2	1.70	40.58
		CH γ	1.80	25.45
		CH_3 δ	0.88	23.28
		CH_3 δ	0.90	21.25
**D-Ser6**				
$^3J_{HNH\alpha}$	7.52	NH	7.16	
		CH α	4.35	56.31
		CO		171.98
		CH_2 β1	3.84	64.66
		CH_2 β2	4.17	64.66
		OH γ	5.03	
**L-Leu7**				
$^3J_{HNH\alpha}$		NH	7.16	
		CH α	4.16	54.89
		CO		173.86
		CH_2 β1	1.61	41.99
		CH_2 β2	1.92	41.99
		CH γ	1.92	25.45
		CH_3 δ	0.92	21.32
		CH_3 δ	1.01	23.44
**D-Ser8**				
$^3J_{HNH\alpha}$		NH	7.99	
		CH α	4.46	56.88
		CO		171.84
		CH_2 β1	3.69	63.11
		CH_2 β2	3.88	63.11
		OH γ	not allocated	
**L-Ile9**				
$^3J_{HNH\alpha}$		NH	6.68	
		CH α	4.59	57.19
		CO		170.00
		CH β	2.00	36.88
		CH_3 γ	0.85	16.23
		CH_2 γ1	1.00	25.17
		CH_2 γ2	1.17	25.17
		CH_3 δ	0.89	12.29

ND= not determined

pseudodesmin C8 (15)

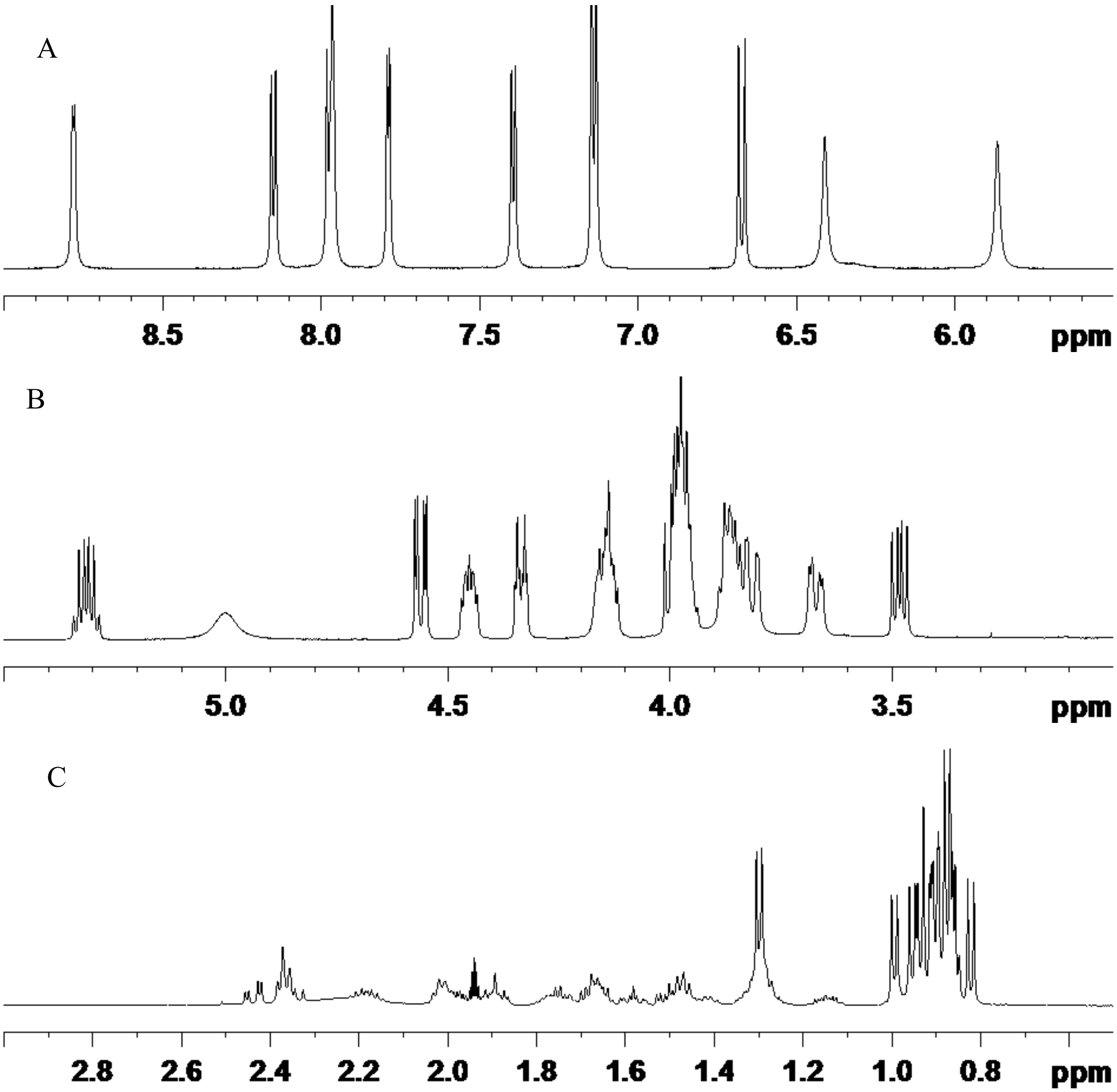

Figure S79. ^{1}H spectrum of pseudodesmin C8 (**15**) (CD_3CN, 25°C, 500MHz) A) H^N region, B) H^α region and C) aliphatic region.

Table S16. ^{1}H and ^{13}C assignment of **pseudodesmin C8** (CD_3CN, 25°C, 500 MHz). Scalar couplings in Hz.

			^{1}H δ [ppm]	^{13}C δ [ppm]				^{1}H δ [ppm]	^{13}C δ [ppm]
**(*R*)-C8**					**D-Leu5**				
		CO		175.09	$^{3}J_{HNH\alpha}$	4.04	NH	7.81	
		CH_2 α1	2.37	44.73			CH α	3.99	55.62
		CH_2 α2	2.46	44.72			CO		173.58
		CH β	3.98	69.51			CH_2 β1	1.52	40.57
		CH_2 γ	1.49	38.14			CH_2 β2	1.69	40.57
		CH_2 δ1	1.33	25.96			CH γ	1.80	25.44
		CH_2 δ2	1.45	25.96			CH_3 δ	0.88	23.27
		CH_2 ε	1.32	23.24			CH_3 δ	0.89	21.24
		CH_2 ζ	1.31	32.43	**D-Ser6**				
		CH_3 η	0.90	14.26	$^{3}J_{HNH\alpha}$	7.45	NH	7.16	
		OH	not allocated				CH α	4.35	56.33
							CO		172.01
							CH_2 β1	3.83	64.66
**L-Leu1**							CH_2 β2	4.17	64.66
$^{3}J_{HNH\alpha}$	ND	NH	7.99				OH γ	5.02	
		CH α	3.88	53.68	**L-Leu7**				
		CO		175.30	$^{3}J_{HNH\alpha}$	7.45	NH	7.16	
		CH_2 β1	1.68	39.23			CH α	4.16	54.90
		CH_2 β2	1.78	39.23			CO		173.90
		CH γ	1.69	25.34			CH_2 β1	1.60	42.00
		CH_3 δ	0.92	22.06			CH_2 β2	1.91	42.00
		CH_3 δ	0.95	23.14			CH γ	1.92	25.44
**D-Gln2**							CH_3 δ	0.92	21.32
$^{3}J_{HNH\alpha}$	3.50	NH	8.80				CH_3 δ	1.01	23.41
		CH α	4.00	57.51	**D-Ser8**				
		CO		176.63	$^{3}J_{HNH\alpha}$	8.89	NH	8.00	
		CH_2 β	2.04	26.50			CH α	4.47	57.01
		CH_2 γ	2.40	31.85			CO		171.83
		CO δ		176.01			CH_2 β1	3.70	63.10
		NH_2	5.89/6.43				CH_2 β2	3.88	63.10
**D-*allo*-Thr3**							OH γ	not allocated	
$^{3}J_{HNH\alpha}$	7.00	NH	8.17		**L-Ile9**				
		CH α	4.01	61.68	$^{3}J_{HNH\alpha}$	10.05	NH	6.69	
		CO		174.26			CH α	4.58	57.09
		CH β	5.34	70.27			CO		169.98
		CH_3 γ	1.32	18.56			CH β	2.00	36.82
**D-Val4**							CH_3 γ	0.84	16.16
$^{3}J_{HNH\alpha}$	6.28	NH	7.41				CH_2 γ1	1.00	25.18
		CH α	3.49	65.01			CH_2 γ2	1.17	25.18
		CO		174.61			CH_3 δ	0.88	12.28
		CH β	2.21	29.84					
		CH_3 γ	0.94	19.45					
		CH_3 γ	0.97	20.98					

ND= not determined

pseudodesmin C12 (16)

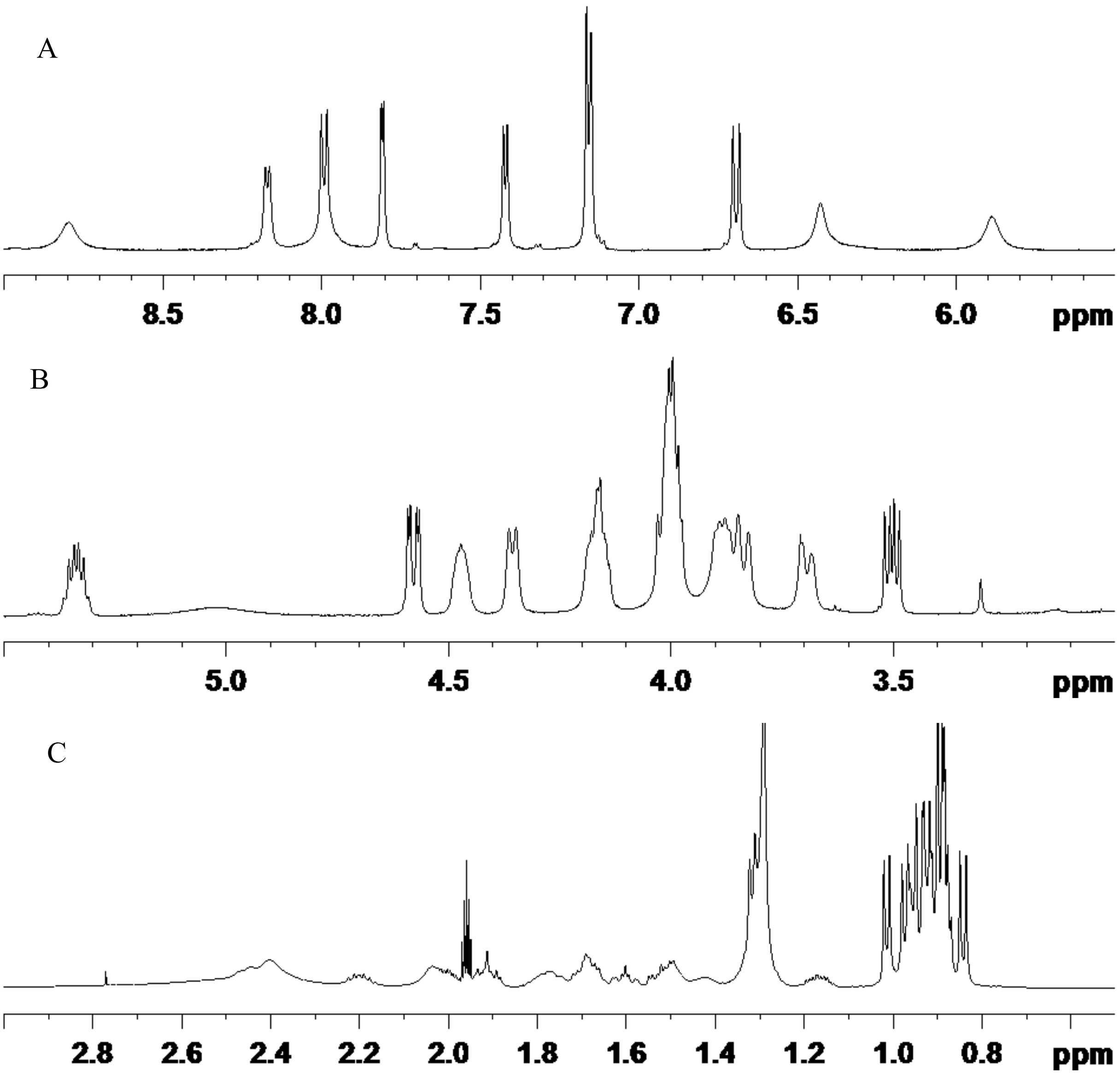

Figure S80. ^{1}H spectrum of pseudodesmin C12 (**16**) (CD_3CN, 25°C, 500MHz) A) H^N region, B) H^{α} region and C) aliphatic region.

Table S17. ^{1}H and ^{13}C assignment of **pseudodesmin C12** (CD_3CN, 25°C, 500 MHz). Scalar couplings in Hz.

			^{1}H δ [ppm]	^{13}C δ [ppm]				^{1}H δ [ppm]	^{13}C δ [ppm]
**(*R*)-C12**					**D-Leu5**				
		CO		175.06	$^3J_{HNH\alpha}$	3.86	NH	7.81	
		CH_2 α1	2.37	44.69			CH α	3.99	55.63
		CH_2 α2	2.47	44.69			CO		173.59
		CH β	4.00	69.51			CH_2 β1	1.52	40.56
		CH_2 γ	1.49	38.16			CH_2 β2	1.69	40.56
		CH_2 δ1	1.32	26.28			CH γ	1.79	25.42
		CH_2 δ2	1.43	26.28			CH_3 δ	0.88	23.28
		CH_2 ε	1.29	30.18			CH_3 δ	0.89	21.26
		CH_2 ζ	1.29	30.18	**D-Ser6**				
		CH_2 η	1.29	30.18	$^3J_{HNH\alpha}$	7.20	NH	7.16	
		CH_2 θ	1.29	30.18			CH α	4.35	56.27
		CH_2 ι	1.29	32.62			CO		172.01
		CH_2 κ	1.30	23.35			CH_2 β1	3.84	64.66
		CH_3 λ	0.90	14.35			CH_2 β2	4.17	64.66
		OH	not allocated				OH γ	5.06	
**L-Leu1**					**L-Leu7**				
$^3J_{HNH\alpha}$	ND	NH	7.98		$^3J_{HNH\alpha}$	7.20	NH	7.16	
		CH α	3.90	53.75			CH α	4.16	54.88
		CO		175.30			CO		173.89
		CH_2 β1	1.68	39.25			CH_2 β1	1.60	41.98
		CH_2 β2	1.77	39.25			CH_2 β2	1.92	41.98
		CH γ	1.69	25.35			CH γ	1.92	25.43
		CH_3 δ	0.92	22.09			CH_3 δ	0.92	21.32
		CH_3 δ	0.96	23.17			CH_3 δ	1.02	23.40
**D-Gln2**					**D-Ser8**				
$^3J_{HNH\alpha}$	ND	NH	8.80		$^3J_{HNH\alpha}$	8.87	NH	7.99	
		CH α	4.00	57.48			CH α	4.48	56.91
		CO		176.63			CO		171.81
		CH_2 β	2.03	26.33			CH_2 β1	3.70	63.11
		CH_2 γ	2.41	32.05			CH_2 β2	3.88	63.11
		CO δ		not allocated			OH γ	not allocated	
		NH_2	5.89/6.43		**L-Ile9**				
**D-*allo*-Thr3**					$^3J_{HNH\alpha}$	10.06	NH	6.69	
$^3J_{HNH\alpha}$	6.44	NH	8.17				CH α	4.58	57.10
		CH α	4.01	61.64			CO		169.97
		CO		174.26			CH β	1.99	36.86
		CH β	5.34	70.23			CH_3 γ	0.85	16.10
		CH_3 γ	1.32	18.54			CH_2 γ1	0.99	25.19
**D-Val4**							CH_2 γ2	1.18	25.19
$^3J_{HNH\alpha}$	6.13	NH	7.42				CH_3 δ	0.89	12.36
		CH α	3.50	64.99					
		CO		174.59					
		CH β	2.21	29.84					
		CH_3 γ	0.94	19.45					
		CH_3 γ	0.98	21.00					

ND= not determined

pseudodesmin C14 (17)

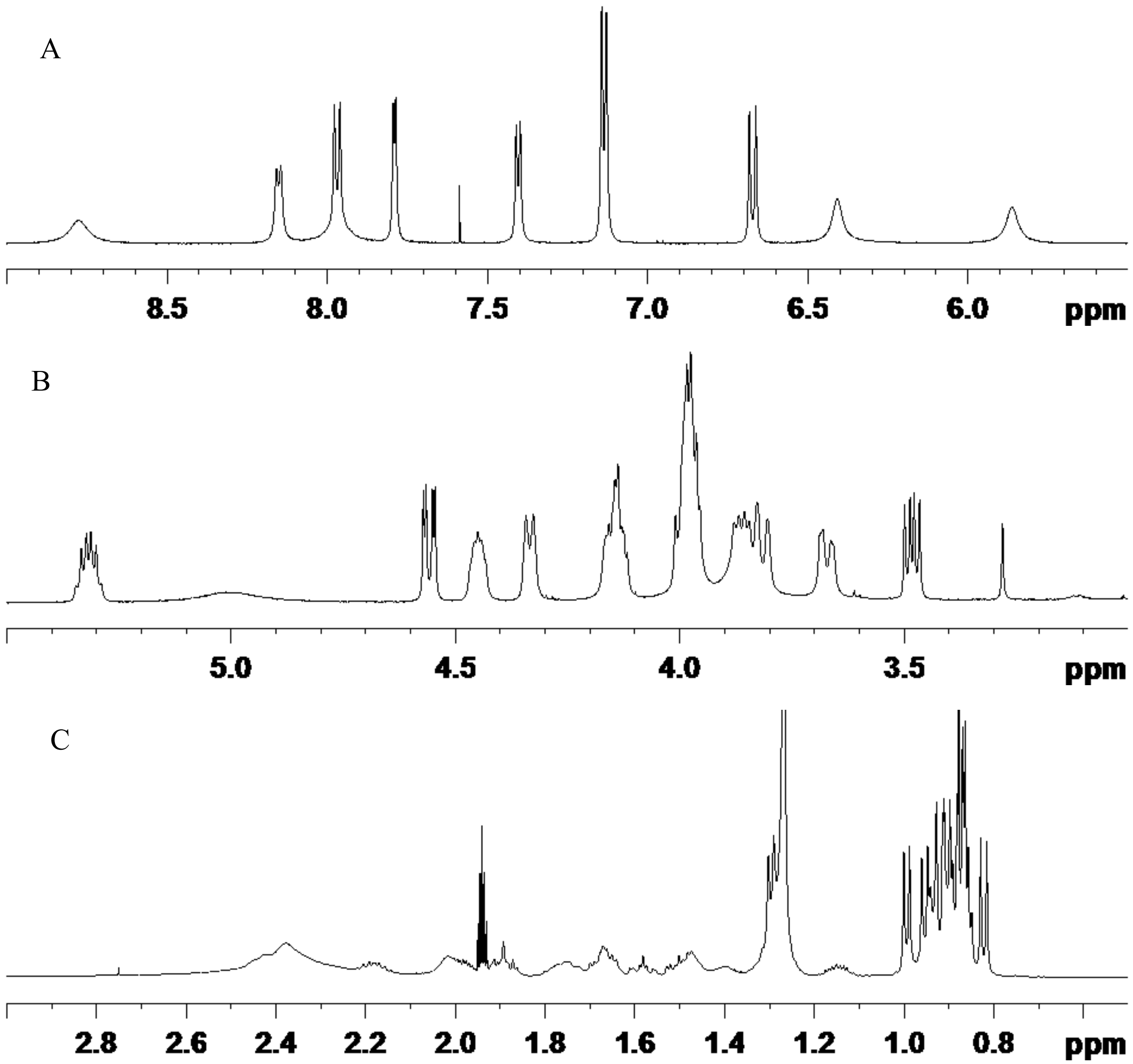

Figure S81. ^{1}H spectrum of pseudodesmin C14 (**17**) (CD_3CN, 25°C, 500MHz) A) H^N region, B) H^{α} region and C) aliphatic region.

Table S18. ^{1}H and ^{13}C assignment of **pseudodesmin C14** (CD_3CN, 25°C, 500 MHz). Scalar couplings in Hz.

			^{1}H δ [ppm]	^{13}C δ [ppm]
**(*R*)-C14**				
		CO		not allocated
		CH_2 α1	2.38	44.69
		CH_2 α2	2.46	44.69
		CH β	3.99	69.55
		CH_2 γ	1.49	38.26
		CH_2 δ1	1.33	26.28
		CH_2 δ2	1.44	26.28
		CH_2 ε	1.29	30.45
		CH_2 ζ	1.29	30.45
		CH_2 η	1.29	30.45
		CH_2 θ	1.29	30.45
		CH_3 ι	1.29	30.45
		CH_2 κ	1.29	30.45
		CH_2 λ	1.29	32.69
		CH_2 μ	1.32	23.34
		CH_3 ν	0.91	14.53
		OH	not allocated	
**L-Leu1**				
$^{3}J_{HNH\alpha}$	ND	NH	7.99	
		CH α	3.89	53.83
		CO		175.29
		CH_2 β1	1.68	39.27
		CH_2 β2	1.76	39.27
		CH γ	1.69	25.34
		CH_3 δ	0.92	22.11
		CH_3 δ	0.95	23.15
**D-Gln2**				
$^{3}J_{HNH\alpha}$	ND	NH	8.80	
		CH α	4.00	57.53
		CO		176.64
		CH_2 β	2.05	26.39
		CH_2 γ	2.41	32.02
		CO δ		not allocated
		NH_2	5.89/6.43	
**D-*allo*-Thr3**				
$^{3}J_{HNH\alpha}$	6.50	NH	8.17	
		CH α	4.01	61.69
		CO		174.27
		CH β	5.34	70.29
		CH_3 γ	1.31	18.47
**D-Val4**				
$^{3}J_{HNH\alpha}$	6.10	NH	7.42	
		CH α	3.51	65.03
		CO		174.59
		CH β	2.21	29.91
		CH_3 γ	0.94	19.48
		CH_3 γ	0.97	21.00
**D-Leu5**				
$^{3}J_{HNH\alpha}$	3.77	NH	7.81	
		CH α	3.99	55.63
		CO		173.58
		CH_2 β1	1.52	40.58
		CH_2 β2	1.69	40.58
		CH γ	1.79	25.44
		CH_3 δ	0.88	23.29
		CH_3 δ	0.89	21.23
**D-Ser6**				
$^{3}J_{HNH\alpha}$	7.09	NH	7.16	
		CH α	4.37	56.40
		CO		172.01
		CH_2 β1	3.83	64.67
		CH_2 β2	4.18	64.67
		OH γ	5.06	
**L-Leu7**				
$^{3}J_{HNH\alpha}$	7.09	NH	7.16	
		CH α	4.16	54.86
		CO		173.90
		CH_2 β1	1.60	41.99
		CH_2 β2	1.91	41.99
		CH γ	1.92	25.44
		CH_3 δ	0.92	21.36
		CH_3 δ	1.01	23.41
**D-Ser8**				
$^{3}J_{HNH\alpha}$	8.86	NH	7.99	
		CH α	4.48	56.86
		CO		171.84
		CH_2 β1	3.69	63.12
		CH_2 β2	3.88	63.12
		OH γ	not allocated	
**L-Ile9**				
$^{3}J_{HNH\alpha}$	10.05	NH	6.69	
		CH α	4.58	57.12
		CO		169.95
		CH β	2.00	36.94
		CH_3 γ	0.85	16.23
		CH_2 γ1	0.99	25.19
		CH_2 γ2	1.17	25.19
		CH_3 δ	0.90	12.29

ND= not determined

3*S*-epi-pseudodesmin (18)

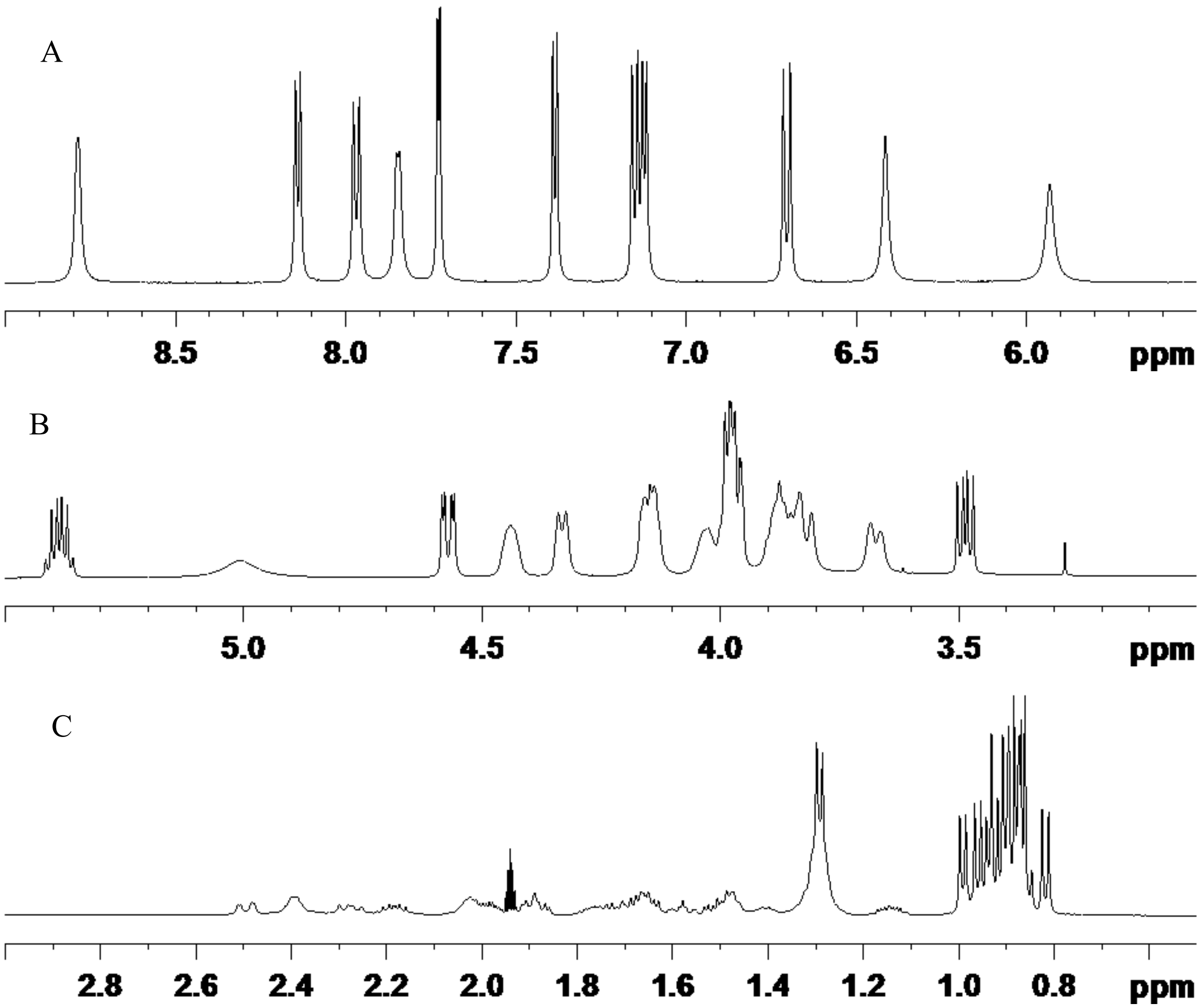

Figure S82. ^{1}H spectrum of 3*S*-epi-pseudodesmin (**18**) (CD_3CN, 25°C, 500MHz) A) H^N region, B) H^{α} region and C) aliphatic region.

Table S19. ^{1}H and ^{13}C assignment of synthetic **3*S*-epi-pseudodesmin** (CD_3CN, 25°C, 500 MHz). Scalar couplings in Hz.

			^{1}H δ [ppm]	^{13}C δ [ppm]
**(*S*)-HDA**				
		CO		175.41
		CH_2 α1	2.28	44.83
		CH_2 α2	2.50	44.83
		CH β	4.03	69.87
		CH_2 γ	1.48	38.40
		CH_2 δ1	1.31	26.17
		CH_2 δ2	1.40	26.17
		CH_2 ε	1.29	29.99
		CH_2 ζ	1.30	30.20
		CH_2 η	1.28	32.55
		CH_2 θ	1.29	23.35
		CH_3 ι	0.88	14.38
		OH	not allocated	
**L-Leu1**				
$^{3}J_{HNH\alpha}$	4.84	NH	7.85	
		CH α	3.88	53.74
		CO		175.30
		CH_2 β1	1.66	39.33
		CH_2 β2	1.72	39.33
		CH γ	1.67	25.30
		CH_3 δ	0.90	22.05
		CH_3 δ	0.94	23.17
**D-Gln2**				
$^{3}J_{HNH\alpha}$	ND	NH	8.79	
		CH α	3.98	57.53
		CO		176.70
		CH_2 β	2.03	26.16
		CH_2 γ	2.39	31.99
		CO δ		176.20
		NH_2	6.42/5.93	
**D-*allo*-Thr3**				
$^{3}J_{HNH\alpha}$	7.07	NH	8.14	
$^{3}J_{H\alpha H\beta}$	10.67	CH α	3.98	61.76
		CO		174.31
		CH β	5.39	70.38
		CH_3 γ	1.29	18.40
**D-Val4**				
$^{3}J_{HNH\alpha}$	6.31	NH	7.39	
		CH α	3.49	64.93
		CO		174.56
		CH β	2.18	29.89
		CH_3 γ	0.92	19.45
		CH_3 γ	0.96	20.98
**D-Leu5**				
$^{3}J_{HNH\alpha}$	4.01	NH	7.73	
		CH α	3.97	55.66
		CO		173.57
		CH_2 β1	1.51	40.62
		CH_2 β2	1.66	40.62
		CH γ	1.76	25.41
		CH_3 δ	0.87	21.28
		CH_3 δ	0.87	23.26
**D-Ser6**				
$^{3}J_{HNH\alpha}$	8.27	NH	7.15	
		CH α	4.33	56.39
		CO		172.01
		CH_2 β1	3.82	64.72
		CH_2 β2	4.15	64.72
		OH γ	5.01	
**L-Leu7**				
$^{3}J_{HNH\alpha}$	6.27	NH	7.12	
		CH α	4.15	54.79
		CO		173.83
		CH_2 β1	1.58	42.00
		CH_2 β2	1.89	42.00
		CH γ	1.89	25.43
		CH_3 δ	0.90	21.31
		CH_3 δ	0.99	23.44
**D-Ser8**				
$^{3}J_{HNH\alpha}$	8.72	NH	7.97	
		CH α	4.44	57.04
		CO		171.74
		CH_2 β1	3.67	63.11
		CH_2 β2	3.86	63.11
		OH γ	5.01	
**L-Ile9**				
$^{3}J_{HNH\alpha}$	10.08	NH	6.70	
		CH α	4.57	57.07
		CO		170.11
		CH β	1.98	36.85
		CH_3 γ	0.82	16.20
		CH_2 γ1	0.96	25.16
		CH_2 γ2	1.14	25.16
		CH_3 δ	0.86	12.25

ND= not determined

3-deoxy-pseudodesmin (19)

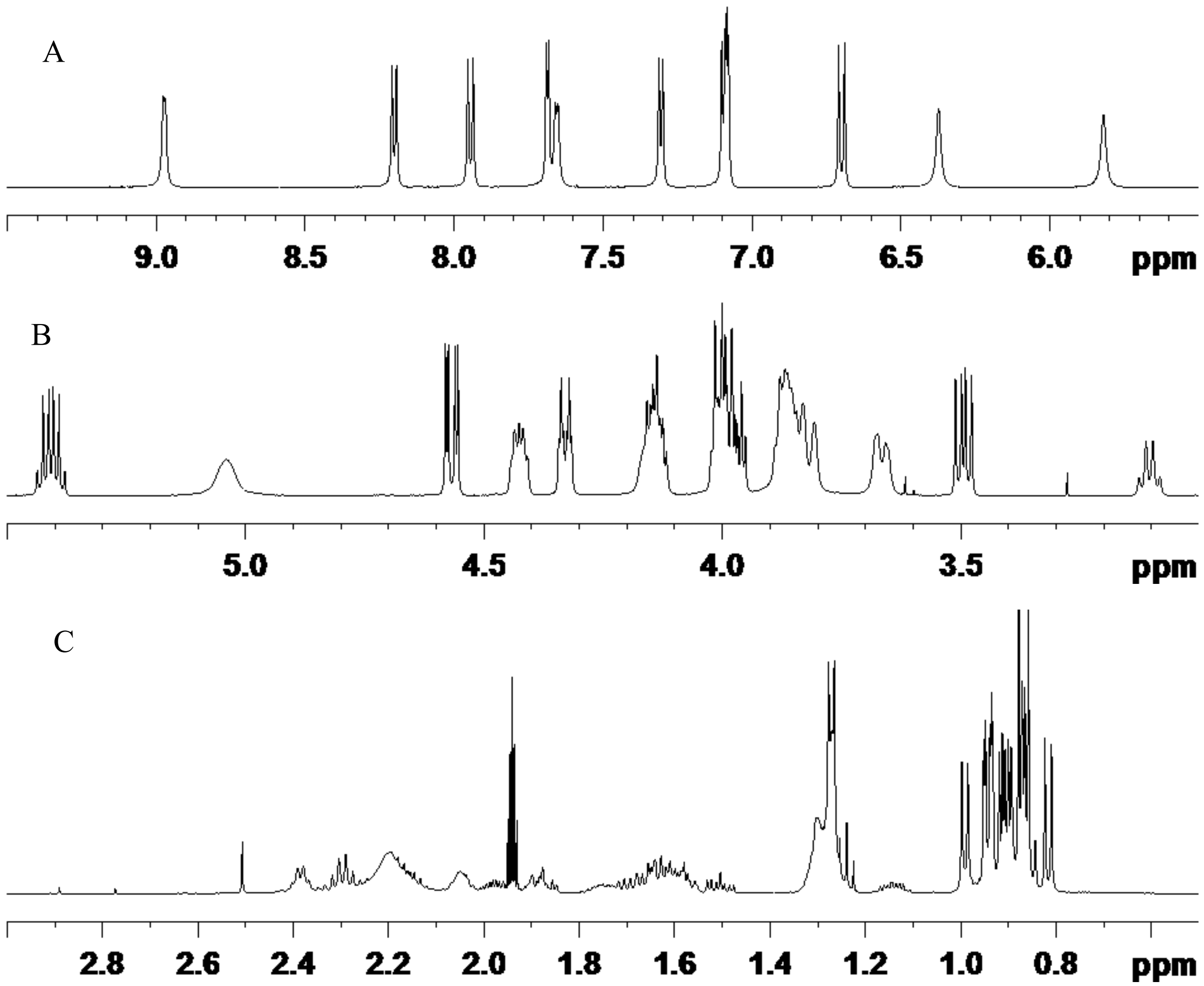

Figure S83. ^{1}H spectrum of 3-deoxy-pseudodesmin (**19**) (CD_3CN, 25°C, 500MHz) A) H^N region, B) H^α region and C) aliphatic region.

Table S20. ^{1}H and ^{13}C assignment of synthetic **3-deoxy-pseudodesmin** (CD_3CN, 25°C, 500 MHz). Scalar couplings in Hz.

			^{1}H δ [ppm]	^{13}C δ [ppm]				^{1}H δ [ppm]	^{13}C δ [ppm]
**DA**					**D-Leu5**				
		CO		177.02	$^3J_{HNH\alpha}$	4.08	NH	7.69	
		CH_2 α1	2.30	36.50			CH α	3.97	55.62
		CH_2 α2	2.30	36.50			CO		173.47
		CH_2 β	1.63	26.00			CH_2 β1	1.50	40.62
		CH_2 γ	1.31	29.76			CH_2 β2	1.65	40.62
		CH_2 δ1	1.31	30.00			CH γ	1.76	25.38
		CH_2 δ2	1.31	30.00			CH_3 δ	0.88	21.21
		CH_2 ε	1.28	30.00			CH_3 δ	0.87	23.26
		CH_2 ζ	1.28	30.21	**D-Ser6**				
		CH_2 η	1.27	32.59	$^3J_{HNH\alpha}$	5.29	NH	7.10	
		CH_2 θ	1.28	23.35			CH α	4.33	56.34
		CH_3 ι	0.88	14.36			CO		171.90
							CH_2 β1	3.82	64.75
**L-Leu1**							CH_2 β2	4.16	64.75
$^3J_{HNH\alpha}$	4.76	NH	7.66				OH γ	5.04	
		CH α	3.87	53.88	**L-Leu7**				
		CO		175.40	$^3J_{HNH\alpha}$	3.21	NH	7.08	
		CH_2 β1	1.59	39.65			CH α	4.14	54.77
		CH_2 β2	1.70	39.65			CO		173.75
		CH γ	1.65	25.30			CH_2 β1	1.58	42.01
		CH_3 δ	0.91	22.20			CH_2 β2	1.88	42.01
		CH_3 δ	0.95	23.03			CH γ	1.90	25.41
**D-Gln2**							CH_3 δ	0.90	21.28
$^3J_{HNH\alpha}$	3.67	NH	8.97				CH_3 δ	0.99	23.44
		CH α	4.01	57.36	**D-Ser8**				
		CO		176.47	$^3J_{HNH\alpha}$	8.88	NH	7.95	
		CH_2 β	2.05	26.09			CH α	4.43	57.01
		CH_2 γ	2.39	31.89			CO		171.70
		CO δ		175.95			CH_2 β1	3.67	63.12
		NH_2	6.38/5.82				CH_2 β2	3.86	63.12
**D-*allo*-Thr3**							OH γ	5.04	
$^3J_{HNH\alpha}$	7.51	NH	8.20		**L-Ile9**				
$^3J_{H\alpha H\beta}$	10.67	CH α	4.00	61.55	$^3J_{HNH\alpha}$	10.08	NH	6.70	
		CO		174.32			CH α	4.57	57.02
		CH β	5.41	70.35			CO		170.01
		CH_3 γ	1.27	18.31			CH β	1.98	36.89
**D-Val4**							CH_3 γ	0.82	16.18
$^3J_{HNH\alpha}$	6.48	NH	7.31				CH_2 γ1	0.97	25.17
		CH α	3.49	64.80			CH_2 γ2	1.14	25.17
		CO		174.51			CH_3 δ	0.87	12.25
		CH β	2.16	29.97					
		CH_3 γ	0.93	19.44					
		CH_3 γ	0.95	20.92					

ND= not determined

pseudodesmin D-Dap3 (20)

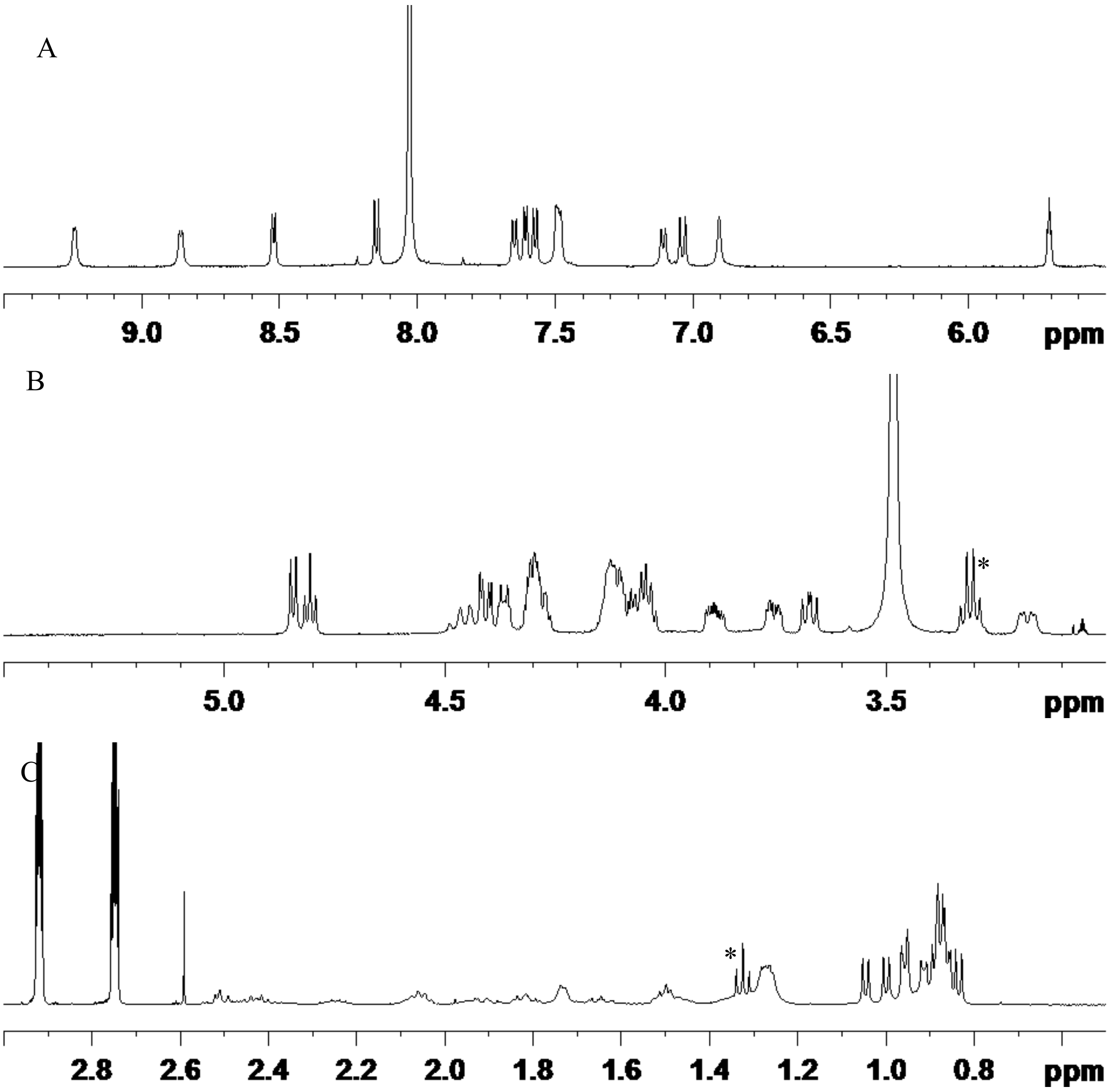

Figure S84. ^{1}H spectrum of pseudodesmin D-Dap3 (**20**) (DMF-d7, 25°C, 500MHz) A) H^N region, B) H^α region and C) aliphatic region. Asterisks indicate the resonances of an impurity.

Table S21. ^{1}H and ^{13}C assignment of **psdA D-Dap3** (DMF-d7, 25°C, 500 MHz). Scalar couplings in Hz.

			^{1}H δ [ppm]	^{13}C δ [ppm]				^{1}H δ [ppm]	^{13}C δ [ppm]
**(*R*)-HDA**					**D-Leu5**				
		CO		173.73	$^3J_{HNH\alpha}$	5.49	NH	7.61	
		CH_2 α1	2.51	44.02			CH α	4.12	54.49
		CH_2 α2	2.51	44.02			CO		173.37
		CH β	4.12	68.58			CH_2 β1	1.50	39.95
		CH_2 γ	1.50	37.86			CH_2 β2	1.82	39.95
		CH_2 δ1	1.36	25.73			CH γ	1.83	24.74
		CH_2 δ2	1.47	25.73			CH_3 δ	0.87	20.77
		CH_2 ε	1.27	29.54			CH_3 δ	0.89	23.01
		CH_2 ζ	1.28	29.70	**D-Ser6**				
		CH_2 η	1.26	32.03	$^3J_{HNH\alpha}$	7.17	NH	7.65	
		CH_2 θ	1.27	22.79			CH α	4.31	57.10
		CH_3 ι	0.88	13.96			CO		171.21
		OH	4.84				CH_2 β1	3.89	63.08
**L-Leu1**							CH_2 β2	4.04	63.08
$^3J_{HNH\alpha}$	5.10	NH	8.86				OH γ	4.81	
		CH α	4.13	53.03	**L-Leu7**				
		CO		175.48	$^3J_{HNH\alpha}$	7.44	NH	7.11	
		CH_2 β1	1.74	39.51			CH α	4.29	52.93
		CH_2 β2	1.74	39.51			CO		172.75
		CH γ	1.74	24.82			CH_2 β1	1.65	41.46
		CH_3 δ	0.92	21.91			CH_2 β2	1.91	41.46
		CH_3 δ	0.96	22.75			CH γ	1.94	24.62
**D-Gln2**							CH_3 δ	0.87	20.77
$^3J_{HNH\alpha}$	4.13	NH	9.24				CH_3 δ	1.00	23.33
		CH α	4.05	56.82	**D-Ser8**				
		CO		175.70	$^3J_{HNH\alpha}$	7.58	NH	8.15	
		CH_2 β	2.05	26.74			CH α	4.37	56.43
		CH_2 γ	2.43	31.82			CO		170.93
		CO δ		174.12			CH_2 β1	3.75	63.40
		NH_2	7.49/7.19				CH_2 β2	4.09	63.40
**D-Dap3**							OH γ	5.71	
$^3J_{HNH\alpha}$	5.92	NH	8.52		**L-Ile9**				
		CH α	4.29	56.21	$^3J_{HNH\alpha}$	9.86	NH	7.04	
		CO		174.52			CH α	4.41	57.31
		CH_2 β1	3.18	39.51			CO		170.66
		CH_2 β2	4.46	39.51			CH β	2.08	35.25
		NH β	7.49				CH_3 γ	0.84	15.88
**D-Val4**							CH_2 γ1	0.91	24.39
$^3J_{HNH\alpha}$	6.81	NH	7.57				CH_2 γ2	1.34	24.39
		CH α	3.67	63.26			CH_3 δ	0.88	11.85
		CO		173.09					
		CH β	2.25	29.32					
		CH_3 γ	0.96	19.29					
		CH_3 γ	1.05	20.50					

ND= not determined

pseudodesmin D-Ser3 (21)

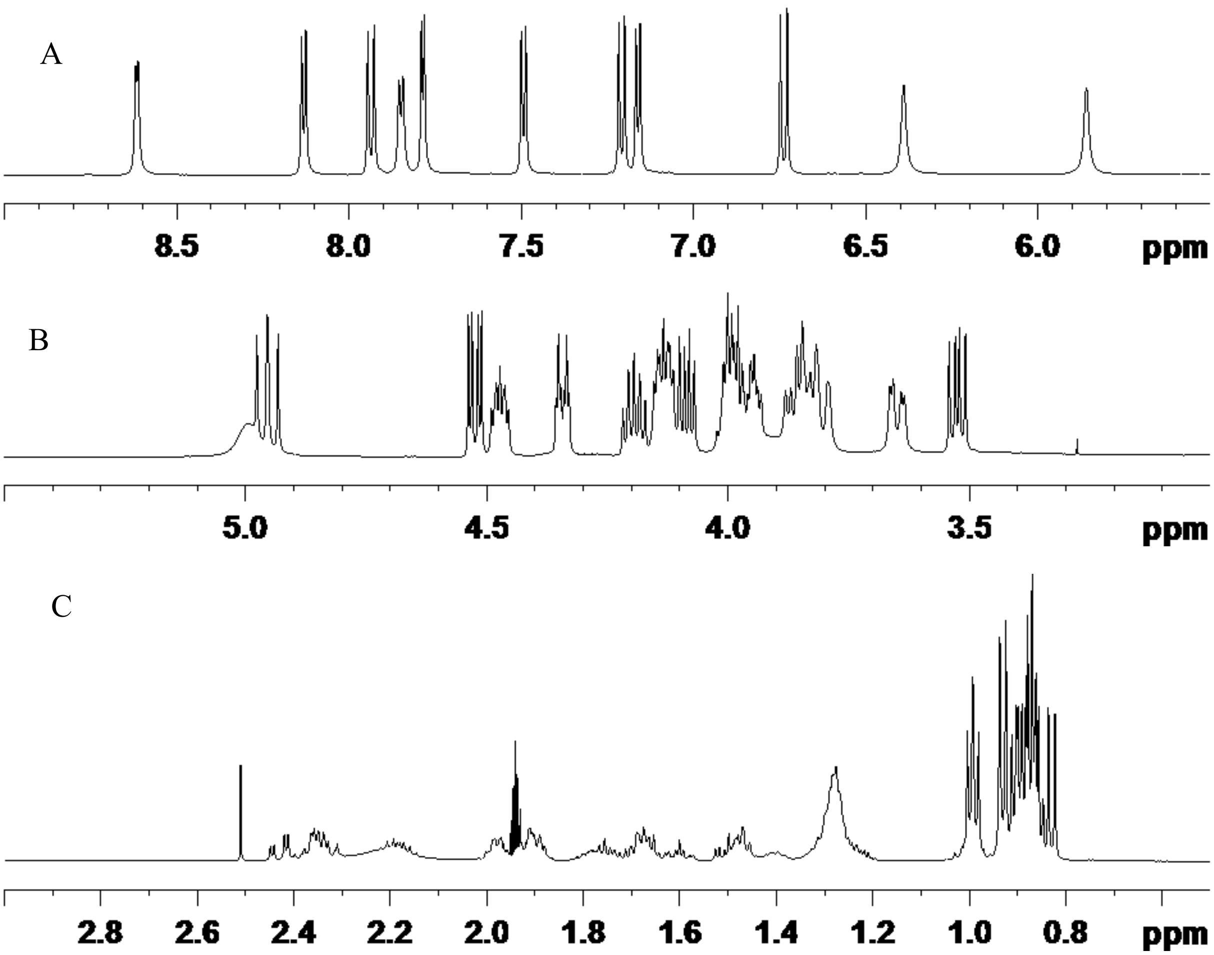

Figure S85. ^1H spectrum of pseudodesmin D-Ser3 (**21**) (CD_3CN, 25°C, 500MHz) A) H^N region, B) H^α region and C) aliphatic region.

Table S22. ^{1}H and ^{13}C assignment of **pseudodesmin D-Ser3** (CD_3CN, 25°C, 500 MHz). Scalar couplings in Hz.

			^{1}H δ [ppm]	^{13}C δ [ppm]
**(*R*)-HDA**				
		CO		174.70
		CH_2 α1	2.34	44.37
		CH_2 α2	2.43	44.37
		CH β	4.00	69.52
		CH_2 γ	1.48	38.04
		CH_2 δ1	1.31	26.26
		CH_2 δ2	1.40	26.26
		CH_2 ε	1.28	29.99
		CH_2 ζ	1.29	30.17
		CH_2 η	1.27	32.54
		CH_2 θ	1.28	23.34
		CH_3 ι	0.88	14.37
		OH	not allocated	
**L-Leu1**				
$^{3}J_{HNH\alpha}$	5.85	NH	7.85	
		CH α	3.84	53.61
		CO		175.36
		CH_2 β1	1.68	39.13
		CH_2 β2	1.76	39.13
		CH γ	1.67	25.37
		CH_3 δ	0.90	22.10
		CH_3 δ	0.93	23.14
**D-Gln2**				
$^{3}J_{HNH\alpha}$	3.64	NH	8.62	
		CH α	3.95	57.52
		CO		176.52
		CH_2 β	1.98	26.34
		CH_2 γ	2.36	31.92
		CO δ		175.86
		NH_2	6.39/5.86	
**D-Ser3**				
$^{3}J_{HNH\alpha}$	6.13	NH	8.13	
		CH α	4.20	56.19
		CO		174.48
		CH_2 β1	4.09	62.49
		CH_2 β2	4.96	62.49
**D-Val4**				
$^{3}J_{HNH\alpha}$	6.33	NH	7.49	
		CH α	3.53	64.90
		CO		174.50
		CH β	2.19	29.99
		CH_3 γ	0.93	19.49
		CH_3 γ	0.99	21.05
**D-Leu5**				
$^{3}J_{HNH\alpha}$	4.31	NH	7.79	
		CH α	3.99	55.61
		CO		173.69
		CH_2 β1	1.50	40.58
		CH_2 β2	1.69	40.58
		CH γ	1.78	25.43
		CH_3 δ	0.88	21.25
		CH_3 δ	0.87	23.30
**D-Ser6**				
$^{3}J_{HNH\alpha}$	8.45	NH	7.21	
		CH α	4.34	56.37
		CO		172.10
		CH_2 β1	3.81	64.68
		CH_2 β2	4.14	64.68
		OH γ	5.00	
**L-Leu7**				
$^{3}J_{HNH\alpha}$	6.10	NH	7.16	
		CH α	4.13	55.02
		CO		174.02
		CH_2 β1	1.61	41.82
		CH_2 β2	1.91	41.82
		CH γ	1.91	25.40
		CH_3 δ	0.90	21.42
		CH_3 δ	1.00	23.43
**D-Ser8**				
$^{3}J_{HNH\alpha}$	9.04	NH	7.94	
		CH α	4.47	56.74
		CO		171.65
		CH_2 β1	3.65	63.18
		CH_2 β2	3.86	63.18
		OH γ	not allocated	
**L-Ile9**				
$^{3}J_{HNH\alpha}$	9.91	NH	6.74	
		CH α	4.53	56.95
		CO		170.41
		CH β	1.91	36.88
		CH_3 γ	0.83	16.18
		CH_2 γ1	1.00	25.33
		CH_2 γ2	1.24	25.33
		CH_3 δ	0.86	12.10

ND= not determined

viscosinamide (22)

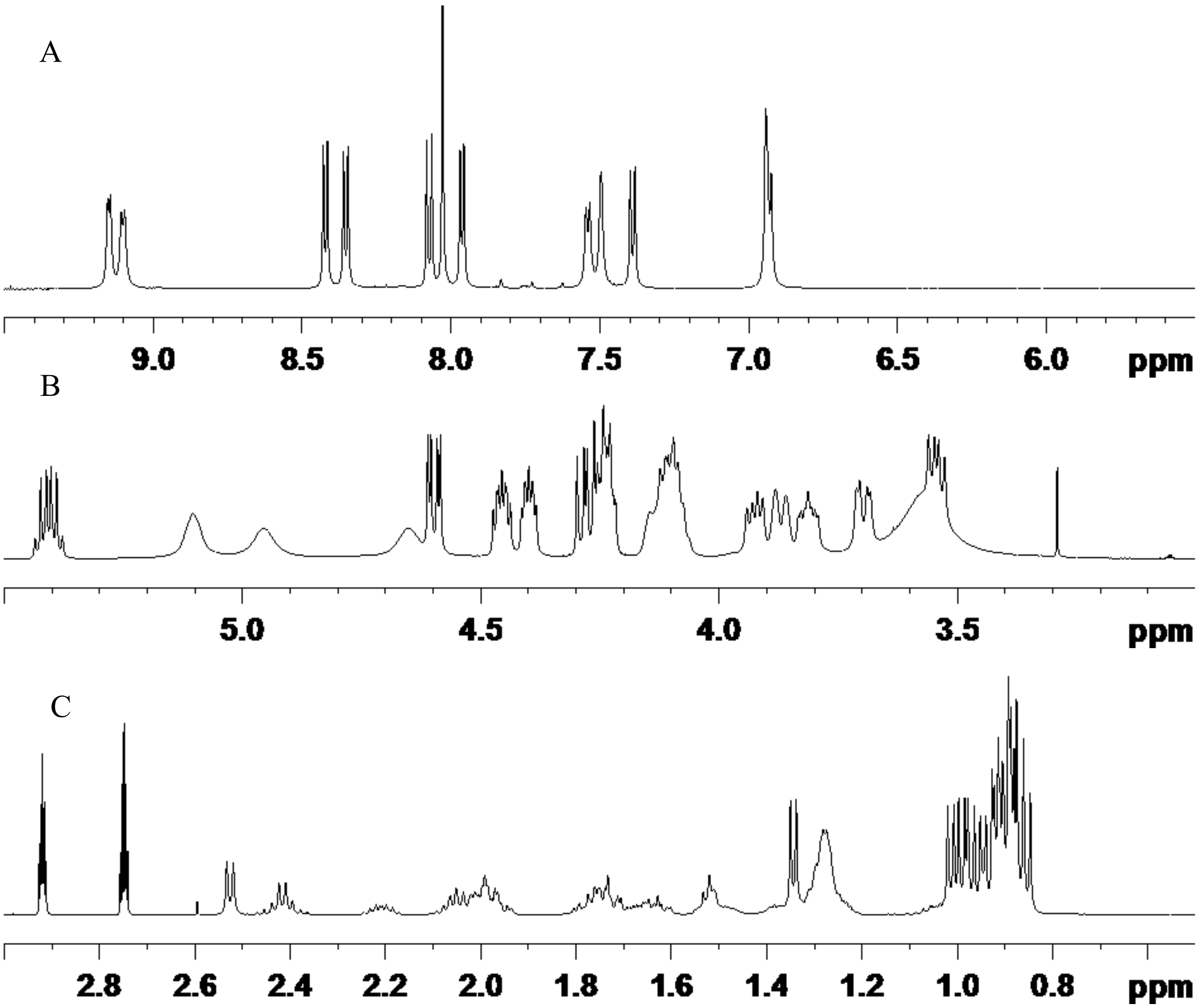

Figure S86. ^{1}H spectrum of synthetic viscosinamide (**22**) (DMF-d7, 25°C, 500MHz) A) H^N region, B) H^α region and C) aliphatic region.

Table S23. ^{1}H and ^{13}C assignment of **viscosinamide** (DMF-d7, 25°C, 500 MHz). Scalar couplings in Hz.

			^{1}H δ [ppm]	^{13}C δ [ppm]				^{1}H δ [ppm]	^{13}C δ [ppm]
**(*R*)-HDA**					**L-Leu5**				
		CO		173.81	$^{3}J_{HNH\alpha}$	6.93	NH	8.42	
		CH_2 α1	2.53	44.17			CH α	3.82	53.00
		CH_2 α2	2.53	44.17			CO		171.11
		CH β	4.09	68.79			CH_2 β1	1.74	37.32
		CH_2 γ	1.52	37.81			CH_2 β2	1.99	37.32
		CH_2 δ1	1.38	25.88			CH γ	1.67	24.93
		CH_2 δ2	1.51	25.88			CH_3 δ	0.89	21.11
		CH_2 ε	1.28	29.53			CH_3 δ	0.92	23.63
		CH_2 ζ	1.29	29.77	**D-Ser6**				
		CH_2 η	1.27	32.00	$^{3}J_{HNH\alpha}$	7.93	NH	7.39	
		CH_2 θ	1.28	22.76			CH α	4.40	56.59
		CH_3 ι	0.88	13.94			CO		171.26
		OH	4.96				CH_2 β1	3.87	63.80
**L-Leu1**							CH_2 β2	4.14	63.80
$^{3}J_{HNH\alpha}$	5.62	NH	9.11				OH γ	5.10	
		CH α	4.10	52.88	**L-Leu7**				
		CO		175.21	$^{3}J_{HNH\alpha}$	6.41	NH	7.54	
		CH_2 β1	1.76	39.34			CH α	4.24	53.71
		CH_2 β2	1.76	39.34			CO		172.89
		CH γ	1.75	24.74			CH_2 β1	1.63	41.28
		CH_3 δ	0.92	21.76			CH_2 β2	1.97	41.28
		CH_3 δ	0.95	22.86			CH γ	2.01	24.64
**D-Gln2**							CH_3 δ	0.89	21.03
$^{3}J_{HNH\alpha}$	4.71	NH	9.15				CH_3 δ	0.99	23.13
		CH α	4.24	56.35	**D-Ser8**				
		CO		175.47	$^{3}J_{HNH\alpha}$	8.56	NH	8.07	
		CH_2 β	2.05	26.72			CH α	4.46	56.58
		CH_2 γ	2.42	31.70			CO		171.05
		CO δ		174.40			CH_2 β1	3.70	62.43
		NH_2	7.50/6.94				CH_2 β2	3.93	62.43
**D-*allo*-Thr3**							OH γ	4.65	
$^{3}J_{HNH\alpha}$	7.45	NH	8.17		**L-Ile9**				
$^{3}J_{H\alpha H\beta}$	10.63	CH α	4.28	60.70	$^{3}J_{HNH\alpha}$	ND	NH	6.94	
		CO		174.12			CH α	4.60	56.44
		CH β	5.41	69.72			CO		169.50
		CH_3 γ	1.35	17.91			CH β	1.99	36.52
**D-Val4**							CH_3 γ	0.85	15.62
$^{3}J_{HNH\alpha}$	6.38	NH	7.96				CH_2 γ1	1.04	24.53
		CH α	3.54	64.48			CH_2 γ2	1.26	24.53
		CO		173.01			CH_3 δ	0.89	11.76
		CH β	2.21	29.37					
		CH_3 γ	0.97	19.17					
		CH_3 γ	1.01	20.39					

ND= not determined

viscosinamide L5I (23)

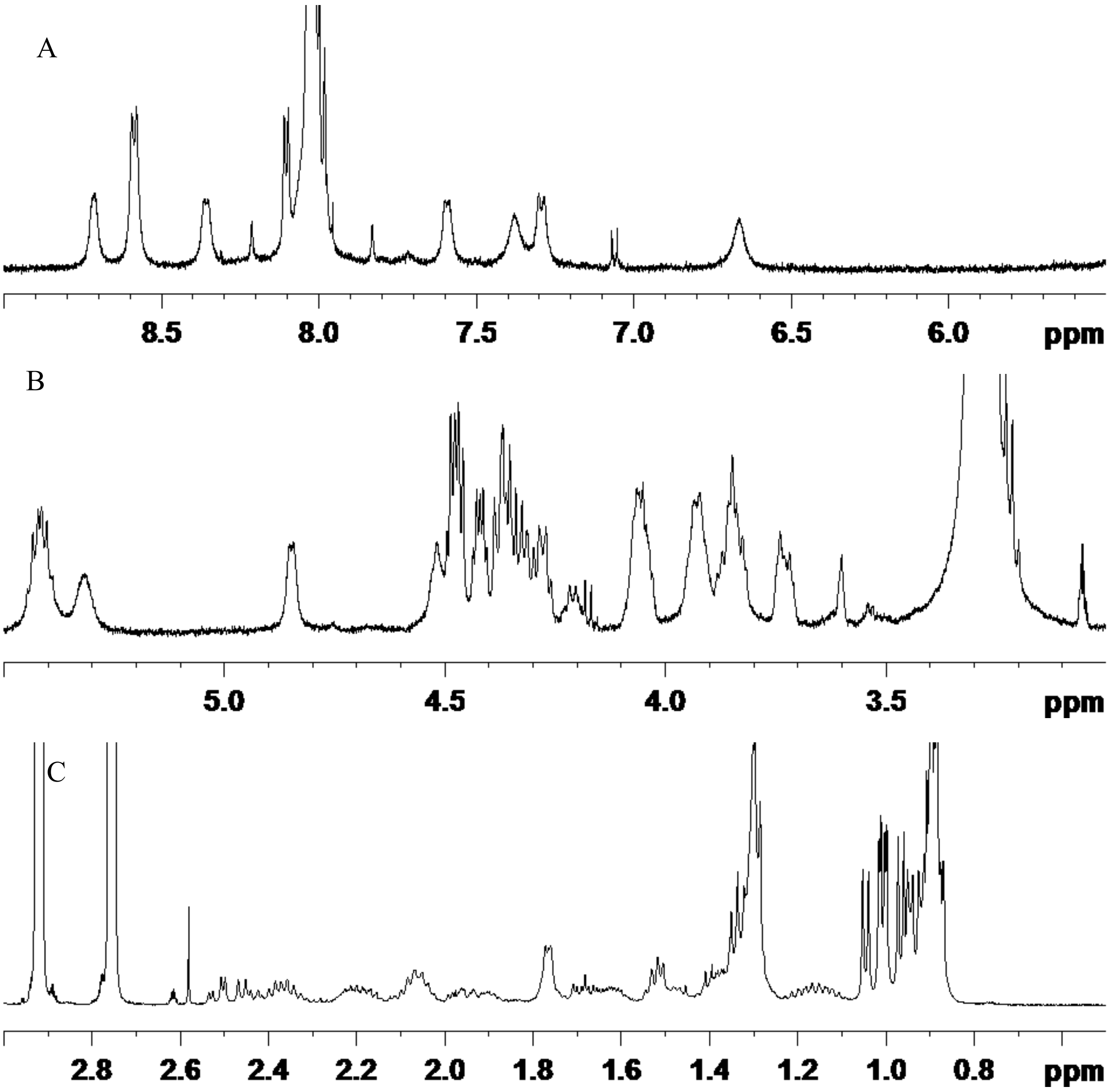

Figure S87. ^{1}H spectrum of viscosinamide L5I (**23**) (DMF-d7, 55°C, 500MHz) A) H^N region, B) H^α region and C) aliphatic region.

Table S24. ^{1}H and ^{13}C assignment of **viscosinamide L5I** (DMF-d7, 55°C, 500 MHz). Scalar couplings in Hz.

			^{1}H δ [ppm]	^{13}C δ [ppm]				^{1}H δ [ppm]	^{13}C δ [ppm]
**(*R*)-HDA**					**L-Ile5**				
		CO		172.84	$^{3}J_{HNH\alpha}$	6.24	NH	8.36	
		CH_2 α1	2.45	44.13			CH α	3.92	58.83
		CH_2 α2	2.52	44.13			CO		not allocated
		CH β	4.06	68.78			CH β	2.21	35.52
		CH_2 γ	1.51	37.80			CH_3 γ	1.00	16.79
		CH_2 δ1	1.38	25.88			CH_2 γ1	1.18	25.56
		CH_2 δ2	1.49	25.88			CH_2 γ2	1.62	25.56
		CH_2 ε	1.30	29.51			CH_3 δ	0.88	11.16
		CH_2 ζ	1.30	29.80	**D-Ser6**				
		CH_2 η	1.29	32.01	$^{3}J_{HNH\alpha}$	ND	NH	7.59	
		CH_2 θ	1.30	22.73			CH α	4.42	56.79
		CH_3 ι	0.89	13.88			CO		not allocated
		OH	4.85				CH_2 β1	3.84	63.86
**L-Leu1**							CH_2 β2	4.05	63.86
$^{3}J_{HNH\alpha}$	ND	NH	8.59				OH γ	5.32	
		CH α	4.32	52.47	**L-Leu7**				
		CO			$^{3}J_{HNH\alpha}$	ND	NH	8.06	
		CH_2 β1	1.77	40.36			CH α	4.36	53.12
		CH_2 β2	1.77	40.36			CO		172.73
		CH γ	1.76	24.92			CH_2 β1	1.68	40.65
		CH_3 δ	0.92	21.65			CH_2 β2	1.96	40.65
		CH_3 δ	0.95	23.08			CH γ	1.90	24.84
**D-Gln2**							CH_3 δ	0.90	21.26
$^{3}J_{HNH\alpha}$	ND	NH	8.72				CH_3 δ	0.96	23.14
		CH α	4.28	55.53	**D-Ser8**				
		CO		not allocated	$^{3}J_{HNH\alpha}$	8.55	NH	7.99	
		CH_2 β	2.07	27.46			CH α	4.48	56.47
		CH_2 γ	2.37	32.01			CO		not allocated
		CO δ		174.58			CH_2 β1	3.73	62.81
		NH_2	7.38/6.67				CH_2 β2	3.85	62.81
**D-*allo*-Thr3**							OH γ	4.52	
$^{3}J_{HNH\alpha}$	ND	NH	8.59		**L-Ile9**				
$^{3}J_{H\alpha H\beta}$	ND	CH α	4.37	59.92	$^{3}J_{HNH\alpha}$	7.88	NH	7.29	
		CO		not allocated			CH α	4.47	57.15
		CH β	5.42	70.30			CO		169.97
		CH_3 γ	1.29	17.65			CH β	2.05	36.46
**D-Val4**							CH_3 γ	0.89	15.72
$^{3}J_{HNH\alpha}$	6.34	NH	8.10				CH_2 γ1	1.14	25.19
		CH α	3.93	62.84			CH_2 γ2	1.37	25.19
		CO		not allocated			CH_3 δ	0.89	11.37
		CH β	2.17	29.82					
		CH_3 γ	1.01	19.46					
		CH_3 γ	1.05	19.88					

ND= not determined

Pseudodesmin NMe1 (24)

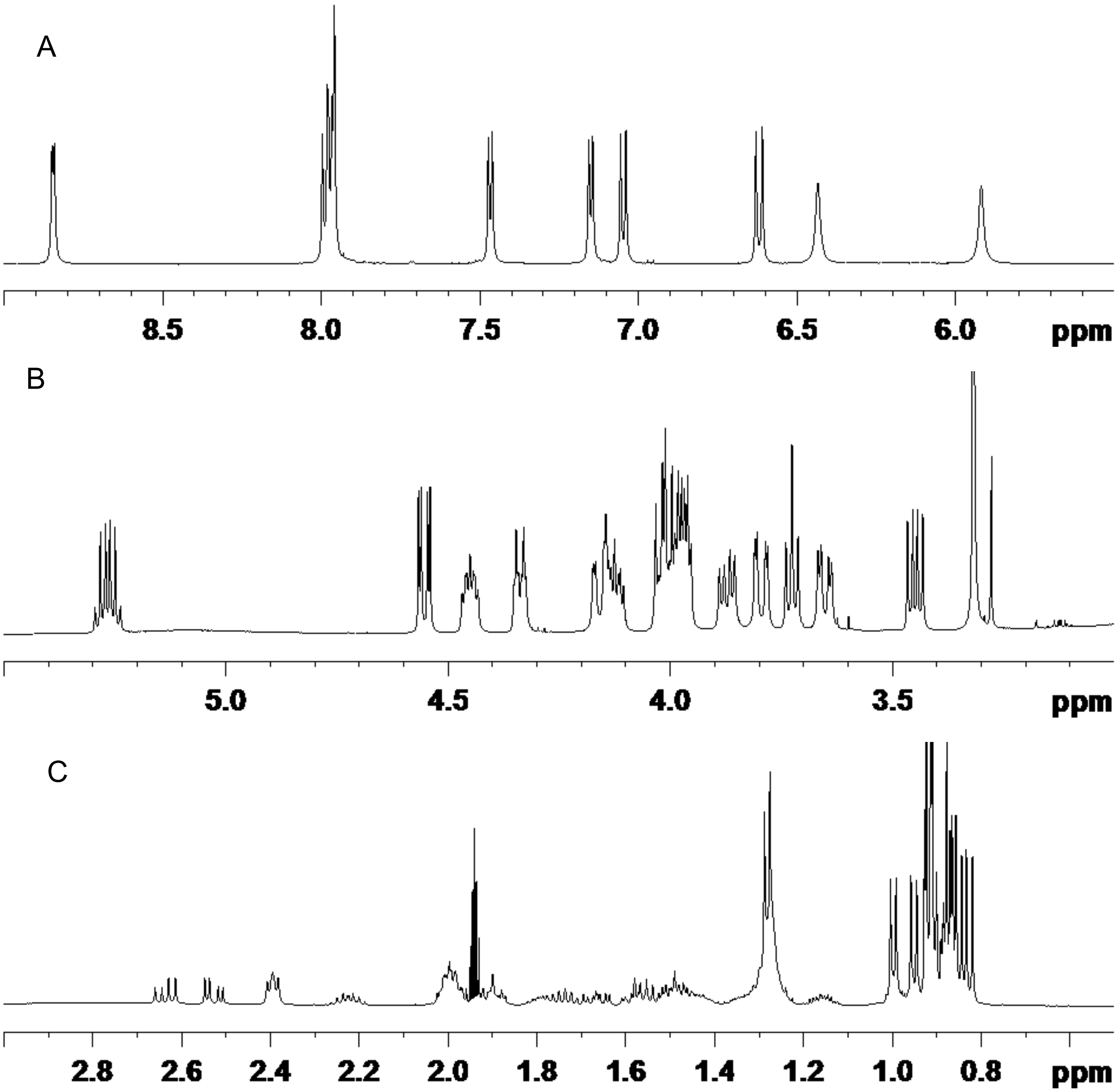

Figure S88. ^{1}H spectrum of pseudodesmin NMe1 (**24**) (CD_3CN, 25°C, 500MHz) A) H^N region, B) H^α region and C) aliphatic region. Asterisks indicate the resonances of an unidentified impurity.

Table S25. ^{1}H and ^{13}C assignment of **pseudodesmin NMe1** (CD_3CN, 25°C, 500 MHz). Scalar couplings in Hz.

			^{1}H δ [ppm]	^{13}C δ [ppm]				^{1}H δ [ppm]	^{13}C δ [ppm]
**(*R*)-HDA**					**D-Leu5**				
		CO		175.10	$^{3}J_{HNH\alpha}$	3.87	NH	7.96	
		CH_2 α1	2.53	42.31			CH α	3.98	55.61
		CH_2 α2	2.64	42.31			CO		173.51
		CH β	4.01	69.79			CH_2 β1	1.50	40.59
		CH_2 γ	1.49	38.24			CH_2 β2	1.67	40.59
		CH_2 δ1	1.33	26.46			CH γ	1.79	25.43
		CH_2 δ2	1.44	26.46			CH_3 δ	0.87	21.12
		CH_2 ε	1.27	30.03			CH_3 δ	0.85	23.36
		CH_2 ζ	1.28	30.31	**D-Ser6**				
		CH_2 η	1.27	32.56	$^{3}J_{HNH\alpha}$	8.56	NH	7.05	
		CH_2 θ	1.28	23.34			CH α	4.34	56.24
		CH_3 ι	0.88	14.37			CO		171.96
		OH	not allocated				CH_2 β1	3.80	64.64
**NMe-L-Leu1**							CH_2 β2	4.16	64.64
		NMe	3.32	40.15			OH γ	not allocated	
		CH α	3.73	63.36	**L-Leu7**				
		CO		173.96	$^{3}J_{HNH\alpha}$	6.12	NH	7.15	
		CH_2 β1	1.56	38.15			CH α	4.13	54.92
		CH_2 β2	2.00	38.15			CO		173.83
		CH γ	1.74	25.66			CH_2 β1	1.58	41.98
		CH_3 δ	0.92	22.52			CH_2 β2	1.90	41.98
		CH_3 δ	0.91	22.87			CH γ	1.90	25.46
**D-Gln2**							CH_3 δ	0.91	21.34
$^{3}J_{HNH\alpha}$	3.74	NH	8.84				CH_3 δ	1.00	23.39
		CH α	3.97	57.62	**D-Ser8**				
		CO		176.71	$^{3}J_{HNH\alpha}$	7.37	NH	7.97	
		CH_2 β	2.00	26.01			CH α	4.45	56.78
		CH_2 γ	2.40	31.90			CO		171.87
		CO δ		not allocated			CH_2 β1	3.65	63.14
		NH_2	6.44/5.92				CH_2 β2	3.87	63.14
**D-*allo*-Thr3**							OH γ	not allocated	
$^{3}J_{HNH\alpha}$	9.47	NH	7.99		**L-Ile9**				
$^{3}J_{H\alpha H\beta}$	10.69	CH α	4.01	61.52	$^{3}J_{HNH\alpha}$	10.15	NH	6.62	
		CO		174.11			CH α	4.55	57.21
		CH β	5.27	70.29			CO		169.91
		CH_3 γ	1.28	18.72			CH β	1.98	36.80
**D-Val4**							CH_3 γ	0.83	16.18
$^{3}J_{HNH\alpha}$	6.07	NH	7.47				CH_2 γ1	0.98	25.22
		CH α	3.45	65.29			CH_2 γ2	1.16	25.22
		CO		174.78			CH_3 δ	0.87	12.28
		CH β	2.22	29.84					
		CH_3 γ	0.92	19.43					
		CH_3 γ	0.95	20.89					

ND= not determined

Pseudodesmin NMe7 (25)

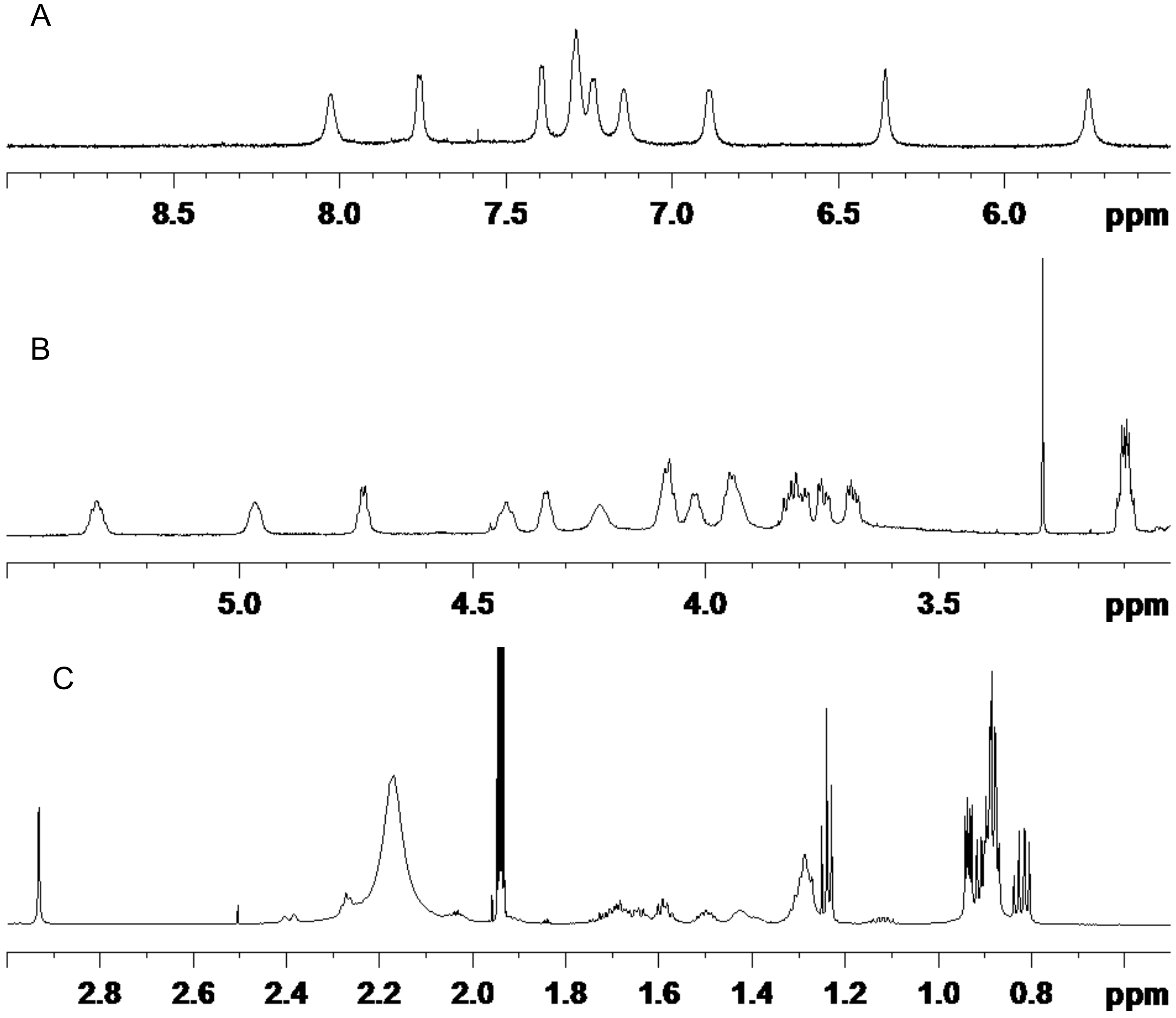

Figure S89. ^{1}H spectrum of pseudodesmin NMe7 (**25**) (CD_3CN, 25°C, 700MHz) A) H^N region, B) H^α region and C) aliphatic region.

Table S26. ^{1}H and ^{13}C assignment of **pseudodesmin NMe7** (CD_3CN, 25°C, 700 MHz). Scalar couplings in Hz.

			^{1}H δ [ppm]	^{13}C δ [ppm]
**(*R*)-HDA**				
		CO		not allocated
		CH_2 α1	2.25	44.03
		CH_2 α2	2.40	44.03
		CH β	3.93	69.35
		CH_2 γ	1.43	38.15
		CH_2 δ1	1.29	26.29
		CH_2 δ2	1.40	26.29
		CH_2 ε	1.29	30.02
		CH_2 ζ	1.29	30.29
		CH_2 η	1.28	32.57
		CH_2 θ	1.30	23.35
		CH_3 ι	0.89	14.36
		OH	not allocated	
**L-Leu1**				
$^{3}J_{HNH\alpha}$	ND	NH	7.28	
		CH α	4.02	53.87
		CO		not allocated
		CH_2 β1	1.59	40.22
		CH_2 β2	1.59	40.22
		CH γ	1.64	25.45
		CH_3 δ	0.89	22.03
		CH_3 δ	0.94	23.12
**D-Gln2**				
$^{3}J_{HNH\alpha}$	ND	NH	8.03	
		CH α	4.09	55.21
		CO		not allocated
		CH_2 β1	1.92	27.12
		CH_2 β2	2.04	27.12
		CH_2 γ	2.27	32.05
		CO δ		not allocated
**D-*allo*-Thr3**		NH_2	5.75/6.36	
$^{3}J_{HNH\alpha}$	6.40	NH	7.76	
		CH α	4.23	59.13
		CO		not allocated
		CH β	5.31	69.79
		CH_3 γ	1.23	17.69
**D-Val4**				
$^{3}J_{HNH\alpha}$	ND	NH	7.24	
		CH α	3.95	60.84
		CO		not allocated
		CH β	2.18	31.04
		CH_3 γ	0.90	18.43
		CH_3 γ	0.88	19.62

			^{1}H δ [ppm]	^{13}C δ [ppm]
**D-Leu5**				
$^{3}J_{HNH\alpha}$	ND	NH	6.89	
		CH α	4.43	51.82
		CO		not allocated
		CH_2 β1	1.59	41.25
		CH_2 β2	1.65	41.25
		CH γ	1.69	25.27
		CH_3 δ	0.88	21.07
		CH_3 δ	0.91	23.60
**D-Ser6**				
$^{3}J_{HNH\alpha}$	ND	NH	7.39	
		CH α	4.34	57.72
		CO		not allocated
		CH_2 β1	3.68	62.48
		CH_2 β2	3.79	62.48
		OH γ	not allocated	
**NMe-L-Leu7**				
		NMe	2.93	32.35
		CH α	4.97	57.09
		CO		not allocated
		CH_2 β1	1.71	36.90
		CH_2 β2	1.71	36.90
		CH γ	1.50	25.42
		CH_3 δ	0.88	21.97
		CH_3 δ	0.93	23.49
**D-Ser8**				
$^{3}J_{HNH\alpha}$	ND	NH	7.15	
		CH α	4.74	53.23
		CO		not allocated
		CH_2 β1	3.75	63.43
		CH_2 β2	3.81	63.43
		OH γ	not allocated	
**L-Ile9**				
$^{3}J_{HNH\alpha}$	ND	NH	7.29	
		CH α	4.08	58.72
		CO		not allocated
		CH β	1.68	36.83
		CH_3 γ	0.81	15.70
		CH_2 γ1	1.12	26.12
		CH_2 γ2	1.50	26.12
		CH_3 δ	0.83	11.43

ND= not determined

pseudodesmin L1W (26)

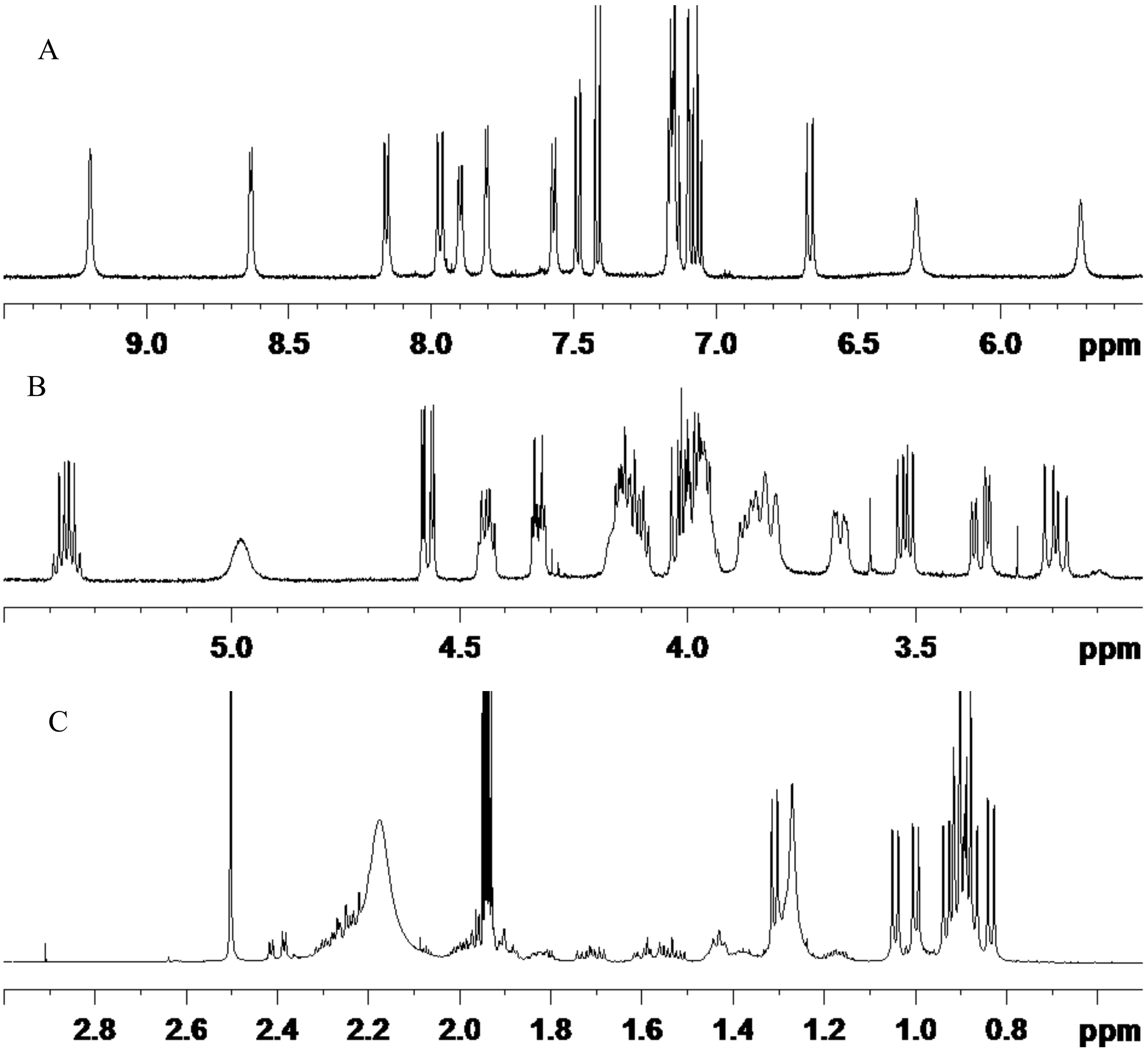

Figure S90. ^{1}H spectrum of pseudodesmin L1W (**26**) (CD_3CN, 25°C, 500MHz) A) H^N region, B) H^{α} region and C) aliphatic region.

Table S27. ^{1}H and ^{13}C assignment of **pseudodesmin L1W** (CD_3CN, 25°C, 500MHz). Scalar couplings in Hz.

			^{1}H δ [ppm]	^{13}C δ [ppm]
**(*R*)-HDA**				
		CO		175.13
		CH_2 α1	2.25	44.65
		CH_2 α2	2.40	44.65
		CH β	3.96	69.51
		CH_2 γ	1.43	38.25
		CH_2 δ1	1.30	26.32
		CH_2 δ2	1.38	26.32
		CH_2 ε	1.27	29.97
		CH_2 ζ	1.27	30.16
		CH_2 η	1.27	32.52
		CH_2 θ	1.27	23.34
		CH_3 ι	0.88	14.35
		OH	not allocated	
**L-Trp1**				
$^{3}J_{HNH\alpha}$	5.80	NH	7.90	
		CH α	4.11	56.48
		CO		174.82
		CH_2 β1	3.19	25.91
		CH_2 β2	3.36	25.91
		NH	9.20	
		2H	7.10	124.42
		4H	7.49	119.02
		5H	7.07	119.93
		6H	7.15	122.52
		7H	7.42	112.44
		3C		111.59
		8C		137.49
		9C		128.44
**D-Gln2**				
$^{3}J_{HNH\alpha}$	3.87	NH	8.63	
		CH α	3.97	57.40
		CO		176.65
		CH_2 β	1.96	26.45
		CH_2 γ	2.25	31.88
		CO δ		not allocated
		NH_2	6.30/5.72	
**D-*allo*-Thr3**				
$^{3}J_{HNH\alpha}$	6.99	NH	8.16	
$^{3}J_{H\alpha H\beta}$	10.69	CH α	4.02	61.75
		CO		174.25
		CH β	5.36	70.23
		CH_3 γ	1.31	18.59
**D-Val4**				
$^{3}J_{HNH\alpha}$	6.23	NH	7.57	
		CH α	3.52	65.16
		CO		174.56
		CH β	2.29	30.02
		CH_3 γ	0.93	19.56
		CH_3 γ	1.04	20.96

			^{1}H δ [ppm]	^{13}C δ [ppm]
**D-Leu5**				
$^{3}J_{HNH\alpha}$	4.04	NH	7.81	
		CH α	4.00	55.69
		CO		173.55
		CH_2 β1	1.54	40.72
		CH_2 β2	1.71	40.72
		CH γ	1.82	25.52
		CH_3 δ	0.89	21.53
		CH_3 δ	0.91	23.23
**D-Ser6**				
$^{3}J_{HNH\alpha}$	ND	NH	7.16	
		CH α	4.33	56.36
		CO		171.91
		CH_2 β1	3.82	64.65
		CH_2 β2	4.14	64.65
		OH γ	4.98	
**L-Leu7**				
$^{3}J_{HNH\alpha}$	ND	NH	7.15	
		CH α	4.14	54.83
		CO		173.81
		CH_2 β1	1.59	41.99
		CH_2 β2	1.90	41.99
		CH γ	1.90	25.44
		CH_3 δ	0.91	21.31
		CH_3 δ	1.00	23.42
**D-Ser8**				
$^{3}J_{HNH\alpha}$	8.93	NH	7.97	
		CH α	4.44	56.90
		CO		171.85
		CH_2 β1	3.67	63.12
		CH_2 β2	3.87	63.12
		OH γ	n.a.	
**L-Ile9**				
$^{3}J_{HNH\alpha}$	10.15	NH	6.67	
		CH α	4.57	57.15
		CO		170.02
		CH β	2.00	36.86
		CH_3 γ	0.83	16.19
		CH_2 γ1	0.99	25.21
		CH_2 γ2	1.18	25.21
		CH_3 δ	0.88	12.28

ND= not determined

pseudodesmin L5W (27)

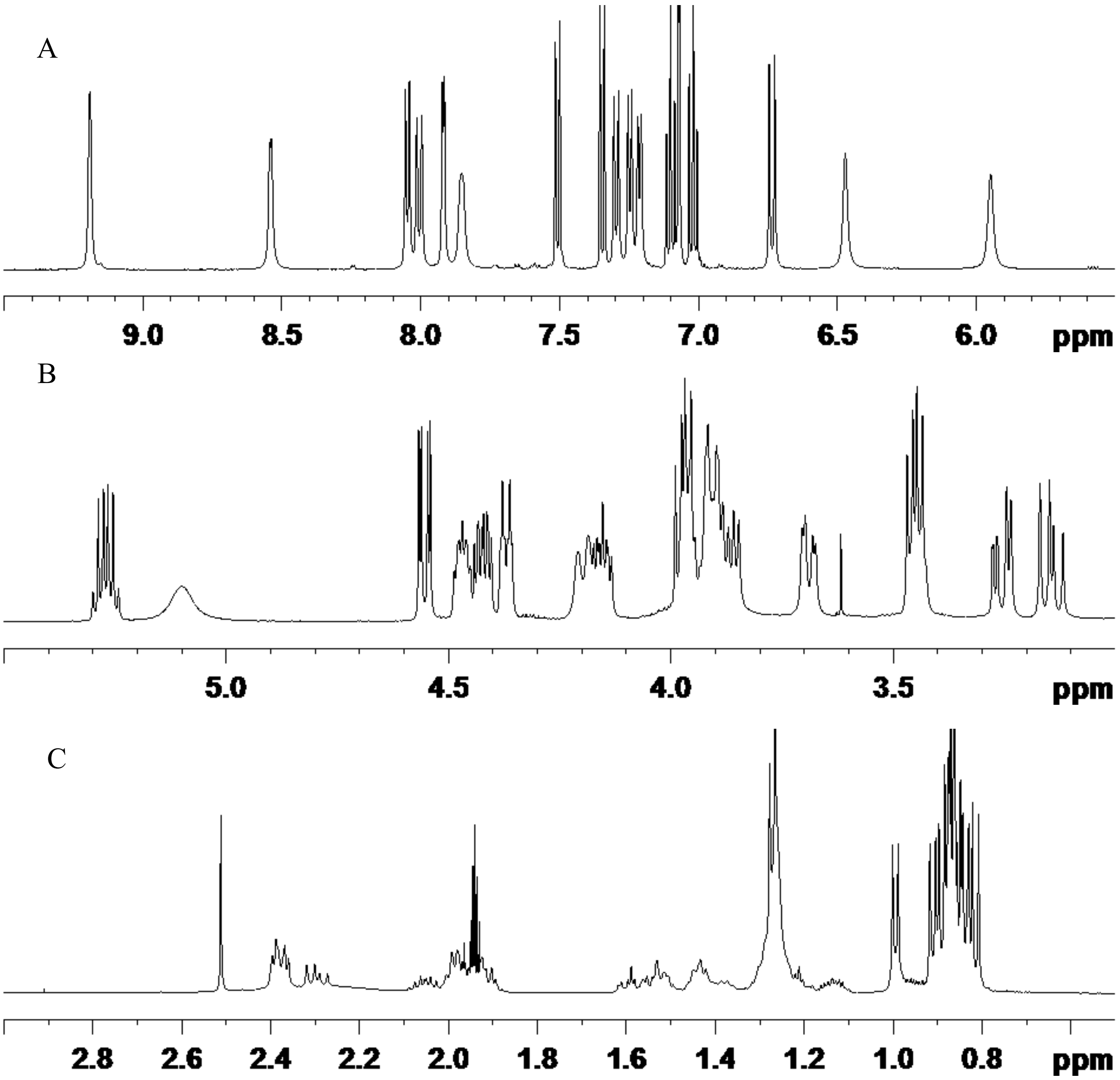

Figure S91. ^{1}H spectrum of pseudodesmin L5W (**27**) (CD_3CN, 25°C, 500MHz) A) H^N region, B) H^{α} region and C) aliphatic region.

Table S28. ^{1}H and ^{13}C assignment of **pseudodesmin L5W** (CD_3CN, 25°C, 500MHz). Scalar couplings in Hz.

			^{1}H δ [ppm]	^{13}C δ [ppm]
**(*R*)-HDA**				
		CO		175.04
		CH_2 α1	2.30	44.69
		CH_2 α2	2.38	44.69
		CH β	3.91	69.45
		CH_2 γ	1.43	38.14
		CH_2 δ1	1.27	26.25
		CH_2 δ2	1.39	26.25
		CH_2 ε	1.26	29.95
		CH_2 ζ	1.26	30.18
		CH_2 η	1.26	32.52
		CH_2 θ	1.26	23.32
		CH_3 ι	0.87	14.36
		OH	not allocated	
**L-Leu1**				
$^{3}J_{HNH\alpha}$	ND	NH	7.85	
		CH α	3.45	53.43
		CO		174.63
		CH_2 β1	1.21	38.37
		CH_2 β2	1.53	38.37
		CH γ	1.52	25.08
		CH_3 δ	0.87	21.57
		CH_3 δ	0.89	23.62
**D-Gln2**				
$^{3}J_{HNH\alpha}$	3.36	NH	8.54	
		CH α	3.96	57.29
		CO		176.75
		CH_2 β	1.99	26.27
		CH_2 γ	2.38	31.87
		CO δ		176.26
		NH_2	6.47/5.95	
**D-*allo*-Thr3**				
$^{3}J_{HNH\alpha}$	7.09	NH	8.05	
$^{3}J_{H\alpha H\beta}$	10.67	CH α	3.97	61.67
		CO		174.19
		CH β	5.27	70.28
		CH_3 γ	1.27	18.49
**D-Val4**				
$^{3}J_{HNH\alpha}$	6.55	NH	7.25	
		CH α	3.45	65.04
		CO		174.86
		CH β	2.05	29.53
		CH_3 γ	0.84	19.44
		CH_3 γ	0.87	20.92

			^{1}H δ [ppm]	^{13}C δ [ppm]
**D-Trp5**				
$^{3}J_{HNH\alpha}$	3.58	NH	7.92	
		CH α	4.42	57.35
		CO		173.08
		CH_2 β1	3.14	27.83
		CH_2 β2	3.26	27.83
		NH	9.19	
		2H	7.07	123.91
		4H	7.51	118.99
		5H	7.02	119.76
		6H	7.10	122.31
		7H	7.35	112.29
		3C		111.01
		8C		137.27
		9C		128.48
**D-Ser6**				
$^{3}J_{HNH\alpha}$	8.31	NH	7.30	
		CH α	4.37	56.44
		CO		172.07
		CH_2 β1	3.91	64.70
		CH_2 β2	4.20	64.70
		OH γ	5.10	
**L-Leu7**				
$^{3}J_{HNH\alpha}$	6.00	NH	7.21	
		CH α	4.15	54.99
		CO		173.95
		CH_2 β1	1.59	41.97
		CH_2 β2	1.92	41.97
		CH γ	1.92	25.46
		CH_3 δ	0.91	21.40
		CH_3 δ	0.99	23.41
**D-Ser8**				
$^{3}J_{HNH\alpha}$	8.96	NH	8.01	
		CH α	4.47	56.99
		CO		171.83
		CH_2 β1	3.69	63.12
		CH_2 β2	3.87	63.12
		OH γ	not allocated	
**L-Ile9**				
$^{3}J_{HNH\alpha}$	10.08	NH	6.74	
		CH α	4.55	57.10
		CO		169.95
		CH β	1.96	36.81
		CH_3 γ	0.82	16.19
		CH_2 γ1	0.96	25.17
		CH_2 γ2	1.14	25.17
		CH_3 δ	0.85	12.24

ND= not determined

pseudodesmin L7W (28)

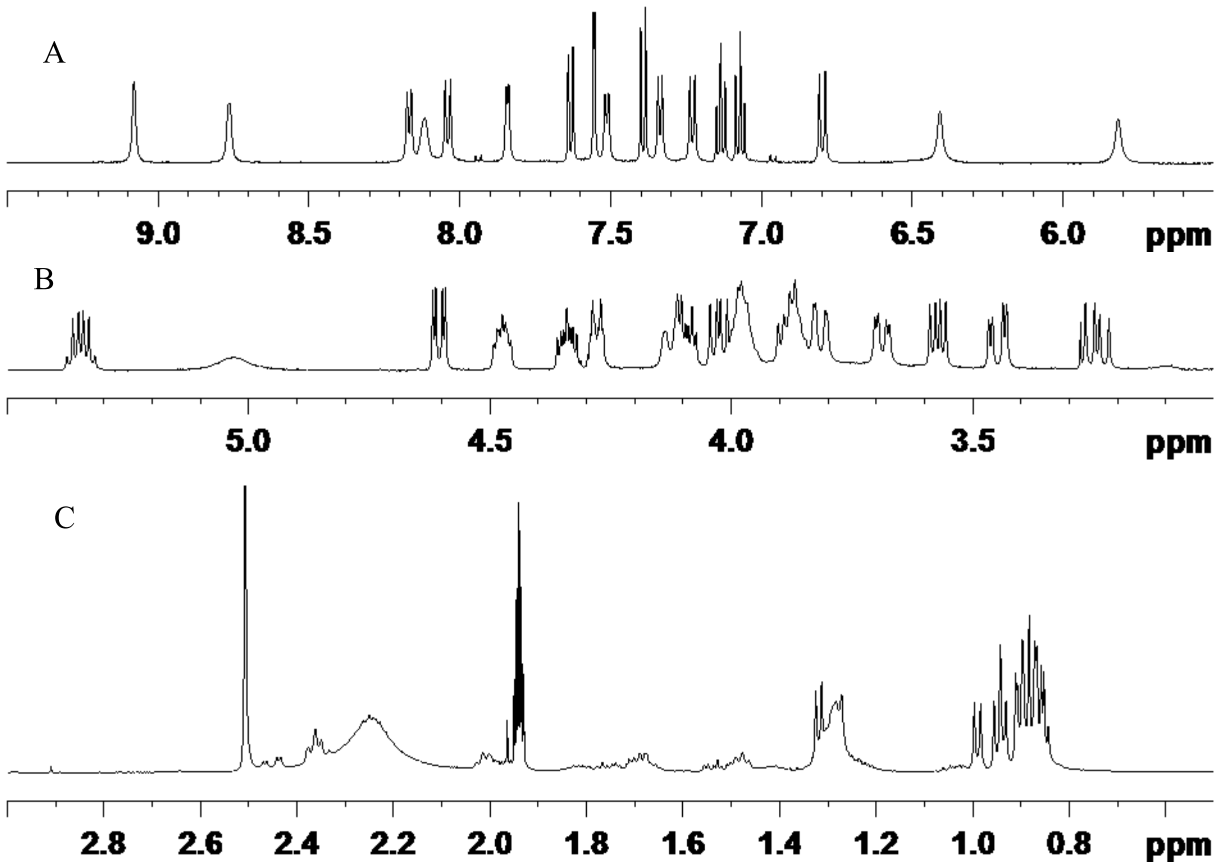

Figure S92. ^{1}H spectrum of pseudodesmin L7W (**28**) (CD_3CN, 25°C, 500MHz) A) H^N region, B) H^{α} region and C) aliphatic region.

Table S29. ^{1}H and ^{13}C assignment of **pseudodesmin L7W** (CD_3CN, 25°C, 500MHz). Scalar couplings in Hz.

			^{1}H δ [ppm]	^{13}C δ [ppm]
**(*R*)-HDA**				
		CO		not allocated
		CH_2 α1	2.36	44.67
		CH_2 α2	2.46	44.67
		CH β	3.97	69.66
		CH_2 γ	1.49	38.25
		CH_2 δ1	1.31	26.32
		CH_2 δ2	1.42	26.32
		CH_2 ε	1.29	29.98
		CH_2 ζ	1.28	30.21
		CH_2 η	1.28	32.53
		CH_2 θ	1.29	23.34
		CH_3 ι	0.88	14.36
		OH	not allocated	
**L-Leu1**				
$^3J_{HNHα}$		NH	8.12	
		CH α	3.87	53.54
		CO		175.27
		CH_2 β1	1.69	39.20
		CH_2 β2	1.77	39.20
		CH γ	1.69	25.37
		CH_3 δ	0.91	22.03
		CH_3 δ	0.94	23.20
**D-Gln2**				
$^3J_{HNHα}$	ND	NH	8.78	
		CH α	3.98	57.53
		CO		176.74
		CH_2 β	2.01	26.59
		CH_2 γ	2.36	32.07
		CO δ		175.74
		NH_2	6.42/5.80	
**D-*allo*-Thr3**				
$^3J_{HNHα}$	7.01	NH	8.18	
$^3J_{HαHβ}$	10.70	CH α	4.03	61.89
		CO		174.38
		CH β	5.35	70.24
		CH_3 γ	1.32	18.56
**D-Val4**				
$^3J_{HNHα}$	5.95	NH	7.53	
		CH α	3.57	65.31
		CO		174.83
		CH β	2.23	29.99
		CH_3 γ	0.95	19.41
		CH_3 γ	0.99	20.99

			^{1}H δ [ppm]	^{13}C δ [ppm]
**D-Leu5**				
$^3J_{HNHα}$	4.36	NH	7.86	
		CH α	4.09	55.54
		CO		173.60
		CH_2 β1	1.53	40.62
		CH_2 β2	1.71	40.62
		CH γ	1.82	25.47
		CH_3 δ	0.91	21.16
		CH_3 δ	0.88	23.34
**D-Ser6**				
$^3J_{HNHα}$	8.35	NH	7.23	
		CH α	4.28	56.46
		CO		171.98
		CH_2 β1	3.82	64.48
		CH_2 β2	4.13	64.48
		OH γ	5.04	
**L-Trp7**				
$^3J_{HNHα}$	6.64	NH	7.34	
		CH α	4.34	57.63
		CO		173.13
		CH_2 β1	3.24	28.40
		CH_2 β2	3.45	28.40
		NH	9.08	
		2H	7.39	112.30
		4H	7.63	119.00
		5H	7.07	119.85
		6H	7.14	122.33
		7H	7.55	125.21
		3C		112.30
		8C		137.31
		9C		128.13
**D-Ser8**				
$^3J_{HNHα}$	8.70	NH	8.04	
		CH α	4.48	57.31
		CO		171.75
		CH_2 β1	3.69	63.24
		CH_2 β2	3.89	63.24
		OH γ	not allocated	
**L-Ile9**				
$^3J_{HNHα}$	9.99	NH	6.80	
		CH α	4.61	57.07
		CO		169.98
		CH β	1.96	37.10
		CH_3 γ	0.86	16.13
		CH_2 γ1	1.03	25.29
		CH_2 γ2	1.23	25.29
		CH_3 δ	0.86	12.35

ND= not determined

Creation of the sequence logo

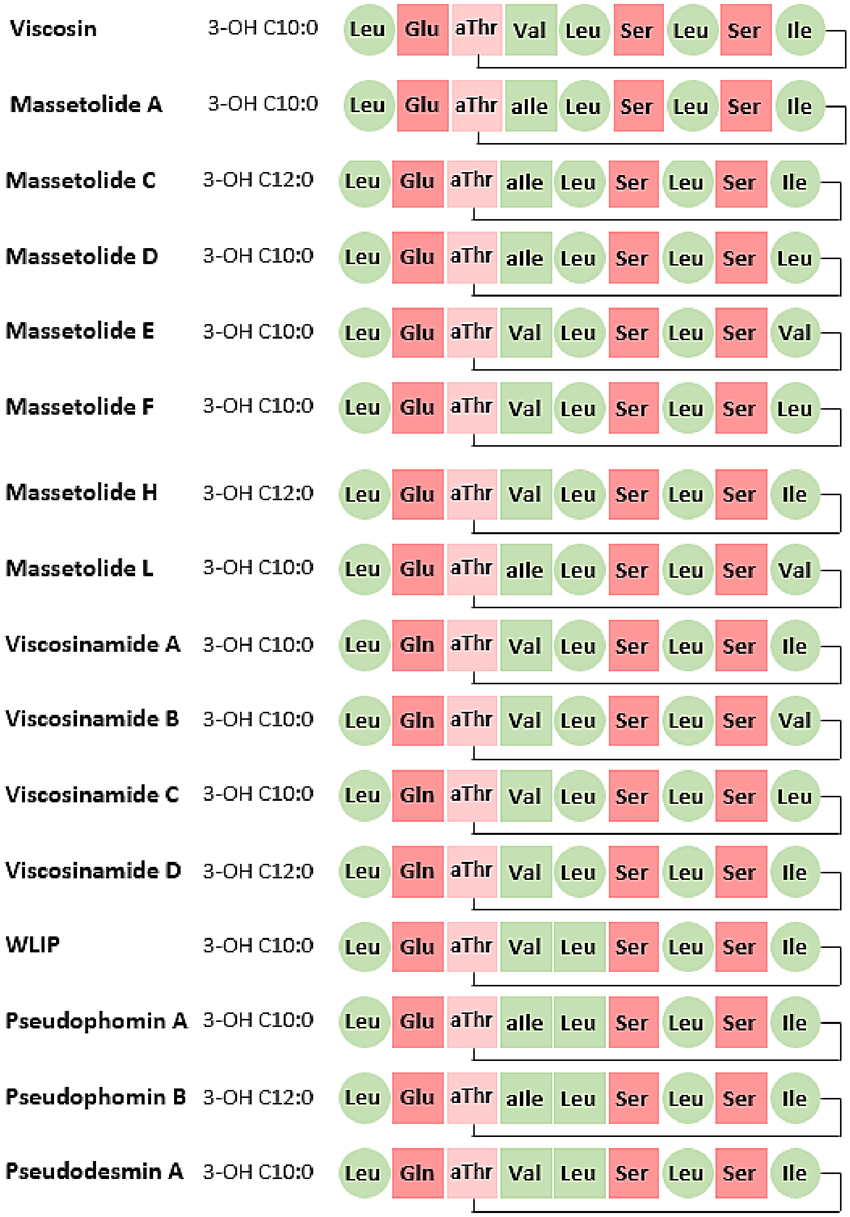

Figure S93: Overview of viscosin group sequences used for the sequence logo creation in Figure 1B. Green and red indicate hydrophobic and hydrophilic residues, while circles and squares indicate D/L stereochemistry. However, in many cases this has not been explicitly proven, therefore this information was not used for logo generation.

frontiers

References

De Vleeschouwer, M., Sinnaeve, D., Matthijs, N., Coenye, T., Madder, A., and Martins, J. C. (2017). Synthesis of N-Methylated Pseudodesmin A Analogues: on the Structural Importance of N-H Hydrogen Bonds. *ChemistrySelect* 2, 640–644. doi:10.1002/slct.201601791.

De Vleeschouwer, M., Martins, J. C., and Madder, A. (2016). First total synthesis of WLIP: On the importance of correct protecting group choice. *J. Pept. Sci.* 22, 149–155. doi:10.1002/psc.2852.

De Vleeschouwer, M., Sinnaeve, D., Van Den Begin, J., Coenye, T., Martins, J. C., and Madder, A. (2014). Rapid total synthesis of cyclic lipodepsipeptides as a premise to investigate their self-assembly and biological activity. *Chem. - A Eur. J.* 20, 7766–7775. doi:10.1002/chem.201402066.

Jiang, H., Gschwend, B., Albrecht, Ł., and Anker Jørgensen, K. (2010). Organocatalytic preparation of simple β-hydroxy and β-amino esters: Low catalyst loadings and gram-scale synthesis. *Org. Lett.* 12, 5052–5055. doi:10.1021/ol102164y.

Kücük, H. B., and Yusufoğlu, A. (2013). Enantioselective synthesis of 3-hydroxytetradecanoic acid and its methyl ester enantiomers as new antioxidants and enzyme inhibitors. *Monatshefte fur Chemie* 144, 1087–1091. doi:10.1007/s00706-012-0917-z.

Pirrung, M. C., Zhang, F., Ambadi, S., and Gangadhara Rao, Y. (2016). Total synthesis of fellutamides, lipopeptide proteasome inhibitors. More sustainable peptide bond formation. *Org. Biomol. Chem.* 14, 8367–8375. doi:10.1039/c6ob01233g.

Rohwedder, B., Mutti, Y., Dumy, P., and Mutter, M. (1998). Hydrazinolysis of Dde: Complete orthogonality with Aloc protecting groups. *Tetrahedron Lett.* 39, 1175–1178. doi:10.1016/S0040-4039(97)10810-3.
